# Effects of Danhong Injection on 90‐Day Functional Outcome in Acute Ischemic Stroke: An Adaptive, Multicenter, Randomized, Double‐Blind, Placebo‐Controlled Phase IV Trial

**DOI:** 10.1002/mco2.71135

**Published:** 2026-11-08

**Authors:** Jun Liu, Yilong Wang, Bingwei Chen, Jingjing Lu, Lin Chen, Shuang Guan, Yinli Shi, Wei Liu, Yanan Yu, Bing Li, Haixia Dang, Xiaolong Zhu, Fei Teng, Xuanzhu Liu, Niwen Zhou, Qiguang Chen, Chunti Shen, Lixing Zhu, Yongyan Wang, Xingquan Zhao, Zhong Wang, Danhong‐Stroke Trial Investigators

**Affiliations:** ^1^ Institute of Basic Research in Clinical Medicine China Academy of Chinese Medical Sciences Beijing China; ^2^ Department of Neurology, Beijing Tiantan Hospital Capital Medical University Beijing China; ^3^ China National Clinical Research Center For Neurological Diseases Beijing China; ^4^ School of Public Health Southeast University Nanjing Jiangsu China; ^5^ Department of Cardiology Xin Hua Hospital of Zhejiang Province Hangzhou Zhejiang China; ^6^ Beijing Key Laboratory of Traditional Chinese Medicine Basic Research On Prevention and Treatment for Major Diseases, Experimental Research Center China Academy of Chinese Medical Sciences Beijing China; ^7^ Institute of Chinese Materia Medica China Academy of Chinese Medical Sciences Beijing China; ^8^ China Academy of Chinese Medical Sciences Beijing China; ^9^ Beijing Genomics Institute Shenzhen Guangdong China; ^10^ Department of Statistics Beijing Normal University Zhuhai China; ^11^ Changzhou TCM Hospital Changzhou Jiangsu China

**Keywords:** Danhong injection, ischemic stroke, modular pharmacology, multitarget drug, RNA sequencing

## Abstract

Therapeutic options for neurological recovery remain limited in acute ischemic stroke (AIS) patients ineligible for thrombolysis, and while multitarget drugs like Danhong injection (*DHI*) demonstrate efficacy, their polypharmacological mechanisms remain understood. In this trial including 1505 thrombolysis‐ineligible AIS patients with anterior‐circulation and blood stasis syndrome (NCT01677208), 14‐day treatment with *DHI* (*n* = 1002) versus placebo (*n* = 503) demonstrated superior 90‐day outcomes (modified Rankin scale [mRS] score ≤1: 64.57 vs. 59.24%; adjusted odds ratio 1.43, 95%CI 1.10–1.86, *p *= 0.007), particularly in females and hypertensive patients, and no significant between‐group differences in any serious adverse events (all *p* > 0.05). In the exploratory RNA‐sequencing substudy of 39 patients (24 *DHI*, 15 placebo), we identified 19 differentially expressed modules, including two paired targeted modules “*HBG2*” and “*TMEM108*,” whose topological features showed opposite correlations with the primary clinical outcome (δmRS at Day 90). Furthermore, a combination effect model was introduced to describe the time‐dependent joint association of these modules with outcome (combination effect score: −0.729 at Day 0, 0.227 at Day 14, and 1 at Day 90). These findings demonstrate *DHI* may be a favorable treatment option for thrombolysis‐ineligible AIS patients and provide a proposed analytical framework for future, independently validated studies of multitarget drug actions in complex diseases.

## Graphical abstract

In a randomized trial of 1505 thrombolysis‐ineligible AIS patients, 14‐day Danhong injection improved neurological recovery, raising mRS ≤1 rate by 5.33% versus placebo. RNA sequencing of 39 patients identified 19 DEMs, including the paired targeted modules *HBG2/TMEM108*, whose time‐dependent joint association with outcome (−0.729 to 1) was described using an exploratory combination effect model.

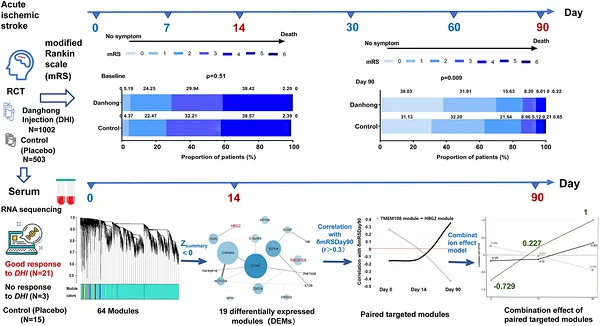

## 1 Introduction

Acute ischemic stroke (AIS) accounts for the majority of strokes, and timely intravenous thrombolysis remains the principal approved reperfusion therapy; however, its use is constrained by narrow time windows, strict imaging eligibility, and the risk of hemorrhagic transformation [1, 2]. Many patients arrive beyond the time window or are otherwise ineligible for thrombolysis; for these patients, guidelines recommend antiplatelet agents (e.g., aspirin or clopidogrel) or anticoagulants (e.g., apixaban for atrial fibrillation), mainly to prevent recurrent ischemic events [3, 4, 5], and none directly promotes neurological recovery or reliably improves functional outcomes. Neuroprotective candidates such as nerinetide and edaravone have shown promise preclinically but have failed or produced inconsistent results in pivotal trials [6, 7], possibly because AIS involves multifactorial, interconnected pathology‐excitotoxicity, oxidative stress, thrombo‐inflammation, blood–brain barrier disruption, and impaired neuroplasticity [8]‐which favors multicomponent strategies over single‐pathway monotherapy [9]. Identifying the multiple targets of such strategies is challenging, but module‐based network methods deconstruct complex pharmacological networks into functionally coherent units, offering a systematic way to study drug actions [10]; targeted modules of multitarget drugs have distinct biological functions [11], although whether modules act in a coordinated, complementary manner to achieve a therapeutic effect has not been examined clinically.

Danhong injection (*DHI*) (Shandong Danhong Pharmaceutical Co., Ltd.; HPLC‐validated batch consistency >90%) is a natural product extracted from *Radix Salviae miltiorrhizae* (Danshen) and *Flos Carthami tinctorii* (*Honghua*), containing five main active compounds: *Danshensu sodium*, *protocatechualdehyde*, *p‐coumaric acid*, *rosmarinic acid*, and *salvianolic acid B* (Figures S1 and S2) [11]. *DHI* demonstrates dose‐dependent neuroprotective effects across multiple mechanisms, including anti‐inflammatory activity, apoptosis suppression, metabolic modulation, and neuroprotection in ischemic stroke [12, 13, 14, 15]. Mechanistically, *DHI* facilitates microglial M2 polarization through JUNB/NF‐κB inhibition to attenuate neuroinflammation [16], downregulates autophagy markers such as ATG12 and LC3‐II/I [17], enhances neuroplasticity via BDNF/AKT/CREB pathway activation [18], and regulates metabolism modules involving Glu–GLT1/Gly–Glyα and glutamine/glutamate metabolism in rodent models [13, 14].

Clinically, a previous registry study suggested that 14‐day *DHI* treatment (40 mL/day) is associated with significant NIHSS score improvements in AIS patients [19], and a meta‐analysis reported benefits in neurological function, self‐care ability, and lipid levels when *DHI* was combined with conventional therapies [20]. However, these studies had important methodological limitations, including unclear risk of bias and a narrow mechanistic focus on isolated pathways or single modules in animal models. Thus, the multitarget actions of *DHI* in AIS patients and their relationship to clinical recovery remain poorly understood. Rigorous, adequately powered randomized evidence combined with molecular profiling within the same population is needed to establish *DHI's* efficacy and safety and generate testable hypotheses about its polypharmacology.

We therefore conducted an adaptive, multicenter, randomized, double‐blind, placebo‐controlled Phase IV trial (NCT01677208) in thrombolysis‐ineligible patients with anterior‐circulation AIS and blood stasis syndrome (*Xueyu Zheng*). It assessed 14‐day *DHI* versus placebo on functional outcome at 90 days and safety outcomes, and integrated RNA sequencing in a prespecified subset to identify genes and modules associated with treatment response. The molecular module analyses, including a novel combination effect model of the targeted modules, were designed to quantify associations between transcriptomic modules and clinical outcomes for elucidating the potential polypharmacological mechanisms underlying its clinical effects.

## 2 Results

### 2.1 Interim Analysis Results

We conducted two interim analyses during our trial using the O'Brien‐Fleming boundary approach (Table S2). At the first interim analysis, 282 patients completed the trial. In the *DHI* group, 106 out of 188 patients (56.50%) achieved excellent functional outcome (an mRS score of 0–1) compared with 46 out of 94 patients (48.94%) in the placebo group (*Z*_1_ = 1.19, *p*_1 _= 0.23) (Table S3). According to the recommendation of data monitoring committee (DMC), the trial continued without sample size re‐estimation (CP = 0.55). At the second interim analysis, 564 patients completed the trial. In the *DHI* group, 60.94% of patients achieved excellent functional outcome (an mRS score of 0–1) compared with 54.82% of patients in the placebo group (*Z*_2 _= 1.41, *p*_2 _= 0.16) (Table S3). According to the recommendation of DMC (CP = 0.34), the sample size was re‐estimated to be 1513 patients, accounting for potential dropouts and losses to follow‐up.

### 2.2 Patient Disposition of the Trial

Of the 2506 individuals assessed for eligibility (Table S4) between March 13, 2013 and March 27, 2019, in 72 participating centers across China, of which 62 centers enrolled patients (10 activated centers withdrew before enrollment and contributed no data; Table S1), 1505 patients were randomly assigned to receive *DHI* (1002 patients) or placebo (503 patients) (Figure 1A). Twelve patients (0.80%; eight in the *DHI* group and four in the placebo group) did not receive treatment, and 128 (8.50%; 90 in the *DHI* group and 38 in the placebo group) did not complete the trial (Figure 1A). Baseline characteristics were similar between the two groups, except for a history of hypertension (*p *= 0.008) or hyperlipidemia (*p *= 0.005) (Table 1). The mean age of the cohort was 59 years; 451 (29.97%) patients were female, 984 (65.38%) had hypertension, 107 (7.11%) had a stroke or transient ischemic attack, and 855 (57.15%) had right hemisphere stroke. The median baseline NIHSS score was 5 (interquartile range, 4–7), and the time from stroke onset to randomization was 26 h (interquartile range, 11–65). The median time from stroke onset to the administration of *DHI* was 79 h (interquartile range, 51–121). The use of concomitant medications was not significantly different between the two groups (*p *> 0.05) except for calcium channel blockers (CCBs) (*p *= 0.045) (Table S5). The number of patients who took aspirin, clopidogrel, statins, CCBs, or neuroprotective drugs (edaravone or oxiracetam) was 1313 (87.24%), 1018 (67.64%), 1336 (88.77%), 722 (47.97%), and 1299 (86.31%), respectively.

**FIGURE 1:**
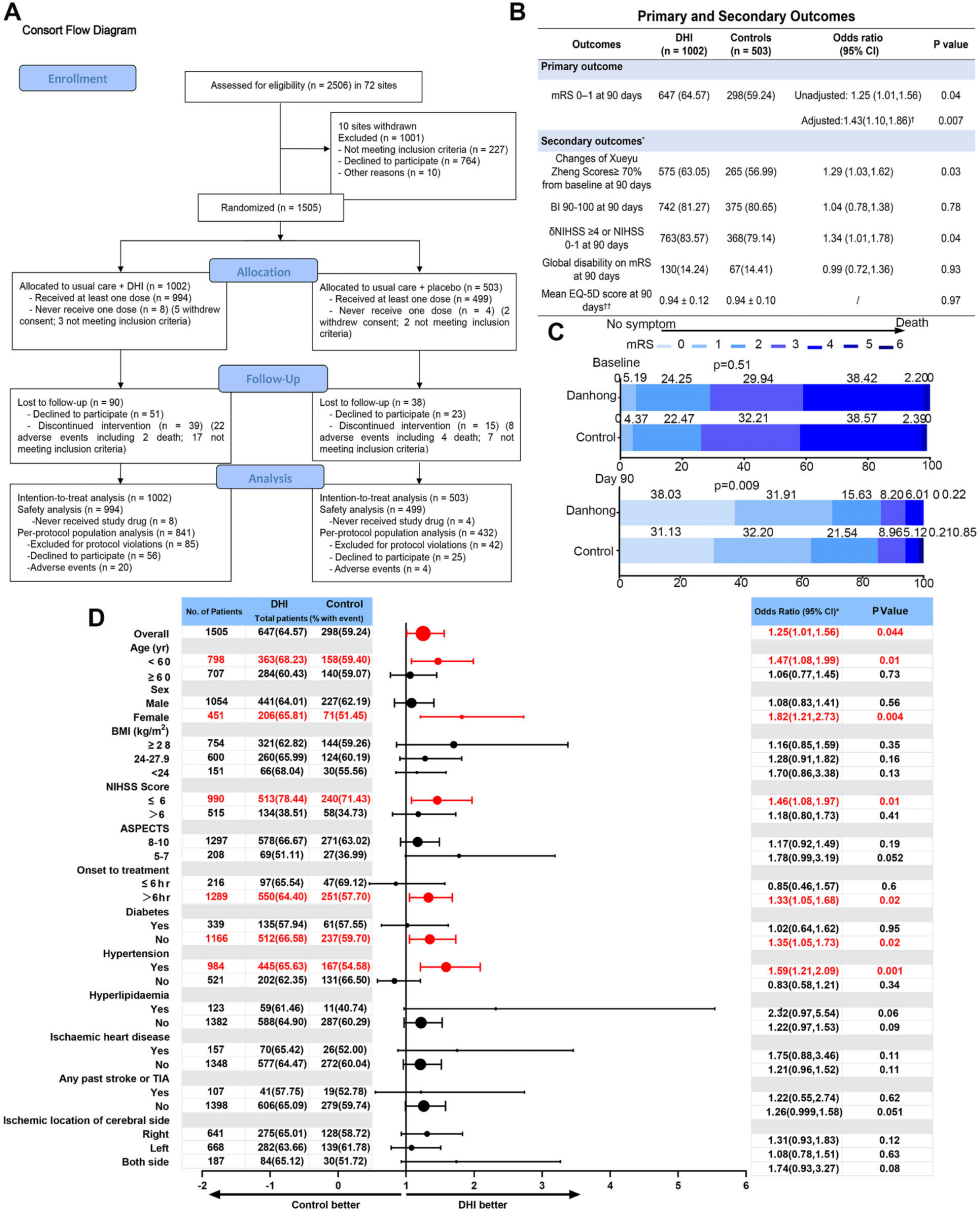
Effects of *DHI* on the neurological recovery of patients with acute ischemic stroke. (A) CONSORT flow diagram of participant enrollment and allocation, follow‐up, and statistical analysis. (B) Primary and secondary outcomes. Data are numbers and percentages or means ± standard deviations. ^†^The odds ratio was adjusted for age, mRS scores, NIHSS scores, ASPECTS scores, time from stroke onset to randomization, atrial fibrillation based on ECG signals, and a history of stroke, TIA, hypertension, hyperlipidemia, diabetes mellitus, and ischemic heart disease. ^*^The secondary outcomes were calculated without imputation, and the odds ratios of the secondary outcomes were not adjusted. (C) mRS in the control and *DHI* group at baseline and 90 days. (D) Effect of treatment by subgroups as a function of demographic and clinical characteristics. ^*^ *p* Values were not adjusted for multiplicity. *Abbreviations*: ASPECTS: Alberta Stroke Program Early CT Score; BI: Barthel index; BMI: body mass index; *DHI*: Danhong injection; ECG: electrocardiogram; EQ‐5D: EuroQoL group 5‐dimension (a standardized questionnaire for measuring health status, with scores ranging from 0.1702 [lowest] to 1.00 [highest]) [41]; mRS: modified Rankin scale; NIHSS: National Institutes of Health Stroke Scale; TIA: transient ischemic attack.

**TABLE 1:** Baseline characteristics of study participants.^a^

Characteristics	*DHI* group (*n* = 1002)	Control group (*n* = 503)
**Age (years)**	59.4 (52.68–64.38)	59.12 (52.7–64.3)
**Sex (%)**		
Men	689 (68.76)	365 (72.56)
Women	313 (31.24)	138 (27.44)
**Race (%)**		
Han	986 (98.40)	495 (98.41)
Others	16 (1.60)	8 (1.59)
BMI (kg/m^2^)^b^	23.88 (22.5–25.76)	24.07 (22.1–25.78)
**Medical history (%)**		
Hypertension	678 (67.66)	306 (60.83)
Hyperlipidemia	96 (9.58)	27 (5.37)
Ischemic heart disease	107 (10.68)	50 (9.94)
Diabetes	233 (23.25)	106 (21.07)
Past stroke/TIA	71 (7.09)	36 (7.16)
**Clinical factors**		
Right hemisphere stroke	572 (57.49)	283 (56.49)
Baseline NIHSS scores	5 (4–8)	5 (4–7)
Baseline mRS ≥ 2 (%)	950 (94.81%)	481 (95.63%)
Baseline *Xueyu Zheng* Score^c^	21 (14–31)	22 (15–30)
Baseline EQ‐5D Scale^d^	0.81 (0.53–0.84)	0.81 (0.57–0.84)
Systolic blood pressure (mmHg)	145 (132–160)	143 (130–160)
Diastolic blood pressure (mmHg)	88 (80–95)	89 (80–96)
Heart rate (beat/min)	75 (70–80)	76 (70–80)
ECG showing atrial fibrillation at baseline‐no. (%)	11 (1.10)	3 (0.60)
ASPECTS (core laboratory determined)	9 (8–9)	9 (8–9)
**Treatment and workflow**		
Aspirin before randomization—no. (%)	526 (52.50)	259 (51.49)
Clopidogrel before randomization—no. (%)	395 (39.42)	194 (38.59)
Time to randomization after onset of symptoms (h)	26 (11–69)	26 (12–59)

*Abbreviations*: *DHI*: Danhong injection; BMI: body mass index; TIA: transient ischemic attack; NIHSS: National Institutes of Health Stroke Scale; mRS: modified Rankin scale; ASPECTS: Alberta Stroke Program Early CT Score; ECG: electrocardiogram; IV, intravenous.

^a^ Data are presented as percentages or median (interquartiles).

^b^ *n* = 989 in the *DHI* group and *n* = 494 in the placebo group.

^c^ *Xueyu Zheng* Score is obtained from the Efficacy Evaluation Scale of Chinese Medicine Symptom in *Xueyu Zheng*, evaluated on a symptom‐specific weighted visual analogue scale (VAS)‐based scoring system for the 12 items of *Xueyu Zheng* shown in Table S8; the more severe the symptom is, the higher the score be determined.

^d^ EQ‐5D Scale is a standardized instrument for the measurement of health status. Scores range from 0.1702 to 1.00 [41], with higher scores indicating a better quality of life.

### 2.3 Neurological Functional Improvement Following *DHI* Therapy

The proportion of patients with excellent functional outcome (a mRS score of 0–1 at 90 days) was 64.57% (647 out of 1002) in the *DHI* group and 59.24% (298 out of 503) in the control (unadjusted odds ratio [OR]: 1.25, 95% confidence interval [CI]: 1.01–1.56, *p *= 0.04; adjusted OR: 1.43, 95% CI: 1.10–1.86, *p *= 0.007) (Figure 1B). Similar findings were obtained in the per‐protocol analysis (unadjusted OR: 1.35, 95% CI: 1.06–1.73, *p *= 0.02; adjusted OR: 1.61, 95% CI: 1.19–2.19, *p *= 0.002) (Table S6). The *DHI* group showed a better distribution of mRS scores at 90 days than the placebo group (*p *= 0.009) (Figure 1C). Subgroup analysis showed that age <60 years, female gender, baseline NIHSS scores ≤ 6, time from stroke onset to treatment >6 h, previous history of hypertension, absence of diabetes, and combination therapies with statins or CCBs predicted a better functional outcome after stroke in the *DHI* group (Figures 1D and S3), and further considering the interaction effects to the treatment, sex (*p *= 0.036) and the hypertension medical history (*p *= 0.006) still showed a better functional outcome after stroke in the *DHI* group (Table S7).

The percentage of patients with significant improvements on the clinical symptoms of *Xueyu Zheng* [21, 22] (Table S8) was significantly higher in the *DHI* group (63.05%) than in the control (56.99%) (OR: 1.29, 95% CI: 1.03–1.62, *p *= 0.03) at Day 90 (Figure 1B). The number of patients with improved neurological outcomes at 90 days was 763 (83.57%) in the *DHI* group and 368 (79.14%) in the placebo group (OR: 1.34, 95% CI: 1.01–1.78, *p *= 0.04) (Figure 1B). There were no significant between‐group differences in functional independence with BI ≥ 90 at 90 days (OR: 1.04, 95% CI: 0.78–1.38, *p *= 0.78), overall disability at 90 days (OR: 0.99, 95% CI: 0.72–1.36, *p* = 0.93), and time trade‐off values for EQ‐5D (*p *= 0.97) (Figure 1B). Intention‐to‐treat and per‐protocol analyses of secondary outcomes are shown in Figure 1B and Table S6.

### 2.4 Safety of *DHI*

The safety population included all randomized patients who received at least one dose of *DHI* (*n* = 1493). The rate of adverse events at 90 days was not significantly different between the *DHI* group and controls (48.39 vs. 46.09%) (OR: 1.10, 95% CI: 0.88–1.36, *p *= 0.40) (Tables S9 and S10). The rate of serious adverse events was not significantly different between the *DHI* and placebo groups (4.53 vs. 5.21%) (OR: 0.86, 95% CI: 0.53–1.42, *p *= 0.56); The number of deaths in the *DHI* and placebo groups was two (0.20%) and four (0.80%) (OR: 0.25, 95% CI: 0.05–1.37, *p *= 0.09). There were no significant between‐group differences in the incidence of pneumonia, major vascular events, and moderate bleeding. The severe bleeding was not observed in any patient in either group (Table S9). General disorders and administration‐site conditions were more frequent in the *DHI* group (38 [3.82%] vs. 8 [1.60%] patients; OR 2.44, 95% CI: 1.13–5.27, *p *= 0.02), driven mainly by pyrexia (17 [1.71%] vs. 1 [0.20%]); these events were predominantly transient and did not translate into differences in serious adverse events or mortality.

### 2.5 Differentially Expressed Genes Modulated by *DHI*

As presented in Table S11, the clinical characteristics of the 39 patients who underwent RNA sequencing were well‐balanced between the *DHI* and control groups, and exhibited statistical consistent with the demographic characteristics of the overall study population (all *p* > 0.05). *DHI* treatment modulated 23 differentially expressed genes (DEGs) at Day 14 (21 upregulated and 2 downregulated) and 27 DEGs at Day 90 (3 upregulated and 24 downregulated) compared with the control group (Figures 2A and S4 and Table S12).

**FIGURE 2:**
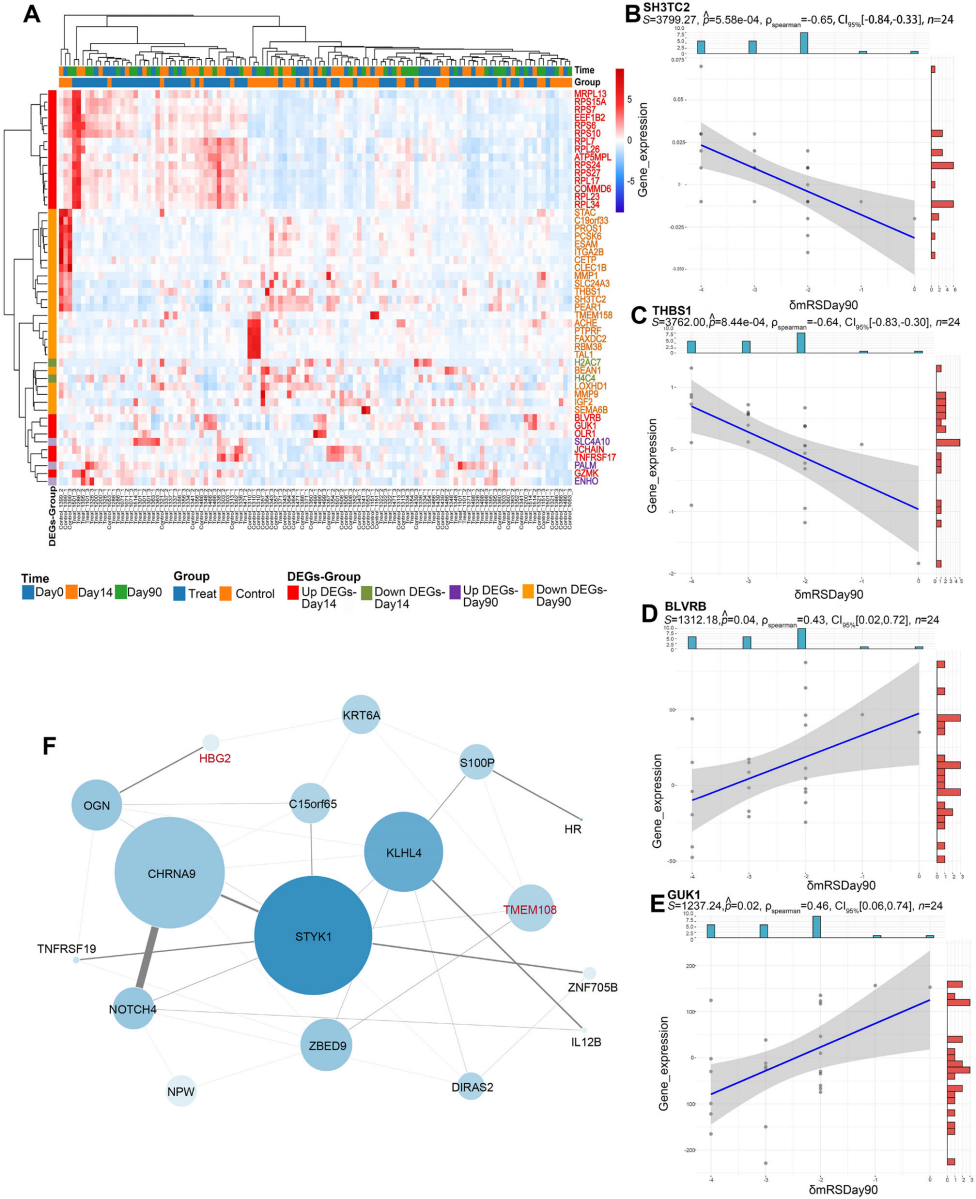
DEGs and target module network regulated by *DHI* in patients with AIS at 90 days. (A) Heatmap of DEGs in the DHI and control groups at 0, 14, and 90 days. The genes upregulated by *DHI* on Days 14 and 90 are shown in red (*N* = 21) and purple (*N* = 3), and the genes downregulated by *DHI* on Days 14 and 90 are shown in green (*N* = 2) and orange (*N* = 24). (B–E) DEGs significantly correlated with δmRSDay90 using Spearman correlation analysis (*p* < 0.05). *SH3TC2* (B) and *THBS1* (C) were negatively associated with clinical outcomes (*r* = −0.65, *p *= 5.58e−4; *r* = −0.64, *p *= 8.44e−4), while *BLVRB* (D) and *GUK1* (E) were positively correlated with outcomes (*r* = 0.43, *p *= 0.04; *r* = 0.46, *p *= 0.02). (F) Targeted module network of *DHI* with 17 nodes (DEMs) and 34 edges (sum of intermodule weight, SW [23]). Each module was named as the hub genes (Figure S6C). The node size indicates the number of genes in each module, and the node color indicates the degree of each module in the TMN. *Abbreviations*: *DHI*: Danhong injection; DEGs: differentially expressed genes; δmRSDay90: mRS at Day 90 minus baseline mRS; DEMs: differentially expressed modules; TMN: target module network.

Functional enrichment analyses showed that *DHI* inhibited thrombo‐inflammatory responses at 14 days, probably by inhibiting neutrophil extracellular trap (NET) formation (hsa04613, *p *= 5.414e−4) or negative regulation of megakaryocyte differentiation (GO:0045653, *p *= 1.914e−3). At 90 days, *DHI* was associated with functions involved in pyramidal neuron development and differentiation (GO:0021860, *p* = 1.754e−3; GO:0021859, *p *= 2.073e−3) and the negative regulation of dopamine receptor signaling pathways (GO:0060160, *p *= 4.787e−4), suggesting potential links to improved neurological recovery (Figure S5A). Furthermore, *DHI* downregulated chronic inflammation‐related pathways, including *IL‐17* signaling (hsa04657, *p *= 9.463e−3), leukocyte transendothelial migration (hsa04670, *p* = 0.014), extracellular matrix–receptor interaction (hsa04512, *p *= 8.332e−3), and the *PI3K–Akt* signaling pathway associated with neuronal apoptosis (hsa04151, *p *= 0.017) (Figure S5B). Collectively, these pathway‐level changes suggest that *DHI* may contribute to a prorecovery molecular profile in AIS.

Moreover, Spearman correlation analysis showed that changes in the expression of four DEGs were significantly correlated with δmRSDay90 (Table S12). Specifically, *SH3TC2* and *THBS1* were negatively associated with clinical outcomes (*r* = −0.65, *p *= 5.58e−4; *r* = −0.64, *p *= 8.44e−4), while *BLVRB* and *GUK1* were positively correlated with outcomes (*r* = 0.43, *p *= 0.04; *r* = 0.46, *p *=  0.02) (Figure 2B–E and Table S12), indicating that changes in DEGs are positively and negatively correlated with clinical outcomes.

### 2.6 Targeted Module Network of *DHI* for AIS Treatment

Using weighted gene coexpression network analysis (WGCNA), 64 modules were identified in the gene coexpression network of the *DHI*‐responsive population (defined as mRS scores ≤ 1 and δmRS scores ≥ −2 at Day 90) (Figure S6A). By comparing module preservation between the *DHI*‐responsive group, placebo group, and *DHI*‐unresponsive group, we further identified 19 *DHI*‐targeted differentially expressed modules (DEMs) comprising 579 genes, all with *Z*‐summary values < 0 (Figure S6B). Each of the 19 DEMs was systematically renamed based on its hub gene, which was determined through integrative analysis of gene significance (GS) for δmRSDay90 and module membership (MM) (Figure S6C). For instance, the *STYK1* gene had the highest GS value (0.41) and MM value (0.84) in its respective module, thus was designated as the hub gene, and this DEM was named the “*STYK1*” module (Figure S6C).

Subsequently, the *DHI*’s targeted module network was constructed, comprising of 17 nodes (DEMs; the “*IL26*” and “*RGS10*” modules were excluded due to no significant intermodule connections) and 34 edges (defined as the sum of intermodule edge weight, SW [23]) (Figure 2F and Table S13). Topological analysis revealed that this network was sparse, with a network density of 0.25, a characteristic path length of 2.059, and a clustering coefficient of 0.242, presenting a modular regulatory structure. In addition, functional enrichment identified 796 biological processes and 52 pathways in these targeted modules, primarily associated with post‐AIS neurological recovery (Table S14). For instance, the “*STYK1*” module, which had the highest node degree (degree = 8) in the targeted module network, was significantly correlated with the maintenance of the blood–brain barrier (GO:0035633, *p *= 3.267e−4). The “*NOTCH4*” module was involved in atherosclerosis and endothelial cell regulation (GO:0001885, *p *= 0.004) [24]; the “*RGS10*” module was associated with blood–brain barrier disruption, neuronal apoptosis, and neuroinflammation through the Wnt signaling pathway (GO:0016055, *p *= 0.005) [25, 26].

### 2.7 Paired Targeted Modules Associated With Neurological Recovery

Correlation analysis between module eigengenes and δmRSDay90 identified two key targeted modules: the “*TMEM108*” module and the “*HBG2*” module, both with absolute correlation coefficients > 0.3 (Figure 3A,B). The “*HBG2*” module, comprising 14 genes, was positively correlated with δmRSDay90 (*r* = 0.353). It was functionally enriched in oxygen transport (GO:0015671, *p *= 3.281e−05) and cellular oxidant detoxification (GO:0098869, *p *= 0.002) (Figure S7A). Topological analysis showed that as treatment response improved (δmRSDay90 increased from −2 to −4), the network structure of the *HBG2* module became increasingly sparser, and its global topological parameter *D* value (integrating normalized network features) was positively correlated with δmRSDay90 (Figure 3A). Conversely, the “*TMEM108*” module, consisting of 37 genes, was negatively correlated with δmRSDay90 (*r* = −0.425). It was functionally related to antithrombotic and anti‐inflammatory effects [27, 28], enriched in the negative regulation of megakaryocyte differentiation (GO:0045653, *p *= 4.257e−4) and NET formation (hsa04613, *p *= 2.394e−4) (Figure S7B). In contrast to the *HBG2* module, the network structure of the *TMEM108* module became progressively denser as treatment response improved, and its *D* value was negatively correlated with δmRSDay90 (Figure 3B).

**FIGURE 3:**
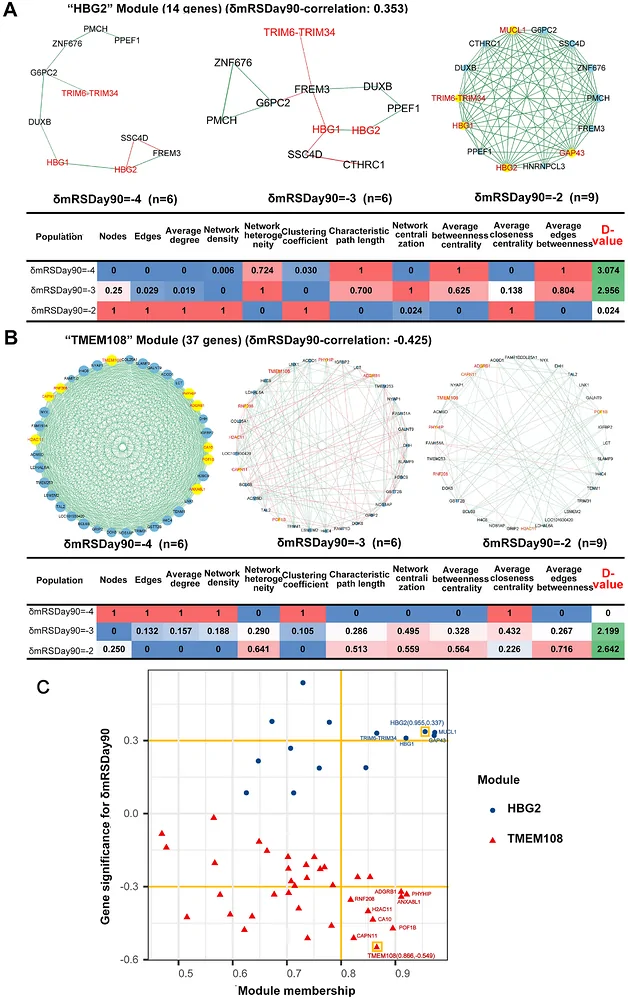
The “*TMEM108*” and “*HBG2*” modules associated with neurological recovery. (A and B) The modules targeted by *DHI* were identified using a correlation with δmRSDay90 of >ǀ0.3ǀ. The topological structure of the modules changed as a function of treatment response. The topological parameters are shown in a table, and D‐values were calculated based on the Euclidean distance. (C) Gene distribution in each module based on the GS for δmRSDay90 and MM. The genes with GS >ǀ0.3ǀ and MM > 0.8 were identified. *Abbreviations*: *DHI*: Danhong injection; δmRSDay90: mRS at Day 90 minus baseline mRS; GS: gene significance; MM: module membership.

Core genes were screened using the criteria of GS > |0.3| and MM > 0.8 [11], resulting in nine core genes in the “*TMEM108*” module and five core genes in the “*HBG2*” module, totaling 14 core genes (Figure 3C). Expression profile analysis of these 14 genes across three time points (Days 0, 14, and 90) in populations with different treatment responses showed consistent expression patterns within each module (Figure 4A). The hub gene of the “*TMEM108*” module, *TMEM108*, previously identified as potential biomarkers for ischemic stroke [29], was significantly decreased in AIS patients compared with the healthy volunteers (*p *= 0.02) (Figure 4B). In *DHI*‐responsive patients, *TMEM108* expression was negatively correlated with δmRSDay90 (*r* = −0.57, *p *= 7.11e−3) (Figure 4D). Furthermore, its expression was significantly higher in patients with an excellent response to treatment (δmRSDay90 = −4) than in those with a poor response (δmRSDay90 = −2) (*p *= 0.04) (Figure 4C).

**FIGURE 4:**
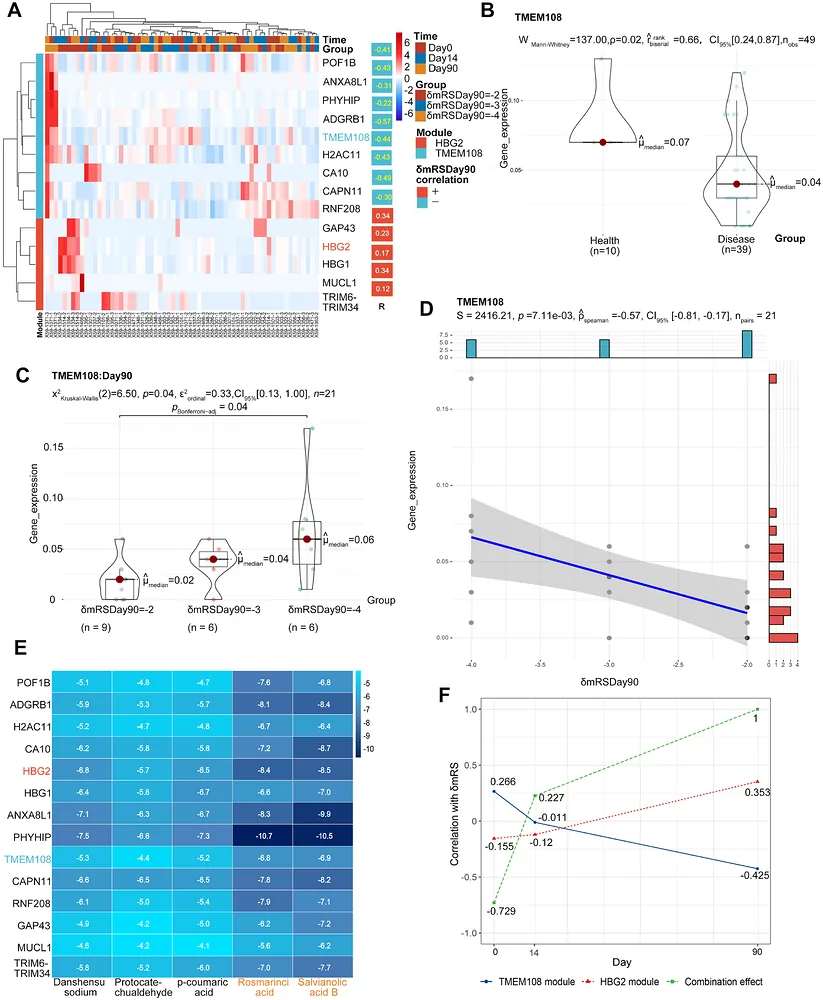
The core genes in the paired targeted modules. Heatmap of 14 core genes (Figure 3C) across different populations at 0, 14, and 90 days. (B) The expression levels of the hub gene “*TMEM108*” between AIS patients and healthy volunteers. (C) The expression levels of the hub gene “*TMEM108*” among the populations with different efficacy using the Kruskal–Wallis test followed by Bonferroni correction (*p*_Bonferroni−adj_ = 0.04). (D) The expression levels of “*TMEM108*” were negatively correlated with δmRSDay90 (*r* = −0.57, *p *= 7.11e−3) using Spearman's correlation analysis. (E) The heatmap of the molecular docking energy between the 5 compounds and the 14 core gene‐encoded proteins. The deeper the color, the higher the binding. (F) The combination effect of the paired targeted modules is based on their correlation with clinical outcomes (mRS at Day 0 and δmRS at Days 14 and 90).

Molecular docking analysis evaluated the binding affinities of the five main active components of *DHI* (*Danshensu sodium*, *protocatechualdehyde*, *p‐coumaric acid*, *rosmarinic acid*, and *salvianolic acid B*) to the proteins encoded by the 14 core genes. The results showed that 82.86% of the compound‐protein pairs had binding energies <−5 kcal/mol, indicating good binding activity; 28.57% of these pairs exhibited high affinity with binding energies ranging from −7.0 to −10.7 kcal/mol (Figure 4E). Among them, *salvianolic acid B* and *rosmarinic acid* displayed the strongest binding affinities, with binding energies ranging from −5.6 to −10.7 kcal/mol (Figure 4E). Structural analysis revealed that *salvianolic acid B* formed stable hydrogen bonds with key residues of *HBG2* (*Asp‐100*, *Phe‐104*, *His‐64*, *Leu‐33*, *Leu‐32*, and *Thr‐28*) and *TMEM108* (*Phe‐15*, *Gln‐11*, and *Cys‐10*). *Rosmarinic acid* had the highest binding affinity (−10.7 kcal/mol) with *PHYHIP*, achieved through hydrogen bonds with residues *Gly‐172*, *Gly‐113*, *Phe‐225*, *Ala‐127*, and *Ala‐223* (Figure S8).

A combination effect model was constructed based on the correlations between the two modules (the “*TMEM108*” and “*HBG2*” modules) and δmRS at different time points. The combination effect score changed from −0.729 at baseline (Day 0) to 0.227 at Day 14 and 1 at Day 90 (Figure 4F), describing the time‐dependent joint association of the two modules with clinical outcome from the disease state at Day 0 into a health state at Day 90. Given their opposite topological changes and complementary correlations with neurological recovery, these two modules were operationally defined as the paired candidate targeted modules of *DHI*.

## 3 Discussion

In our study, for AIS patients with blood stasis syndrome (*Xueyu Zheng*) who miss the time window for intravenous thrombolysis, treatment with the multitarget drug *DHI* for 14 days was associated with a higher likelihood of achieving excellent neurological recovery at 90 days compared with placebo. The randomization system, the inclusion of a placebo group, double‐blinding, and the involvement of three committees should have prevented significant biases in selection, performance, and measurement. This result is the first robust evidence of the efficacy and safety of *DHI* in the management of AIS, consistent with the findings of small clinical studies included in the meta‐analysis [20]. Moreover, our results showed subgroup analyses with interaction effect testing identified significantly greater treatment benefits in females and hypertensive patients. Hypertension is the most significant modifiable risk factor for stroke [30, 31]. In our cohort, 984 patients (65.38%) had hypertension. In this subgroup, the percentage of patients with excellent neurological recovery at 90 days was 11.1% higher in the *DHI* group than in the control group, indicating the potential therapeutic benefit of *DHI* in this high‐risk group of AIS patients. The observed gender difference may be attributed to biological differences in the response to treatment, which warrants further investigation.

In this study, although the sample size for RNA sequencing appears modest (*n* = 30–50 is typical for transcriptomic studies), no significant differences were observed between the RNA subset and the overall trial populations (all *p* > 0.05; Table S11). Within the RNA subset, baseline features were also balanced between *DHI* and placebo group (all *p* > 0.05; Table S11). We divided the populations according to the effect of *DHI* and this intentional selection of molecularly evaluable cases may maximize analytical validity and reduce technical confounding. Thus, the RNA cohort enabled proof‐of‐concept exploration of *DHI*’s multitarget mechanisms. Our study identified 19 targeted DEMs by *DHI*, with functions primarily associated with neurological recovery after AIS; this multitarget characteristic aligns with previous studies [13, 14, 15]. Particularly, regarding the two paired targeted modules significantly related to clinical efficacy, most of the five active compounds in *DHI* can bind to the proteins related to the core genes within these two modules. More importantly, we developed an exploratory combination effect model that quantifies the time‐dependent joint association of paired modules with clinical outcome; although hypothesis generating and not causal, this framework could, after independent validation, be adapted to studies of other multitarget drugs and diseases. The paired targeted modules were identified in patients with a good response to *DHI* treatment.

The paired targeted modules identified in this study reflect two biologically distinct yet complementary dimensions of poststroke recovery. The “HBG2” module, enriched in oxygen transport and cellular oxidant detoxification (Figure S7A), was positively associated with neurological improvement and may represent a recovery‐associated molecular program linked to oxygen handling and redox homeostasis; this interpretation is consistent with evidence that impaired oxygen delivery, mitochondrial dysfunction, and oxidative stress are central to ischemic brain injury, and that strategies improving cerebral perfusion, microvascular stability, and oxygen delivery (e.g., hemoglobin‐based oxygen carriers) can reduce infarct progression and improve functional outcomes in experimental models [32, 33]. In contrast, the “TMEM108” module, enriched in NET formation and negative regulation of megakaryocyte differentiation (Figure S7B), was inversely correlated with neurological recovery; this is compatible with reports that excessive NET formation under oxidative and iron‐dependent stress is associated with microvascular obstruction, endothelial injury, blood–brain barrier disruption, and secondary neuroinflammation after ischemic stroke, and that targeting NET‐associated pathways improves poststroke outcomes in experimental models [34, 35, 36, 37]. Notably, major constituents of *DHI* exhibit anti‐inflammatory, antioxidant, endothelial‐protective, and antithrombotic effects in preclinical studies [15], with *Salvia miltiorrhiza*‐derived compounds reported to regulate NOX‐dependent NET formation [38], which is consistent with the module associations observed in our study. The combination effect score changed from −0.729 at baseline to 0.227 at Day 14 and 1 at Day 90, describing a temporal convergence of the two module‐outcome correlations during the study period, which shows a gradual shift from a disease‐associated to a recovery‐associated transcriptomic pattern; molecular docking further demonstrated favorable binding affinities of *salvianolic acid B* and *rosmarinic acid* with core proteins encoded by these modules, supporting a multicomponent, multitarget regulatory mechanism, though the causal relevance of this paired‐module model requires further experimental validation.

However, several limitations should be acknowledged. First, the paired‐module model was constructed using correlation coefficients between module expression profiles and clinical outcomes at different time points. Although this approach provides a quantitative framework for evaluating the combined contribution of *DHI*‐targeted modules, it does not establish causal relationships between these modules and neurological recovery. Future studies should incorporate intermodule topological changes, dynamic regulatory networks, and external validation cohorts to further refine this model. Second, although subgroup analyses suggested greater clinical benefits in female patients and those with hypertension (Figure 1D), we did not directly link subgroup‐specific clinical phenotypes to the *DHI*‐targeted modules. Further studies are needed to determine whether distinct molecular responses explain the differential efficacy of *DHI* across patient subgroups. Third, our mechanistic interpretations are primarily based on transcriptomic profiling, pathway enrichment, and molecular docking analyses, which provide correlative and predictive evidence but not direct functional validation. We did not directly measure cerebral oxygen metabolism, vascular density, blood–brain barrier permeability, NET formation, platelet–neutrophil interactions, neuronal survival, or angiogenesis. Therefore, the proposed roles of the *HBG2* module in oxygen transport/redox recovery and the *TMEM108* module in thrombo‐inflammatory regulation remain preliminary, which should be confirmed through targeted in vitro and in vivo experiments.

## 4 Conclusion

In conclusion, in patients with AIS and blood stasis syndrome (*Xueyu Zheng*) who missed the time window for intravenous thrombolysis, 14‐day treatment with *DHI* was associated with a higher likelihood of excellent functional outcome at 90 days than placebo, with no significant between‐group differences in any serious adverse event, major vascular events, moderate or severe bleeding, and mortality; the benefit was particularly evident in female patients and patients with a history of hypertension. In exploratory transcriptomic analyses, two paired targeted modules represented by *HBG2* and *TMEM108* were associated with clinical recovery and with biological processes related to oxygen transport/redox homeostasis and thrombo‐inflammatory responses, respectively. The combination effect model offers a hypothesis‐generating framework for studying how multicomponent therapies such as *DHI* may coordinate multiple modules in complex diseases.

## 5 Materials and Methods

### 5.1 Study Design

We conducted an adaptive, randomized, double‐blind, placebo‐controlled trial in 72 participating centers across China, of which 62 centers enrolled patients (10 activated centers withdrew before enrollment and contributed no data; Table S1) from March 2013 through March 2019. The study was approved by the central ethics review committee at Beijing Tiantan Hospital, Capital Medical University (YW2012‐005‐03), and ethical approval, or formal recognition and filing of the central approval by the local institutional review board, was obtained at every participating center before any patient was enrolled at that center. Written informed consent was obtained from all subjects, and participants who underwent RNA sequencing signed an additional informed consent form that included the research purposes of RNA sequencing. The clinical trial steering committee, DMC, and outcome evaluation committee were established for trial monitoring (Table S1). The study protocol has been published [21], with details provided in the *Protocol and Statistical Analysis Plan*, Supporting Information, and the trial was registered at http://clinicaltrials.gov/ (NCT01677208) on August 29, 2012.

### 5.2 Participants

Eligible patients were adults aged between 18 and 70 years, with a diagnosis of AIS at the time of randomization and a baseline NIHSS score ≥4 and <20. Patients were diagnosed with *Xueyu Zheng*, representing systemic microcirculatory dysfunction in patients with AIS. Diagnosis relied on the *Xueyu Zheng* Chinese Medicine Symptom Diagnostic Scale containing 12 items: hemiplegia, limb numbness, dim dark complexion, dark‐purple lips, rough skin, fixed focal pain, dark/purple tongue, tongue ecchymosis, purple sublingual vessels, sublingual varices, and uneven/intermittent pulse. Each item was scored as present (full weighted maximum score) or absent (0), with total scores ranging 0–93; a score ≥20 defined Xueyu Zheng [21, 22]. The paired *Xueyu Zheng* Chinese Medicine Symptom Efficacy Evaluation Scale (Table S8) shares the same 12 items and maximum total score of 93 as the diagnostic scale but uses different scoring: whereas the diagnostic scale applies binary present/absent scoring, the efficacy scale assesses symptom severity via item‐specific weighted visual analogue scales ranging from 0 to each item's maximum. This efficacy scale was applied for baseline severity characterization (Table 1) and treatment response assessment (see Outcomes). Patients were enrolled up to 1 week after the stroke onset and who with a history of prior stroke should have no significant prestroke disability. All patients missed the time window for thrombolysis. Computed tomography and magnetic resonance imaging were performed to identify patients with an anterior circulation occlusion. Patients who underwent endovascular treatment, or with a documented history of atrial fibrillation or atrial fibrillation on the qualifying screening ECG, severe cardiac, renal or hepatic dysfunction were excluded. Any other Chinese medicine with a similar pharmacological profile of *DHI* was not allowed throughout the study period. Interpretation of imaging was performed using the ASPECTS [39] and clinical data were verified by independent monitors. A full list of inclusion and exclusion criteria are available in Table S4.

### 5.3 Interventions

Routine medical care was provided to all patients according to the China Guideline for the Diagnosis and Treatment of Acute Ischemic Stroke [40]. Study sites adhered to National Guidelines for stroke unit care, stroke rehabilitation, and stroke prevention care. All treatments were recorded on electronic case report forms. Participants were randomly assigned to receive a daily intravenous injection of 40 mL *DHI* or placebo (40 mL of 0.9% saline), added to 250 mL of 0.9% saline, for 14 days. The follow‐up lasted to 90 days after randomization. All patients underwent standard assessments, including demographic and clinical characteristics, and laboratory tests.

### 5.4 Outcomes

The primary outcome was excellent functional outcome, defined as a mRS score of 0–1 at 90 days. Secondary outcomes involved: (a) the proportion of patients who had significant improvements measured through the efficacy evaluation scale of Chinese Medicine Symptom in “*Xueyu Zheng*” of ischemic stroke [21, 22] (Table S8); (b) neurological outcome, defined as a NIHSS score of 0–1 or an improvement ≥ 4 points from baseline; (c) functional independence in activities of daily living through the BI score ≥ 90; (d) global disability, defined as a mRS score ≥ 3; (e) health‐related quality of life measured by the EQ‐5D [41]. All the investigators were intensively trained with the assessment scales before the study. Safety outcomes included the incidence of adverse events, major vascular events, transient ischemic attack, myocardial infarction, and vascular‐related death, moderate and severe bleeding according to the Global Utilization of Streptokinase and TPA for Occluded Arteries (GUSTO) criteria [42] and mortality at 90 days.

### 5.5 Randomization and Masking

Eligible participants were randomly assigned to receive 14‐day intravenous *DHI* or placebo (saline) in a 2:1 ratio via a central randomization system (Brightech, Somerset, USA). The system generated a randomization number and randomized patients in randomly generated blocks of 6. Patients, investigators, outcome assessors, and statisticians were blinded to treatment allocation. Since the *DHI* and placebo did not appear identical, infusion bottles were wrapped in sealed and shaded bags, and then administered through shading infusion devices. The procedures were implemented by two professional nurses who signed a confidentiality agreement before study initiation. One nurse was in charge of preparing the drugs and sealing the infusion bottles with shaded bags. The second nurse supervised the infusion process and ensured a correct allocation of the study drugs with blinded to the patients.

### 5.6 Interim Analyses and Re‐Estimation of Sample Size

Based on the previous study, we assumed to achieve a 45% proportion in controls [43], with a 10% difference compared with treated patients, at 5% significance with 80% power. Considering a drop‐out rate of 20%, we estimated that 846 patients should be enrolled in the study. We planned interim analyses at *n* = 282 and *n* = 564 patients using the O'Brien‐Fleming boundary approach (Table S2). The interim analyses were conducted with comparative analyses by an independent statistician from DMC. During interim analysis, the DMC bases its recommendations on the CP value: trial discontinuation for futility if CP < 0.3, early termination for efficacy if CP > 0.8, or sample size reassessment if 0.3 ≤ CP ≤ 0.8 to maintain statistical power.

### 5.7 Statistical Analysis

The primary analysis was done on the intention‐to‐treat and per‐protocol population. The safety population included all patients who received the study treatment. Missing data of the primary outcome were handled using the baseline‐observation‐carried‐forward approach, while missing data of secondary outcomes were not imputed. We reported the primary outcome with adjusted and unadjusted ORs and 95% CIs from multiple logistic regression models. Subgroup analyses were performed on prespecified covariates (age, sex, BMI, baseline NIHSS/ASPECTS, onset‐to‐treatment time) and posthoc variables (medical history, ischemic location, concomitant medications), followed by formal interaction testing between treatment groups and these subgroup characteristics using logistic regression with multiplicative interaction terms. Other analyses included an unadjusted comparison of the distribution of mRS scores at baseline and after 90 days using Wilcoxon rank‐sum test, and binary comparisons of other secondary outcomes using chi‐square or Fisher test, reported as unadjusted ORs with 95% CIs. EQ‐5D scores, standardized with time trade‐off values based on a representative sample of Chinese population [41], were compared using *t*‐test. All *p* values are two‐sided. All analyses were performed with the SAS software, version 9.4 (SAS Institute, Cary, NC).

### 5.8 Discovery of DEGs in RNA‐Seq Analysis

Serum samples from 39 patients in three sites signed informed consent on the RNA‐Seq analysis (24 in the *DHI* group and 15 in the control group) were obtained for RNA sequencing using the Illumina HiSeq 4000 sequencing platform. We divided these patients into three groups for further analysis: 21 cases with good response to *DHI* (defined as mRS scores 0–1 and δmRSDay90 ≤ −2 at Day 90, while δmRSDay90 was defined as mRS at Day 90 minus baseline mRS), three unresponsive cases, and 15 placebo cases. Significant DEGs were defined as having a *p* value < 0.05 and at least a 1.5‐fold change using DESeq2 [44]. Spearman correlation analysis were used to detect the DEGs correlated with clinical outcomes (δmRSDay90). The enriched Gene Ontology (GOs) and Kyoto Encyclopedia of Genes and Genomes (KEGG) pathways of the DEGs were evaluated by using R software (v.4.2.2) package clusterProfiler (v.4.5.0) [45].

### 5.9 Construction of the Targeted Modular Network by *DHI*

The construction of the coexpressed gene network at Day 90 of the group with a good response to *DHI*, and the identification of the corresponding modules were implemented using WGCNA, an R package for weighted correlation network analysis [46]. Coexpression modules were detected through hierarchical clustering using topological overlap measure with average linkage, followed by dynamic hybrid tree‐cut algorithm to define dendrogram branches as modules (minimum size = 3) [47]. Each module received a unique color identifier. To evaluate *DHI*‐responsive module specificity, we computed *Z*_summary_ statistics via WGCNA's preservation test, designating modules with *Z*_summary_ < 0 versus controls as DEMs potentially drug‐activated [48]. The equation defining *Z*_summary_ is as follows [49]:

(1) $$Z_{\mathrm{summary}}=\frac{\mathrm{median}\left(Z_{\mathrm{meanCor}},Z_{\mathrm{mean}\mathrm{Adj}},Z_{\mathrm{prop}\mathrm{VarE}\mathrm{xpl}},Z_{\mathrm{mean}\mathrm{KME}}\right)+\mathrm{median}\left(Z_{\mathrm{cor}.\mathrm{KIM}},Z_{\mathrm{cor}.\mathrm{KME}},Z_{\mathrm{cor}.\mathrm{cor}}\right)}{2}$$

We reconstructed a DEM‐based network using modules as nodes and summed edge weights (SW) between module pairs as edges [23]. For any connected modules *M_x_* and *M_y_*, SW was calculated as:

(2) $$\mathrm{SW}\left(M_{x},M_{y}\right)=\sum_{i\in M_{x},j\in M_{y}}a_{ij}$$

where i∈M_x_, j∈M_y_, and *a_ij_* represents edge weight. This quantified all direct intermodule connections.

Network visualization was used Cytoscape 3.9.1 [50], with DEMs renamed by their hub genes. Hub genes were selected as: (1) highest GS when GS(δmRSDay90) > 0.3 and MM > 0.8, or (2) highest MM when no genes met MM > 0.8 threshold.

### 5.10 Determining the Paired Targeted Modules of *DHI*

Significant targeted DEMs were detected as the Pearson correlation coefficients (*r*) over 0.3 between each DEM and δmRSDay90. The paired targeted modules from significant targeted DEMs were further determined according to their global topological *D* value in the good responsive population with different effects (δmRSDay90 = −4, −3, or −2) based on Euclidean distance. If *a* = (*a*_1_, *a*_2_, …, *a_n_*) and *b* = (*b*_1_, *b*_2_, …, *b_n_*) are two modules in Euclidean *n*‐space, then the *D* score is the distance (*d*) from *a* to *b* and is defined as follows [11]:

(3) $$d=\sqrt{{\left(a_{1}-b_{1}\right)}^{2}+\cdot \cdot \cdot \cdot \cdot \cdot +{\left(a_{n}-b_{n}\right)}^{2}}\;$$

We employed the Kruskal–Wallis test, adjusted with Bonferroni correction, to compare the expression levels of hub genes in the paired targeted modules between healthy volunteers and AIS patients, as well as across different efficacy groups at Day 90. Spearman's correlation analysis was further utilized to assess the relationship between hub genes and clinical efficacy. In order to assess binding affinities between the active compounds of *DHI* and the proteins encoded by the core genes, molecular docking between module core genes and the five active compounds was performed by: (1) retrieving compound structures from PubChem (https://pubchem.ncbi.nlm.nih.gov/) and converting their SDF files into PDB files using Open Babel 2.3.2 software [51]; (2) obtaining predicted protein structures from AlphaFold (https://alphafold.ebi.ac.uk/) [52] with subsequent structural refinement; (3) establishing flexible binding pockets through grid parameter optimization; (4) executing systematic docking simulations using AutoDock Vina 1.1.2 [53] to calculate binding affinities, with scores ≤−4.25 kcal/mol indicating potential binding activity; (5) visualizing protein–ligand interactions in three dimensions using PyMOL.

### 5.11 The Combination Effect Model

We constructed a combination effect model as an exploratory, hypothesis‐generating tool to quantify the time‐dependent joint association of the paired modules with clinical outcome during *DHI* treatment. First, we calculated the correlation coefficients between gene expression levels of the paired modules and clinical outcomes (mRS at Day 0 and δmRS at Days 14 and 90) at each time point. The time‐dependent correlation coefficients of the *HBG2* and *TMEM108* modules with δmRS were defined as *N*_(_ *_t_*_)_ and *P*_(_ *_t_*_)_, respectively. The combined effect was derived as the weighted sum of *P*_(_ *_t_*_)_ and *N*_(_ *_t_*_)_, with Day 0 set as the baseline. The model is formulated as:

(4) $$f\left(t\right)=-\frac{\omega_{1}N_{t}+\omega_{2}P_{t}}{\sqrt{\omega_{1}^{2}N_{0}^{2}+\omega_{2}^{2}P_{0}^{2}}\;}$$

In this model, *N*_(_ *_t_*_)_ and *P*_(_ *_t_*_)_ denote, respectively, the time‐dependent correlation coefficients between the *HBG2* module (positively correlated with δmRS) or the *TMEM108* module (negatively correlated with δmRS) and the outcome at time *t*; *N*_0_ and *P*_0_ are the corresponding baseline (Day 0) correlations. *ω*_1_ and *ω*_2_ are non‐negative normalized weights of the paired modules, constrained by *ω*_1_ + *ω*_2_ = 1, and solved using the boundary condition *f*(90) = 1.

Additional details regarding the study methods are included in *Materials and Methods*, Supporting Information. Details regarding the trial design and statistical analysis are provided in *Protocol and Statistical Analysis Plan*, Supporting Information.

## Author Contributions

*Conceptualization*: Zhong Wang, Xingquan Zhao, and Yongyan Wang. *Data curation*: Bingwei Chen, Wei Liu, Shuang Guan, and Yinli Shi. *Formal analysis*: Bingwei Chen, Jun Liu, Shuang Guan, Yinli Shi, Bing Li, Xiaolong Zhu, Fei Teng, and Xuanzhu Liu. *Funding acquisition*: Zhong Wang, Yongyan Wang, and Jun Liu. *Investigation*: Yilong Wang, Jingjing Lu, and Xingquan Zhao. *Methodology*: Yilong Wang, Jun Liu, Bingwei Chen, Zhong Wang, Niwen Zhou, and Lixing Zhu. *Supervision*: Chunti Shen, Qiguang Chen, Bing Li, Yanan Yu, and Haixia Dang. *Validation*: Jun Liu and Yinli Shi. *Visualization*: Jun Liu, Shuang Guan, and Yinli Shi. *Writing – original draft*: Jun Liu, Bingwei Chen, Lin Chen, and Zhong Wang. *Writing – review and editing*: all the authors, led by Dr. Zhong Wang. All authors reviewed, edited, and approved the final version. The corresponding authors attests that all listed authors meet authorship criteria and that no others meeting the criteria were omitted.

## Fundings

This work was funded by Scientific and Technological Innovation Project of China Academy of Chinese Medical Sciences (CI2023C063YLL), National Natural Science Foundation of China (81673833), and China National Science and Technology Major Project for “Significant New Drugs Development” (2011ZX09304‐07).

## Ethics Statement

This study was approved by the central ethics review committee at Beijing Tiantan Hospital, Capital Medical University (YW2012‐005‐03), and ethical approval (or local institutional review board recognition and filing of the central approval) was obtained from every participating center before enrollment began at that center. Written informed consent for publication was obtained from all patients. Participants who underwent RNA sequencing signed an additional informed consent form that included the research purposes of RNA sequencing. Data have been deidentified for publication. Studies were performed in accordance with the Declaration of Helsinki. The trial was registered in http://clinicaltrials.gov/ (No. NCT01677208).

## Conflicts of Interest

The authors declare no conflicts of interest.

## Supporting information




**Supporting File 1**: mco271135‐sup‐0001‐SuppMat.docx.

**Supporting File 2**: mco271135‐sup‐0002‐Fileprotocol.docx

## Data Availability

Individual deidentified participant data (including data dictionary), statistical code, and any other materials should be submitted to the corresponding author Zhong Wang (zhonw@vip.sina.com) with an approval of a proposal and subject to a signed data sharing agreement. The RNA sequencing data that support the findings of this study have been deposited into CNGB Sequence Archive (CNSA) of China National GeneBank DataBase (CNGBdb) with accession number CNP0002663 (https://db.cngb.org/search/project/CNP0002663/).
