# Supplementary material for: Effects of Danhong Injection on 90‐Day Functional Outcome in Acute Ischemic Stroke: An Adaptive, Multicenter, Randomized, Double‐Blind, Placebo‐Controlled Phase IV Trial
Source: MedComm (2020). 2026 Nov 8;7(11):e71135. doi: 10.1002/mco2.71135 (PMC13744646; doi:10.1002/mco2.71135)
Supplement: Supplementary file 1 [file MCO2-7-e71135-s001.docx]

Supplementary Materials for

Effects of Danhong injection on 90-day functional outcome in acute ischemic stroke: an adaptive, multicenter, randomized, double-blind, placebo-controlled phase IV trial

Jun Liu^1#^, Yilong Wang^2,3#^, Bingwei Chen^4^, Jingjing Lu^2,3^, Lin Chen^1,5^, Shuang Guan^1^, Yinli Shi^1^, Wei Liu^6^, Yanan Yu^1^, Bing Li^7^, Haixia Dang^8^, Xiaolong Zhu^9^, Fei Teng^9^, Xuanzhu Liu^9^, Niwen Zhou^10^, Qiguang Chen^4^, Chunti Shen^11^, Lixing Zhu^10^, Yongyan Wang^1*^, Xingquan Zhao^2,3*^, Zhong Wang^1*^; Danhong-Stroke Trial Investigators^†^.

Correspondence to: Zhong Wang (zhonw@vip.sina.com) or Xingquan Zhao(zxq@vip.163.com) or Yong-Yan Wang (wangyongyan2010@sina.cn)

**This file includes:**

Materials and Methods

Figure S1 The standard of the fingerprint electropherogram of *DHI*.

Figure S2 The chemical structure of the 5 main component content in *DHI*.

Figure S3 Effect of *DHI* treatment by prespecified subgroups of combination therapy

Figure S4 The number of DEGs modulated by *DHI* at 14 days and 90 days

Figure S5 Gene Ontology (GO) and Kyoto Encyclopedia of Genes and Genomes (KEGG) pathway enrichment analysis of DEGs.

Figure S6 The differentially expressed modules (DEMs) targeted by *DHI*.

Figure S7 Bar graph of enriched GO terms and KEGG pathways of “HBG2” and “TMEM108”

Figure S8 Molecular docking binding energies of *Rosmarinic Acid* and *Salvianolic Acid B* with the gene-encoded proteins in the paired targeted modules.

Table S1 Full list of “*Danhong*-Stroke Trial Investigators”

Table S2 The α spending using an O’BrienFleming boundary in the two inter analyses

Table S3 The treatment effect on the primary outcome (modified Rankin Scale 0-1) in the two interim analyses in intention-to-treat population and the Per Protocol Population

Table S4 Trial Inclusion and Exclusion Criteria

Table S5 The concomitant medications between two groups

Table S6 Treatment Effect in the Per Protocol Population (n=1273)

Table S7 Interaction effects between characteristics of subgroups and treatments

Table S8 The efficacy evaluation scale of Chinese Medicine Symptom in “*Xueyu Zheng*” of ischemic stroke

Table S9 The safety outcomes between two groups in the safety population (n = 1493)

Table S10 The significant adverse events by MedDRA preferred term

Table S11 Clinical characteristics of patients who provided serum samples for RNA sequencing

Table S12 The differentially expressed genes before and after treatment with *DHI* compared with control group

Table S13 The targeted modular network of 19 DEMs treated with *DHI*

Table S14 The enriched GOs and KEGG pathways of the 19 DEMs treated with *DHI*

**Other Supplementary Materials for this manuscript include the following:**

**Supplementary File:** Protocol&SAP

**Materials and Methods**

**Human subjects in the trial**

***Study design***

This was a multicenter, randomized, double-blind, placebo-controlled, parallel-group, adaptive-design study aimed to assess the efficacy and safety of intravenous DHI in patients with acute ischemic stroke who missed the time window of thrombolysis. The trial was conducted in 72 participating centers across China, of which 62 centers enrolled patients (10 activated centers withdrew before enrollment and contributed no data; Table S1). The study was approved by the central ethics review committee at Beijing Tiantan Hospital, Capital Medical University (YW2012-005-03), and ethical approval, or formal recognition and filing of the central approval by the local institutional review board, was obtained at every participating center before any patient was enrolled at that center. Written informed consent was obtained from all subjects, and participants who underwent RNA sequencing signed an additional informed consent form that included the research purposes of RNA sequencing. The study protocol has been reported [1] and registered in http://clinicaltrials.gov/, Registration Number (NCT01677208). The clinical trial steering committee, the data monitoring committee (DMC), and the outcome evaluation committee were established for clinical trial monitoring (Table S1).

***Inclusion and exclusion criteria of the participants***

Eligible patients were adults aged between 18 and 70 years, with a diagnosis of acute ischemic stroke at the time of randomisation and a baseline National Institutes of Health Stroke Scale (NIHSS) score ≥ 4 and < 20. Patients were diagnosed with “*Xueyu Zheng*” (blood stasis syndrome), which reflects systemic microcirculatory dysfunction in AIS patients, defined as a total score of the Chinese Medicine Symptom Diagnostic Scale of “*Xueyu Zheng*” ≥ 20, which assesses the same 12 items (hemiplegia, numbness of limbs, dimmish and darkish complexion, dark-purple lips, rough skin, fixed localized pain, purple or darkish tongue, ecchymosis on the tongue, purple sublingual vessels, sublingual varices, and an unsmooth or intermittent pulse) scored as present (assigned the item’s full weighted score, i.e., its maximum) or absent (0), with a total score range of 0–93 [1-3]. The Efficacy Evaluation Scale of Chinese Medicine Symptom in “*Xueyu Zheng*” (Table S8) share the same 12 evaluation items and a maximum total score of 93 but use different scoring methods: the diagnostic scale scores presence or absence (as described above), whereas the efficacy scale grades the severity of each present item on a symptom-specific weighted Visual Analogue Scale, from 0 up to that item’s maximum, and is used to describe baseline severity and to assess treatment response. Patients were enrolled up to one week after the stroke onset and who with a history of prior stroke should have no significant pre-stroke disability. All patients missed the time window for thrombolysis. Computed tomography (CT) and magnetic resonance imaging (MRI) were performed to identify patients with an anterior circulation occlusion. Patients who underwent endovascular treatment, or with a documented history of atrial fibrillation or atrial fibrillation on the qualifying screening ECG, severe cardiac, renal or hepatic dysfunction were excluded. Any other Chinese medicine with a similar pharmacological profile of *DHI* was not allowed throughout the study period. Interpretation of imaging was performed using the Alberta Stroke Program Early CT Score (ASPECTS) [4] and clinical data were verified by independent monitors. The details with the inclusion and exclusion criteria of the participants was shown in Table S4.

**Cohort details with RNA sequencing**

Serum samples for RNA sequencing were obtained from 39 patients (24 in the DHI group and 15 in the control group). We divided these patients into 3 groups for further analysis: 21 cases with good response to DHI (defined as mRS scores 0-1 and δmRSDay90 ≤ -2, while δmRSDay90 was defined as mRS at Day 90 minus baseline mRS; 6 for δmRSDay90=-4, 6 for δmRSDay90=-3 and 9 for δmRSDay90=-2), 3 unresponsive cases and 15 placebo cases.

**Trial Details**

***Trial procedures***

Routine medical care was provided to all patients according to the China Guideline for the Diagnosis and Treatment of Acute Ischemic Stroke [5]. Study sites adhered to National Guidelines for stroke unit care, stroke rehabilitation, and stroke prevention care. All treatments were recorded on electronic case report forms (eCRFs). Participants were randomly assigned to receive a daily intravenous injection of 40 ml *DHI* or placebo (40 ml of 0.9% saline), added to 250 ml of 0.9% saline, for 14 days. The follow-up lasted to 90 days after randomisation. All patients underwent standard assessments, including demographic and clinical characteristics, and laboratory tests.

***Randomization and masking***

Eligible participants were randomly assigned to receive 14-day intravenous *DHI* or placebo (saline) in a 2:1 ratio via a central randomization system (Brightech, Somerset, USA). The system generated a randomization number and randomized patients in randomly generated blocks of 6. Patients, investigators, outcome assessors, and statisticians were blinded to treatment allocation. Since the *DHI* and placebo did not appear identical, infusion bottles were wrapped in sealed and shaded bags, and then administered through shading infusion devices. The procedures were implemented by two professional nurses who signed a confidentiality agreement before study initiation. One nurse was in charge of preparing the drugs and sealing the infusion bottles with shaded bags. The second nurse supervised the infusion process and ensured a correct allocation of the study drugs with blinded to the patients.

***Trial outcomes***

The primary outcome was excellent functional outcome, defined as a mRS score of 0-1 at 90 days.

Secondary outcomes involved: a) the proportion of patients who had significant improvements measured through the efficacy evaluation scale of Chinese Medicine Symptom in “*Xueyu Zheng*” of ischemic stroke [1-3] (Table S8). *Xueyu Zheng* reflects systemic microcirculatory dysfunction in AIS patients and is characterized by 12 typical clinical manifestations such as hemiplegia, numbness of limbs, dimmish and darkish complexion, dark-purple lips, rough skin, fixed localized pain, purple or darkish tongue, ecchymosis on the tongue, purple sublingual vessels, sublingual varices, and an unsmooth or intermittent pulse. These features were assessed using the validated Ischemic Stroke TCM Syndrome Diagnostic Scale (ISDS) (Table S8), developed by the China Association of Chinese Medicine [2-3]. b) neurological outcome, defined as a NIHSS score of 0-1 or an improvement ≥ 4 points from baseline; c) functional independence in activities of daily living (ADL) through the Barthel Index score ≥ 90; d) global disability, defined as a mRS score ≥ 3; e) health-related quality of life measured by the EuroQol Group 5-Dimension Self-Report Questionnaire (EQ-5D).[6] All the investigators were intensively trained with the assessment scales before the study. Safety outcomes included the incidence of adverse events, major vascular events including stroke (ischemic or hemorrhagic) transient ischemic attack (TIA), myocardial infarction, and vascular-related death, moderate and severe bleeding according to the GUSTO bleeding criteria [7] (severe bleeding defined as fatal bleeding, intracranial hemorrhage, or bleeding requiring transfusion, infusion, vasoconstrictor drugs, or surgical intervention; moderate bleeding defined as bleeding requiring transfusion but not meeting severe-bleeding criteria) and mortality at 90 days.

***Interim analyses and reestimation of Sample size***

Calculation of sample size was based on the difference between the proportion of patients with an mRS of 0-1 at 90 days after randomization in the treatment and control groups, at 5% significance with 80% power. We assumed to achieve a 45% proportion in controls [8], with a 10% difference compared to treated patients. Considering a drop-out rate of 20%, we estimated that 846 patients should be enrolled in the study. We planned interim analyses at n = 282 and n = 564 patients using the O’Brien-Fleming boundary approach (Table S2). The interim analyses were conducted with comparative analyses by an independent statistician from DMC. During the interim analysis, the Conditional Power (CP) value was calculated. If the CP value falls below 0.3, the DMC would recommend discontinuing the trial due to futility. If the CP value exceeds 0.8, the trial may be terminated early for efficacy. For CP values ranging between 0.3 and 0.8, a reassessment of the sample size is required to ensure adequate statistical power for further evaluation.

***Statistical analysis***

The primary analysis was done on the intention-to-treat and per-protocol population. Missing data of the primary outcome were handled using the baseline-observation-carried-forward approach, while missing data of secondary outcomes were not imputed. The estimate of primary outcome was adjusted for covariates (age, baseline mRS, baseline NIHSS score, baseline ASPECTS [4], time from stroke onset to randomization, history of previous stroke or TIA, hypertension, hyperlipidemia, diabetes mellitus, ischemic heart disease and ECG showing atrial fibrillation). We reported adjusted and unadjusted odds ratios (ORs) and 95% confidence intervals (CIs) from multiple logistic regression models. An exploratory analysis of heterogeneity was conducted to evaluate potential effect modifications across prespecified covariates, including demographic characteristics (age, sex), anthropometric measures (BMI), clinical severity indicators (baseline NIHSS score, ASPECTS [4]), stroke onset-to-treatment time, as well as post-hoc variables including medical history profiles (history of previous stroke or TIA, hypertension, hyperlipidemia, diabetes mellitus, and ischemic heart disease), neuroanatomical localization of ischemia, and concomitant administration of aspirin, clopidogrel, anticoagulants, neuroprotective agents, statins or calcium channel blockers (CCB). Significant treatment interaction effects were subsequently examined through multivariable logistic regression models incorporating multiplicative interaction terms between treatment group assignment and these subgroup characteristics. Other analyses included an unadjusted comparison of the distribution of mRS scores at baseline and after 90 days using Wilcoxon rank-sum test, and binary comparisons of improvements based on *Xueyu Zheng* scores [1-3], NIHSS scores, Barthel Index, global disability rates, incidence of adverse events, serious adverse events, deaths, major vascular events and moderate or severe bleeding at 90 days using chi-square or Fisher test. EQ-5D scores, standardized with time trade-off values based on a representative sample of Chinese population [6] were compared using t-test. The safety population included all patients who received the study treatment. Adverse events were reported using standardized terminology (Medical Dictionary for Regulatory activities v.22.0). Binary outcomes were analyzed with logistic regression and are reported as unadjusted ORs with 95% CIs. All *P* values are two-sided. All analyses were performed with the SAS software, version 9.4 (SAS Institute, Cary, NC).

**RNA Sequencing Data Analysis**

***RNA sequencing flow***

The serum samples of 39 patients (117 samples) were sequenced using the Illumina HiSeq 4000 sequencing platform. We used internal software SOAPnuke(v1.5.2) to filter reads, followed as: 1) Remove reads with adaptors; 2) Remove reads in which unknown bases(N) were more than 10%; 3) Remove low quality reads (we defined the low quality read as the percentage of base which quality was lesser than 20 is greater than 40% in a read). After filtering, the remaining reads were called "Clean Reads" and stored in FASTQ format [9]. After reads filtering, we mapped clean reads to reference genome using HISAT2 [10]. We mapped clean reads to reference transcripts using Bowtie2 [11], then calculated gene expression level for each sample with RSEM [12]. The average mapping ratio with reference genome is 92.37%, the average mapping ratio with gene is 89.69%; A total of 18716 mRNAs were detected.

***Discovery of differentially expressed genes (DEGs)***

The DEG results were obtained using DESeq2 [13]. Student’s t-test was used to detect significantly differences in expression profiles before and after drug administration, compared with the control group. Significant DEGs were defined as having a p-value <0.05 and at least a 1.5-fold change (FC). Spearman correlation analysis were used to detect the DEGs correlated with clinical outcomes (δmRSDay90).

***Function enrichment analysis***

The enriched Gene Ontology (GOs) and Kyoto Encyclopedia of Genes and Genomes (KEGG) pathways of the DEGs were evaluated by using R software (v.4.2.2) package clusterProfiler (v.4.5.0) [14] through Hiplot Pro (https://hiplot.com.cn/), a comprehensive web service for biomedical data analysis and visualization.

**Coexpressed Gene Network Analysis**

***Construction of the targeted modular network by DHI***

The construction of the coexpressed gene network at Day 90 of the group with a good response to *DHI*, defined as mRS scores 0-1 and δmRSDay90 ≤ -2 at Day 90, and the identification of the corresponding modules were implemented using WGCNA, an R package for weighted correlation network analysis [15]. To identify coexpressed modules, we used a topological overlap measure to perform average linkage hierarchical clustering to obtain a dendrogram whose branches were identified using the dynamic hybrid tree cut algorithm [16]. Then, the branches were defined as modules, and each module was assigned a color. We set the minimum module size to 3.

To quantitatively assess whether modules in the group with a good response to *DHI* were changed in coexpression patterns independent of the placebo and the unresponsive groups, we adopted a Z_summary_ statistic implemented in the module preservation function of WGCNA[15]. We defined a module with a Z_summary_ < 0 compared to the control groups as a targeted differentially expressed module (DEM), which might be activated by a drug [17]. The equation defining Z_summary_ is as follows[18]:

$\text{Z}_{\text{summary}}\text{=}\frac{\text{median(Z}_{\text{meanCor}}\text{,Z}_{\text{meanAdj}}\text{,Z}_{\text{propVarExpl}}\text{,Z}_{\text{meanKME}}\text{)+median(Z}_{\text{cor.KIM}}\text{,Z}_{\text{cor.KME}}\text{,Z}_{\text{cor.cor}}\text{)}}{2}$ (1)

Furthermore, we reconstructed the targeted modular network (TMN) with the DEMs as nodes and the interactive relationships between modules defined by the the sum of weight of edges between pairs of modules (SW) [19], as the edges:

$SW(M_{x},M_{y})=\sum_{i\in Mx,j\in My} a$*_ij_*	(2)

where M_x_ and M_y_ denote any two modules connected by at least one edge, *i* and *j* are a gene in M_x_ and M_y_, respectively, and a*_ij_* is the weight of edge between gene i and j. Using this formula, we calculated the direct inter-module connections for any module pair possessing one or more edges.

This network was visualized using Cytoscape 3.9.1[20]. Afterwards, we renamed each targeted DEM using its hub gene. In modules with gene significance (GS) for δmRSDay90 > 0.3 and module membership (MM) >0.8 calculated by WGCNA, the hub gene was defined as the one with the highest GS. In modules without genes with MM>0.8, the hub gene was defined as the one with the highest MM.

***Determining the paired targeted modules correlated with the efficacy of DHI***

Furthermore, to detect the targeted DEMs associated with the clinical efficacy of *DHI*, we calculated the pearson correlation coefficients (r) between each DEM and the primary clinical outcome (δmRSDay90) by WGCNA[15]. Significant targeted DEMs were defined as those with r>0.3. Then, we utilized genes from the DEMs associated with the clinical efficacy of *DHI* to reconstruct the corresponding gene coexpression modules in the good responsive population with different effects (δmRSDay90=-4, δmRSDay90=-3 and δmRSDay90=-2). This was achieved by calculating the Pearson coexpression correlation of pairwise genes, selecting edges with a correlation coefficient exceeding 0.5, and employing Cytoscape 3.9.1 for visualization [20]. A topological analysis of these modules was performed with the network analysis functionality of Cytoscape 3.9.1[20]. In addition, we obtained a global topological parameter D score by integrating each normalized topology parameter in these modules in the good responsive populations with different effects treated with *DHI*. The D value model was based on Euclidean distance, which calculates the distance (similarity) between two points, especially for measurements of similarity between multidimensional data (Euclidean n-space) [21]. If a= (a_1_, a_2_, ..., a_n_) and b = (b_1_, b_2_, ..., b_n_) are two modules in Euclidean n-space, then the D value is the distance (d) from a to b and is defined as follows:

$\mathbf{d=}\sqrt{{\mathbf{（}\mathbf{a}_{\mathbf{1}}\mathbf{-}\mathbf{b}_{\mathbf{1}}\mathbf{）}}^{\mathbf{2}}\mathbf{+.......+}{\mathbf{（}\mathbf{a}_{\mathbf{n}}\mathbf{-}\mathbf{b}_{\mathbf{n}}\mathbf{）}}^{\mathbf{2}}}$ (3)

The paired targeted module were defined as the modules whose topological structural features exhibiting inverse temporal correlation with the primary clinical outcome (δmRSDay90). We further compare the expression levels of hub genes in these two modules between healthy volunteers and AIS patients, as well as across different efficacy groups at Day 90 using the Kruskal-Wallis test, adjusted with Bonferroni correction. Additionally, Spearman’s correlation analysis was utilized to assess the relationship between hub genes and clinical efficacy.

***Molecular docking***

We calculated the molecular binding energies between the 5 major active compounds of *DHI* (*Danshensu sodium, Protocatechualdehyde, p-coumaric acid, Rosmarinic acid, Salvianolic acid B*) and the receptor proteins encoded by the core genes in the paired targeted modules through the following steps: (1) The 2D structures of the 5 main active compounds in *DHI* were obtained from the PubChem website (https://pubchem.ncbi.nlm.nih.gov/). Their SDF files were downloaded and converted into PDB files using Open Babel 2.3.2 software. [22] (2) The structures of the receptor proteins were retrieved from the AlphaFold protein structure database(https://alphafold.ebi.ac.uk/). [23] PyMOL 2.3.4 software was used to dehydrate and remove ligands from these 14 proteins, while AutoDockTools software was employed for hydrogenation and charge balancing modifications. (3) The Grid Box command within the Grid program was used to access the Grid Options tool for processing each protein. The size of the ligand-binding pocket was defined by the number of grid points in each direction and the spacing between them. The grid spacing was initially set to 1, and the volume of the binding pocket was adjusted to ensure the pre-docking molecule could rotate within the box, even when fully extended. The center of the binding pocket was aligned with the center of the binding site. Finally, the receptor protein and ligand small molecule were converted into PDBQT format.(4) Molecular docking was performed using AutoDock Vina 1.1.2 [24] for each of the 14 proteins with the 5 ingredients. AutoDock Vina outputs the results as an affinity score, which is calculated by evaluating various factors, including the spatial effects of the receptor-ligand complex, repulsive interactions, hydrogen bonds, hydrophobic interactions, and the flexibility of the molecules. The affinity score is a core parameter of AutoDock Vina [24] and serves as a critical indicator for assessing whether a ligand can effectively bind to a receptor molecule. Generally, a docking score of less than -4.25 kcal/mol indicates binding activity between the ligand and the target, a score below -5.0 kcal/mol suggests better binding activity, and a score below -7.0 kcal/mol indicates highly binding activity.[24] (5) The interactions between the proteins and the five compounds were visualized in three dimensions using PyMOL.

**The combination effect model of the paired targeted modules**

We constructed a combination effect model as an exploratory, hypothesis-generating tool to quantify the time-dependent joint association of the paired modules with clinical outcome during *DHI* treatment. First, we respectively calculated the correlation coefficients between the expression profile levels of the corresponding genes from the paired targeted modules and the clinical outcomes at each time point (mRS on Day 0 and δmRS on days 14 and 90). Then We treated the correlation coefficients with δmRS of the *HBG2* module as N(t) which was a function of time. Similarly, we defined P(t) as the correlation coefficients with δmRS of the *TMEM108* module. Finally, we considered the combination effect of the paired targeted modules as a weighted sum of P(t) and N(t). Moreover, to better understand the therapeutic effect caused by *DHI*, we treated the effect of Day 0 as the baseline. In sum, the combination effect model was constructed as:

$f(t)=-\frac{\omega_{1}N_{t}+\omega_{2}P_{t}}{\sqrt{\omega_{1}^{2}N_{0}^{2}+\omega_{2}^{2}P_{0}^{2}}}$	(4)

where $\omega_{1}$+$\omega_{2}$ = 1. Note that on Day 90, there was a significant therapeutic effect of *DHI*, thus we set f(90) = 1. Based on this condition, it could be obtained that $\omega_{1}$ ≈ 0.229, and then $\omega_{2}$ = 0.701. We considered the 0 line marks the transition from disease to health. Then, we could get f(0) = −0.729, f(14) =0.227 and f(90) =1.

**Reference**

1. Li B, Wang Y, Lu J, et al. Evaluating the effects of Danhong injection in treatment of acute ischemic stroke: study protocol for a multicenter randomized controlled trial. *Trials.* 2015;16:516. doi:10.1186/s13063-015-1076-4

2. Gao Y, Ma B, Liu Q, Wang Y. Methodological Study and Establishment of the Diagnostic Scale for TCM Syndromes of Ischemic Stroke [in Chinese]. *J Tradit Chin Med.* 2011;52(24):2097-2101.

3. Wang XN, Zhou V, Liu Q, Gao Y, Zhou XH. Evaluation of the accuracy of diagnostic scales for a syndrome in Chinese medicine in the absence of a gold standard. *Chin Med.* 2016;11:35. doi:10.1186/s13020-016-0100-2

4. Barber PA, Demchuk AM, Zhang J, Buchan AM. Validity and reliability of a quantitative computed tomography score in predicting outcome of hyperacute stroke before thrombolytic therapy. *Lancet.* 2000;355(9216):1670-1674. doi:10.1016/S0140-6736(00)02237-6

5. Chinese Society of Neurology, Cerebrovascular Disease Group, Writing Group for the Diagnosis and Treatment Guidelines of Acute Ischemic Stroke. Chinese guidelines for the diagnosis and treatment of acute ischemic stroke 2010 [in Chinese]. *Chin J Neurol.* 2010;43(2):146-153. doi:10.3760/cma.j.issn.1006-7876.2010.02.022

6. Zhuo L, Xu L, Ye J, et al. Time trade-off value set for EQ-5D-3L based on a nationally representative Chinese population survey. *Value Health.* 2018;21(11):1330-1337. doi:10.1016/j.jval.2018.04.1370

7. The GUSTO Investigators. An international randomized trial comparing four thrombolytic strategies for acute myocardial infarction. *N Engl J Med.* 1993;329(10):673-682. doi:10.1056/NEJM199309023291001

8. Hacke W, Kaste M, Bluhmki E, et al. Thrombolysis with alteplase 3 to 4.5 hours after acute ischemic stroke. *N Engl J Med.* 2008;359(13):1317-1329. doi:10.1056/NEJMoa0804656

9. Cock PJ, Fields CJ, Goto N, Heuer ML, Rice PM. The Sanger FASTQ file format for sequences with quality scores, and the Solexa/Illumina FASTQ variants. *Nucleic Acids Res.* 2010;38(6):1767-1771. doi:10.1093/nar/gkp1137

10. Kim D, Langmead B, Salzberg SL. HISAT: a fast spliced aligner with low memory requirements. *Nat Methods.* 2015;12(4):357-360. doi:10.1038/nmeth.3317

11. Langmead B, Salzberg SL. Fast gapped-read alignment with Bowtie 2. *Nat Methods.* 2012;9(4):357-359. doi:10.1038/nmeth.1923

12. Li B, Dewey CN. RSEM: accurate transcript quantification from RNA-Seq data with or without a reference genome. *BMC Bioinformatics.* 2011;12:323. doi:10.1186/1471-2105-12-323

13. Love MI, Huber W, Anders S. Moderated estimation of fold change and dispersion for RNA-seq data with DESeq2. *Genome Biol.* 2014;15(12):550. doi:10.1186/s13059-014-0550-8

14. Wu T, Hu E, Xu S, et al. clusterProfiler 4.0: a universal enrichment tool for interpreting omics data. *Innovation (Camb).* 2021;2(3):100141. doi:10.1016/j.xinn.2021.100141

15. Langfelder P, Horvath S. WGCNA: an R package for weighted correlation network analysis. *BMC Bioinformatics.* 2008;9:559. doi:10.1186/1471-2105-9-559

16. Langfelder P, Zhang B, Horvath S. Defining clusters from a hierarchical cluster tree: the Dynamic Tree Cut package for R. *Bioinformatics.* 2008;24(5):719-720. doi:10.1093/bioinformatics/btm563

17. Li B, Liu J, Zhang Y, et al. Quantitative identification of compound-dependent on-modules and differential allosteric modules from homologous ischemic networks. CPT Pharmacometrics Syst Pharmacol. 2016;5(10):575-584. doi:10.1002/psp4.12127

18. Langfelder P, Luo R, Oldham MC, Horvath S. Is my network module preserved and reproducible? *PLoS Comput Biol.* 2011;7(1):e1001057. doi:10.1371/journal.pcbi.1001057

19. Wang P, Yu Y, Liu J, et al. IMCC: a novel quantitative approach revealing variation of global modular map and local inter-module coordination among differential drug's targeted cerebral ischemic networks. *Front Pharmacol.* 2021;12:637253. doi:10.3389/fphar.2021.637253

20. Shannon P, Markiel A, Ozier A, et al. Cytoscape: a software environment for integrated models of biomolecular interaction networks. *Genome Res.* 2003;13(11):2498-2504. doi:10.1101/gr.1239303

21. Liu J, Li D, Dong W, et al. Detection of an anti-angina therapeutic module in the effective population treated by a multi-target drug Danhong injection: a randomized trial. *Signal Transduct Target Ther.* 2021;6(1):329. doi:10.1038/s41392-021-00741-x

22. O'Boyle NM, Banck M, James CA, Morley C, Vandermeersch T, Hutchison GR. Open Babel: an open chemical toolbox. *J Cheminform*. 2011;3:33. doi:10.1186/1758-2946-3-33

23. Jumper J, Evans R, Pritzel A, et al. Highly accurate protein structure prediction with AlphaFold. *Nature.* 2021;596(7873):583-589. doi:10.1038/s41586-021-03819-2

24. Trott O, Olson AJ. AutoDock Vina: improving the speed and accuracy of docking with a new scoring function, efficient optimization, and multithreading. *J Comput Chem.* 2010;31(2):455-461. doi:10.1002/jcc.21334

**Figure S1 The standard of the fingerprint electropherogram of *DHI*.**

The fingerprint electropherogram is obtained using Agilent 1100 Series High Performance Liquid Chromatograph (Agilent Chemstation Workstation) or Shimadzu LC-20AT High Performance Liquid Chromatograph (Shimadzu LC Solution Chromatographic Workstation) and Kromasil C18 chromatographic column (250mm×4.6mm,5μm). The main components in the injection includes: 1. Danshensu sodium; 2.Protocatechualdehyde; 3. p-coumaric acid; 4. Rosmarinic acid; 5. Salvianolic acid B. The fingerprint electropherogram of the test sample of Danhong injection should show the same peak value as the chromatographic peak retention time of the corresponding reference solution. According to the similarity evaluation system for fingerprint electropherogram of reference solution, the similarity should be more than 0.9 between the test sample (using in our trial) and the reference solution.

1.*Danshensu sodium* C_9_H_10_O_5_	2_._ *Protocatechualdehyde* C_7_H_6_O_3_

3. *p-coumaric acid* C_9_H_8_O_3_	4. Rosmarinic acid C_18_H_16_O_8_

 5. *Salvianolic acid B* C_36_H_30_O_16_

**Figure S2 The chemical structure of the 5 main component content in *DHI*.**

*Danhong* injection (*DHI*) should contain Danshensu sodium not less than 0.80mg, protocatechualdehyde not less than 0.10mg, p-coumaric acid not less than 20μg, rosmarinic acid not less than 0.10mg, and salvianolic acid B not less than 0.16mg.

**Figure S3 Effect of *DHI* treatment by prespecified subgroups of combination therapy. *** Comparisons of Odds Ratios are unadjusted for multiplicity. CCBs: calcium channel blockers.

**Figure S4 The number of DEGs modulated by *DHI* at 14 days (lower panels) and 90 days (upper panels)**

**Figure S5 Gene Ontology (GO) and Kyoto Encyclopedia of Genes and Genomes (KEGG) pathway enrichment analysis of DEGs.**

**Figure S6 The differentially expressed modules (DEMs) targeted by *DHI*.**

A. A hierarchical cluster tree (dendrogram) for gene co-expression networks in the population responsive to *DHI* treatment. 64 modules were detected within the coexpressed gene network of the group that exhibited a good response to *DHI.* B. The DEMs targeted by *DHI.* The 19 DEMs targeted by *DHI*, were identified with Z-scores < 0 after comparing the *DHI*-responsive group with baseline, the control group, and the unresponsive group at 90 days. C.Module renaming based on hub genes of differentially expressed modules (DEMs) modulated by *DHI*. The 19 DEMs were were systematically reannotated according to their respective hub genes, which were identified through integrative analysis of gene significance (GS) for δmRSDay90 and module membership (MM) scores.

**Figure S7 Bar graph of enriched GO terms and KEGG pathways of “HBG2”(A) and “TMEM108”(B).**

**Figure S8 Molecular docking binding energies of *Rosmarinic Acid* and *Salvianolic Acid B* with the gene-encoded proteins in the paired targeted modules.**

These 2 compounds in *DHI* displayed high-affinity binding to core gene-encoded proteins, *TMEM108* (A), *HBG2* (B), and *PHYHIP*(C), in the paired targeted modules.

**Table S1 Full list of “Danhong-Stroke Trial Investigators”**

**Clinical Trial Steering Committee**

Prof. Yong-Yan Wang (Chair), Institute of Basic Research in Clinical Medicine, China Academy of Chinese Medical Sciences

Prof. Xiao-Xi Du (Member), State Food and Drug Administration

Prof. Bo-Li Zhang (Member), China Academy of Chinese Medical Sciences

Prof. Run-Lin Gao (Member), Fuwai Hospital Chinese Academy of Medical Sciences

Prof. Wei-Liang Weng (Member), Xi-yuan Hospital Affiliated to China Academy of Chinese Medical Sciences

Dr. Ai-Ping Lv (Member), 1. Institute of Basic Research in Clinical Medicine, China Academy of Chinese Medical Sciences; 2. School of Chinese Medicine, Hong Kong Baptist University

Prof. Ming-Hui Yang (Member), Chinese PLA General Hospital

**Data Monitoring Committee**

Prof. Chun-Ti Shen (Chair), Changzhou TCM Hospital Affiliated to Nanjing University of Chinese Medicine

Prof. Qi-Guang Chen (Member, independent statistician), Southeast University

Prof. Hua-Qiang Zhang (Member), Jiangsu Provincial Commission of Health and Family Planning

Dr. Hong-Wu Wang (Member), Tianjin University of Traditional Chinese Medicine

Dr. Xiao-Chun Sun (Member), Beijing Kelunrui Pharmaceutical Technology Co. Ltd

Prof. Cheng-Shu Wang (Member), Zhongda Hospital Southeast University

Dr. Hai-Xia Dang (Member), China Academy of Chinese Medical Sciences

Dr. Jun Liu (Member), Institute of Basic Research in Clinical Medicine, China Academy of Chinese Medical Sciences

**Outcome Evaluation Committee**

Prof. Yi-Huai Zou (Chair), Dongzhimen Hospital of Beijing University of Chinese Medicine

Prof. Xing-Quan Zhao (Member), Beijing Tiantan Hospital, Capital Medical University

Prof. Yun-Dai Chen (Member), Chinese PLA General Hospital

Prof. Qing-Quan Liu (Member), Beijing hospital of traditional chinese medicine

Prof. Sheng-Xian Wu (Member), Dongzhimen Hospital of Beijing University of Chinese Medicine

**Independent Statistician of the Trial**

Prof. Bi-Wei Chen, Ph.D., School of Public Health, Southeast University

**Principal Investigators and Coordinators (according to province and hospital center, with numbers of patients in parentheses)***

**Beijing (5 hospitals - 88 patients)**

PLA Rocket Force General Hospital (31): Lei Wang

Beijing Tiantan Hospital, Capital Medical University (29): Xing-quan Zhao, Yin-Long Wang, Jing-Jing Lu

Xuan Wu Hospital, Capital Medical University (27): Li Gao, Jue-Xian Song

Peking University First Hospital (1) **(Terminated)**: Xue-Zhi Zhang, Wei Liu

Peking University of People's Hospital **(Withdrawn):** Xu-guang Gao

**Chongqing (2 hospitals - 36 patients)**

Chongqing Three Gorges Central Hospital (18): Wei Xue, Wei Feng

Xinqiao Hospital of Third Military Medical University (18): Qing-Wu Yang, Xiao-Yan Chen

**Guangdong (3 hospitals - 28 patients)**

Guangdong Second Provincial General Hospital (19): Xintong Liu, Feng Wei

The Second People's Hospital of Shenzhen (6): Xiaohua Xiao, Huo-You Hu, Ying-Hui Shao

Guangzhou hospital of integrated Chinese and Western medicine (3): Chao-Jun Chen, Xiang Jia

**Hebei (4 hospitals - 24 patients)**

Affiliated Hospital of Chengde Medical University (8): Yan-Jun Gao,Guang-Mei Jiao

Handan Central Hospital (13): Jun-Tao Li, Jing-Xue

The Second Central Hospital of Baoding City (3)**:** Shuangxia Du, Yan-Zun Yuan

The Second Affiliated Hospital of Xingtai Medical College **(Withdrawn)**: Feng-qing Gao

**Heilongjiang (2 hospitals - 5 patients)**

The Second Affiliated Hopital of Harbin Medical University (5): Li-hua Wang, Hui-Xue Zhang

The People's Hospital of Daqing City **(Withdrawn)**: De-xin Luo

**Henan (7 hospitals - 143 patients)**

The First Affiliated Hospital of Henan University of T.C.M (85): Xin-Zhi Wang, Hai-Yan Yang

People's Hospital of Zhengzhou (11): Lai-hui Feng

The 7th People's Hospital of Zhengzhou (12): Jun-Xing Dong

The Second Chinese Medical Hospital of Luoyang City (20): Xue-Wen Huo, An-Yu Zhou

Nanyang T.C.M. Hospital (2): Zeng-Xiang Li, Ya-Xiang Li, Ran Li

Zhoukou Hospital of T.C.M (5): Yan-Xia Zhang, Gao Liang

Nanshi Hospital of Nanyang (8): Yu-kun Yang, Yan-Zhen liu, Xiao-Hua Xiao

**Hubei (8 hospitals - 126 patients)**

Renmin Hospital, Wuhan University (30): Zhao-Hui Zhang

Zhongnan Hospital, Wuhan University (17): Yu-Min Liu

Zhongshan Hospital of Hubei Province (The Third People's Hospital of Hubei Province) (36): Xiao-Rong Deng, Min Du, Hong-Ping Wan

The Central Hospital of Wuhan (12): Lin-Hong Zhang

Fifth Hospital in Wuhan (12): Ming Li

Tianyou Hospital Affiliated to Wuhan University of Science & Technology (18): Jun Peng

Huangpi District Hospital of T.C.M (1): Jun-Hua He

Hubei Provincial Hospital of T.C.M **(Withdrawn)**: Yan-Bing Ding

**Hunan (4 hospitals - 107 patients)**

Xiangya Hospital, Central South University (22): Xiao-Su Yang, Jun Yin

Changsha Central Hospital (30): Zhu-Qin Liu

The First Hospital of Changsha (19): Ai-Min Wang, Qing-Fang Liu

The Second People's Hospital of Hunan Province /Brain Hospital of Hunan Province (36): Qiu-Ting Liu

**Jiangsu (3 hospitals - 40 patients)**

Nanjing Hospital of TCM. **(Terminated)** (6): Shu-Ping Cao, Yang Zhao

Nanjing Drum Tower Hospital **(Terminated)** (8): Yun Xu, Yang Zhang

The Second Affiliated Hospital of Nanjing Medical University (26): Zhi-Qiang Gao, Wei Liu, Peng Jia

**Jilin (7 hospitals - 223 patients)**

The Second Hospital of Jilin University **(Terminated)** (1): Ting-Min Yu, Jia Fan

The First Hospital Affiliated to Jilin University (48): Jia-Feng Chen, Xin-Hui Wang

Jilin Province People's Hospital (36): Hong-Xia Sun, Song Liu, Bo-Na Liang

The Affiliated Hopital to Changchun University of Chinese Medicine **(Terminated)** (2): Jian-Jun Zhao, Hong-Mei Nan

China-Japan Union Hospital of Jilin University (36): Zhong-Xin Xu, Jin-Ting He, Jing Mang

Liaoyuan Traditional Chinese Medical Hospital (100): Hong-Wei Dai, Zhong-Min Jie

Yanbian University Hospital (Yanbian Hospital) **(Withdrawn)**: Chao Liu

**Liaoning (8 hospitals - 269 patients)**

Affiliated Zhongshan Hospital of Dalian University (36): Qiang Ma, Kun Liu

People's Liberation Army No.202 Hospital (43): Zhi-Lin Jiang, Shu-Yang Lin, Cong Li

People's Liberation Army No.463 Hospital (42): Yan-Song Li, Sheng-Nan Cai

Dandong First Hospital (5): Li-Shu Wan, Mei-Yu Li

Panjin Central Hospital (74): Yan-Hua Zhou, Ying Zhao

Anshan Central Hospital (21): Zhen Jiao, Wei Hu

The General Hospital of Shenyang Military (48): Hui-Sheng Chen, Lin Tao

The Second Affiliated Hospital of Liaoning University of Traditional Chinese **(Withdrawn)**: Fu-Ying Jiao

**Neimenggu (1 hospitals - 30 patients)**

Affiliated Hospital of Chifeng University (30): Wei-Li Zhao,

**Shaanxi (5 hospitals - 168 patients)**

Affiliated Hospital of Shaanxi University of Chinese Medicine (72): Yong-Mei Yan, Yan-Ni Liu, Hui Zhang

Shaanxi Provincial People's Hospital (30): Qian Yang, Qi Lei, He-Nan Guo

The PLA 451 Hospital (18): Li-Yi Chi, Yang-Feng Liu, Guan-Bing Bai

The Second Affiliated Hospital of Shaanxi University of T.C.M (42): Ju-Mei Du, Jiang Yan, Ying Bian

Shaanxi Province Hospital of T.C.M **(Terminated)** (6): Zu-Cheng Han, La-Qun Tian, Cui Wang

**Shanxi (2 hospitals - 67 patients)**

The Second Affiliated Hospital to Shanxi Medical University (24): Guang-Lai Li,

Fenyang Hospital of Shanxi Province (43): Bao-Ai Wang

**Sichuan (2 hospitals - 44 patients)**

Teaching Hospital of Chengdu University of Traditional Chinese Medicine (38): Yun Lu, Fan-Lin Meng

The First Affiliated Hospital of Chengdu Medical College (6): Jie Yang

**Tianjin (2 hospitals - 35 patients)**

First Teaching Hospital of Tianjin University of Traditional Chinese Medicine (35): Zhi-Hong Meng

Tianjin Medical University General Hospital **(Withdrawn)**: Fu-Dong Shi

**Zhejiang (5 hospitals - 72 patients)**

Xin Hua Hospital of Zhejiang Province (33): Bin Xu, You Wu, Shun-Li Liang

Zhejiang Provincial People's Hospital (20): Yu Geng, Yi-Qi Wang, Li-Ping Du

Zhejiang Provincial Hospital of T.C.M (18): Qun Hou, Li-Ping Zhang

Huzhou Central Hospital **(Terminated)** (1): Ya-Xian Wang, Zheng-Gang Hu

The First Affiliated Hospital of College of Medicine, Zhejiang University **(Withdrawn)**: Ben-Yan Luo

**Shanghai (2 hospitals - 0 patients)**

Xuhui District Central Hospital of Shanghai City **(Withdrawn)**: Hui Zhang

The Sixth People's Hospital of Shanghai City **(Withdrawn)**: Qing-Ping Huo

*Seventy-two centers were activated and approved to participate. Ten centers (marked “Withdrawn”) were activated but withdrew before enrolling any patient and contributed no data; the remaining 62 centers enrolled patients, consistent with the 62 enrolling centers in the trial registry. Centers marked “Terminated” enrolled patients before early termination.

**Table S2 The α spending using an O’BrienFleming boundary in the two inter analyses**

| *K* | $t_{k}$ | $Z_{k}$ | $\alpha_{k}$(single-side) | $\alpha_{k}$ (two-side) |
| --- | --- | --- | --- | --- |
| 3 | 1/3 | 3.471 | 0.00025 | 0.0005 |
|  | 2/3 | 2.454 | 0.00725 | 0.0145 |
|  | 3/3 | 2.004 | 0.0225 | 0.045 |

**Table S3 The treatment effect on the primary outcome (modified Rankin Scale 0-1) in the two interim analyses in intention-to-treat population and the Per Protocol Population***

| **Interim analysis** | **Populations** | ***DHI*(%)** | **Control(%)** | **Difference(%)** | **Z-value** | **P-value** | **CP value** |
| --- | --- | --- | --- | --- | --- | --- | --- |
| 1 | ITT | 56.50 | 48.94 | 7.56 | 1.19 | 0.23 | 0.55 |
|  | PPS | 58.24 | 49.46 | 10.38 | 1.37 | 0.17 | / |
| 2 | ITT | 60.94 | 54.82 | 6.12 | 1.41 | 0.16 | 0.34 |
|  | PPS | 62.43 | 55.03 | 7.40 | 1.67 | 0.09 | / |

* *DHI* =Danhong injection, ITT= intention-to-treat , PPS=Per Protocol Set. The difference between two groups were done using chi-square test.

**Table S4 Trial Inclusion and Exclusion Criteria**

**Inclusion criteria**

(1). Female or male inpatients.

(2). Age: 18–70 years.

(3). Clinical diagnosis of ischemic stroke causing a measurable neurological deficit defined as impairment of language, motor function, cognition and/or gaze, vision or neglect. Ischemic stroke is defined as an event characterized by the sudden onset of an acute focal neurologic deficit presumed to be due to cerebral ischemia after computed tomography (CT) scan excludes hemorrhage.

(4). Clinical diagnosis of "*Xueyu Zheng*" (Blood Stasis Syndrome) as the scores of Chinese Medicine Symptom Diagnostic Scale of "*Xueyu Zheng*" in ischemic stroke ≥ 20.

(5). Onset of symptoms within 1 week prior to initiation of study drug administration.

(6). Patients with a clinical diagnosis of cerebral anterior circulation obstruction.

(7). 4≤National Institutes of Health Stroke Scale (NIHSS) score <20.

(8). Patient is willing to participate voluntarily and to sign a written patient informed consent. Informed consent will be obtained from each patient or the subject's legally authorized representative or relatives.

**Exclusion criteria**

(1). Evidence of intracranial hemorrhage (ICH) or other cerebral diseases (e.g., vascular malformation, tumor, abscess or multiple sclerosis) on the CT or magnetic resonance imaging (MRI) scan.

(2). Patients who underwent thrombolysis or endovascular treatment.

(3). Known history of allergy or suspected allergy to the study drug.

(4). Fasting blood glucose <2.8 or >16.8 mmol/l or with severe complications due to diabetes (e.g., peripheral neuropathy, or diabetic gangrene).

(5). Liver function impairment with the value of alanine aminotransferase (ALT) or aspartate aminotransaminase (AST) over 1.5-fold the upper limit of normal range.

(6). Renal dysfunction with the value of serum creatinine over 1.5-fold the upper limit of normal range.

(7). Severe cardiac dysfunction on echocardiogram or the New York Heart Association (NYHA) heart functional class III or IV.

(8). History of prior stroke with disability.

(9). Complicated with atrial fibrillation.

(10). Severe stroke as assessed by appropriate imaging techniques (e.g., massive cerebral infarction affecting more than one lobe of the brain or over one-third of the blood-supply area of the middle cerebral artery).

(11). Physical disability before onset.

(12).Hemorrhagic tendency or severe or dangerous bleeding within the prior 3 months.

(13).Suspected alcohol addiction or drug abuse.

(14).Severe complications that would make the condition more complicated as assessed by the investigator.

(15).Women who are pregnant, lactating or have a positive pregnancy test, or women who plan to become pregnant in the subsequent 6 months.

(16).Women who have a menstrual period at baseline.

(17).Patients who are participating in other trials or have participated in other trials within the prior 3 months.

**Table S5 The concomitant medications between two groups***

| **Concomitant drugs** | ***DHI* (n=1002)** | **Control (n=503)** | **P value** |
| --- | --- | --- | --- |
| **Clopidogrel** | | | |
| No n(%) | 323 (32.24) | 164 (32.60) | 0.89 |
| Yes n(%) | 679 (67.76) | 339 (67.40) |  |
| **Aspirin** | | | |
| No n(%) | 127 (12.67) | 65 (12.92) | 0.89 |
| Yes n(%) | 875 (87.33) | 438 (87.08) |  |
| **Stains** | | | |
| No n(%) | 112 (11.18) | 57 (11.33) | 0.93 |
| Yes n(%) | 890 (88.82) | 446 (88.67) |  |
| **Anticoagulants^$^** | | | |
| No n(%) | 924 (92.22) | 460 (91.45) | 0.61 |
| Yes n(%) | 78 (7.78) | 43 (8.55) |  |
| **Neuroprotective drugs^✝^** | | | |
| No n(%) | 145 (14.47) | 61 (12.13) | 0.21 |
| Yes n(%) | 857 (85.53) | 442 (87.87) |  |
| **Calcium channel blockers** | | | |
| No n(%) | 503 (50.20) | 280 (55.67) | 0.045 |
| Yes n(%) | 499 (49.80) | 223 (44.33) |  |
| **Angiotensin II-receptor blockers** | | | |
| No n(%) | 726 (72.46) | 359 (71.37) | 0.66 |
| Yes n(%) | 276 (27.54) | 144 (28.63) |  |
| **Angiotensin converting enzyme inhibitors** | | | |
| No n(%) | 879 (87.72) | 446 (88.67) | 0.59 |
| Yes n(%) | 123 (12.28) | 57(11.33) |  |
| **Antidiabetic Agents^#^** | | | |
| No n(%) | 739 (73.75) | 383 (76.14) | 0.32 |
| Yes n(%) | 263 (26.25) | 120 (23.86) |  |

^*^ The concomitant medications were analysis according to the intention-totreat population. P values were not adjusted for multiple testing. The differences of the concomitant medications between the treatment groups were not significant, with the exception of the proportions of patients using calcium channel blockers. *DHI* denotes Danhong injection.

^$^ Anticoagulants includes heparin sodium, low molecule heparin calcium, argatroban,dabigatran and rivaroxaban. And the use of each anticoagulant is similar between two groups.

^✝^Neuroprotective drugs includes edaravone, oxiracetam, cattle encephalon glycoside and ignotin injection, muscular amino acids and nucleosides, citicoline sodium, idebenone tablets, piracetam, gangliosides, mouse nerve growth factor injection,cerebroprotein hydrolysate. And the use of each neuroprotective drug is similar between two groups.

^#^ Antidiabetic agents includes metformin, different kinds of insulins, glucosidase inhibitors, sulfonylurea, insulin secretagogue and thiazolidinediones. And the use of each antidiabetic agent is similar between two groups.

**Table S6 Treatment Effect in the Per Protocol Population (n=1273)^✭^**

| **Outcomes** | ***DHI*** | | | **Controls** | | | **Odd ratio (95% CI)** | **P-value** |
| --- | --- | --- | --- | --- | --- | --- | --- | --- |
|  | No. of total Participants (No of miss data) | | No. of patients (% with events) | No. of total Participants | | No. of patients (% with events) |  |  |
| **Primary outcome** | | | | | | | | |
| **mRS 0–1 at Day 90** | 841 (0) | 591 (70.27) | | 432 (0) | 275  (63.66) | | Unadjusted:  1.35 (1.06,1.73) | 0.02 |
|  |  |  |  |  |  |  | Adjusted:  1.62 (1.19,2.19)^†^ | 0.002 |
| **Secondary outcomes** | | | | | | | | |
| ***Xueyu Zheng* scores ≥ 70% from baseline at 90 days*** | 838 (3) | 530 (63.25) | | 428 (4) | 245 (57.24) | | 1.29 (1.01,1.63) | 0.04 |
| **BI of 90-100 at 90 days** | 839 (2) | 687(81.88) | | 428 (4) | 347 (81.07) | | 1.06 (0.78,1.42) | 0.73 |
| **δ NIHSS ≥4 or NIHSS 0 to 1 at 90 days** | 839 (2) | 704 (83.91) | | 428 (4) | 346 (80.84) | | 1.24 (0.91,1.67) | 0.17 |
| **Global disability at 90 days** | 841 (0) | 118(14.03) | | 432 (0) | 64 (14.81) | | 0.94 (0.68,1.30) | 0.71 |
| **Mean EQ-5D score at 90 days** | 839 (2) | 0.94±0.12 | | 428 (4) | 0.95±0.09 | | / | 0.72 |

**^✭^**Plus-minus values are means ± SD. Data are n (%) or medians (interquartiles). *DHI*: Danhong injections; mRS: modified Rankin Scale; BI: Barthel Index; NIHSS: National Institutes of Health Stroke Scale; EQ-5D: EuroQoL Group 5-Dimension Self-Report Questionnaire; TIA: transient ischemic attack.

†Values were adjusted for age, mRS, NIHSS score, ASPECTS score, time from stroke onset to randomization, history of previous stroke or TIA, hypertension, hyperlipidemia, diabetes mellitus, ischemic heart disease and ECG showing atrial fibrillation. The odd ratio was estimated in a logistic-regression model and indicates the increase in odds of moving from a lower level of disability or stroke severity to a higher one in the *DHI* group relative to the control group. An odds ratio of higher than 1 favors the *DHI*.

* *Xueyu Zheng* Score is obtained from the Efficacy Evaluation Scale of Chinese Medicine Symptom in *Xueyu Zheng*, evaluated on a symptom-specific weighted Visual Analogue Scale (VAS)-based scoring system for the 12 diagnostic items of *Xueyu Zheng* shown in Table S8; the more severe the symptom is, the higher the score be determined. If the symptom is disappeared, the score will be recorded as 0.

††EQ-5D is a standardized instrument for the measurement of health status. Scores range from 0.1702 to 1.00 [6], with higher scores indicating a better quality of life.

**Table S7 Interaction effects between characteristics of subgroups and treatments**

| **Characteristics of subgroups** | **Models** | **Estimate** | **StdErr** | **Wald ChiSq** | **Prob ChiSq** |
| --- | --- | --- | --- | --- | --- |
| Age (yrs) | grp*Age | -0.16 | 0.11 | 2.12 | 0.145 |
| Sex | grp*Sex | 0.26 | 0.12 | 4.41 | 0.036 |
| Race | grp*Race | 0.40 | 0.45 | 0.80 | 0.370 |
| BMI(Kg/m^2) | grp*BMI | -0.06 | 0.17 | 0.12 | 0.726 |
|  | grp*BMI | 0.22 | 0.25 | 0.81 | 0.368 |
| NIHSS | grp*NIHSS | -0.11 | 0.12 | 0.73 | 0.394 |
| ASPECTS | grp*ASPECTS | 0.21 | 0.16 | 1.68 | 0.195 |
| Onset to treatment | grp*Onset to treatment | 0.22 | 0.17 | 1.75 | 0.186 |
| Diabetes | grp*Diabetes | -0.14 | 0.13 | 1.08 | 0.298 |
| Hypertension | grp*Hypertension | 0.32 | 0.12 | 7.47 | 0.006 |
| Hyperlipidemia | grp*Hyperlipidemia | 0.32 | 0.23 | 1.97 | 0.161 |
| Ischaemic heart disease | grp*Ischaemic heart disease | 0.18 | 0.18 | 1.01 | 0.316 |
| Any past stroke or TIA | grp*Past stroke or TIA | -0.01 | 0.21 | 0.00 | 0.949 |
| Ischemic location of cerebral side | grp*brain_side | -0.22 | 0.17 | 1.77 | 0.183 |
|  | grp*brain_side | 0.25 | 0.23 | 1.24 | 0.266 |

**Table S8 The efficacy evaluation scale of Chinese Medicine Symptom in “*Xueyu Zheng*” of ischemic stroke**

| Symptoms | Visual Analogue Scale* | Score |
| --- | --- | --- |
| Hemiplegia | 0------------------------------------10 |  |
| Numbness of limbs | 0------------------------------------10 |  |
| Dimmish and darkish complexion | 0------------------------------------9 |  |
| Dark purple lips | 0------------------------------------8 |  |
| Rough skin | 0------------------------------------4 |  |
| Pain in a fixed body location | 0------------------------------------5 |  |
| Purple or darkish tongue | 0------------------------------------10 |  |
| Ecchymosis on the tongue | 0------------------------------------10 |  |
| Purple sublingual vessels | 0------------------------------------10 |  |
| Sublingual varices | 0------------------------------------8 |  |
| Unsmooth pulse | 0------------------------------------8 |  |
| Intermittent pulse | 0------------------------------------1 |  |

* According to the severity of the symptoms, the more severe the symptom is, the higher the score be determined. If the symptom is disappeared, the score will be recorded as 0. The mean total score of symptoms in a questionnaire of *Xueyu Zheng* will be analyzed and compared between two groups at each visit. Moreover, the proportions of patients with improvement in *Xueyu Zheng* between the two groups will be recorded at each visit. Significant “*Xueyu Zheng*” improvement is defined as at least 70% reduction in the *Xueyu Zheng* score. This Efficacy Evaluation Scale (Visual Analogue Scale–based) is distinct from the Chinese Medicine Symptom Diagnostic Scale of “Xueyu Zheng” used to establish eligibility (each item scored present [assigned the item’s full weighted score] or absent [0]; total ≥ 20 required). Although the two scales share the same 12 evaluation items and a maximum total score of 93, they use different scoring methods: the diagnostic scale scores presence or absence, whereas the efficacy scale grades the severity of each present item on the Visual Analogue Scale from 0 up to that item’s maximum.

**Table S9 The safety outcomes between two groups in the safety population (n = 1493)**

| **Safety outcomes** | ***DHI***  **(N=994)** | **Control**  **(N=499)** | **Odds ratio**  **(95% CI)** | **P value** |
| --- | --- | --- | --- | --- |
| **Patients with any adverse event** | 481 (48.39) | 230 (46.09) | 1.10(0.88,1.36) | 0.40 |
| **Patients with any serious adverse event** | 45 (4.53) | 26 (5.21) | 0.86(0.53,1.42) | 0.56 |
| Pneumonia | 7 (0.70) | 8 (1.60) | 0.44(0.16-1.21) | 0.11 |
| **Mortality at 90 days** | 2 (0.20) | 4 (0.80) | 0.25(0.05,1.37) | 0.08 |
| **New-onset major vascular events at 90 days** | 24 (2.41) | 18 (3.61) | 0.66(0.36,1.23) | 0.19 |
| Ischemic stroke | 22 (2.21) | 16 (3.21) | 0.68(0.36,1.31) | 0.25 |
| Hemorrhagic stroke | 2 (0.20) | 0 (0.00) | / | 0.56 |
| TIA | 0 (0.00) | 1 (0.20) | / | 0.33 |
| Myocardial infarction | 0 (0.00) | 1 (0.20) | / | 0.33 |
| Vascular-death | 2 (0.20) | 4 (0.80) | 0.25(0.05,1.37) | 0.08 |
| **Moderate hemorrhages at 90 days^#^** | 15 (1.51) | 5 (1.00) | 1.51(0.55,4.19) | 0.42 |
| **Severe hemorrhages at 90 days^#^** | 0 (0.00) | 0 (0.00) | — | — |

^#^ Moderate hemorrhages events indicate the hemorrhages events which need transfusion but not up to the standard of severe hemorrhage, including absolute decrease of hemoglobin/hematocrit, severe disability, intraocular hemorrhage with severe visual field defect, etc. Severe hemorrhages were defined according to the GUSTO bleeding criteria as fatal bleeding, intracranial hemorrhage, or bleeding requiring transfusion, infusion, vasoconstrictor drugs, or surgical intervention.

**Table S10 The significant adverse events by MedDRA preferred term**

| **SOC ( PT )** | ***DHI* group (N=994)** | | | **Control group (N=499)** | | | **Odds ratio (95% CI)** | **P value** |
| --- | --- | --- | --- | --- | --- | --- | --- | --- |
|  | **No. of events** | **No. of patients** | **Incidence %** | **No. of events** | **No. of patients** | **Incidence %** |  |  |
| **All** | **1005** | **481** | **48.39** | **473** | **230** | **46.09** | **1.10 (0.88, 1.36)** | **0.40** |
| **Metabolism and nutrition disorders** | **74** | **63** | **6.34** | **35** | **24** | **4.81** | **1.34 (0.83, 2.17)** | **0.23** |
| Type 2 diabetes mellitus | 7 | 7 | 0.70 | 4 | 4 | 0.80 |  |  |
| Hypokalaemia | 15 | 15 | 1.51 | 5 | 5 | 1.00 |  |  |
| Hyperhomocysteinaemia | 14 | 14 | 1.41 | 8 | 8 | 1.60 |  |  |
| Hyperlipidaemia | 9 | 8 | 0.80 | 3 | 3 | 0.60 |  |  |
| Decreased appetite | 5 | 5 | 0.50 | 0 | 0 | 0.00 |  |  |
| Dyslipidaemia | 10 | 10 | 1.01 | 3 | 3 | 0.60 |  |  |
| **Ear and labyrinth disorders** | **2** | **2** | **0.20** | **1** | **1** | **0.20** | **1.00 (0.09, 11.10)** | **1.00** |
| **Hepatobiliary disorders** | **56** | **52** | **5.23** | **24** | **23** | **4.61** | **1.14 (0.69, 1.89)** | **0.60** |
| Hepatic function abnormal | 32 | 32 | 3.22 | 18 | 18 | 3.61 |  |  |
| Hepatic steatosis | 16 | 16 | 1.61 | 2 | 2 | 0.40 |  |  |
| **Infections and infestations** | **9** | **9** | **0.91** | **4** | **4** | **0.80** | **1.13 (0.35, 3.69)** | **0.84** |
| **Investigations** | **92** | **66** | **6.64** | **47** | **37** | **7.41** | **0.89 (0.59, 1.35)** | **0.58** |
| Alanine aminotransferase increased | 12 | 12 | 1.21 | 5 | 5 | 1.00 |  |  |
| White blood cells urine positive | 6 | 6 | 0.60 | 2 | 2 | 0.40 |  |  |
| Red blood cells urine positive | 4 | 4 | 0.40 | 4 | 4 | 0.80 |  |  |
| Occult blood positive | 3 | 3 | 0.30 | 4 | 4 | 0.80 |  |  |
| Aspartate aminotransferase increased | 9 | 9 | 0.91 | 2 | 2 | 0.40 |  |  |
| **Nervous system disorders** | **97** | **87** | **8.75** | **67** | **50** | **10.02** | **0.86 (0.60, 1.24)** | **0.42** |
| Ischemic stroke | 22 | 22 | 2.21 | 16 | 16 | 3.21 |  |  |
| Headache | 17 | 17 | 1.71 | 9 | 9 | 1.80 |  |  |
| Dizziness | 15 | 14 | 1.41 | 14 | 14 | 2.81 |  |  |
| **Injury,poisoning and procedural complications** | **9** | **9** | **0.91** | **6** | **6** | **1.20** | **0.75 (0.27, 2.12)** | **0.59** |
| Fracture | 1 | 1 | 0.10 | 3 | 3 | 0.60 |  |  |
| **Musculoskeletal and connective tissue disorders** | **30** | **30** | **3.02** | **16** | **13** | **2.61** | **1.16 (0.60, 2.25)** | **0.65** |
| Back pain | 2 | 2 | 0.20 | 3 | 3 | 0.60 |  |  |
| Musculoskeletal pain | 4 | 4 | 0.40 | 2 | 2 | 0.40 |  |  |
| Arthralgia | 3 | 3 | 0.30 | 3 | 3 | 0.60 |  |  |
| Pain in extremity | 7 | 7 | 0.70 | 2 | 2 | 0.40 |  |  |
| Intervertebral disc disorder | 5 | 5 | 0.50 | 0 | 0 | 0.00 |  |  |
| **Surgical and medical procedures** | **2** | **2** | **0.20** | **1** | **1** | **0.20** | **1.00 (0.09, 11.10)** | **1.00** |
| **Respiratory, thoracic and mediastinal disorders** | **107** | **88** | **8.85** | **47** | **43** | **8.62** | **1.03 (0.70, 1.51)** | **0.89** |
| Upper respiratory tract infection | 46 | 46 | 4.63 | 23 | 23 | 4.61 |  |  |
| Cough | 23 | 22 | 2.21 | 7 | 7 | 1.40 |  |  |
| Productive cough | 9 | 9 | 0.91 | 3 | 3 | 0.60 |  |  |
| Pneumonia | 7 | 7 | 0.70 | 8 | 8 | 1.60 |  |  |
| **Psychiatric disorders** | **73** | **68** | **6.84** | **41** | **39** | **7.82** | **0.87 (0.58, 1.30)** | **0.49** |
| Anxiety | 4 | 4 | 0.40 | 7 | 7 | 1.40 |  |  |
| Insomnia | 42 | 42 | 4.23 | 20 | 20 | 4.01 |  |  |
| Depression | 12 | 12 |  | 6 | 6 | 1.20 |  |  |
| **Neoplasms benign, malinant and unspecified(incl cysts and polyps)** | **0** | **0** | **0.00** | **1** | **1** | **0.20** | **/** | **0.33** |
| Lipoma | 0 | 0 | 0.00 | 1 | 1 | 0.20 |  |  |
| **Immune system disorders** | **4** | **4** | **0.40** | **1** | **1** | **0.20** | **2.0 (0.22, 18.05)** | **0.67** |
| Hypersensitivity | 4 | 4 | 0.40 | 1 | 1 | 0.20 |  |  |
| **Endocrine disorders** | **6** | **6** | **0.60** | **2** | **2** | **0.40** | **1.51 (0.30, 7.50)** | **0.73** |
| Hypothyroidism | 3 | 3 | 0.30 | 2 | 2 | 0.40 |  |  |
| Thyroid mass | 2 | 2 | 0.20 | 0 | 0 | 0.00 |  |  |
| **Skin and subcutaneous tissue disorders** | **25** | **22** | **2.21** | **6** | **6** | **1.20** | **1.86 (0.75, 4.62)** | **0.17** |
| Flushing | 4 | 4 | 0.40 | 1 | 1 | 0.20 |  |  |
| Dermatitis | 2 | 2 | 0.20 | 1 | 1 | 0.20 |  |  |
| Rash | 9 | 9 | 0.90 | 3 | 3 | 0.60 |  |  |
| Decubitus ulcer | 1 | 1 | 0.10 | 0 | 0 | 0.00 |  |  |
| Pruritus | 3 | 3 | 0.30 | 0 | 0 | 0.00 |  |  |
| **General disorders and administration site conditions**^#^ | **41** | **38** | **3.82** | **9** | **8** | **1.60** | **2.44 (1.13, 5.27)** | **0.02** |
| Pyrexia | 17 | 17 | 1.71 | 1 | 1 | 0.20 |  |  |
| Oedema peripheral | 5 | 5 | 0.50 | 0 | 0 | 0.00 |  |  |
| Hyperhidrosis | 4 | 4 | 0.40 | 2 | 2 | 0.40 |  |  |
| Asthenia | 4 | 4 | 0.40 | 1 | 1 | 0.20 |  |  |
| Chills | 3 | 3 | 0.30 | 1 | 1 | 0.20 |  |  |
| **Renal and urinary disorders** | **51** | **47** | **4.73** | **19** | **19** | **3.81** | **1.25 (0.73, 2.16)** | **0.41** |
| Urinary tract infection | 26 | 26 | 2.62 | 10 | 10 | 2.00 |  |  |
| Renal impairment | 4 | 4 | 0.40 | 3 | 3 | 0.60 |  |  |
| Proteinuria | 3 | 3 | 0.30 | 0 | 0 | 0.00 |  |  |
| Haematuria | 1 | 1 | 0.10 | 0 | 0 | 0.00 |  |  |
| **Reproductive system and breast disorders** | **16** | **16** | **1.61** | **9** | **7** | **1.40** | **1.15 (0.47, 2.81)** | **0.76** |
| Benign prostatic hyperplasia | 8 | 8 | 0.80 | 3 | 3 | 0.60 |  |  |
| Prostatomegaly | 5 | 5 | 0.50 | 1 | 1 | 0.20 |  |  |
| Vaginal haemorrhage | 2 | 2 | 0.20 | 0 | 0 | 0.00 |  |  |
| Heavy menstrual bleeding | 1 | 1 | 0.10 | 0 | 0 | 0.00 |  |  |
| Uterine haemorrhage | 0 | 0 | 0.00 | 1 | 1 | 0.20 |  |  |
| **Gastrointestinal disorders** | **192** | **158** | **15.90** | **77** | **69** | **13.83** | **1.18 (0.87, 1.60)** | **0.29** |
| Constipation | 99 | 96 | 9.66 | 50 | 48 | 9.62 |  |  |
| Diarrhoea | 14 | 14 | 1.41 | 2 | 2 | 0.40 |  |  |
| Abdominal discomfort | 8 | 8 | 0.80 | 5 | 5 | 1.00 |  |  |
| Vomiting | 8 | 7 | 0.70 | 0 | 0 | 0.00 |  |  |
| Gastrointestinal haemorrhage | 5 | 4 | 0.40 | 1 | 1 | 0.20 |  |  |
| Abdominal distension | 4 | 3 | 0.30 | 4 | 4 | 0.80 |  |  |
| Nausea | 4 | 4 | 0.40 | 1 | 1 | 0.20 |  |  |
| **Cardiac disorders** | **54** | **38** | **3.82** | **14** | **12** | **2.40** | **1.61 (0.84, 3.12)** | **0.15** |
| Coronary artery disease | 5 | 5 | 0.50 | 0 | 0 | 0.00 |  |  |
| Dyspnoea | 7 | 6 | 0.60 | 1 | 1 | 0.20 |  |  |
| Acute myocardial infarction | 0 | 0 | 0.00 | 1 | 1 | 0.20 |  |  |
| Palpitations | 7 | 7 | 0.70 | 2 | 2 | 0.40 |  |  |
| Chest discomfort | 22 | 20 | 2.01 | 5 | 5 | 1.00 |  |  |
| **Vascular disorders** | **30** | **28** | **2.82** | **20** | **19** | **3.81** | **0.73 (0.41, 1.33)** | **0.30** |
| Arteriosclerosis | 3 | 2 | 0.20 | 1 | 1 | 0.20 |  |  |
| Hypertension | 15 | 15 | 1.51 | 11 | 11 | 2.20 |  |  |
| **Blood and lymphatic system disorders** | **14** | **13** | **1.31** | **6** | **6** | **1.20** | **1.09 (0.41, 2.88)** | **0.86** |
| Leukaemia | 2 | 2 | 0.20 | 0 | 0 | 0.00 |  |  |
| Hyperfibrinogenaemia | 4 | 4 | 0.40 | 2 | 2 | 0.40 |  |  |
| Coagulopathy | 2 | 2 | 0.20 | 1 | 1 | 0.20 |  |  |
| Anaemia | 2 | 2 | 0.20 | 1 | 1 | 0.20 |  |  |
| Platelet disorder | 3 | 3 | 0.30 | 1 | 1 | 0.20 |  |  |
| **Eye disorders** | **20** | **20** | **2.01** | **20** | **16** | **3.21** | **0.62 (0.32, 1.21)** | **0.16** |
| Conjunctivitis | 3 | 3 | 0.30 | 3 | 3 | 0.60 |  |  |
| Cataract | 2 | 2 | 0.20 | 2 | 2 | 0.40 |  |  |
| Arteriosclerotic retinopathy | 1 | 1 | 0.10 | 2 | 2 | 0.40 |  |  |
| Dry eye | 5 | 5 | 0.50 | 0 | 0 | 0.00 |  |  |

^#^General disorders and administration-site conditions, including pyrexia, were more frequent in the DHI group (38 [3.82%] vs 8 [1.60%] patients; OR 2.44, 95% CI 1.13–5.27, p=0.02); these events were predominantly transient infusion-related reactions and did not translate into differences in serious adverse events (4.53% vs 5.21%; p=0.56) or mortality (see Table S9).

**Table S11 Clinical characteristics of patients who provided serum samples for RNA sequencing**

| **Characteristics** | **Total populations**  **(n=1505)** | **RNA sequencing populations**  **(n=39)** | ***DHI* group**  **(n = 24)** | **Control group**  **(n = 15)** |
| --- | --- | --- | --- | --- |
| **Age (years)** | 59(53,64) | 59 (53, 65) | 57 (52, 63) | 63 (54, 67) |
| **Sex (%)** | | | | |
| Men | 1054(70%) | 26 (67%) | 15 (62%) | 11 (73%) |
| Women | 451(30%) | 13 (33%) | 9 (38%) | 4 (27%) |
| **BMI (kg/m^2^)** | 23.88 (22.48, 25.78) | 24.62 (23.34, 25.66) | 24.53 (22.75, 25.63) | 24.74 (24.22, 25.59) |
| **Medical history (%)** | | | | |
| Hypertension | 984(65%) | 27(69%) | 17 (42%) | 10 (67%) |
| Diabetes | 339(23%) | 9(23%) | 5 (21%) | 4 (27%) |
| Past stroke / TIA | 107(7%) | 5 (13%) | 3 (13%) | 2 (13%) |
| **Clinical factors** | | | | |
| Right hemisphere stroke | 855(57%) | 19(49%) | 10 (42%) | 9 (60%) |
| Baseline NIHSS scores | 5(4,7) | 6(5,7) | 6(5,7) | 5 (4,7) |
| Baseline *Xueyu Zheng* *Score* ^#^ | 22(14,31) | 13(11,54) | 16 (12, 56) | 13 (11, 18) |
| Baseline EQ-5D Scale^##^ | 0.81 (0.56,0.84) | 0.83 (0.61, 0.84) | 0.82 (0.38, 0.84) | 0.84 (0.70, 0.84) |
| ASPECTS (core laboratory determined) | 9 (8,9) | 9 (8,9) | 9 (8,9) | 9 (8,9) |
| Time to randomization after onset of symptoms (hr) | 26(11,65) | 20 (8, 24) | 20 (10, 25) | 20 (6, 22) |

^✮^Data are presented as percentages or median (interquartiles). *DHI*: Danhong injection; BMI: body mass index; TIA: transient ischemic attack; NIHSS: National Institutes of Health Stroke Scale; ASPECTS: Alberta Stroke Program Early CT Score

^#^ *Xueyu Zheng* *Score* is obtained from the Efficacy Evaluation Scale of Chinese Medicine Symptom in *Xueyu Zheng,* evaluated on the severity of *Xueyu Zheng* using Visual Analogue Scale shown in Table S8; the more severe the symptom is, the higher the score be determined. If the symptom is disappeared, the score will be recorded as 0.

**^##^** EQ-5D Scale is a standardized instrument for the measurement of health status. Scores range from 0.1702 to 1.00 [6], with higher scores indicating a better quality of life.

**Table S12 The differentially expressed genes before and after treatment with *DHI* compared with control group**

| **Gene** | **Time_Diff** | **FoldChange**  **(*DHI*/Control)** | **log2FoldChange**  **(*DHI*/Control)** | **P-value** | **Up/Down-Regulation** | **Cor-δmRSDay90** | **Cor-P-Value** |
| --- | --- | --- | --- | --- | --- | --- | --- |
| JCHAIN | Day0-VS-Day14 | 1.669 | 0.739 | 0.014 | Up | 0.296 | 0.16 |
| EEF1B2 | Day0-VS-Day14 | 1.694 | 0.761 | 0.008 | Up | -0.079 | 0.714 |
| RPS6 | Day0-VS-Day14 | 1.581 | 0.660 | 0.011 | Up | 0.13 | 0.545 |
| TNFRSF17 | Day0-VS-Day14 | 1.620 | 0.696 | 0.034 | Up | 0.105 | 0.627 |
| BLVRB | Day0-VS-Day14 | 1.630 | 0.705 | 0.034 | Up | 0.429 | 0.036 |
| RPL17 | Day0-VS-Day14 | 1.737 | 0.797 | 0.010 | Up | -0.008 | 0.971 |
| RPS24 | Day0-VS-Day14 | 1.607 | 0.685 | 0.019 | Up | -0.059 | 0.785 |
| RPL23 | Day0-VS-Day14 | 1.662 | 0.733 | 0.014 | Up | -0.002 | 0.992 |
| RPS10 | Day0-VS-Day14 | 1.539 | 0.622 | 0.018 | Up | 0.214 | 0.316 |
| ATP5MPL | Day0-VS-Day14 | 1.556 | 0.638 | 0.025 | Up | -0.109 | 0.613 |
| RPS27 | Day0-VS-Day14 | 1.631 | 0.706 | 0.021 | Up | 0.163 | 0.447 |
| RPL34 | Day0-VS-Day14 | 1.648 | 0.721 | 0.044 | Up | -0.025 | 0.909 |
| RPL7 | Day0-VS-Day14 | 1.794 | 0.844 | 0.024 | Up | -0.077 | 0.722 |
| MRPL13 | Day0-VS-Day14 | 1.543 | 0.626 | 0.031 | Up | -0.266 | 0.209 |
| OLR1 | Day0-VS-Day14 | 2.047 | 1.033 | 0.045 | Up | -0.067 | 0.754 |
| COMMD6 | Day0-VS-Day14 | 1.572 | 0.653 | 0.034 | Up | 0.115 | 0.592 |
| RPL26 | Day0-VS-Day14 | 1.626 | 0.701 | 0.047 | Up | -0.081 | 0.708 |
| RPS15A | Day0-VS-Day14 | 1.666 | 0.736 | 0.014 | Up | 0.065 | 0.764 |
| GZMK | Day0-VS-Day14 | 1.719 | 0.782 | 0.020 | Up | -0.048 | 0.823 |
| RPS7 | Day0-VS-Day14 | 1.689 | 0.756 | 0.029 | Up | -0.014 | 0.949 |
| GUK1 | Day0-VS-Day14 | 1.608 | 0.685 | 0.045 | Up | 0.462 | 0.023 |
| H2AC7 | Day0-VS-Day14 | 0.132 | -2.918 | 0.029 | Down | 0.092 | 0.67 |
| H4C4 | Day0-VS-Day14 | 0.530 | -0.915 | 0.003 | Down | 0.178 | 0.405 |
| PALM | Day0-VS-Day90 | 1.987 | 0.990 | 0.006 | Up | -0.334 | 0.11 |
| SLC4A10 | Day0-VS-Day90 | 2.213 | 1.146 | 0.000 | Up | -0.201 | 0.346 |
| ENHO | Day0-VS-Day90 | 1.747 | 0.805 | 0.002 | Up | -0.251 | 0.236 |
| MMP1 | Day0-VS-Day90 | 0.257 | -1.962 | 0.000 | Down | 0.109 | 0.612 |
| THBS1 | Day0-VS-Day90 | 0.563 | -0.830 | 0.001 | Down | -0.636 | 0.001 |
| LOXHD1 | Day0-VS-Day90 | 0.588 | -0.766 | 0.002 | Down | -0.234 | 0.272 |
| TMEM158 | Day0-VS-Day90 | 0.502 | -0.994 | 0.004 | Down | -0.063 | 0.77 |
| MMP9 | Day0-VS-Day90 | 0.358 | -1.484 | 0.000 | Down | -0.225 | 0.291 |
| STAC | Day0-VS-Day90 | 0.160 | -2.641 | 0.000 | Down | -0.267 | 0.207 |
| ACHE | Day0-VS-Day90 | 0.387 | -1.370 | 0.001 | Down | 0.155 | 0.47 |
| SH3TC2 | Day0-VS-Day90 | 0.593 | -0.754 | 0.002 | Down | -0.652 | 0.001 |
| C19orf33 | Day0-VS-Day90 | 0.395 | -1.341 | 0.001 | Down | -0.01 | 0.963 |
| IGF2 | Day0-VS-Day90 | 0.454 | -1.140 | 0.003 | Down | 0.219 | 0.303 |
| CETP | Day0-VS-Day90 | 0.588 | -0.767 | 0.003 | Down | -0.102 | 0.637 |
| ESAM | Day0-VS-Day90 | 0.523 | -0.936 | 0.000 | Down | -0.168 | 0.434 |
| RBM38 | Day0-VS-Day90 | 0.433 | -1.207 | 0.000 | Down | 0.071 | 0.741 |
| ITGA2B | Day0-VS-Day90 | 0.486 | -1.041 | 0.006 | Down | -0.211 | 0.322 |
| SEMA6B | Day0-VS-Day90 | 0.461 | -1.118 | 0.001 | Down | -0.269 | 0.203 |
| PROS1 | Day0-VS-Day90 | 0.420 | -1.253 | 0.001 | Down | -0.22 | 0.301 |
| PEAR1 | Day0-VS-Day90 | 0.627 | -0.674 | 0.005 | Down | -0.29 | 0.169 |
| PCSK6 | Day0-VS-Day90 | 0.561 | -0.833 | 0.002 | Down | -0.325 | 0.121 |
| CLEC1B | Day0-VS-Day90 | 0.456 | -1.134 | 0.000 | Down | -0.134 | 0.533 |
| PTPRF | Day0-VS-Day90 | 0.476 | -1.070 | 0.003 | Down | 0.029 | 0.894 |
| FAXDC2 | Day0-VS-Day90 | 0.464 | -1.108 | 0.000 | Down | -0.169 | 0.43 |
| BEAN1 | Day0-VS-Day90 | 0.414 | -1.272 | 0.000 | Down | -0.259 | 0.222 |
| SLC24A3 | Day0-VS-Day90 | 0.520 | -0.944 | 0.000 | Down | -0.114 | 0.595 |
| TAL1 | Day0-VS-Day90 | 0.395 | -1.341 | 0.000 | Down | 0.111 | 0.607 |

**Table S13 The targeted modular network of 19 DEMs treated with *DHI***

| **DEM1** | **DEM2** | **SW** |
| --- | --- | --- |
| OGN | HBG2 | 0.98 |
| KRT6A | HBG2 | 0.044 |
| CHRNA9 | ZBED9 | 0.044 |
| TNFRSF19 | ZBED9 | 0.021 |
| TNFRSF19 | OGN | 0.024 |
| ZNF705B | DIRAS2 | 0.087 |
| C15orf65 | OGN | 0.138 |
| C15orf65 | KRT6A | 0.021 |
| C15orf65 | CHRNA9 | 0.062 |
| NPW | ZBED9 | 0.022 |
| NPW | NOTCH4 | 0.057 |
| NOTCH4 | DIRAS2 | 0.021 |
| NOTCH4 | CHRNA9 | 5.685 |
| S100P | KRT6A | 0.031 |
| S100P | HR | 1.015 |
| IL12B | NOTCH4 | 0.202 |
| TMEM108 | ZBED9 | 0.253 |
| TMEM108 | KRT6A | 0.05 |
| TMEM108 | S100P | 0.02 |
| STYK1 | DIRAS2 | 0.042 |
| STYK1 | OGN | 0.119 |
| STYK1 | CHRNA9 | 1.581 |
| STYK1 | TNFRSF19 | 0.949 |
| STYK1 | ZNF705B | 1.142 |
| STYK1 | C15orf65 | 0.551 |
| STYK1 | NOTCH4 | 0.314 |
| STYK1 | TMEM108 | 0.099 |
| KLHL4 | ZBED9 | 0.185 |
| KLHL4 | DIRAS2 | 0.128 |
| KLHL4 | OGN | 0.02 |
| KLHL4 | CHRNA9 | 0.043 |
| KLHL4 | S100P | 0.485 |
| KLHL4 | IL12B | 1.153 |
| KLHL4 | STYK1 | 0.166 |

**Table S14 The enriched GOs and KEGG pathways of the 19 DEMs treated with *DHI***

| **Module name** | **GO/KEGG ID** | **Description** | **Gene Ratio** | **BgRatio** | **P-value** | **GeneID** | **Count** |
| --- | --- | --- | --- | --- | --- | --- | --- |
| KLHL4 | GO:0010466 | negative regulation of peptidase activity | 6/50 | 262/18723 | 6.725E-05 | WFDC1/THBS1/MT3/NR4A1/ECM1/C3 | 6 |
| KLHL4 | GO:0045861 | negative regulation of proteolysis | 6/50 | 351/18723 | 3.297E-04 | WFDC1/THBS1/MT3/NR4A1/ECM1/C3 | 6 |
| KLHL4 | GO:0051346 | negative regulation of hydrolase activity | 6/50 | 379/18723 | 4.954E-04 | WFDC1/THBS1/MT3/NR4A1/ECM1/C3 | 6 |
| KLHL4 | GO:0050678 | regulation of epithelial cell proliferation | 6/50 | 381/18723 | 5.093E-04 | WFDC1/THBS1/NR4A1/ECM1/CDKN1C/CDH13 | 6 |
| KLHL4 | GO:0050673 | epithelial cell proliferation | 6/50 | 437/18723 | 1.041E-03 | WFDC1/THBS1/NR4A1/ECM1/CDKN1C/CDH13 | 6 |
| KLHL4 | GO:0052547 | regulation of peptidase activity | 6/50 | 461/18723 | 1.370E-03 | WFDC1/THBS1/MT3/NR4A1/ECM1/C3 | 6 |
| KLHL4 | GO:0007156 | homophilic cell adhesion via plasma membrane adhesion molecules | 5/50 | 167/18723 | 8.144E-05 | CADM3/PCDHB15/PCDHGA6/PCDHGB3/CDH13 | 5 |
| KLHL4 | GO:0010951 | negative regulation of endopeptidase activity | 5/50 | 252/18723 | 5.487E-04 | WFDC1/THBS1/MT3/NR4A1/C3 | 5 |
| KLHL4 | GO:0098742 | cell-cell adhesion via plasma-membrane adhesion molecules | 5/50 | 273/18723 | 7.876E-04 | CADM3/PCDHB15/PCDHGA6/PCDHGB3/CDH13 | 5 |
| KLHL4 | GO:0052548 | regulation of endopeptidase activity | 5/50 | 432/18723 | 0.006 | WFDC1/THBS1/MT3/NR4A1/C3 | 5 |
| KLHL4 | GO:0001936 | regulation of endothelial cell proliferation | 4/50 | 179/18723 | 1.320E-03 | THBS1/NR4A1/ECM1/CDH13 | 4 |
| KLHL4 | GO:0001935 | endothelial cell proliferation | 4/50 | 193/18723 | 0.002 | THBS1/NR4A1/ECM1/CDH13 | 4 |
| KLHL4 | GO:0010038 | response to metal ion | 4/50 | 373/18723 | 0.017 | SUCNR1/THBS1/PENK/MT3 | 4 |
| KLHL4 | GO:0032103 | positive regulation of response to external stimulus | 4/50 | 427/18723 | 0.027 | SUCNR1/THBS1/CDH13/C3 | 4 |
| KLHL4 | GO:0001667 | ameboidal-type cell migration | 4/50 | 475/18723 | 0.038 | AMOTL2/THBS1/NR4A1/CDH13 | 4 |
| KLHL4 | GO:0043154 | negative regulation of cysteine-type endopeptidase activity involved in apoptotic process | 3/50 | 78/18723 | 1.184E-03 | THBS1/MT3/NR4A1 | 3 |
| KLHL4 | GO:2000117 | negative regulation of cysteine-type endopeptidase activity | 3/50 | 86/18723 | 0.002 | THBS1/MT3/NR4A1 | 3 |
| KLHL4 | GO:0001938 | positive regulation of endothelial cell proliferation | 3/50 | 111/18723 | 0.003 | NR4A1/ECM1/CDH13 | 3 |
| KLHL4 | GO:0050921 | positive regulation of chemotaxis | 3/50 | 141/18723 | 0.006 | SUCNR1/THBS1/CDH13 | 3 |
| KLHL4 | GO:0051592 | response to calcium ion | 3/50 | 149/18723 | 0.007 | SUCNR1/THBS1/PENK | 3 |
| KLHL4 | GO:0050680 | negative regulation of epithelial cell proliferation | 3/50 | 164/18723 | 0.010 | WFDC1/THBS1/CDKN1C | 3 |
| KLHL4 | GO:0048469 | cell maturation | 3/50 | 171/18723 | 0.011 | SRRM4/ANGPTL8/C3 | 3 |
| KLHL4 | GO:0045766 | positive regulation of angiogenesis | 3/50 | 181/18723 | 0.012 | THBS1/ECM1/C3 | 3 |
| KLHL4 | GO:1904018 | positive regulation of vasculature development | 3/50 | 181/18723 | 0.012 | THBS1/ECM1/C3 | 3 |
| KLHL4 | GO:0002040 | sprouting angiogenesis | 3/50 | 185/18723 | 0.013 | THBS1/NR4A1/CDH13 | 3 |
| KLHL4 | GO:0050679 | positive regulation of epithelial cell proliferation | 3/50 | 207/18723 | 0.018 | NR4A1/ECM1/CDH13 | 3 |
| KLHL4 | GO:0043281 | regulation of cysteine-type endopeptidase activity involved in apoptotic process | 3/50 | 209/18723 | 0.018 | THBS1/MT3/NR4A1 | 3 |
| KLHL4 | GO:0050920 | regulation of chemotaxis | 3/50 | 223/18723 | 0.022 | SUCNR1/THBS1/CDH13 | 3 |
| KLHL4 | GO:0071695 | anatomical structure maturation | 3/50 | 229/18723 | 0.023 | SRRM4/ANGPTL8/C3 | 3 |
| KLHL4 | GO:2000116 | regulation of cysteine-type endopeptidase activity | 3/50 | 235/18723 | 0.025 | THBS1/MT3/NR4A1 | 3 |
| KLHL4 | GO:0071560 | cellular response to transforming growth factor beta stimulus | 3/50 | 250/18723 | 0.029 | THBS1/PENK/CDKN1C | 3 |
| KLHL4 | GO:0071559 | response to transforming growth factor beta | 3/50 | 256/18723 | 0.031 | THBS1/PENK/CDKN1C | 3 |
| KLHL4 | GO:0031349 | positive regulation of defense response | 3/50 | 278/18723 | 0.038 | SUCNR1/PENK/C3 | 3 |
| KLHL4 | GO:0043542 | endothelial cell migration | 3/50 | 279/18723 | 0.038 | THBS1/NR4A1/CDH13 | 3 |
| KLHL4 | GO:0021700 | developmental maturation | 3/50 | 280/18723 | 0.039 | SRRM4/ANGPTL8/C3 | 3 |
| KLHL4 | GO:0090287 | regulation of cellular response to growth factor stimulus | 3/50 | 304/18723 | 0.047 | THBS1/MT3/CDKN1C | 3 |
| KLHL4 | GO:0001666 | response to hypoxia | 3/50 | 307/18723 | 0.049 | THBS1/PENK/MT3 | 3 |
| KLHL4 | GO:0030511 | positive regulation of transforming growth factor beta receptor signaling pathway | 2/50 | 32/18723 | 0.003 | THBS1/CDKN1C | 2 |
| KLHL4 | GO:0035767 | endothelial cell chemotaxis | 2/50 | 32/18723 | 0.003 | THBS1/NR4A1 | 2 |
| KLHL4 | GO:1903846 | positive regulation of cellular response to transforming growth factor beta stimulus | 2/50 | 32/18723 | 0.003 | THBS1/CDKN1C | 2 |
| KLHL4 | GO:0042551 | neuron maturation | 2/50 | 45/18723 | 0.006 | SRRM4/C3 | 2 |
| KLHL4 | GO:0043277 | apoptotic cell clearance | 2/50 | 52/18723 | 0.009 | THBS1/C3 | 2 |
| KLHL4 | GO:0071320 | cellular response to cAMP | 2/50 | 53/18723 | 0.009 | PENK/AQP8 | 2 |
| KLHL4 | GO:0006749 | glutathione metabolic process | 2/50 | 64/18723 | 0.013 | GSTA4/GSTA2 | 2 |
| KLHL4 | GO:0070613 | regulation of protein processing | 2/50 | 65/18723 | 0.013 | ANGPTL8/THBS1 | 2 |
| KLHL4 | GO:1903317 | regulation of protein maturation | 2/50 | 67/18723 | 0.014 | ANGPTL8/THBS1 | 2 |
| KLHL4 | GO:0033555 | multicellular organismal response to stress | 2/50 | 71/18723 | 0.015 | THBS1/PENK | 2 |
| KLHL4 | GO:0061045 | negative regulation of wound healing | 2/50 | 78/18723 | 0.018 | WFDC1/THBS1 | 2 |
| KLHL4 | GO:0051591 | response to cAMP | 2/50 | 93/18723 | 0.026 | PENK/AQP8 | 2 |
| KLHL4 | GO:0002042 | cell migration involved in sprouting angiogenesis | 2/50 | 94/18723 | 0.026 | THBS1/NR4A1 | 2 |
| KLHL4 | GO:1903035 | negative regulation of response to wounding | 2/50 | 94/18723 | 0.026 | WFDC1/THBS1 | 2 |
| KLHL4 | GO:0048661 | positive regulation of smooth muscle cell proliferation | 2/50 | 104/18723 | 0.031 | THBS1/CDH13 | 2 |
| KLHL4 | GO:0042116 | macrophage activation | 2/50 | 106/18723 | 0.033 | SUCNR1/THBS1 | 2 |
| KLHL4 | GO:0006805 | xenobiotic metabolic process | 2/50 | 111/18723 | 0.035 | GSTA4/GSTA2 | 2 |
| KLHL4 | GO:0044344 | cellular response to fibroblast growth factor stimulus | 2/50 | 113/18723 | 0.037 | THBS1/NR4A1 | 2 |
| KLHL4 | GO:0090100 | positive regulation of transmembrane receptor protein serine/threonine kinase signaling pathway | 2/50 | 114/18723 | 0.037 | THBS1/CDKN1C | 2 |
| KLHL4 | GO:0071675 | regulation of mononuclear cell migration | 2/50 | 115/18723 | 0.038 | THBS1/ECM1 | 2 |
| KLHL4 | GO:0071774 | response to fibroblast growth factor | 2/50 | 120/18723 | 0.041 | THBS1/NR4A1 | 2 |
| KLHL4 | GO:0006911 | phagocytosis, engulfment | 2/50 | 127/18723 | 0.045 | THBS1/C3 | 2 |
| KLHL4 | GO:0001952 | regulation of cell-matrix adhesion | 2/50 | 128/18723 | 0.046 | THBS1/CDH13 | 2 |
| KLHL4 | GO:0017015 | regulation of transforming growth factor beta receptor signaling pathway | 2/50 | 128/18723 | 0.046 | THBS1/CDKN1C | 2 |
| KLHL4 | GO:0006334 | nucleosome assembly | 2/50 | 129/18723 | 0.047 | H4C11/H3-4 | 2 |
| KLHL4 | GO:0046683 | response to organophosphorus | 2/50 | 131/18723 | 0.048 | PENK/AQP8 | 2 |
| KLHL4 | GO:1903844 | regulation of cellular response to transforming growth factor beta stimulus | 2/50 | 131/18723 | 0.048 | THBS1/CDKN1C | 2 |
| KLHL4 | GO:0061041 | regulation of wound healing | 2/50 | 134/18723 | 0.049 | WFDC1/THBS1 | 2 |
| KLHL4 | hsa04010 | MAPK signaling pathway | 4/22 | 294/8142 | 0.007 | MRAS/PPP3R2/NR4A1/DUSP5 | 4 |
| KLHL4 | hsa05322 | Systemic lupus erythematosus | 3/22 | 136/8142 | 0.006 | H4C11/H3-4/C3 | 3 |
| KLHL4 | hsa04613 | Neutrophil extracellular trap formation | 3/22 | 190/8142 | 0.014 | H4C11/H3-4/C3 | 3 |
| KLHL4 | hsa00480 | Glutathione metabolism | 2/22 | 58/8142 | 0.011 | GSTA4/GSTA2 | 2 |
| KLHL4 | hsa05204 | Chemical carcinogenesis - DNA adducts | 2/22 | 69/8142 | 0.015 | GSTA4/GSTA2 | 2 |
| KLHL4 | hsa00982 | Drug metabolism - cytochrome P450 | 2/22 | 72/8142 | 0.016 | GSTA4/GSTA2 | 2 |
| KLHL4 | hsa01524 | Platinum drug resistance | 2/22 | 73/8142 | 0.016 | GSTA4/GSTA2 | 2 |
| KLHL4 | hsa00980 | Metabolism of xenobiotics by cytochrome P450 | 2/22 | 78/8142 | 0.018 | GSTA4/GSTA2 | 2 |
| KLHL4 | hsa00983 | Drug metabolism - other enzymes | 2/22 | 80/8142 | 0.019 | GSTA4/GSTA2 | 2 |
| KLHL4 | hsa04625 | C-type lectin receptor signaling pathway | 2/22 | 104/8142 | 0.032 | MRAS/PPP3R2 | 2 |
| STYK1 | GO:0035633 | maintenance of blood-brain barrier | 3/72 | 35/18723 | 3.267E-04 | WNK3/JAM2/GJB6 | 3 |
| STYK1 | GO:0045214 | sarcomere organization | 3/72 | 42/18723 | 5.621E-04 | OBSCN/KLHL41/MYH3 | 3 |
| STYK1 | GO:0055001 | muscle cell development | 5/72 | 184/18723 | 7.132E-04 | MYO18B/OBSCN/KLHL41/MYH3/LOX | 5 |
| STYK1 | GO:0030195 | negative regulation of blood coagulation | 3/72 | 49/18723 | 8.849E-04 | C1QTNF1/SERPINE1/KLKB1 | 3 |
| STYK1 | GO:1900047 | negative regulation of hemostasis | 3/72 | 50/18723 | 9.388E-04 | C1QTNF1/SERPINE1/KLKB1 | 3 |
| STYK1 | GO:0033629 | negative regulation of cell adhesion mediated by integrin | 2/72 | 12/18723 | 9.388E-04 | SERPINE1/MUC1 | 2 |
| STYK1 | GO:0050819 | negative regulation of coagulation | 3/72 | 53/18723 | 1.113E-03 | C1QTNF1/SERPINE1/KLKB1 | 3 |
| STYK1 | GO:0051917 | regulation of fibrinolysis | 2/72 | 14/18723 | 1.288E-03 | SERPINE1/KLKB1 | 2 |
| STYK1 | GO:0030199 | collagen fibril organization | 3/72 | 61/18723 | 0.002 | LOX/COL11A2/COL5A2 | 3 |
| STYK1 | GO:0030239 | myofibril assembly | 3/72 | 63/18723 | 0.002 | OBSCN/KLHL41/MYH3 | 3 |
| STYK1 | GO:0055002 | striated muscle cell development | 3/72 | 64/18723 | 0.002 | OBSCN/KLHL41/MYH3 | 3 |
| STYK1 | GO:0030193 | regulation of blood coagulation | 3/72 | 66/18723 | 0.002 | C1QTNF1/SERPINE1/KLKB1 | 3 |
| STYK1 | GO:1900046 | regulation of hemostasis | 3/72 | 68/18723 | 0.002 | C1QTNF1/SERPINE1/KLKB1 | 3 |
| STYK1 | GO:0050818 | regulation of coagulation | 3/72 | 71/18723 | 0.003 | C1QTNF1/SERPINE1/KLKB1 | 3 |
| STYK1 | GO:0010766 | negative regulation of sodium ion transport | 2/72 | 21/18723 | 0.003 | WNK3/DRD3 | 2 |
| STYK1 | GO:0061045 | negative regulation of wound healing | 3/72 | 78/18723 | 0.003 | C1QTNF1/SERPINE1/KLKB1 | 3 |
| STYK1 | GO:0042692 | muscle cell differentiation | 6/72 | 384/18723 | 0.004 | MYO18B/OBSCN/JAM2/KLHL41/MYH3/LOX | 6 |
| STYK1 | GO:0007286 | spermatid development | 4/72 | 165/18723 | 0.004 | CCDC42/JAM2/CABYR/CHD5 | 4 |
| STYK1 | GO:0031639 | plasminogen activation | 2/72 | 24/18723 | 0.004 | SERPINE1/KLKB1 | 2 |
| STYK1 | GO:0042730 | fibrinolysis | 2/72 | 25/18723 | 0.004 | SERPINE1/KLKB1 | 2 |
| STYK1 | GO:0048515 | spermatid differentiation | 4/72 | 172/18723 | 0.004 | CCDC42/JAM2/CABYR/CHD5 | 4 |
| STYK1 | GO:0051146 | striated muscle cell differentiation | 5/72 | 283/18723 | 0.005 | MYO18B/OBSCN/JAM2/KLHL41/MYH3 | 5 |
| STYK1 | GO:1903035 | negative regulation of response to wounding | 3/72 | 94/18723 | 0.006 | C1QTNF1/SERPINE1/KLKB1 | 3 |
| STYK1 | GO:0007162 | negative regulation of cell adhesion | 5/72 | 303/18723 | 0.006 | C1QTNF1/SERPINE1/MUC1/TACSTD2/IFNB1 | 5 |
| STYK1 | GO:0002323 | natural killer cell activation involved in immune response | 2/72 | 31/18723 | 0.006 | KLRF2/IFNB1 | 2 |
| STYK1 | GO:0051588 | regulation of neurotransmitter transport | 3/72 | 100/18723 | 0.007 | STX1A/DRD3/ASIC1 | 3 |
| STYK1 | GO:0031032 | actomyosin structure organization | 4/72 | 196/18723 | 0.007 | OBSCN/KLHL41/MYH3/TACSTD2 | 4 |
| STYK1 | GO:0010927 | cellular component assembly involved in morphogenesis | 3/72 | 107/18723 | 0.008 | OBSCN/KLHL41/MYH3 | 3 |
| STYK1 | GO:0023061 | signal release | 6/72 | 463/18723 | 0.009 | C1QTNF1/CAMK2N1/BRSK2/STX1A/DRD3/ASIC1 | 6 |
| STYK1 | GO:0042471 | ear morphogenesis | 3/72 | 115/18723 | 0.010 | OSR2/MYO3A/GJB6 | 3 |
| STYK1 | GO:0045777 | positive regulation of blood pressure | 2/72 | 40/18723 | 0.010 | CAMK2N1/GLP1R | 2 |
| STYK1 | GO:0042476 | odontogenesis | 3/72 | 130/18723 | 0.014 | OSR2/ENAM/SERPINE1 | 3 |
| STYK1 | GO:0033628 | regulation of cell adhesion mediated by integrin | 2/72 | 48/18723 | 0.015 | SERPINE1/MUC1 | 2 |
| STYK1 | GO:0042552 | myelination | 3/72 | 134/18723 | 0.015 | CMTM8/JAM2/PLP1 | 3 |
| STYK1 | GO:0061041 | regulation of wound healing | 3/72 | 134/18723 | 0.015 | C1QTNF1/SERPINE1/KLKB1 | 3 |
| STYK1 | GO:0070169 | positive regulation of biomineral tissue development | 2/72 | 49/18723 | 0.015 | OSR2/ENAM | 2 |
| STYK1 | GO:0046883 | regulation of hormone secretion | 4/72 | 249/18723 | 0.016 | C1QTNF1/CAMK2N1/BRSK2/STX1A | 4 |
| STYK1 | GO:0007272 | ensheathment of neurons | 3/72 | 136/18723 | 0.016 | CMTM8/JAM2/PLP1 | 3 |
| STYK1 | GO:0008366 | axon ensheathment | 3/72 | 136/18723 | 0.016 | CMTM8/JAM2/PLP1 | 3 |
| STYK1 | GO:0110151 | positive regulation of biomineralization | 2/72 | 50/18723 | 0.016 | OSR2/ENAM | 2 |
| STYK1 | GO:0014706 | striated muscle tissue development | 5/72 | 384/18723 | 0.016 | MYO18B/MYLPF/GJB6/KLHL41/LOX | 5 |
| STYK1 | GO:0061448 | connective tissue development | 4/72 | 252/18723 | 0.016 | OSR2/CHRDL2/LOX/COL11A2 | 4 |
| STYK1 | GO:0090596 | sensory organ morphogenesis | 4/72 | 256/18723 | 0.017 | OSR2/MYO3A/GJB6/COL5A2 | 4 |
| STYK1 | GO:0060537 | muscle tissue development | 5/72 | 403/18723 | 0.019 | MYO18B/MYLPF/GJB6/KLHL41/LOX | 5 |
| STYK1 | GO:0050433 | regulation of catecholamine secretion | 2/72 | 56/18723 | 0.020 | STX1A/DRD3 | 2 |
| STYK1 | GO:0050708 | regulation of protein secretion | 4/72 | 268/18723 | 0.020 | CAMK2N1/BRSK2/STX1A/DRD3 | 4 |
| STYK1 | GO:0050879 | multicellular organismal movement | 2/72 | 57/18723 | 0.020 | MYH3/DRD3 | 2 |
| STYK1 | GO:0050881 | musculoskeletal movement | 2/72 | 57/18723 | 0.020 | MYH3/DRD3 | 2 |
| STYK1 | GO:0071333 | cellular response to glucose stimulus | 3/72 | 151/18723 | 0.020 | CAMK2N1/GJB6/BRSK2 | 3 |
| STYK1 | GO:0050432 | catecholamine secretion | 2/72 | 58/18723 | 0.021 | STX1A/DRD3 | 2 |
| STYK1 | GO:2000649 | regulation of sodium ion transmembrane transporter activity | 2/72 | 58/18723 | 0.021 | WNK3/DRD3 | 2 |
| STYK1 | GO:0071331 | cellular response to hexose stimulus | 3/72 | 153/18723 | 0.021 | CAMK2N1/GJB6/BRSK2 | 3 |
| STYK1 | GO:0007605 | sensory perception of sound | 3/72 | 154/18723 | 0.022 | MYO3A/GJB6/COL11A2 | 3 |
| STYK1 | GO:0071326 | cellular response to monosaccharide stimulus | 3/72 | 154/18723 | 0.022 | CAMK2N1/GJB6/BRSK2 | 3 |
| STYK1 | GO:0007281 | germ cell development | 4/72 | 278/18723 | 0.022 | CCDC42/JAM2/CABYR/CHD5 | 4 |
| STYK1 | GO:0031638 | zymogen activation | 2/72 | 60/18723 | 0.022 | SERPINE1/KLKB1 | 2 |
| STYK1 | GO:0099072 | regulation of postsynaptic membrane neurotransmitter receptor levels | 2/72 | 62/18723 | 0.024 | CPT1C/DRD3 | 2 |
| STYK1 | GO:0043271 | negative regulation of ion transport | 3/72 | 160/18723 | 0.024 | WNK3/GABRE/DRD3 | 3 |
| STYK1 | GO:0071322 | cellular response to carbohydrate stimulus | 3/72 | 163/18723 | 0.025 | CAMK2N1/GJB6/BRSK2 | 3 |
| STYK1 | GO:0061178 | regulation of insulin secretion involved in cellular response to glucose stimulus | 2/72 | 64/18723 | 0.025 | CAMK2N1/BRSK2 | 2 |
| STYK1 | GO:0050796 | regulation of insulin secretion | 3/72 | 165/18723 | 0.026 | CAMK2N1/BRSK2/STX1A | 3 |
| STYK1 | GO:0070613 | regulation of protein processing | 2/72 | 65/18723 | 0.026 | SERPINE1/KLKB1 | 2 |
| STYK1 | GO:0071677 | positive regulation of mononuclear cell migration | 2/72 | 65/18723 | 0.026 | JAM2/SERPINE1 | 2 |
| STYK1 | GO:0034728 | nucleosome organization | 3/72 | 167/18723 | 0.027 | H2AB2/H2AB3/CHD5 | 3 |
| STYK1 | GO:1903034 | regulation of response to wounding | 3/72 | 167/18723 | 0.027 | C1QTNF1/SERPINE1/KLKB1 | 3 |
| STYK1 | GO:0046879 | hormone secretion | 4/72 | 295/18723 | 0.027 | C1QTNF1/CAMK2N1/BRSK2/STX1A | 4 |
| STYK1 | GO:0031214 | biomineral tissue development | 3/72 | 169/18723 | 0.027 | OSR2/ENAM/LOX | 3 |
| STYK1 | GO:0043967 | histone H4 acetylation | 2/72 | 67/18723 | 0.027 | CHD5/MUC1 | 2 |
| STYK1 | GO:1903317 | regulation of protein maturation | 2/72 | 67/18723 | 0.027 | SERPINE1/KLKB1 | 2 |
| STYK1 | GO:0110148 | biomineralization | 3/72 | 171/18723 | 0.028 | OSR2/ENAM/LOX | 3 |
| STYK1 | GO:0010812 | negative regulation of cell-substrate adhesion | 2/72 | 68/18723 | 0.028 | SERPINE1/TACSTD2 | 2 |
| STYK1 | GO:1902305 | regulation of sodium ion transmembrane transport | 2/72 | 68/18723 | 0.028 | WNK3/DRD3 | 2 |
| STYK1 | GO:0001678 | cellular glucose homeostasis | 3/72 | 172/18723 | 0.029 | CAMK2N1/GJB6/BRSK2 | 3 |
| STYK1 | GO:0050805 | negative regulation of synaptic transmission | 2/72 | 69/18723 | 0.029 | DRD3/ASIC1 | 2 |
| STYK1 | GO:0050954 | sensory perception of mechanical stimulus | 3/72 | 175/18723 | 0.030 | MYO3A/GJB6/COL11A2 | 3 |
| STYK1 | GO:0009914 | hormone transport | 4/72 | 306/18723 | 0.030 | C1QTNF1/CAMK2N1/BRSK2/STX1A | 4 |
| STYK1 | GO:0035725 | sodium ion transmembrane transport | 3/72 | 178/18723 | 0.031 | WNK3/DRD3/ASIC1 | 3 |
| STYK1 | GO:0033627 | cell adhesion mediated by integrin | 2/72 | 72/18723 | 0.031 | SERPINE1/MUC1 | 2 |
| STYK1 | GO:0051047 | positive regulation of secretion | 4/72 | 310/18723 | 0.032 | C1QTNF1/CAMK2N1/STX1A/DRD3 | 4 |
| STYK1 | GO:0035773 | insulin secretion involved in cellular response to glucose stimulus | 2/72 | 73/18723 | 0.032 | CAMK2N1/BRSK2 | 2 |
| STYK1 | GO:0051937 | catecholamine transport | 2/72 | 73/18723 | 0.032 | STX1A/DRD3 | 2 |
| STYK1 | GO:0060249 | anatomical structure homeostasis | 4/72 | 314/18723 | 0.033 | WNK3/JAM2/GJB6/LOX | 4 |
| STYK1 | GO:0008217 | regulation of blood pressure | 3/72 | 186/18723 | 0.035 | CAMK2N1/GLP1R/DRD3 | 3 |
| STYK1 | GO:0008306 | associative learning | 2/72 | 78/18723 | 0.036 | DRD3/ASIC1 | 2 |
| STYK1 | GO:0051216 | cartilage development | 3/72 | 190/18723 | 0.037 | OSR2/CHRDL2/COL11A2 | 3 |
| STYK1 | GO:0007517 | muscle organ development | 4/72 | 327/18723 | 0.037 | MYLPF/KLHL41/MYH3/LOX | 4 |
| STYK1 | GO:0030073 | insulin secretion | 3/72 | 195/18723 | 0.039 | CAMK2N1/BRSK2/STX1A | 3 |
| STYK1 | GO:0090276 | regulation of peptide hormone secretion | 3/72 | 196/18723 | 0.040 | CAMK2N1/BRSK2/STX1A | 3 |
| STYK1 | GO:0006323 | DNA packaging | 3/72 | 199/18723 | 0.041 | H2AB2/H2AB3/CHD5 | 3 |
| STYK1 | GO:0015844 | monoamine transport | 2/72 | 84/18723 | 0.042 | STX1A/DRD3 | 2 |
| STYK1 | GO:0002791 | regulation of peptide secretion | 3/72 | 200/18723 | 0.042 | CAMK2N1/BRSK2/STX1A | 3 |
| STYK1 | GO:0099003 | vesicle-mediated transport in synapse | 3/72 | 200/18723 | 0.042 | BRSK2/STX1A/DRD3 | 3 |
| STYK1 | GO:0060021 | roof of mouth development | 2/72 | 85/18723 | 0.042 | OSR2/COL11A2 | 2 |
| STYK1 | GO:0090087 | regulation of peptide transport | 3/72 | 202/18723 | 0.043 | CAMK2N1/BRSK2/STX1A | 3 |
| STYK1 | GO:0046928 | regulation of neurotransmitter secretion | 2/72 | 86/18723 | 0.043 | STX1A/ASIC1 | 2 |
| STYK1 | GO:0030101 | natural killer cell activation | 2/72 | 88/18723 | 0.045 | KLRF2/IFNB1 | 2 |
| STYK1 | GO:0006836 | neurotransmitter transport | 3/72 | 208/18723 | 0.046 | STX1A/DRD3/ASIC1 | 3 |
| STYK1 | GO:0002028 | regulation of sodium ion transport | 2/72 | 90/18723 | 0.047 | WNK3/DRD3 | 2 |
| STYK1 | GO:0042475 | odontogenesis of dentin-containing tooth | 2/72 | 90/18723 | 0.047 | ENAM/SERPINE1 | 2 |
| STYK1 | GO:0043491 | protein kinase B signaling | 3/72 | 211/18723 | 0.048 | C1QTNF1/LOX/DRD3 | 3 |
| STYK1 | GO:0002065 | columnar/cuboidal epithelial cell differentiation | 2/72 | 91/18723 | 0.048 | ENAM/SERPINE1 | 2 |
| STYK1 | GO:0051952 | regulation of amine transport | 2/72 | 91/18723 | 0.048 | STX1A/DRD3 | 2 |
| STYK1 | GO:2001233 | regulation of apoptotic signaling pathway | 4/72 | 356/18723 | 0.048 | SIAH2/SERPINE1/MUC1/IFNB1 | 4 |
| STYK1 | GO:0009749 | response to glucose | 3/72 | 212/18723 | 0.048 | CAMK2N1/GJB6/BRSK2 | 3 |
| STYK1 | GO:0001505 | regulation of neurotransmitter levels | 3/72 | 213/18723 | 0.049 | STX1A/DRD3/ASIC1 | 3 |
| STYK1 | GO:0043583 | ear development | 3/72 | 213/18723 | 0.049 | OSR2/MYO3A/GJB6 | 3 |
| STYK1 | GO:0009306 | protein secretion | 4/72 | 359/18723 | 0.049 | CAMK2N1/BRSK2/STX1A/DRD3 | 4 |
| STYK1 | GO:1901796 | regulation of signal transduction by p53 class mediator | 2/72 | 93/18723 | 0.049 | CHD5/MUC1 | 2 |
| STYK1 | GO:0035592 | establishment of protein localization to extracellular region | 4/72 | 360/18723 | 0.049 | CAMK2N1/BRSK2/STX1A/DRD3 | 4 |
| STYK1 | hsa04217 | Necroptosis | 4/25 | 159/8142 | 0.001 | H2AB2/H2AB3/H2AC20/IFNB1 | 4 |
| STYK1 | hsa05142 | Chagas disease | 3/25 | 102/8142 | 0.004 | PPP2R2C/SERPINE1/IFNB1 | 3 |
| STYK1 | hsa05322 | Systemic lupus erythematosus | 3/25 | 136/8142 | 0.008 | H2AB2/H2AB3/H2AC20 | 3 |
| STYK1 | hsa04390 | Hippo signaling pathway | 3/25 | 157/8142 | 0.012 | WNT8B/PPP2R2C/SERPINE1 | 3 |
| STYK1 | hsa05034 | Alcoholism | 3/25 | 187/8142 | 0.019 | H2AB2/H2AB3/H2AC20 | 3 |
| STYK1 | hsa04613 | Neutrophil extracellular trap formation | 3/25 | 190/8142 | 0.020 | H2AB2/H2AB3/H2AC20 | 3 |
| STYK1 | hsa04610 | Complement and coagulation cascades | 2/25 | 85/8142 | 0.028 | SERPINE1/KLKB1 | 2 |
| STYK1 | hsa04911 | Insulin secretion | 2/25 | 86/8142 | 0.028 | STX1A/GLP1R | 2 |
| STYK1 | hsa04974 | Protein digestion and absorption | 2/25 | 103/8142 | 0.039 | COL11A2/COL5A2 | 2 |
| STYK1 | hsa04670 | Leukocyte transendothelial migration | 2/25 | 114/8142 | 0.047 | JAM2/MYLPF | 2 |
| TMEM108 | GO:0045653 | negative regulation of megakaryocyte differentiation | 2/32 | 18/18723 | 4.257E-04 | H4C8/H4C4 | 2 |
| TMEM108 | GO:0006335 | DNA replication-dependent nucleosome assembly | 2/32 | 32/18723 | 1.359E-03 | H4C8/H4C4 | 2 |
| TMEM108 | GO:0034723 | DNA replication-dependent nucleosome organization | 2/32 | 32/18723 | 1.359E-03 | H4C8/H4C4 | 2 |
| TMEM108 | GO:0006334 | nucleosome assembly | 3/32 | 129/18723 | 1.369E-03 | H4C8/H4C4/H2BC9 | 3 |
| TMEM108 | GO:0006336 | DNA replication-independent nucleosome assembly | 2/32 | 33/18723 | 1.446E-03 | H4C8/H4C4 | 2 |
| TMEM108 | GO:0034724 | DNA replication-independent nucleosome organization | 2/32 | 34/18723 | 0.002 | H4C8/H4C4 | 2 |
| TMEM108 | GO:0045652 | regulation of megakaryocyte differentiation | 2/32 | 36/18723 | 0.002 | H4C8/H4C4 | 2 |
| TMEM108 | GO:0031497 | chromatin assembly | 3/32 | 151/18723 | 0.002 | H4C8/H4C4/H2BC9 | 3 |
| TMEM108 | GO:0034728 | nucleosome organization | 3/32 | 167/18723 | 0.003 | H4C8/H4C4/H2BC9 | 3 |
| TMEM108 | GO:0006333 | chromatin assembly or disassembly | 3/32 | 177/18723 | 0.003 | H4C8/H4C4/H2BC9 | 3 |
| TMEM108 | GO:0030219 | megakaryocyte differentiation | 2/32 | 57/18723 | 0.004 | H4C8/H4C4 | 2 |
| TMEM108 | GO:0006323 | DNA packaging | 3/32 | 199/18723 | 0.005 | H4C8/H4C4/H2BC9 | 3 |
| TMEM108 | GO:0065004 | protein-DNA complex assembly | 3/32 | 201/18723 | 0.005 | H4C8/H4C4/H2BC9 | 3 |
| TMEM108 | GO:0043627 | response to estrogen | 2/32 | 75/18723 | 0.007 | DHH/IGFBP2 | 2 |
| TMEM108 | GO:2000379 | positive regulation of reactive oxygen species metabolic process | 2/32 | 76/18723 | 0.007 | ACOD1/ADGRB1 | 2 |
| TMEM108 | GO:0071824 | protein-DNA complex subunit organization | 3/32 | 241/18723 | 0.008 | H4C8/H4C4/H2BC9 | 3 |
| TMEM108 | GO:0006338 | chromatin remodeling | 3/32 | 255/18723 | 0.009 | H4C8/H4C4/H2BC9 | 3 |
| TMEM108 | GO:0045638 | negative regulation of myeloid cell differentiation | 2/32 | 90/18723 | 0.010 | H4C8/H4C4 | 2 |
| TMEM108 | GO:0071103 | DNA conformation change | 3/32 | 290/18723 | 0.013 | H4C8/H4C4/H2BC9 | 3 |
| TMEM108 | GO:0006352 | DNA-templated transcription, initiation | 2/32 | 130/18723 | 0.021 | H4C8/H4C4 | 2 |
| TMEM108 | GO:0032355 | response to estradiol | 2/32 | 141/18723 | 0.024 | DHH/IGFBP2 | 2 |
| TMEM108 | GO:2000377 | regulation of reactive oxygen species metabolic process | 2/32 | 157/18723 | 0.029 | ACOD1/ADGRB1 | 2 |
| TMEM108 | GO:0032200 | telomere organization | 2/32 | 159/18723 | 0.030 | H4C8/H4C4 | 2 |
| TMEM108 | GO:0006325 | chromatin organization | 3/32 | 409/18723 | 0.032 | H4C8/H4C4/H2BC9 | 3 |
| TMEM108 | GO:0099173 | postsynapse organization | 2/32 | 168/18723 | 0.033 | TMEM108/NOS1AP | 2 |
| TMEM108 | GO:0050808 | synapse organization | 3/32 | 426/18723 | 0.036 | TMEM108/NOS1AP/ADGRB1 | 3 |
| TMEM108 | GO:0007565 | female pregnancy | 2/32 | 193/18723 | 0.043 | ACOD1/IGFBP2 | 2 |
| TMEM108 | GO:0045637 | regulation of myeloid cell differentiation | 2/32 | 210/18723 | 0.0498 | H4C8/H4C4 | 2 |
| TMEM108 | hsa05322 | Systemic lupus erythematosus | 4/14 | 136/8142 | 6.547E-05 | H2AC11/H4C8/H4C4/H2BC9 | 4 |
| TMEM108 | hsa05034 | Alcoholism | 4/14 | 187/8142 | 2.252E-04 | H2AC11/H4C8/H4C4/H2BC9 | 4 |
| TMEM108 | hsa04613 | Neutrophil extracellular trap formation | 4/14 | 190/8142 | 2.394E-04 | H2AC11/H4C8/H4C4/H2BC9 | 4 |
| TMEM108 | hsa05203 | Viral carcinogenesis | 3/14 | 204/8142 | 4.600E-03 | H4C8/H4C4/H2BC9 | 3 |
| NPW | GO:0060044 | negative regulation of cardiac muscle cell proliferation | 2/19 | 20/18723 | 1.834E-04 | RBP4/KCNK2 | 2 |
| NPW | GO:0055022 | negative regulation of cardiac muscle tissue growth | 2/19 | 31/18723 | 4.458E-04 | RBP4/KCNK2 | 2 |
| NPW | GO:0061117 | negative regulation of heart growth | 2/19 | 31/18723 | 4.458E-04 | RBP4/KCNK2 | 2 |
| NPW | GO:0046621 | negative regulation of organ growth | 2/19 | 39/18723 | 7.070E-04 | RBP4/KCNK2 | 2 |
| NPW | GO:0060043 | regulation of cardiac muscle cell proliferation | 2/19 | 51/18723 | 1.208E-03 | RBP4/KCNK2 | 2 |
| NPW | GO:0071805 | potassium ion transmembrane transport | 3/19 | 219/18723 | 1.332E-03 | KCNK2/KCNJ10/EDN3 | 3 |
| NPW | GO:0051353 | positive regulation of oxidoreductase activity | 2/19 | 59/18723 | 0.002 | EDN3/CYP27B1 | 2 |
| NPW | GO:0060038 | cardiac muscle cell proliferation | 2/19 | 60/18723 | 0.002 | RBP4/KCNK2 | 2 |
| NPW | GO:0006813 | potassium ion transport | 3/19 | 241/18723 | 0.002 | KCNK2/KCNJ10/EDN3 | 3 |
| NPW | GO:0045926 | negative regulation of growth | 3/19 | 249/18723 | 0.002 | RBP4/KCNK2/CYP27B1 | 3 |
| NPW | GO:0055021 | regulation of cardiac muscle tissue growth | 2/19 | 72/18723 | 0.002 | RBP4/KCNK2 | 2 |
| NPW | GO:0014855 | striated muscle cell proliferation | 2/19 | 76/18723 | 0.003 | RBP4/KCNK2 | 2 |
| NPW | GO:0060420 | regulation of heart growth | 2/19 | 79/18723 | 0.003 | RBP4/KCNK2 | 2 |
| NPW | GO:0014032 | neural crest cell development | 2/19 | 81/18723 | 0.003 | EDN3/CDH2 | 2 |
| NPW | GO:0014031 | mesenchymal cell development | 2/19 | 86/18723 | 0.003 | EDN3/CDH2 | 2 |
| NPW | GO:0048864 | stem cell development | 2/19 | 86/18723 | 0.003 | EDN3/CDH2 | 2 |
| NPW | GO:0014033 | neural crest cell differentiation | 2/19 | 91/18723 | 0.004 | EDN3/CDH2 | 2 |
| NPW | GO:0055017 | cardiac muscle tissue growth | 2/19 | 96/18723 | 0.004 | RBP4/KCNK2 | 2 |
| NPW | GO:0060419 | heart growth | 2/19 | 104/18723 | 0.005 | RBP4/KCNK2 | 2 |
| NPW | GO:0046620 | regulation of organ growth | 2/19 | 106/18723 | 0.005 | RBP4/KCNK2 | 2 |
| NPW | GO:0051341 | regulation of oxidoreductase activity | 2/19 | 107/18723 | 0.005 | EDN3/CYP27B1 | 2 |
| NPW | GO:0048640 | negative regulation of developmental growth | 2/19 | 112/18723 | 0.006 | RBP4/KCNK2 | 2 |
| NPW | GO:0046887 | positive regulation of hormone secretion | 2/19 | 133/18723 | 0.008 | RBP4/EDN3 | 2 |
| NPW | GO:0010977 | negative regulation of neuron projection development | 2/19 | 137/18723 | 0.008 | NEU4/KANK1 | 2 |
| NPW | GO:0010975 | regulation of neuron projection development | 3/19 | 445/18723 | 0.010 | NEU4/KANK1/CDH2 | 3 |
| NPW | GO:0003205 | cardiac chamber development | 2/19 | 161/18723 | 0.011 | RBP4/KCNK2 | 2 |
| NPW | GO:0001667 | ameboidal-type cell migration | 3/19 | 475/18723 | 0.012 | KANK1/EDN3/CDH2 | 3 |
| NPW | GO:0006694 | steroid biosynthetic process | 2/19 | 173/18723 | 0.013 | CYB5R2/CYP27B1 | 2 |
| NPW | GO:0035265 | organ growth | 2/19 | 178/18723 | 0.014 | RBP4/KCNK2 | 2 |
| NPW | GO:0031345 | negative regulation of cell projection organization | 2/19 | 186/18723 | 0.015 | NEU4/KANK1 | 2 |
| NPW | GO:0048863 | stem cell differentiation | 2/19 | 206/18723 | 0.018 | EDN3/CDH2 | 2 |
| NPW | GO:0007601 | visual perception | 2/19 | 215/18723 | 0.020 | RBP4/KCNJ10 | 2 |
| NPW | GO:0009612 | response to mechanical stimulus | 2/19 | 216/18723 | 0.020 | KCNK2/CDH2 | 2 |
| NPW | GO:0042445 | hormone metabolic process | 2/19 | 218/18723 | 0.020 | RBP4/CYP27B1 | 2 |
| NPW | GO:0050953 | sensory perception of light stimulus | 2/19 | 219/18723 | 0.020 | RBP4/KCNJ10 | 2 |
| NPW | GO:0010001 | glial cell differentiation | 2/19 | 225/18723 | 0.021 | KCNJ10/CDH2 | 2 |
| NPW | GO:0030072 | peptide hormone secretion | 2/19 | 236/18723 | 0.023 | RBP4/EDN3 | 2 |
| NPW | GO:0048738 | cardiac muscle tissue development | 2/19 | 236/18723 | 0.023 | RBP4/KCNK2 | 2 |
| NPW | GO:0048762 | mesenchymal cell differentiation | 2/19 | 236/18723 | 0.023 | EDN3/CDH2 | 2 |
| NPW | GO:1901617 | organic hydroxy compound biosynthetic process | 2/19 | 237/18723 | 0.024 | CYB5R2/CYP27B1 | 2 |
| NPW | GO:0002790 | peptide secretion | 2/19 | 242/18723 | 0.025 | RBP4/EDN3 | 2 |
| NPW | GO:0033002 | muscle cell proliferation | 2/19 | 248/18723 | 0.026 | RBP4/KCNK2 | 2 |
| NPW | GO:0046883 | regulation of hormone secretion | 2/19 | 249/18723 | 0.026 | RBP4/EDN3 | 2 |
| NPW | GO:0060828 | regulation of canonical Wnt signaling pathway | 2/19 | 253/18723 | 0.027 | KANK1/CDH2 | 2 |
| NPW | GO:0015833 | peptide transport | 2/19 | 264/18723 | 0.029 | RBP4/EDN3 | 2 |
| NPW | GO:0030522 | intracellular receptor signaling pathway | 2/19 | 265/18723 | 0.029 | TRIM15/CYP27B1 | 2 |
| NPW | GO:1903532 | positive regulation of secretion by cell | 2/19 | 282/18723 | 0.033 | RBP4/EDN3 | 2 |
| NPW | GO:0060485 | mesenchyme development | 2/19 | 291/18723 | 0.035 | EDN3/CDH2 | 2 |
| NPW | GO:0046879 | hormone secretion | 2/19 | 295/18723 | 0.035 | RBP4/EDN3 | 2 |
| NPW | GO:0050890 | cognition | 2/19 | 296/18723 | 0.036 | TMPRSS11E/KCNK2 | 2 |
| NPW | GO:0042063 | gliogenesis | 2/19 | 301/18723 | 0.037 | KCNJ10/CDH2 | 2 |
| NPW | GO:0042886 | amide transport | 2/19 | 301/18723 | 0.037 | RBP4/EDN3 | 2 |
| NPW | GO:0060070 | canonical Wnt signaling pathway | 2/19 | 303/18723 | 0.037 | KANK1/CDH2 | 2 |
| NPW | GO:0009914 | hormone transport | 2/19 | 306/18723 | 0.038 | RBP4/EDN3 | 2 |
| NPW | GO:0051047 | positive regulation of secretion | 2/19 | 310/18723 | 0.039 | RBP4/EDN3 | 2 |
| NPW | GO:0008202 | steroid metabolic process | 2/19 | 319/18723 | 0.041 | CYB5R2/CYP27B1 | 2 |
| NPW | GO:0016042 | lipid catabolic process | 2/19 | 320/18723 | 0.041 | NEU4/CYP27B1 | 2 |
| NPW | GO:0002831 | regulation of response to biotic stimulus | 2/19 | 327/18723 | 0.043 | TRIM15/SUSD4 | 2 |
| NPW | GO:0030111 | regulation of Wnt signaling pathway | 2/19 | 328/18723 | 0.043 | KANK1/CDH2 | 2 |
| NPW | GO:0048638 | regulation of developmental growth | 2/19 | 330/18723 | 0.043 | RBP4/KCNK2 | 2 |
| NPW | GO:0002697 | regulation of immune effector process | 2/19 | 339/18723 | 0.046 | SUSD4/RBP4 | 2 |
| NPW | GO:0006066 | alcohol metabolic process | 2/19 | 353/18723 | 0.049 | RBP4/CYP27B1 | 2 |
| NPW | GO:0015711 | organic anion transport | 2/19 | 354/18723 | 0.049 | CYB5R2/KCNJ10 | 2 |
| NPW | hsa04971 | Gastric acid secretion | 2/10 | 76/8142 | 0.004 | KCNK2/KCNJ10 | 2 |
| S100P | GO:0051592 | response to calcium ion | 2/25 | 149/18723 | 0.017 | SYT3/KCNMA1 | 2 |
| S100P | GO:0016052 | carbohydrate catabolic process | 2/25 | 154/18723 | 0.018 | MLXIPL/FUT5 | 2 |
| S100P | GO:0023061 | signal release | 3/25 | 463/18723 | 0.023 | ILDR2/SYT3/FAM3B | 3 |
| S100P | GO:0071897 | DNA biosynthetic process | 2/25 | 180/18723 | 0.024 | DSCC1/TEX12 | 2 |
| S100P | GO:0030073 | insulin secretion | 2/25 | 195/18723 | 0.028 | ILDR2/FAM3B | 2 |
| S100P | GO:0009749 | response to glucose | 2/25 | 212/18723 | 0.032 | ILDR2/MLXIPL | 2 |
| S100P | GO:0009746 | response to hexose | 2/25 | 219/18723 | 0.034 | ILDR2/MLXIPL | 2 |
| S100P | GO:0034284 | response to monosaccharide | 2/25 | 225/18723 | 0.036 | ILDR2/MLXIPL | 2 |
| S100P | GO:0030072 | peptide hormone secretion | 2/25 | 236/18723 | 0.039 | ILDR2/FAM3B | 2 |
| S100P | GO:0002790 | peptide secretion | 2/25 | 242/18723 | 0.041 | ILDR2/FAM3B | 2 |
| S100P | GO:0090257 | regulation of muscle system process | 2/25 | 252/18723 | 0.044 | SGCA/KCNMA1 | 2 |
| S100P | GO:0009743 | response to carbohydrate | 2/25 | 253/18723 | 0.044 | ILDR2/MLXIPL | 2 |
| S100P | GO:0042593 | glucose homeostasis | 2/25 | 258/18723 | 0.046 | FAM3B/MLXIPL | 2 |
| S100P | GO:0033500 | carbohydrate homeostasis | 2/25 | 259/18723 | 0.046 | FAM3B/MLXIPL | 2 |
| S100P | GO:0015833 | peptide transport | 2/25 | 264/18723 | 0.048 | ILDR2/FAM3B | 2 |
| IL26 | GO:0046427 | positive regulation of receptor signaling pathway via JAK-STAT | 2/5 | 44/18723 | 5.373E-05 | IL26/IL4 | 2 |
| IL26 | GO:1904894 | positive regulation of receptor signaling pathway via STAT | 2/5 | 49/18723 | 6.676E-05 | IL26/IL4 | 2 |
| IL26 | GO:0046425 | regulation of receptor signaling pathway via JAK-STAT | 2/5 | 103/18723 | 2.965E-04 | IL26/IL4 | 2 |
| IL26 | GO:1904892 | regulation of receptor signaling pathway via STAT | 2/5 | 114/18723 | 3.631E-04 | IL26/IL4 | 2 |
| IL26 | GO:0007259 | receptor signaling pathway via JAK-STAT | 2/5 | 170/18723 | 8.050E-04 | IL26/IL4 | 2 |
| IL26 | GO:0097696 | receptor signaling pathway via STAT | 2/5 | 181/18723 | 9.118E-04 | IL26/IL4 | 2 |
| IL26 | GO:0010632 | regulation of epithelial cell migration | 2/5 | 292/18723 | 0.002 | JCAD/IL4 | 2 |
| IL26 | GO:0090287 | regulation of cellular response to growth factor stimulus | 2/5 | 304/18723 | 0.003 | JCAD/IL4 | 2 |
| IL26 | GO:0010631 | epithelial cell migration | 2/5 | 357/18723 | 0.003 | JCAD/IL4 | 2 |
| IL26 | GO:0090132 | epithelium migration | 2/5 | 360/18723 | 0.004 | JCAD/IL4 | 2 |
| IL26 | GO:0090130 | tissue migration | 2/5 | 365/18723 | 0.004 | JCAD/IL4 | 2 |
| IL26 | GO:0050678 | regulation of epithelial cell proliferation | 2/5 | 381/18723 | 0.004 | JCAD/IL26 | 2 |
| IL26 | GO:0050673 | epithelial cell proliferation | 2/5 | 437/18723 | 0.005 | JCAD/IL26 | 2 |
| IL26 | GO:0001819 | positive regulation of cytokine production | 2/5 | 467/18723 | 0.006 | IL26/IL4 | 2 |
| IL26 | GO:0001667 | ameboidal-type cell migration | 2/5 | 475/18723 | 0.006 | JCAD/IL4 | 2 |
| IL26 | GO:0043410 | positive regulation of MAPK cascade | 2/5 | 480/18723 | 0.006 | JCAD/IL26 | 2 |
| IL26 | hsa04060 | Cytokine-cytokine receptor interaction | 2/3 | 295/8142 | 0.004 | IL26/IL4 | 2 |
| NOTCH4 | GO:0042634 | regulation of hair cycle | 2/26 | 26/18723 | 5.904E-04 | TRPV3/PER1 | 2 |
| NOTCH4 | GO:0031641 | regulation of myelination | 2/26 | 47/18723 | 0.002 | TNFRSF21/SOX10 | 2 |
| NOTCH4 | GO:0061138 | morphogenesis of a branching epithelium | 3/26 | 182/18723 | 0.002 | DCHS1/SOX10/NOTCH4 | 3 |
| NOTCH4 | GO:0045806 | negative regulation of endocytosis | 2/26 | 53/18723 | 0.002 | SCAMP5/PACSIN1 | 2 |
| NOTCH4 | GO:0001763 | morphogenesis of a branching structure | 3/26 | 196/18723 | 0.002 | DCHS1/SOX10/NOTCH4 | 3 |
| NOTCH4 | GO:0022408 | negative regulation of cell-cell adhesion | 3/26 | 196/18723 | 0.002 | TNFRSF21/SOCS1/NOTCH4 | 3 |
| NOTCH4 | GO:0001885 | endothelial cell development | 2/26 | 64/18723 | 0.004 | PTPRS/NOTCH4 | 2 |
| NOTCH4 | GO:0042509 | regulation of tyrosine phosphorylation of STAT protein | 2/26 | 85/18723 | 0.006 | SOCS1/CTF1 | 2 |
| NOTCH4 | GO:0007260 | tyrosine phosphorylation of STAT protein | 2/26 | 88/18723 | 0.007 | SOCS1/CTF1 | 2 |
| NOTCH4 | GO:0060485 | mesenchyme development | 3/26 | 291/18723 | 0.007 | DCHS1/SOX10/NOTCH4 | 3 |
| NOTCH4 | GO:0048709 | oligodendrocyte differentiation | 2/26 | 95/18723 | 0.008 | TNFRSF21/SOX10 | 2 |
| NOTCH4 | GO:0007162 | negative regulation of cell adhesion | 3/26 | 303/18723 | 0.008 | TNFRSF21/SOCS1/NOTCH4 | 3 |
| NOTCH4 | GO:0042303 | molting cycle | 2/26 | 107/18723 | 0.010 | TRPV3/PER1 | 2 |
| NOTCH4 | GO:0042633 | hair cycle | 2/26 | 107/18723 | 0.010 | TRPV3/PER1 | 2 |
| NOTCH4 | GO:0045446 | endothelial cell differentiation | 2/26 | 118/18723 | 0.012 | PTPRS/NOTCH4 | 2 |
| NOTCH4 | GO:0050868 | negative regulation of T cell activation | 2/26 | 122/18723 | 0.012 | TNFRSF21/SOCS1 | 2 |
| NOTCH4 | GO:0048565 | digestive tract development | 2/26 | 127/18723 | 0.013 | DCHS1/SOX10 | 2 |
| NOTCH4 | GO:0042552 | myelination | 2/26 | 134/18723 | 0.015 | TNFRSF21/SOX10 | 2 |
| NOTCH4 | GO:0003158 | endothelium development | 2/26 | 136/18723 | 0.015 | PTPRS/NOTCH4 | 2 |
| NOTCH4 | GO:0007272 | ensheathment of neurons | 2/26 | 136/18723 | 0.015 | TNFRSF21/SOX10 | 2 |
| NOTCH4 | GO:0008366 | axon ensheathment | 2/26 | 136/18723 | 0.015 | TNFRSF21/SOX10 | 2 |
| NOTCH4 | GO:0055123 | digestive system development | 2/26 | 137/18723 | 0.015 | DCHS1/SOX10 | 2 |
| NOTCH4 | GO:0050768 | negative regulation of neurogenesis | 2/26 | 140/18723 | 0.016 | SOX10/PTPRS | 2 |
| NOTCH4 | GO:1903038 | negative regulation of leukocyte cell-cell adhesion | 2/26 | 141/18723 | 0.016 | TNFRSF21/SOCS1 | 2 |
| NOTCH4 | GO:0031644 | regulation of nervous system process | 2/26 | 144/18723 | 0.017 | TNFRSF21/SOX10 | 2 |
| NOTCH4 | GO:0051961 | negative regulation of nervous system development | 2/26 | 145/18723 | 0.017 | SOX10/PTPRS | 2 |
| NOTCH4 | GO:0010959 | regulation of metal ion transport | 3/26 | 406/18723 | 0.018 | LRRC26/TRPV3/PER1 | 3 |
| NOTCH4 | GO:0048754 | branching morphogenesis of an epithelial tube | 2/26 | 151/18723 | 0.019 | DCHS1/NOTCH4 | 2 |
| NOTCH4 | GO:0051250 | negative regulation of lymphocyte activation | 2/26 | 157/18723 | 0.020 | TNFRSF21/SOCS1 | 2 |
| NOTCH4 | GO:0051960 | regulation of nervous system development | 3/26 | 443/18723 | 0.023 | TNFRSF21/SOX10/PTPRS | 3 |
| NOTCH4 | GO:0007259 | receptor signaling pathway via JAK-STAT | 2/26 | 170/18723 | 0.023 | SOCS1/CTF1 | 2 |
| NOTCH4 | GO:0022407 | regulation of cell-cell adhesion | 3/26 | 448/18723 | 0.024 | TNFRSF21/SOCS1/NOTCH4 | 3 |
| NOTCH4 | GO:0071466 | cellular response to xenobiotic stimulus | 2/26 | 177/18723 | 0.025 | UGT2B11/SOX10 | 2 |
| NOTCH4 | GO:0010721 | negative regulation of cell development | 2/26 | 180/18723 | 0.026 | SOX10/PTPRS | 2 |
| NOTCH4 | GO:0097696 | receptor signaling pathway via STAT | 2/26 | 181/18723 | 0.026 | SOCS1/CTF1 | 2 |
| NOTCH4 | GO:0061564 | axon development | 3/26 | 467/18723 | 0.026 | TNFRSF21/PLPPR4/PTPRS | 3 |
| NOTCH4 | GO:0002695 | negative regulation of leukocyte activation | 2/26 | 187/18723 | 0.028 | TNFRSF21/SOCS1 | 2 |
| NOTCH4 | GO:0048839 | inner ear development | 2/26 | 188/18723 | 0.028 | PLPPR4/DCHS1 | 2 |
| NOTCH4 | GO:0032872 | regulation of stress-activated MAPK cascade | 2/26 | 192/18723 | 0.029 | PER1/MID1 | 2 |
| NOTCH4 | GO:0070302 | regulation of stress-activated protein kinase signaling cascade | 2/26 | 195/18723 | 0.030 | PER1/MID1 | 2 |
| NOTCH4 | GO:0071383 | cellular response to steroid hormone stimulus | 2/26 | 204/18723 | 0.032 | SOX10/PER1 | 2 |
| NOTCH4 | GO:0060348 | bone development | 2/26 | 205/18723 | 0.033 | DCHS1/PHEX | 2 |
| NOTCH4 | GO:0050866 | negative regulation of cell activation | 2/26 | 210/18723 | 0.034 | TNFRSF21/SOCS1 | 2 |
| NOTCH4 | GO:0030100 | regulation of endocytosis | 2/26 | 211/18723 | 0.034 | SCAMP5/PACSIN1 | 2 |
| NOTCH4 | GO:0043583 | ear development | 2/26 | 213/18723 | 0.035 | PLPPR4/DCHS1 | 2 |
| NOTCH4 | GO:0002064 | epithelial cell development | 2/26 | 220/18723 | 0.037 | PTPRS/NOTCH4 | 2 |
| NOTCH4 | GO:0010001 | glial cell differentiation | 2/26 | 225/18723 | 0.039 | TNFRSF21/SOX10 | 2 |
| NOTCH4 | GO:0016485 | protein processing | 2/26 | 225/18723 | 0.039 | PRSS12/PHEX | 2 |
| NOTCH4 | GO:0071695 | anatomical structure maturation | 2/26 | 229/18723 | 0.040 | DCHS1/SOX10 | 2 |
| NOTCH4 | GO:0048762 | mesenchymal cell differentiation | 2/26 | 236/18723 | 0.042 | SOX10/NOTCH4 | 2 |
| NOTCH4 | GO:0051403 | stress-activated MAPK cascade | 2/26 | 239/18723 | 0.043 | PER1/MID1 | 2 |
| NOTCH4 | GO:0016358 | dendrite development | 2/26 | 243/18723 | 0.044 | PACSIN1/PTPRS | 2 |
| NOTCH4 | GO:0006814 | sodium ion transport | 2/26 | 245/18723 | 0.045 | SLC9B1/PER1 | 2 |
| NOTCH4 | GO:0031098 | stress-activated protein kinase signaling cascade | 2/26 | 247/18723 | 0.046 | PER1/MID1 | 2 |
| NOTCH4 | hsa04120 | Ubiquitin mediated proteolysis | 2/11 | 142/8142 | 0.015 | SOCS1/MID1 | 2 |
| NOTCH4 | hsa04630 | JAK-STAT signaling pathway | 2/11 | 162/8142 | 0.019 | SOCS1/CTF1 | 2 |
| C15orf65 | GO:0042472 | inner ear morphogenesis | 3/22 | 96/18723 | 1.874E-04 | CLRN1/POU4F3/GLI2 | 3 |
| C15orf65 | GO:0042471 | ear morphogenesis | 3/22 | 115/18723 | 3.192E-04 | CLRN1/POU4F3/GLI2 | 3 |
| C15orf65 | GO:0008544 | epidermis development | 4/22 | 324/18723 | 5.033E-04 | CLRN1/POU4F3/GLI2/DNASE1L2 | 4 |
| C15orf65 | GO:0042491 | inner ear auditory receptor cell differentiation | 2/22 | 38/18723 | 9.031E-04 | CLRN1/POU4F3 | 2 |
| C15orf65 | GO:0048839 | inner ear development | 3/22 | 188/18723 | 1.333E-03 | CLRN1/POU4F3/GLI2 | 3 |
| C15orf65 | GO:0035315 | hair cell differentiation | 2/22 | 47/18723 | 1.380E-03 | CLRN1/POU4F3 | 2 |
| C15orf65 | GO:0050885 | neuromuscular process controlling balance | 2/22 | 47/18723 | 1.380E-03 | CLRN1/POU4F3 | 2 |
| C15orf65 | GO:0009913 | epidermal cell differentiation | 3/22 | 202/18723 | 0.002 | CLRN1/POU4F3/DNASE1L2 | 3 |
| C15orf65 | GO:0043583 | ear development | 3/22 | 213/18723 | 0.002 | CLRN1/POU4F3/GLI2 | 3 |
| C15orf65 | GO:0060113 | inner ear receptor cell differentiation | 2/22 | 62/18723 | 0.002 | CLRN1/POU4F3 | 2 |
| C15orf65 | GO:0042490 | mechanoreceptor differentiation | 2/22 | 67/18723 | 0.003 | CLRN1/POU4F3 | 2 |
| C15orf65 | GO:0090596 | sensory organ morphogenesis | 3/22 | 256/18723 | 0.003 | CLRN1/POU4F3/GLI2 | 3 |
| C15orf65 | GO:0001942 | hair follicle development | 2/22 | 81/18723 | 0.004 | GLI2/DNASE1L2 | 2 |
| C15orf65 | GO:0022404 | molting cycle process | 2/22 | 84/18723 | 0.004 | GLI2/DNASE1L2 | 2 |
| C15orf65 | GO:0022405 | hair cycle process | 2/22 | 84/18723 | 0.004 | GLI2/DNASE1L2 | 2 |
| C15orf65 | GO:0048562 | embryonic organ morphogenesis | 3/22 | 285/18723 | 0.004 | CLRN1/POU4F3/GLI2 | 3 |
| C15orf65 | GO:0098773 | skin epidermis development | 2/22 | 85/18723 | 0.004 | GLI2/DNASE1L2 | 2 |
| C15orf65 | GO:0045582 | positive regulation of T cell differentiation | 2/22 | 91/18723 | 0.005 | NLRP3/GLI2 | 2 |
| C15orf65 | GO:0045621 | positive regulation of lymphocyte differentiation | 2/22 | 104/18723 | 0.007 | NLRP3/GLI2 | 2 |
| C15orf65 | GO:0042303 | molting cycle | 2/22 | 107/18723 | 0.007 | GLI2/DNASE1L2 | 2 |
| C15orf65 | GO:0042633 | hair cycle | 2/22 | 107/18723 | 0.007 | GLI2/DNASE1L2 | 2 |
| C15orf65 | GO:0034446 | substrate adhesion-dependent cell spreading | 2/22 | 108/18723 | 0.007 | NTN4/FN1 | 2 |
| C15orf65 | GO:0002526 | acute inflammatory response | 2/22 | 112/18723 | 0.008 | NLRP3/FN1 | 2 |
| C15orf65 | GO:0022612 | gland morphogenesis | 2/22 | 118/18723 | 0.008 | NTN4/GLI2 | 2 |
| C15orf65 | GO:0048675 | axon extension | 2/22 | 120/18723 | 0.009 | POU4F3/FN1 | 2 |
| C15orf65 | GO:0007409 | axonogenesis | 3/22 | 418/18723 | 0.012 | POU4F3/GLI2/FN1 | 3 |
| C15orf65 | GO:0061351 | neural precursor cell proliferation | 2/22 | 145/18723 | 0.012 | DIXDC1/GLI2 | 2 |
| C15orf65 | GO:0045580 | regulation of T cell differentiation | 2/22 | 146/18723 | 0.013 | NLRP3/GLI2 | 2 |
| C15orf65 | GO:0050905 | neuromuscular process | 2/22 | 148/18723 | 0.013 | CLRN1/POU4F3 | 2 |
| C15orf65 | GO:0048568 | embryonic organ development | 3/22 | 427/18723 | 0.013 | CLRN1/POU4F3/GLI2 | 3 |
| C15orf65 | GO:0007605 | sensory perception of sound | 2/22 | 154/18723 | 0.014 | CLRN1/POU4F3 | 2 |
| C15orf65 | GO:0045785 | positive regulation of cell adhesion | 3/22 | 437/18723 | 0.014 | NLRP3/GLI2/FN1 | 3 |
| C15orf65 | GO:1902107 | positive regulation of leukocyte differentiation | 2/22 | 157/18723 | 0.014 | NLRP3/GLI2 | 2 |
| C15orf65 | GO:1903708 | positive regulation of hemopoiesis | 2/22 | 157/18723 | 0.014 | NLRP3/GLI2 | 2 |
| C15orf65 | GO:2000241 | regulation of reproductive process | 2/22 | 168/18723 | 0.016 | PLB1/TPPP2 | 2 |
| C15orf65 | GO:0061564 | axon development | 3/22 | 467/18723 | 0.017 | POU4F3/GLI2/FN1 | 3 |
| C15orf65 | GO:1990138 | neuron projection extension | 2/22 | 172/18723 | 0.017 | POU4F3/FN1 | 2 |
| C15orf65 | GO:0045619 | regulation of lymphocyte differentiation | 2/22 | 174/18723 | 0.018 | NLRP3/GLI2 | 2 |
| C15orf65 | GO:0050954 | sensory perception of mechanical stimulus | 2/22 | 175/18723 | 0.018 | CLRN1/POU4F3 | 2 |
| C15orf65 | GO:0061138 | morphogenesis of a branching epithelium | 2/22 | 182/18723 | 0.019 | NTN4/GLI2 | 2 |
| C15orf65 | GO:0043433 | negative regulation of DNA-binding transcription factor activity | 2/22 | 185/18723 | 0.020 | SIK1/NLRP3 | 2 |
| C15orf65 | GO:0001763 | morphogenesis of a branching structure | 2/22 | 196/18723 | 0.022 | NTN4/GLI2 | 2 |
| C15orf65 | GO:0045664 | regulation of neuron differentiation | 2/22 | 196/18723 | 0.022 | DIXDC1/GLI2 | 2 |
| C15orf65 | GO:0010952 | positive regulation of peptidase activity | 2/22 | 197/18723 | 0.022 | NLRP3/FN1 | 2 |
| C15orf65 | GO:0007601 | visual perception | 2/22 | 215/18723 | 0.026 | CLRN1/POU4F3 | 2 |
| C15orf65 | GO:0050870 | positive regulation of T cell activation | 2/22 | 216/18723 | 0.026 | NLRP3/GLI2 | 2 |
| C15orf65 | GO:0050953 | sensory perception of light stimulus | 2/22 | 219/18723 | 0.027 | CLRN1/POU4F3 | 2 |
| C15orf65 | GO:0002064 | epithelial cell development | 2/22 | 220/18723 | 0.027 | CLRN1/DNASE1L2 | 2 |
| C15orf65 | GO:0007411 | axon guidance | 2/22 | 227/18723 | 0.029 | POU4F3/GLI2 | 2 |
| C15orf65 | GO:0097485 | neuron projection guidance | 2/22 | 228/18723 | 0.029 | POU4F3/GLI2 | 2 |
| C15orf65 | GO:0048588 | developmental cell growth | 2/22 | 234/18723 | 0.030 | POU4F3/FN1 | 2 |
| C15orf65 | GO:0060560 | developmental growth involved in morphogenesis | 2/22 | 234/18723 | 0.030 | POU4F3/FN1 | 2 |
| C15orf65 | GO:1903039 | positive regulation of leukocyte cell-cell adhesion | 2/22 | 239/18723 | 0.032 | NLRP3/GLI2 | 2 |
| C15orf65 | GO:0032886 | regulation of microtubule-based process | 2/22 | 240/18723 | 0.032 | TPPP2/DIXDC1 | 2 |
| C15orf65 | GO:0006814 | sodium ion transport | 2/22 | 245/18723 | 0.033 | SLC6A19/SIK1 | 2 |
| C15orf65 | GO:0030217 | T cell differentiation | 2/22 | 257/18723 | 0.036 | NLRP3/GLI2 | 2 |
| C15orf65 | GO:0043588 | skin development | 2/22 | 263/18723 | 0.038 | GLI2/DNASE1L2 | 2 |
| C15orf65 | GO:1902105 | regulation of leukocyte differentiation | 2/22 | 279/18723 | 0.042 | NLRP3/GLI2 | 2 |
| C15orf65 | GO:0022409 | positive regulation of cell-cell adhesion | 2/22 | 284/18723 | 0.043 | NLRP3/GLI2 | 2 |
| C15orf65 | GO:0044403 | biological process involved in symbiotic interaction | 2/22 | 290/18723 | 0.045 | CLDN6/FN1 | 2 |
| C15orf65 | hsa05135 | Yersinia infection | 2/13 | 137/8142 | 0.019 | NLRP3/FN1 | 2 |
| C15orf65 | hsa04217 | Necroptosis | 2/13 | 159/8142 | 0.026 | NLRP3/H2AC7 | 2 |
| C15orf65 | hsa05130 | Pathogenic Escherichia coli infection | 2/13 | 197/8142 | 0.038 | NLRP3/CLDN6 | 2 |
| ZNF705B | GO:0042246 | tissue regeneration | 2/11 | 72/18723 | 7.843E-04 | YAP1/FZD9 | 2 |
| ZNF705B | GO:0001656 | metanephros development | 2/11 | 85/18723 | 1.091E-03 | FMN1/YAP1 | 2 |
| ZNF705B | GO:0072080 | nephron tubule development | 2/11 | 91/18723 | 1.249E-03 | FMN1/YAP1 | 2 |
| ZNF705B | GO:0061326 | renal tubule development | 2/11 | 94/18723 | 1.332E-03 | FMN1/YAP1 | 2 |
| ZNF705B | GO:0090263 | positive regulation of canonical Wnt signaling pathway | 2/11 | 106/18723 | 0.002 | YAP1/FZD9 | 2 |
| ZNF705B | GO:0072009 | nephron epithelium development | 2/11 | 109/18723 | 0.002 | FMN1/YAP1 | 2 |
| ZNF705B | GO:0072073 | kidney epithelium development | 2/11 | 136/18723 | 0.003 | FMN1/YAP1 | 2 |
| ZNF705B | GO:0030177 | positive regulation of Wnt signaling pathway | 2/11 | 140/18723 | 0.003 | YAP1/FZD9 | 2 |
| ZNF705B | GO:0072006 | nephron development | 2/11 | 142/18723 | 0.003 | FMN1/YAP1 | 2 |
| ZNF705B | GO:0009755 | hormone-mediated signaling pathway | 2/11 | 190/18723 | 0.005 | YAP1/ASIP | 2 |
| ZNF705B | GO:0031099 | regeneration | 2/11 | 198/18723 | 0.006 | YAP1/FZD9 | 2 |
| ZNF705B | GO:2001234 | negative regulation of apoptotic signaling pathway | 2/11 | 224/18723 | 0.007 | YAP1/FZD9 | 2 |
| ZNF705B | GO:0060560 | developmental growth involved in morphogenesis | 2/11 | 234/18723 | 0.008 | FMN1/YAP1 | 2 |
| ZNF705B | GO:0060828 | regulation of canonical Wnt signaling pathway | 2/11 | 253/18723 | 0.009 | YAP1/FZD9 | 2 |
| ZNF705B | GO:0001822 | kidney development | 2/11 | 293/18723 | 0.012 | FMN1/YAP1 | 2 |
| ZNF705B | GO:0072001 | renal system development | 2/11 | 302/18723 | 0.013 | FMN1/YAP1 | 2 |
| ZNF705B | GO:0060070 | canonical Wnt signaling pathway | 2/11 | 303/18723 | 0.013 | YAP1/FZD9 | 2 |
| ZNF705B | GO:0060562 | epithelial tube morphogenesis | 2/11 | 325/18723 | 0.015 | FMN1/YAP1 | 2 |
| ZNF705B | GO:0030111 | regulation of Wnt signaling pathway | 2/11 | 328/18723 | 0.015 | YAP1/FZD9 | 2 |
| ZNF705B | GO:0001655 | urogenital system development | 2/11 | 338/18723 | 0.016 | FMN1/YAP1 | 2 |
| ZNF705B | GO:0010639 | negative regulation of organelle organization | 2/11 | 348/18723 | 0.017 | YAP1/FZD9 | 2 |
| ZNF705B | GO:2001233 | regulation of apoptotic signaling pathway | 2/11 | 356/18723 | 0.018 | YAP1/FZD9 | 2 |
| ZNF705B | GO:0014706 | striated muscle tissue development | 2/11 | 384/18723 | 0.020 | YAP1/MYOM2 | 2 |
| ZNF705B | GO:0060537 | muscle tissue development | 2/11 | 403/18723 | 0.022 | YAP1/MYOM2 | 2 |
| ZNF705B | GO:0001503 | ossification | 2/11 | 408/18723 | 0.023 | YAP1/FZD9 | 2 |
| ZNF705B | GO:0016055 | Wnt signaling pathway | 2/11 | 444/18723 | 0.027 | YAP1/FZD9 | 2 |
| ZNF705B | GO:0198738 | cell-cell signaling by wnt | 2/11 | 446/18723 | 0.027 | YAP1/FZD9 | 2 |
| ZNF705B | hsa04916 | Melanogenesis | 2/5 | 101/8142 | 0.001 | FZD9/ASIP | 2 |
| ZNF705B | hsa04390 | Hippo signaling pathway | 2/5 | 157/8142 | 0.004 | YAP1/FZD9 | 2 |
| ZNF705B | hsa05022 | Pathways of neurodegeneration - multiple diseases | 2/5 | 476/8142 | 0.030 | FZD9/PRPH | 2 |
| RGS10 | GO:0016055 | Wnt signaling pathway | 2/5 | 444/18723 | 0.005 | DKKL1/CELA1 | 2 |
| RGS10 | GO:0198738 | cell-cell signaling by wnt | 2/5 | 446/18723 | 0.005 | DKKL1/CELA1 | 2 |
| CHRNA9 | GO:0003230 | cardiac atrium development | 4/70 | 36/18723 | 9.641E-06 | HEY2/WNT5A/PROX1/ANK2 | 4 |
| CHRNA9 | GO:0003279 | cardiac septum development | 5/70 | 103/18723 | 4.164E-05 | XIRP2/HEY2/WNT5A/PROX1/ANK2 | 5 |
| CHRNA9 | GO:0055008 | cardiac muscle tissue morphogenesis | 4/70 | 60/18723 | 7.459E-05 | XIRP2/HEY2/WNT5A/PROX1 | 4 |
| CHRNA9 | GO:0003283 | atrial septum development | 3/70 | 23/18723 | 8.400E-05 | HEY2/WNT5A/ANK2 | 3 |
| CHRNA9 | GO:0045664 | regulation of neuron differentiation | 6/70 | 196/18723 | 9.161E-05 | HEY2/TRPC6/PROX1/NRCAM/MYCN/ADRA2B | 6 |
| CHRNA9 | GO:0060415 | muscle tissue morphogenesis | 4/70 | 70/18723 | 1.364E-04 | XIRP2/HEY2/WNT5A/PROX1 | 4 |
| CHRNA9 | GO:0003281 | ventricular septum development | 4/70 | 71/18723 | 1.441E-04 | XIRP2/HEY2/WNT5A/PROX1 | 4 |
| CHRNA9 | GO:0003209 | cardiac atrium morphogenesis | 3/70 | 29/18723 | 1.706E-04 | HEY2/WNT5A/PROX1 | 3 |
| CHRNA9 | GO:0048644 | muscle organ morphogenesis | 4/70 | 77/18723 | 1.974E-04 | XIRP2/HEY2/WNT5A/PROX1 | 4 |
| CHRNA9 | GO:0045162 | clustering of voltage-gated sodium channels | 2/70 | 6/18723 | 2.047E-04 | SPTBN4/NRCAM | 2 |
| CHRNA9 | GO:0003205 | cardiac chamber development | 5/70 | 161/18723 | 3.407E-04 | XIRP2/HEY2/WNT5A/PROX1/ANK2 | 5 |
| CHRNA9 | GO:0003190 | atrioventricular valve formation | 2/70 | 8/18723 | 3.802E-04 | HEY2/OLFM1 | 2 |
| CHRNA9 | GO:0010463 | mesenchymal cell proliferation | 3/70 | 39/18723 | 4.153E-04 | DCHS2/WNT5A/MYCN | 3 |
| CHRNA9 | GO:0045666 | positive regulation of neuron differentiation | 4/70 | 95/18723 | 4.414E-04 | TRPC6/PROX1/NRCAM/ADRA2B | 4 |
| CHRNA9 | GO:0060412 | ventricular septum morphogenesis | 3/70 | 40/18723 | 4.478E-04 | HEY2/WNT5A/PROX1 | 3 |
| CHRNA9 | GO:0045607 | regulation of inner ear auditory receptor cell differentiation | 2/70 | 9/18723 | 4.877E-04 | HEY2/MYCN | 2 |
| CHRNA9 | GO:0045631 | regulation of mechanoreceptor differentiation | 2/70 | 9/18723 | 4.877E-04 | HEY2/MYCN | 2 |
| CHRNA9 | GO:2000980 | regulation of inner ear receptor cell differentiation | 2/70 | 9/18723 | 4.877E-04 | HEY2/MYCN | 2 |
| CHRNA9 | GO:0042551 | neuron maturation | 3/70 | 45/18723 | 6.346E-04 | SPTBN4/NRCAM/EPHA8 | 3 |
| CHRNA9 | GO:0048839 | inner ear development | 5/70 | 188/18723 | 6.907E-04 | CHRNA9/HEY2/WNT5A/PROX1/MYCN | 5 |
| CHRNA9 | GO:0035315 | hair cell differentiation | 3/70 | 47/18723 | 7.213E-04 | TMEM132E/HEY2/MYCN | 3 |
| CHRNA9 | GO:0045161 | neuronal ion channel clustering | 2/70 | 11/18723 | 7.415E-04 | SPTBN4/NRCAM | 2 |
| CHRNA9 | GO:0055015 | ventricular cardiac muscle cell development | 2/70 | 11/18723 | 7.415E-04 | HEY2/PROX1 | 2 |
| CHRNA9 | GO:0060900 | embryonic camera-type eye formation | 2/70 | 11/18723 | 7.415E-04 | WNT5A/PROX1 | 2 |
| CHRNA9 | GO:0006805 | xenobiotic metabolic process | 4/70 | 111/18723 | 7.940E-04 | UGT2B28/CYP46A1/SULT1A1/CYP2A6 | 4 |
| CHRNA9 | GO:0003231 | cardiac ventricle development | 4/70 | 123/18723 | 1.164E-03 | XIRP2/HEY2/WNT5A/PROX1 | 4 |
| CHRNA9 | GO:0043583 | ear development | 5/70 | 213/18723 | 1.208E-03 | CHRNA9/HEY2/WNT5A/PROX1/MYCN | 5 |
| CHRNA9 | GO:0003188 | heart valve formation | 2/70 | 14/18723 | 1.218E-03 | HEY2/OLFM1 | 2 |
| CHRNA9 | GO:0099638 | endosome to plasma membrane protein transport | 2/70 | 14/18723 | 1.218E-03 | TRARG1/ARHGAP44 | 2 |
| CHRNA9 | GO:0055012 | ventricular cardiac muscle cell differentiation | 2/70 | 15/18723 | 1.402E-03 | HEY2/PROX1 | 2 |
| CHRNA9 | GO:0048638 | regulation of developmental growth | 6/70 | 330/18723 | 1.456E-03 | SPTBN4/HEY2/OLFM1/WNT5A/PROX1/NRCAM | 6 |
| CHRNA9 | GO:0019748 | secondary metabolic process | 3/70 | 61/18723 | 0.002 | WNT5A/DCT/CYP2A6 | 3 |
| CHRNA9 | GO:0048588 | developmental cell growth | 5/70 | 234/18723 | 0.002 | POSTN/OLFM1/SORBS2/WNT5A/NRCAM | 5 |
| CHRNA9 | GO:0035418 | protein localization to synapse | 3/70 | 65/18723 | 0.002 | SHANK1/ARHGAP44/WNT5A | 3 |
| CHRNA9 | GO:0048738 | cardiac muscle tissue development | 5/70 | 236/18723 | 0.002 | XIRP2/HEY2/SORBS2/WNT5A/PROX1 | 5 |
| CHRNA9 | GO:0042490 | mechanoreceptor differentiation | 3/70 | 67/18723 | 0.002 | TMEM132E/HEY2/MYCN | 3 |
| CHRNA9 | GO:0060411 | cardiac septum morphogenesis | 3/70 | 68/18723 | 0.002 | HEY2/WNT5A/PROX1 | 3 |
| CHRNA9 | GO:0003007 | heart morphogenesis | 5/70 | 246/18723 | 0.002 | XIRP2/HEY2/OLFM1/WNT5A/PROX1 | 5 |
| CHRNA9 | GO:0001502 | cartilage condensation | 2/70 | 21/18723 | 0.003 | MYCN/MGP | 2 |
| CHRNA9 | GO:1902473 | regulation of protein localization to synapse | 2/70 | 21/18723 | 0.003 | ARHGAP44/WNT5A | 2 |
| CHRNA9 | GO:0002053 | positive regulation of mesenchymal cell proliferation | 2/70 | 22/18723 | 0.003 | WNT5A/MYCN | 2 |
| CHRNA9 | GO:0014706 | striated muscle tissue development | 6/70 | 384/18723 | 0.003 | XIRP2/HEY2/MYH15/SORBS2/WNT5A/PROX1 | 6 |
| CHRNA9 | GO:0042438 | melanin biosynthetic process | 2/70 | 23/18723 | 0.003 | WNT5A/DCT | 2 |
| CHRNA9 | GO:0003181 | atrioventricular valve morphogenesis | 2/70 | 24/18723 | 0.004 | HEY2/OLFM1 | 2 |
| CHRNA9 | GO:0006582 | melanin metabolic process | 2/70 | 24/18723 | 0.004 | WNT5A/DCT | 2 |
| CHRNA9 | GO:0051923 | sulfation | 2/70 | 24/18723 | 0.004 | HS3ST5/SULT1A1 | 2 |
| CHRNA9 | GO:0098743 | cell aggregation | 2/70 | 24/18723 | 0.004 | MYCN/MGP | 2 |
| CHRNA9 | GO:0044550 | secondary metabolite biosynthetic process | 2/70 | 25/18723 | 0.004 | WNT5A/DCT | 2 |
| CHRNA9 | GO:0048596 | embryonic camera-type eye morphogenesis | 2/70 | 25/18723 | 0.004 | WNT5A/PROX1 | 2 |
| CHRNA9 | GO:0007219 | Notch signaling pathway | 4/70 | 172/18723 | 0.004 | CHAC1/HEY2/POSTN/SORBS2 | 4 |
| CHRNA9 | GO:1990138 | neuron projection extension | 4/70 | 172/18723 | 0.004 | POSTN/OLFM1/WNT5A/NRCAM | 4 |
| CHRNA9 | GO:0060537 | muscle tissue development | 6/70 | 403/18723 | 0.004 | XIRP2/HEY2/MYH15/SORBS2/WNT5A/PROX1 | 6 |
| CHRNA9 | GO:0021700 | developmental maturation | 5/70 | 280/18723 | 0.004 | SPTBN4/SHANK1/WNT5A/NRCAM/EPHA8 | 5 |
| CHRNA9 | GO:0055013 | cardiac muscle cell development | 3/70 | 85/18723 | 0.004 | HEY2/SORBS2/PROX1 | 3 |
| CHRNA9 | GO:2000177 | regulation of neural precursor cell proliferation | 3/70 | 87/18723 | 0.004 | WNT5A/PROX1/DCT | 3 |
| CHRNA9 | GO:0071466 | cellular response to xenobiotic stimulus | 4/70 | 177/18723 | 0.004 | UGT2B28/CYP46A1/SULT1A1/CYP2A6 | 4 |
| CHRNA9 | GO:0003171 | atrioventricular valve development | 2/70 | 27/18723 | 0.005 | HEY2/OLFM1 | 2 |
| CHRNA9 | GO:0010721 | negative regulation of cell development | 4/70 | 180/18723 | 0.005 | POSTN/WNT5A/TRPC6/MYCN | 4 |
| CHRNA9 | GO:0055006 | cardiac cell development | 3/70 | 91/18723 | 0.005 | HEY2/SORBS2/PROX1 | 3 |
| CHRNA9 | GO:0010464 | regulation of mesenchymal cell proliferation | 2/70 | 28/18723 | 0.005 | WNT5A/MYCN | 2 |
| CHRNA9 | GO:0055001 | muscle cell development | 4/70 | 184/18723 | 0.005 | HEY2/SORBS2/PROX1/ANK2 | 4 |
| CHRNA9 | GO:0008217 | regulation of blood pressure | 4/70 | 186/18723 | 0.005 | RASL10B/POSTN/NPR3/ADRA2B | 4 |
| CHRNA9 | GO:1902414 | protein localization to cell junction | 3/70 | 94/18723 | 0.005 | SHANK1/ARHGAP44/WNT5A | 3 |
| CHRNA9 | GO:0008593 | regulation of Notch signaling pathway | 3/70 | 95/18723 | 0.005 | CHAC1/HEY2/POSTN | 3 |
| CHRNA9 | GO:0030516 | regulation of axon extension | 3/70 | 95/18723 | 0.005 | OLFM1/WNT5A/NRCAM | 3 |
| CHRNA9 | GO:0042472 | inner ear morphogenesis | 3/70 | 96/18723 | 0.006 | CHRNA9/WNT5A/PROX1 | 3 |
| CHRNA9 | GO:0010800 | positive regulation of peptidyl-threonine phosphorylation | 2/70 | 30/18723 | 0.006 | WNT5A/TRPC6 | 2 |
| CHRNA9 | GO:0061311 | cell surface receptor signaling pathway involved in heart development | 2/70 | 30/18723 | 0.006 | HEY2/WNT5A | 2 |
| CHRNA9 | GO:0032148 | activation of protein kinase B activity | 2/70 | 31/18723 | 0.006 | WNT5A/ADRA2B | 2 |
| CHRNA9 | GO:0070588 | calcium ion transmembrane transport | 5/70 | 312/18723 | 0.006 | MICU3/TMEM37/CHRNA9/TRPC6/ANK2 | 5 |
| CHRNA9 | GO:0060317 | cardiac epithelial to mesenchymal transition | 2/70 | 32/18723 | 0.006 | HEY2/OLFM1 | 2 |
| CHRNA9 | GO:0006874 | cellular calcium ion homeostasis | 6/70 | 448/18723 | 0.007 | MICU3/CHRNA9/WNT5A/TRPC6/BOK/ANK2 | 6 |
| CHRNA9 | GO:0048048 | embryonic eye morphogenesis | 2/70 | 33/18723 | 0.007 | WNT5A/PROX1 | 2 |
| CHRNA9 | GO:0002027 | regulation of heart rate | 3/70 | 103/18723 | 0.007 | SPTBN4/HEY2/ANK2 | 3 |
| CHRNA9 | GO:0008202 | steroid metabolic process | 5/70 | 319/18723 | 0.007 | UGT2B28/CYP46A1/SULT1A1/PROX1/CYP2A6 | 5 |
| CHRNA9 | GO:0009913 | epidermal cell differentiation | 4/70 | 202/18723 | 0.007 | TMEM132E/HEY2/WNT5A/MYCN | 4 |
| CHRNA9 | GO:0060419 | heart growth | 3/70 | 104/18723 | 0.007 | HEY2/SORBS2/PROX1 | 3 |
| CHRNA9 | GO:0035909 | aorta morphogenesis | 2/70 | 34/18723 | 0.007 | HEY2/PROX1 | 2 |
| CHRNA9 | GO:0008544 | epidermis development | 5/70 | 324/18723 | 0.007 | TMEM132E/HEY2/WNT5A/MYCN/DCT | 5 |
| CHRNA9 | GO:0055074 | calcium ion homeostasis | 6/70 | 460/18723 | 0.007 | MICU3/CHRNA9/WNT5A/TRPC6/BOK/ANK2 | 6 |
| CHRNA9 | GO:0007517 | muscle organ development | 5/70 | 327/18723 | 0.008 | XIRP2/HEY2/MYH15/WNT5A/PROX1 | 5 |
| CHRNA9 | GO:0031076 | embryonic camera-type eye development | 2/70 | 35/18723 | 0.008 | WNT5A/PROX1 | 2 |
| CHRNA9 | GO:0006699 | bile acid biosynthetic process | 2/70 | 36/18723 | 0.008 | CYP46A1/PROX1 | 2 |
| CHRNA9 | GO:0045746 | negative regulation of Notch signaling pathway | 2/70 | 36/18723 | 0.008 | CHAC1/HEY2 | 2 |
| CHRNA9 | GO:0018958 | phenol-containing compound metabolic process | 3/70 | 110/18723 | 0.008 | WNT5A/SULT1A1/DCT | 3 |
| CHRNA9 | GO:0061387 | regulation of extent of cell growth | 3/70 | 110/18723 | 0.008 | OLFM1/WNT5A/NRCAM | 3 |
| CHRNA9 | GO:0042491 | inner ear auditory receptor cell differentiation | 2/70 | 38/18723 | 0.009 | HEY2/MYCN | 2 |
| CHRNA9 | GO:0042471 | ear morphogenesis | 3/70 | 115/18723 | 0.009 | CHRNA9/WNT5A/PROX1 | 3 |
| CHRNA9 | GO:0072503 | cellular divalent inorganic cation homeostasis | 6/70 | 486/18723 | 0.010 | MICU3/CHRNA9/WNT5A/TRPC6/BOK/ANK2 | 6 |
| CHRNA9 | GO:0048675 | axon extension | 3/70 | 120/18723 | 0.010 | OLFM1/WNT5A/NRCAM | 3 |
| CHRNA9 | GO:0051480 | regulation of cytosolic calcium ion concentration | 5/70 | 353/18723 | 0.010 | CHRNA9/WNT5A/TRPC6/BOK/ANK2 | 5 |
| CHRNA9 | GO:0003206 | cardiac chamber morphogenesis | 3/70 | 121/18723 | 0.011 | HEY2/WNT5A/PROX1 | 3 |
| CHRNA9 | GO:0071695 | anatomical structure maturation | 4/70 | 229/18723 | 0.011 | SPTBN4/WNT5A/NRCAM/EPHA8 | 4 |
| CHRNA9 | GO:0030574 | collagen catabolic process | 2/70 | 42/18723 | 0.011 | MMP21/MMP11 | 2 |
| CHRNA9 | GO:0007368 | determination of left/right symmetry | 3/70 | 124/18723 | 0.011 | MMP21/DYNC2LI1/WNT5A | 3 |
| CHRNA9 | GO:0060560 | developmental growth involved in morphogenesis | 4/70 | 234/18723 | 0.011 | POSTN/OLFM1/WNT5A/NRCAM | 4 |
| CHRNA9 | GO:0055007 | cardiac muscle cell differentiation | 3/70 | 125/18723 | 0.011 | HEY2/SORBS2/PROX1 | 3 |
| CHRNA9 | GO:0050767 | regulation of neurogenesis | 5/70 | 364/18723 | 0.012 | HEY2/WNT5A/TRPC6/MYCN/DCT | 5 |
| CHRNA9 | GO:1901617 | organic hydroxy compound biosynthetic process | 4/70 | 237/18723 | 0.012 | CYP46A1/WNT5A/PROX1/DCT | 4 |
| CHRNA9 | GO:0046189 | phenol-containing compound biosynthetic process | 2/70 | 45/18723 | 0.012 | WNT5A/DCT | 2 |
| CHRNA9 | GO:0060421 | positive regulation of heart growth | 2/70 | 45/18723 | 0.012 | HEY2/PROX1 | 2 |
| CHRNA9 | GO:0055010 | ventricular cardiac muscle tissue morphogenesis | 2/70 | 46/18723 | 0.013 | HEY2/PROX1 | 2 |
| CHRNA9 | GO:0060976 | coronary vasculature development | 2/70 | 46/18723 | 0.013 | MMP21/HEY2 | 2 |
| CHRNA9 | GO:0010799 | regulation of peptidyl-threonine phosphorylation | 2/70 | 47/18723 | 0.013 | WNT5A/TRPC6 | 2 |
| CHRNA9 | GO:0009855 | determination of bilateral symmetry | 3/70 | 133/18723 | 0.014 | MMP21/DYNC2LI1/WNT5A | 3 |
| CHRNA9 | GO:0009799 | specification of symmetry | 3/70 | 134/18723 | 0.014 | MMP21/DYNC2LI1/WNT5A | 3 |
| CHRNA9 | GO:0008206 | bile acid metabolic process | 2/70 | 49/18723 | 0.014 | CYP46A1/PROX1 | 2 |
| CHRNA9 | GO:0009066 | aspartate family amino acid metabolic process | 2/70 | 49/18723 | 0.014 | ASPG/PHGDH | 2 |
| CHRNA9 | GO:0001961 | positive regulation of cytokine-mediated signaling pathway | 2/70 | 50/18723 | 0.015 | C1QTNF4/WNT5A | 2 |
| CHRNA9 | GO:0090102 | cochlea development | 2/70 | 50/18723 | 0.015 | HEY2/WNT5A | 2 |
| CHRNA9 | GO:2000677 | regulation of transcription regulatory region DNA binding | 2/70 | 50/18723 | 0.015 | ZBTB7C/HEY2 | 2 |
| CHRNA9 | GO:0045598 | regulation of fat cell differentiation | 3/70 | 139/18723 | 0.015 | ZBTB7C/WNT5A/MMP11 | 3 |
| CHRNA9 | GO:1903522 | regulation of blood circulation | 4/70 | 256/18723 | 0.015 | SPTBN4/HEY2/ANK2/ADRA2B | 4 |
| CHRNA9 | GO:0050768 | negative regulation of neurogenesis | 3/70 | 140/18723 | 0.016 | WNT5A/TRPC6/MYCN | 3 |
| CHRNA9 | GO:0009948 | anterior/posterior axis specification | 2/70 | 51/18723 | 0.016 | HEY2/WNT5A | 2 |
| CHRNA9 | GO:0021872 | forebrain generation of neurons | 2/70 | 51/18723 | 0.016 | PROX1/DCT | 2 |
| CHRNA9 | GO:0008584 | male gonad development | 3/70 | 141/18723 | 0.016 | ADAM29/WNT5A/BOK | 3 |
| CHRNA9 | GO:0046546 | development of primary male sexual characteristics | 3/70 | 142/18723 | 0.016 | ADAM29/WNT5A/BOK | 3 |
| CHRNA9 | GO:0072132 | mesenchyme morphogenesis | 2/70 | 52/18723 | 0.016 | HEY2/WNT5A | 2 |
| CHRNA9 | GO:0032970 | regulation of actin filament-based process | 5/70 | 397/18723 | 0.016 | SPTBN4/SHANK1/ARHGAP44/PROX1/ANK2 | 5 |
| CHRNA9 | GO:0051961 | negative regulation of nervous system development | 3/70 | 145/18723 | 0.017 | WNT5A/TRPC6/MYCN | 3 |
| CHRNA9 | GO:0061351 | neural precursor cell proliferation | 3/70 | 145/18723 | 0.017 | WNT5A/PROX1/DCT | 3 |
| CHRNA9 | GO:0048813 | dendrite morphogenesis | 3/70 | 146/18723 | 0.017 | SHANK1/ARHGAP44/TRPC6 | 3 |
| CHRNA9 | GO:2000179 | positive regulation of neural precursor cell proliferation | 2/70 | 54/18723 | 0.017 | PROX1/DCT | 2 |
| CHRNA9 | GO:0002067 | glandular epithelial cell differentiation | 2/70 | 55/18723 | 0.018 | WNT5A/PROX1 | 2 |
| CHRNA9 | GO:0003179 | heart valve morphogenesis | 2/70 | 55/18723 | 0.018 | HEY2/OLFM1 | 2 |
| CHRNA9 | GO:0003229 | ventricular cardiac muscle tissue development | 2/70 | 55/18723 | 0.018 | HEY2/PROX1 | 2 |
| CHRNA9 | GO:0046164 | alcohol catabolic process | 2/70 | 55/18723 | 0.018 | CYP46A1/SULT1A1 | 2 |
| CHRNA9 | GO:0042733 | embryonic digit morphogenesis | 2/70 | 56/18723 | 0.019 | WNT5A/MYCN | 2 |
| CHRNA9 | GO:0045599 | negative regulation of fat cell differentiation | 2/70 | 56/18723 | 0.019 | WNT5A/MMP11 | 2 |
| CHRNA9 | GO:0046622 | positive regulation of organ growth | 2/70 | 56/18723 | 0.019 | HEY2/PROX1 | 2 |
| CHRNA9 | GO:0060760 | positive regulation of response to cytokine stimulus | 2/70 | 57/18723 | 0.019 | C1QTNF4/WNT5A | 2 |
| CHRNA9 | GO:0021915 | neural tube development | 3/70 | 152/18723 | 0.019 | PHGDH/WNT5A/PROX1 | 3 |
| CHRNA9 | GO:0035904 | aorta development | 2/70 | 58/18723 | 0.020 | HEY2/PROX1 | 2 |
| CHRNA9 | GO:0045604 | regulation of epidermal cell differentiation | 2/70 | 58/18723 | 0.020 | HEY2/MYCN | 2 |
| CHRNA9 | GO:0007409 | axonogenesis | 5/70 | 418/18723 | 0.020 | SPTBN4/OLFM1/WNT5A/NRCAM/EPHA8 | 5 |
| CHRNA9 | GO:0035051 | cardiocyte differentiation | 3/70 | 156/18723 | 0.021 | HEY2/SORBS2/PROX1 | 3 |
| CHRNA9 | GO:0006816 | calcium ion transport | 5/70 | 422/18723 | 0.021 | MICU3/TMEM37/CHRNA9/TRPC6/ANK2 | 5 |
| CHRNA9 | GO:0001837 | epithelial to mesenchymal transition | 3/70 | 157/18723 | 0.021 | HEY2/OLFM1/WNT5A | 3 |
| CHRNA9 | GO:0061951 | establishment of protein localization to plasma membrane | 2/70 | 60/18723 | 0.021 | TRARG1/ARHGAP44 | 2 |
| CHRNA9 | GO:0048568 | embryonic organ development | 5/70 | 427/18723 | 0.022 | CHRNA9/HEY2/WNT5A/PROX1/MYCN | 5 |
| CHRNA9 | GO:0072659 | protein localization to plasma membrane | 4/70 | 284/18723 | 0.022 | TRARG1/SPTBN4/ARHGAP44/ANK2 | 4 |
| CHRNA9 | GO:0030199 | collagen fibril organization | 2/70 | 61/18723 | 0.022 | MMP11/FMOD | 2 |
| CHRNA9 | GO:0060997 | dendritic spine morphogenesis | 2/70 | 61/18723 | 0.022 | SHANK1/ARHGAP44 | 2 |
| CHRNA9 | GO:0048562 | embryonic organ morphogenesis | 4/70 | 285/18723 | 0.022 | CHRNA9/WNT5A/PROX1/MYCN | 4 |
| CHRNA9 | GO:0032757 | positive regulation of interleukin-8 production | 2/70 | 62/18723 | 0.023 | PRG3/WNT5A | 2 |
| CHRNA9 | GO:0046148 | pigment biosynthetic process | 2/70 | 62/18723 | 0.023 | WNT5A/DCT | 2 |
| CHRNA9 | GO:0060113 | inner ear receptor cell differentiation | 2/70 | 62/18723 | 0.023 | HEY2/MYCN | 2 |
| CHRNA9 | GO:0070059 | intrinsic apoptotic signaling pathway in response to endoplasmic reticulum stress | 2/70 | 63/18723 | 0.023 | CHAC1/BOK | 2 |
| CHRNA9 | GO:0046661 | male sex differentiation | 3/70 | 165/18723 | 0.024 | ADAM29/WNT5A/BOK | 3 |
| CHRNA9 | GO:0003170 | heart valve development | 2/70 | 65/18723 | 0.025 | HEY2/OLFM1 | 2 |
| CHRNA9 | GO:0045682 | regulation of epidermis development | 2/70 | 65/18723 | 0.025 | HEY2/MYCN | 2 |
| CHRNA9 | GO:0007015 | actin filament organization | 5/70 | 442/18723 | 0.025 | XIRP2/SPTBN4/SHANK1/SORBS2/PROX1 | 5 |
| CHRNA9 | GO:0051960 | regulation of nervous system development | 5/70 | 443/18723 | 0.025 | HEY2/WNT5A/TRPC6/MYCN/DCT | 5 |
| CHRNA9 | GO:0099173 | postsynapse organization | 3/70 | 168/18723 | 0.025 | SHANK1/ARHGAP44/WNT5A | 3 |
| CHRNA9 | GO:0061371 | determination of heart left/right asymmetry | 2/70 | 67/18723 | 0.026 | MMP21/WNT5A | 2 |
| CHRNA9 | GO:0030198 | extracellular matrix organization | 4/70 | 301/18723 | 0.026 | MMP21/POSTN/MMP11/FMOD | 4 |
| CHRNA9 | GO:0048469 | cell maturation | 3/70 | 171/18723 | 0.026 | SPTBN4/NRCAM/EPHA8 | 3 |
| CHRNA9 | GO:0043062 | extracellular structure organization | 4/70 | 302/18723 | 0.027 | MMP21/POSTN/MMP11/FMOD | 4 |
| CHRNA9 | GO:0045229 | external encapsulating structure organization | 4/70 | 304/18723 | 0.027 | MMP21/POSTN/MMP11/FMOD | 4 |
| CHRNA9 | GO:0048639 | positive regulation of developmental growth | 3/70 | 174/18723 | 0.027 | SPTBN4/HEY2/PROX1 | 3 |
| CHRNA9 | GO:0030324 | lung development | 3/70 | 177/18723 | 0.029 | WNT5A/PROX1/MYCN | 3 |
| CHRNA9 | GO:0003208 | cardiac ventricle morphogenesis | 2/70 | 71/18723 | 0.029 | HEY2/PROX1 | 2 |
| CHRNA9 | GO:0032722 | positive regulation of chemokine production | 2/70 | 71/18723 | 0.029 | POSTN/WNT5A | 2 |
| CHRNA9 | GO:0035265 | organ growth | 3/70 | 178/18723 | 0.029 | HEY2/SORBS2/PROX1 | 3 |
| CHRNA9 | GO:0046394 | carboxylic acid biosynthetic process | 4/70 | 314/18723 | 0.030 | PHGDH/CYP46A1/PRG3/PROX1 | 4 |
| CHRNA9 | GO:0008361 | regulation of cell size | 3/70 | 181/18723 | 0.030 | OLFM1/WNT5A/NRCAM | 3 |
| CHRNA9 | GO:0030323 | respiratory tube development | 3/70 | 181/18723 | 0.030 | WNT5A/PROX1/MYCN | 3 |
| CHRNA9 | GO:0061564 | axon development | 5/70 | 467/18723 | 0.030 | SPTBN4/OLFM1/WNT5A/NRCAM/EPHA8 | 5 |
| CHRNA9 | GO:0032456 | endocytic recycling | 2/70 | 73/18723 | 0.031 | TRARG1/ARHGAP44 | 2 |
| CHRNA9 | GO:0016053 | organic acid biosynthetic process | 4/70 | 316/18723 | 0.031 | PHGDH/CYP46A1/PRG3/PROX1 | 4 |
| CHRNA9 | GO:0061138 | morphogenesis of a branching epithelium | 3/70 | 182/18723 | 0.031 | WNT5A/PROX1/MYCN | 3 |
| CHRNA9 | GO:0003151 | outflow tract morphogenesis | 2/70 | 74/18723 | 0.031 | HEY2/WNT5A | 2 |
| CHRNA9 | GO:0048844 | artery morphogenesis | 2/70 | 74/18723 | 0.031 | HEY2/PROX1 | 2 |
| CHRNA9 | GO:0031345 | negative regulation of cell projection organization | 3/70 | 186/18723 | 0.033 | ARHGAP44/WNT5A/TRPC6 | 3 |
| CHRNA9 | GO:0031016 | pancreas development | 2/70 | 77/18723 | 0.034 | WNT5A/PROX1 | 2 |
| CHRNA9 | GO:0016049 | cell growth | 5/70 | 482/18723 | 0.034 | POSTN/OLFM1/SORBS2/WNT5A/NRCAM | 5 |
| CHRNA9 | GO:0051216 | cartilage development | 3/70 | 190/18723 | 0.034 | WNT5A/MYCN/MGP | 3 |
| CHRNA9 | GO:0007193 | adenylate cyclase-inhibiting G protein-coupled receptor signaling pathway | 2/70 | 78/18723 | 0.034 | NPR3/MTNR1A | 2 |
| CHRNA9 | GO:0070373 | negative regulation of ERK1 and ERK2 cascade | 2/70 | 78/18723 | 0.034 | CHRNA9/TIMP3 | 2 |
| CHRNA9 | GO:1901616 | organic hydroxy compound catabolic process | 2/70 | 78/18723 | 0.034 | CYP46A1/SULT1A1 | 2 |
| CHRNA9 | GO:0060420 | regulation of heart growth | 2/70 | 79/18723 | 0.035 | HEY2/PROX1 | 2 |
| CHRNA9 | GO:1990778 | protein localization to cell periphery | 4/70 | 333/18723 | 0.036 | TRARG1/SPTBN4/ARHGAP44/ANK2 | 4 |
| CHRNA9 | GO:1901605 | alpha-amino acid metabolic process | 3/70 | 195/18723 | 0.037 | ASPG/PHGDH/DCT | 3 |
| CHRNA9 | GO:0001763 | morphogenesis of a branching structure | 3/70 | 196/18723 | 0.037 | WNT5A/PROX1/MYCN | 3 |
| CHRNA9 | GO:0006790 | sulfur compound metabolic process | 4/70 | 339/18723 | 0.038 | HS3ST5/CHAC1/PHGDH/SULT1A1 | 4 |
| CHRNA9 | GO:0051899 | membrane depolarization | 2/70 | 83/18723 | 0.039 | BOK/ANK2 | 2 |
| CHRNA9 | GO:0002088 | lens development in camera-type eye | 2/70 | 84/18723 | 0.039 | WNT5A/PROX1 | 2 |
| CHRNA9 | GO:0015844 | monoamine transport | 2/70 | 84/18723 | 0.039 | SLC18A1/ADRA2B | 2 |
| CHRNA9 | GO:0042440 | pigment metabolic process | 2/70 | 84/18723 | 0.039 | WNT5A/DCT | 2 |
| CHRNA9 | GO:0097061 | dendritic spine organization | 2/70 | 84/18723 | 0.039 | SHANK1/ARHGAP44 | 2 |
| CHRNA9 | GO:0060541 | respiratory system development | 3/70 | 203/18723 | 0.040 | WNT5A/PROX1/MYCN | 3 |
| CHRNA9 | GO:0009798 | axis specification | 2/70 | 86/18723 | 0.041 | HEY2/WNT5A | 2 |
| CHRNA9 | GO:0008016 | regulation of heart contraction | 3/70 | 206/18723 | 0.042 | SPTBN4/HEY2/ANK2 | 3 |
| CHRNA9 | GO:0099175 | regulation of postsynapse organization | 2/70 | 90/18723 | 0.045 | ARHGAP44/WNT5A | 2 |
| CHRNA9 | GO:0032956 | regulation of actin cytoskeleton organization | 4/70 | 358/18723 | 0.045 | SPTBN4/SHANK1/ARHGAP44/PROX1 | 4 |
| CHRNA9 | GO:0002065 | columnar/cuboidal epithelial cell differentiation | 2/70 | 91/18723 | 0.046 | WNT5A/PROX1 | 2 |
| CHRNA9 | GO:0032755 | positive regulation of interleukin-6 production | 2/70 | 93/18723 | 0.047 | C1QTNF4/WNT5A | 2 |
| CHRNA9 | GO:0106027 | neuron projection organization | 2/70 | 93/18723 | 0.047 | SHANK1/ARHGAP44 | 2 |
| CHRNA9 | GO:0008406 | gonad development | 3/70 | 221/18723 | 0.049 | ADAM29/WNT5A/BOK | 3 |
| CHRNA9 | hsa05204 | Chemical carcinogenesis - DNA adducts | 3/36 | 69/8142 | 0.003 | UGT2B28/SULT1A1/CYP2A6 | 3 |
| CHRNA9 | hsa00561 | Glycerolipid metabolism | 2/36 | 61/8142 | 0.030 | GPAT2/DGKK | 2 |
| CHRNA9 | hsa00830 | Retinol metabolism | 2/36 | 68/8142 | 0.036 | UGT2B28/CYP2A6 | 2 |
| CHRNA9 | hsa00982 | Drug metabolism - cytochrome P450 | 2/36 | 72/8142 | 0.040 | UGT2B28/CYP2A6 | 2 |
| CHRNA9 | hsa04360 | Axon guidance | 3/36 | 182/8142 | 0.046 | WNT5A/TRPC6/EPHA8 | 3 |
| CHRNA9 | hsa00980 | Metabolism of xenobiotics by cytochrome P450 | 2/36 | 78/8142 | 0.046 | UGT2B28/CYP2A6 | 2 |
| CHRNA9 | hsa00983 | Drug metabolism - other enzymes | 2/36 | 80/8142 | 0.048 | UGT2B28/CYP2A6 | 2 |
| CHRNA9 | hsa05034 | Alcoholism | 3/36 | 187/8142 | 0.049 | H2BC13/H2AC13/SLC18A1 | 3 |
| KRT6A | GO:0060351 | cartilage development involved in endochondral bone morphogenesis | 2/24 | 30/18723 | 6.702E-04 | IHH/COL1A1 | 2 |
| KRT6A | GO:0060323 | head morphogenesis | 2/24 | 32/18723 | 7.629E-04 | IHH/COL1A1 | 2 |
| KRT6A | GO:0010171 | body morphogenesis | 2/24 | 43/18723 | 1.377E-03 | IHH/COL1A1 | 2 |
| KRT6A | GO:0032964 | collagen biosynthetic process | 2/24 | 51/18723 | 0.002 | IHH/COL1A1 | 2 |
| KRT6A | GO:0060350 | endochondral bone morphogenesis | 2/24 | 54/18723 | 0.002 | IHH/COL1A1 | 2 |
| KRT6A | GO:0051965 | positive regulation of synapse assembly | 2/24 | 60/18723 | 0.003 | LRRC4B/BDNF | 2 |
| KRT6A | GO:0060349 | bone morphogenesis | 2/24 | 93/18723 | 0.006 | IHH/COL1A1 | 2 |
| KRT6A | GO:0051963 | regulation of synapse assembly | 2/24 | 101/18723 | 0.007 | LRRC4B/BDNF | 2 |
| KRT6A | GO:0032963 | collagen metabolic process | 2/24 | 104/18723 | 0.008 | IHH/COL1A1 | 2 |
| KRT6A | GO:1901890 | positive regulation of cell junction assembly | 2/24 | 104/18723 | 0.008 | LRRC4B/BDNF | 2 |
| KRT6A | GO:0040029 | regulation of gene expression, epigenetic | 2/24 | 105/18723 | 0.008 | DNMT3L/LHX2 | 2 |
| KRT6A | GO:0048706 | embryonic skeletal system development | 2/24 | 121/18723 | 0.010 | IHH/COL1A1 | 2 |
| KRT6A | GO:0032355 | response to estradiol | 2/24 | 141/18723 | 0.014 | IHH/COL1A1 | 2 |
| KRT6A | GO:0060041 | retina development in camera-type eye | 2/24 | 147/18723 | 0.015 | LHX2/IHH | 2 |
| KRT6A | GO:0051960 | regulation of nervous system development | 3/24 | 443/18723 | 0.018 | LRRC4B/LHX2/BDNF | 3 |
| KRT6A | GO:0007416 | synapse assembly | 2/24 | 179/18723 | 0.022 | LRRC4B/BDNF | 2 |
| KRT6A | GO:0051216 | cartilage development | 2/24 | 190/18723 | 0.024 | IHH/COL1A1 | 2 |
| KRT6A | GO:1901888 | regulation of cell junction assembly | 2/24 | 204/18723 | 0.028 | LRRC4B/BDNF | 2 |
| KRT6A | GO:0060348 | bone development | 2/24 | 205/18723 | 0.028 | IHH/COL1A1 | 2 |
| KRT6A | GO:0050807 | regulation of synapse organization | 2/24 | 211/18723 | 0.030 | LRRC4B/BDNF | 2 |
| KRT6A | GO:0009612 | response to mechanical stimulus | 2/24 | 216/18723 | 0.031 | IHH/COL1A1 | 2 |
| KRT6A | GO:0050803 | regulation of synapse structure or activity | 2/24 | 218/18723 | 0.031 | LRRC4B/BDNF | 2 |
| KRT6A | GO:0048705 | skeletal system morphogenesis | 2/24 | 220/18723 | 0.032 | IHH/COL1A1 | 2 |
| KRT6A | GO:0007411 | axon guidance | 2/24 | 227/18723 | 0.034 | LHX2/BDNF | 2 |
| KRT6A | GO:0097485 | neuron projection guidance | 2/24 | 228/18723 | 0.034 | LHX2/BDNF | 2 |
| KRT6A | GO:0001649 | osteoblast differentiation | 2/24 | 229/18723 | 0.034 | IHH/COL1A1 | 2 |
| KRT6A | GO:0048588 | developmental cell growth | 2/24 | 234/18723 | 0.036 | LHX2/BDNF | 2 |
| KRT6A | GO:0060560 | developmental growth involved in morphogenesis | 2/24 | 234/18723 | 0.036 | LHX2/BDNF | 2 |
| KRT6A | GO:0061448 | connective tissue development | 2/24 | 252/18723 | 0.041 | IHH/COL1A1 | 2 |
| KRT6A | GO:0043588 | skin development | 2/24 | 263/18723 | 0.044 | LHX2/COL1A1 | 2 |
| KRT6A | GO:0051962 | positive regulation of nervous system development | 2/24 | 272/18723 | 0.047 | LRRC4B/BDNF | 2 |
| KRT6A | GO:0098742 | cell-cell adhesion via plasma-membrane adhesion molecules | 2/24 | 273/18723 | 0.047 | CDHR4/LRRC4B | 2 |
| KRT6A | hsa05205 | Proteoglycans in cancer | 2/8 | 205/8142 | 0.016 | IHH/COL1A1 | 2 |
| KRT6A | hsa04151 | PI3K-Akt signaling pathway | 2/8 | 354/8142 | 0.044 | COL1A1/BDNF | 2 |
| OGN | GO:0045785 | positive regulation of cell adhesion | 7/37 | 437/18723 | 2.020E-05 | MEGF10/PDCD1LG2/CHST2/NRP1/GFUS/VIT/IGF2 | 7 |
| OGN | GO:2000179 | positive regulation of neural precursor cell proliferation | 3/37 | 54/18723 | 1.644E-04 | GJC2/NES/DRD2 | 3 |
| OGN | GO:2000251 | positive regulation of actin cytoskeleton reorganization | 2/37 | 19/18723 | 6.362E-04 | CCL27/NRP1 | 2 |
| OGN | GO:2000177 | regulation of neural precursor cell proliferation | 3/37 | 87/18723 | 6.717E-04 | GJC2/NES/DRD2 | 3 |
| OGN | GO:0048641 | regulation of skeletal muscle tissue development | 2/37 | 24/18723 | 1.020E-03 | MEGF10/IGF2 | 2 |
| OGN | GO:0016079 | synaptic vesicle exocytosis | 3/37 | 105/18723 | 1.159E-03 | ERC2/CPLX2/DRD2 | 3 |
| OGN | GO:0040018 | positive regulation of multicellular organism growth | 2/37 | 29/18723 | 1.492E-03 | IGF2/DRD2 | 2 |
| OGN | GO:0050730 | regulation of peptidyl-tyrosine phosphorylation | 4/37 | 264/18723 | 0.002 | ALKAL2/NRP1/PPP2R5B/IGF2 | 4 |
| OGN | GO:0010811 | positive regulation of cell-substrate adhesion | 3/37 | 123/18723 | 0.002 | NRP1/GFUS/VIT | 3 |
| OGN | GO:0022409 | positive regulation of cell-cell adhesion | 4/37 | 284/18723 | 0.002 | MEGF10/PDCD1LG2/CHST2/IGF2 | 4 |
| OGN | GO:0016202 | regulation of striated muscle tissue development | 2/37 | 37/18723 | 0.002 | MEGF10/IGF2 | 2 |
| OGN | GO:1901861 | regulation of muscle tissue development | 2/37 | 39/18723 | 0.003 | MEGF10/IGF2 | 2 |
| OGN | GO:2000249 | regulation of actin cytoskeleton reorganization | 2/37 | 39/18723 | 0.003 | CCL27/NRP1 | 2 |
| OGN | GO:0007269 | neurotransmitter secretion | 3/37 | 145/18723 | 0.003 | ERC2/CPLX2/DRD2 | 3 |
| OGN | GO:0061351 | neural precursor cell proliferation | 3/37 | 145/18723 | 0.003 | GJC2/NES/DRD2 | 3 |
| OGN | GO:0099643 | signal release from synapse | 3/37 | 145/18723 | 0.003 | ERC2/CPLX2/DRD2 | 3 |
| OGN | GO:0048634 | regulation of muscle organ development | 2/37 | 41/18723 | 0.003 | MEGF10/IGF2 | 2 |
| OGN | GO:0046006 | regulation of activated T cell proliferation | 2/37 | 44/18723 | 0.003 | PDCD1LG2/IGF2 | 2 |
| OGN | GO:0035272 | exocrine system development | 2/37 | 45/18723 | 0.004 | NRP1/IGF2 | 2 |
| OGN | GO:0050798 | activated T cell proliferation | 2/37 | 47/18723 | 0.004 | PDCD1LG2/IGF2 | 2 |
| OGN | GO:0048639 | positive regulation of developmental growth | 3/37 | 174/18723 | 0.005 | NRP1/IGF2/DRD2 | 3 |
| OGN | GO:0099504 | synaptic vesicle cycle | 3/37 | 181/18723 | 0.005 | ERC2/CPLX2/DRD2 | 3 |
| OGN | GO:0051896 | regulation of protein kinase B signaling | 3/37 | 185/18723 | 0.006 | FAM110C/IGF2/DRD2 | 3 |
| OGN | GO:0001954 | positive regulation of cell-matrix adhesion | 2/37 | 58/18723 | 0.006 | NRP1/GFUS | 2 |
| OGN | GO:0007159 | leukocyte cell-cell adhesion | 4/37 | 371/18723 | 0.006 | PDCD1LG2/CHST2/GFUS/IGF2 | 4 |
| OGN | GO:0018108 | peptidyl-tyrosine phosphorylation | 4/37 | 375/18723 | 0.006 | ALKAL2/NRP1/PPP2R5B/IGF2 | 4 |
| OGN | GO:0018212 | peptidyl-tyrosine modification | 4/37 | 378/18723 | 0.006 | ALKAL2/NRP1/PPP2R5B/IGF2 | 4 |
| OGN | GO:0040014 | regulation of multicellular organism growth | 2/37 | 61/18723 | 0.006 | IGF2/DRD2 | 2 |
| OGN | GO:0050731 | positive regulation of peptidyl-tyrosine phosphorylation | 3/37 | 193/18723 | 0.006 | ALKAL2/NRP1/IGF2 | 3 |
| OGN | GO:0048167 | regulation of synaptic plasticity | 3/37 | 198/18723 | 0.007 | ERC2/CPLX2/DRD2 | 3 |
| OGN | GO:0099003 | vesicle-mediated transport in synapse | 3/37 | 200/18723 | 0.007 | ERC2/CPLX2/DRD2 | 3 |
| OGN | GO:0006836 | neurotransmitter transport | 3/37 | 208/18723 | 0.008 | ERC2/CPLX2/DRD2 | 3 |
| OGN | GO:0043491 | protein kinase B signaling | 3/37 | 211/18723 | 0.008 | FAM110C/IGF2/DRD2 | 3 |
| OGN | GO:0001505 | regulation of neurotransmitter levels | 3/37 | 213/18723 | 0.008 | ERC2/CPLX2/DRD2 | 3 |
| OGN | GO:0070374 | positive regulation of ERK1 and ERK2 cascade | 3/37 | 217/18723 | 0.009 | ALKAL2/NRP1/DRD2 | 3 |
| OGN | GO:0010810 | regulation of cell-substrate adhesion | 3/37 | 221/18723 | 0.009 | NRP1/GFUS/VIT | 3 |
| OGN | GO:0050769 | positive regulation of neurogenesis | 3/37 | 225/18723 | 0.010 | GJC2/NRP1/DRD2 | 3 |
| OGN | GO:0021536 | diencephalon development | 2/37 | 76/18723 | 0.010 | NRP1/DRD2 | 2 |
| OGN | GO:0140029 | exocytic process | 2/37 | 76/18723 | 0.010 | ERC2/CPLX2 | 2 |
| OGN | GO:0051495 | positive regulation of cytoskeleton organization | 3/37 | 226/18723 | 0.010 | CCL27/NES/NRP1 | 3 |
| OGN | GO:0045055 | regulated exocytosis | 3/37 | 230/18723 | 0.010 | ERC2/CPLX2/DRD2 | 3 |
| OGN | GO:0021954 | central nervous system neuron development | 2/37 | 81/18723 | 0.011 | NRP1/DRD2 | 2 |
| OGN | GO:0022407 | regulation of cell-cell adhesion | 4/37 | 448/18723 | 0.011 | MEGF10/PDCD1LG2/CHST2/IGF2 | 4 |
| OGN | GO:1903039 | positive regulation of leukocyte cell-cell adhesion | 3/37 | 239/18723 | 0.012 | PDCD1LG2/CHST2/IGF2 | 3 |
| OGN | GO:0046928 | regulation of neurotransmitter secretion | 2/37 | 86/18723 | 0.013 | CPLX2/DRD2 | 2 |
| OGN | GO:0051781 | positive regulation of cell division | 2/37 | 89/18723 | 0.013 | IGF2/DRD2 | 2 |
| OGN | GO:0045927 | positive regulation of growth | 3/37 | 259/18723 | 0.014 | NRP1/IGF2/DRD2 | 3 |
| OGN | GO:0043410 | positive regulation of MAPK cascade | 4/37 | 480/18723 | 0.014 | ALKAL2/NRP1/IGF2/DRD2 | 4 |
| OGN | GO:0051962 | positive regulation of nervous system development | 3/37 | 272/18723 | 0.016 | GJC2/NRP1/DRD2 | 3 |
| OGN | GO:0051588 | regulation of neurotransmitter transport | 2/37 | 100/18723 | 0.017 | CPLX2/DRD2 | 2 |
| OGN | GO:0042102 | positive regulation of T cell proliferation | 2/37 | 101/18723 | 0.017 | PDCD1LG2/IGF2 | 2 |
| OGN | GO:0021761 | limbic system development | 2/37 | 107/18723 | 0.019 | NRP1/DRD2 | 2 |
| OGN | GO:0031532 | actin cytoskeleton reorganization | 2/37 | 107/18723 | 0.019 | CCL27/NRP1 | 2 |
| OGN | GO:0014812 | muscle cell migration | 2/37 | 110/18723 | 0.020 | MEGF10/NRP1 | 2 |
| OGN | GO:0001938 | positive regulation of endothelial cell proliferation | 2/37 | 111/18723 | 0.020 | NRP1/IGF2 | 2 |
| OGN | GO:0015718 | monocarboxylic acid transport | 2/37 | 111/18723 | 0.020 | SLCO2B1/DRD2 | 2 |
| OGN | GO:0010720 | positive regulation of cell development | 3/37 | 298/18723 | 0.021 | GJC2/NRP1/DRD2 | 3 |
| OGN | GO:0070372 | regulation of ERK1 and ERK2 cascade | 3/37 | 309/18723 | 0.023 | ALKAL2/NRP1/DRD2 | 3 |
| OGN | GO:0010906 | regulation of glucose metabolic process | 2/37 | 119/18723 | 0.023 | IGF2/GCKR | 2 |
| OGN | GO:0051897 | positive regulation of protein kinase B signaling | 2/37 | 120/18723 | 0.023 | FAM110C/IGF2 | 2 |
| OGN | GO:0043010 | camera-type eye development | 3/37 | 322/18723 | 0.025 | NES/NRP1/DRD2 | 3 |
| OGN | GO:0001952 | regulation of cell-matrix adhesion | 2/37 | 128/18723 | 0.026 | NRP1/GFUS | 2 |
| OGN | GO:0048638 | regulation of developmental growth | 3/37 | 330/18723 | 0.027 | NRP1/IGF2/DRD2 | 3 |
| OGN | GO:0070371 | ERK1 and ERK2 cascade | 3/37 | 330/18723 | 0.027 | ALKAL2/NRP1/DRD2 | 3 |
| OGN | GO:0043467 | regulation of generation of precursor metabolites and energy | 2/37 | 130/18723 | 0.027 | IGF2/COX7A1 | 2 |
| OGN | GO:0035264 | multicellular organism growth | 2/37 | 132/18723 | 0.028 | IGF2/DRD2 | 2 |
| OGN | GO:0010595 | positive regulation of endothelial cell migration | 2/37 | 133/18723 | 0.028 | NRP1/GFUS | 2 |
| OGN | GO:1903037 | regulation of leukocyte cell-cell adhesion | 3/37 | 336/18723 | 0.028 | PDCD1LG2/CHST2/IGF2 | 3 |
| OGN | GO:0007266 | Rho protein signal transduction | 2/37 | 137/18723 | 0.030 | NRP1/RTKN | 2 |
| OGN | GO:0050671 | positive regulation of lymphocyte proliferation | 2/37 | 137/18723 | 0.030 | PDCD1LG2/IGF2 | 2 |
| OGN | GO:0032946 | positive regulation of mononuclear cell proliferation | 2/37 | 138/18723 | 0.030 | PDCD1LG2/IGF2 | 2 |
| OGN | GO:0050921 | positive regulation of chemotaxis | 2/37 | 141/18723 | 0.032 | CCL27/NRP1 | 2 |
| OGN | GO:0006887 | exocytosis | 3/37 | 352/18723 | 0.032 | ERC2/CPLX2/DRD2 | 3 |
| OGN | GO:0031346 | positive regulation of cell projection organization | 3/37 | 353/18723 | 0.032 | ALKAL2/NRP1/PPP2R5B | 3 |
| OGN | GO:0043524 | negative regulation of neuron apoptotic process | 2/37 | 145/18723 | 0.033 | NES/NRP1 | 2 |
| OGN | GO:0010675 | regulation of cellular carbohydrate metabolic process | 2/37 | 146/18723 | 0.034 | IGF2/GCKR | 2 |
| OGN | GO:0031589 | cell-substrate adhesion | 3/37 | 363/18723 | 0.035 | NRP1/GFUS/VIT | 3 |
| OGN | GO:0050767 | regulation of neurogenesis | 3/37 | 364/18723 | 0.035 | GJC2/NRP1/DRD2 | 3 |
| OGN | GO:0070665 | positive regulation of leukocyte proliferation | 2/37 | 150/18723 | 0.035 | PDCD1LG2/IGF2 | 2 |
| OGN | GO:0001654 | eye development | 3/37 | 371/18723 | 0.036 | NES/NRP1/DRD2 | 3 |
| OGN | GO:0150063 | visual system development | 3/37 | 375/18723 | 0.037 | NES/NRP1/DRD2 | 3 |
| OGN | GO:0007519 | skeletal muscle tissue development | 2/37 | 155/18723 | 0.038 | MEGF10/IGF2 | 2 |
| OGN | GO:0051147 | regulation of muscle cell differentiation | 2/37 | 155/18723 | 0.038 | MEGF10/IGF2 | 2 |
| OGN | GO:0001764 | neuron migration | 2/37 | 156/18723 | 0.038 | NRP1/DRD2 | 2 |
| OGN | GO:0048880 | sensory system development | 3/37 | 381/18723 | 0.039 | NES/NRP1/DRD2 | 3 |
| OGN | GO:1903169 | regulation of calcium ion transmembrane transport | 2/37 | 159/18723 | 0.039 | GJC2/DRD2 | 2 |
| OGN | GO:0042692 | muscle cell differentiation | 3/37 | 384/18723 | 0.040 | MEGF10/ADAM12/IGF2 | 3 |
| OGN | GO:0045860 | positive regulation of protein kinase activity | 3/37 | 386/18723 | 0.040 | ALKAL2/IGF2/DRD2 | 3 |
| OGN | GO:0021953 | central nervous system neuron differentiation | 2/37 | 162/18723 | 0.041 | NRP1/DRD2 | 2 |
| OGN | GO:0010976 | positive regulation of neuron projection development | 2/37 | 163/18723 | 0.041 | ALKAL2/PPP2R5B | 2 |
| OGN | GO:0060538 | skeletal muscle organ development | 2/37 | 166/18723 | 0.042 | MEGF10/IGF2 | 2 |
| OGN | GO:0042129 | regulation of T cell proliferation | 2/37 | 171/18723 | 0.045 | PDCD1LG2/IGF2 | 2 |
| OGN | GO:0000910 | cytokinesis | 2/37 | 173/18723 | 0.046 | RTKN/DRD2 | 2 |
| OGN | GO:0010469 | regulation of signaling receptor activity | 2/37 | 173/18723 | 0.046 | NRP1/PPP2R5B | 2 |
| OGN | GO:0022900 | electron transport chain | 2/37 | 175/18723 | 0.047 | GFUS/COX7A1 | 2 |
| OGN | GO:0010634 | positive regulation of epithelial cell migration | 2/37 | 176/18723 | 0.047 | NRP1/GFUS | 2 |
| OGN | GO:0051302 | regulation of cell division | 2/37 | 177/18723 | 0.048 | IGF2/DRD2 | 2 |
| OGN | GO:0006109 | regulation of carbohydrate metabolic process | 2/37 | 178/18723 | 0.048 | IGF2/GCKR | 2 |
| OGN | GO:0001936 | regulation of endothelial cell proliferation | 2/37 | 179/18723 | 0.049 | NRP1/IGF2 | 2 |
| OGN | GO:0045766 | positive regulation of angiogenesis | 2/37 | 181/18723 | 0.049 | NRP1/ADAM12 | 2 |
| OGN | GO:1904018 | positive regulation of vasculature development | 2/37 | 181/18723 | 0.049 | NRP1/ADAM12 | 2 |
| OGN | hsa04071 | Sphingolipid signaling pathway | 2/14 | 119/8142 | 0.017 | DEGS2/PPP2R5B | 2 |
| OGN | hsa04728 | Dopaminergic synapse | 2/14 | 132/8142 | 0.021 | PPP2R5B/DRD2 | 2 |
| HBG2 | GO:0015671 | oxygen transport | 2/11 | 15/18723 | 3.281E-05 | HBG2/HBG1 | 2 |
| HBG2 | GO:0015669 | gas transport | 2/11 | 20/18723 | 5.928E-05 | HBG2/HBG1 | 2 |
| HBG2 | GO:0042744 | hydrogen peroxide catabolic process | 2/11 | 32/18723 | 1.542E-04 | HBG2/HBG1 | 2 |
| HBG2 | GO:0042743 | hydrogen peroxide metabolic process | 2/11 | 58/18723 | 5.095E-04 | HBG2/HBG1 | 2 |
| HBG2 | GO:0098869 | cellular oxidant detoxification | 2/11 | 101/18723 | 0.002 | HBG2/HBG1 | 2 |
| HBG2 | GO:1990748 | cellular detoxification | 2/11 | 116/18723 | 0.002 | HBG2/HBG1 | 2 |
| HBG2 | GO:0097237 | cellular response to toxic substance | 2/11 | 124/18723 | 0.002 | HBG2/HBG1 | 2 |
| HBG2 | GO:0098754 | detoxification | 2/11 | 152/18723 | 0.003 | HBG2/HBG1 | 2 |
| HBG2 | GO:0072593 | reactive oxygen species metabolic process | 2/11 | 239/18723 | 0.008 | HBG2/HBG1 | 2 |
| HBG2 | GO:0009636 | response to toxic substance | 2/11 | 262/18723 | 0.010 | HBG2/HBG1 | 2 |
| HBG2 | GO:0016311 | dephosphorylation | 2/11 | 417/18723 | 0.024 | G6PC2/PPEF1 | 2 |
| DIRAS2 | GO:0030900 | forebrain development | 2/17 | 379/18723 | 0.045 | CDK5R2/KIF1A | 2 |
| DIRAS2 | hsa04270 | Vascular smooth muscle contraction | 2/9 | 134/8142 | 0.009 | MYH14/ACTG2 | 2 |
| ZBED9 | GO:0009584 | detection of visible light | 2/31 | 51/18723 | 0.003 | SEMA5B/RGR | 2 |
| ZBED9 | GO:0009583 | detection of light stimulus | 2/31 | 67/18723 | 0.005 | SEMA5B/RGR | 2 |
| ZBED9 | GO:0006520 | cellular amino acid metabolic process | 3/31 | 284/18723 | 0.011 | ACCSL/GLUD2/DPYS | 3 |
| ZBED9 | GO:0071868 | cellular response to monoamine stimulus | 2/31 | 107/18723 | 0.014 | RGS8/PKLR | 2 |
| ZBED9 | GO:0071870 | cellular response to catecholamine stimulus | 2/31 | 107/18723 | 0.014 | RGS8/PKLR | 2 |
| ZBED9 | GO:0071867 | response to monoamine | 2/31 | 111/18723 | 0.014 | RGS8/PKLR | 2 |
| ZBED9 | GO:0071869 | response to catecholamine | 2/31 | 111/18723 | 0.014 | RGS8/PKLR | 2 |
| ZBED9 | GO:0009581 | detection of external stimulus | 2/31 | 135/18723 | 0.021 | SEMA5B/RGR | 2 |
| ZBED9 | GO:0009582 | detection of abiotic stimulus | 2/31 | 138/18723 | 0.022 | SEMA5B/RGR | 2 |
| ZBED9 | GO:0035725 | sodium ion transmembrane transport | 2/31 | 178/18723 | 0.035 | SLC6A13/FGF14 | 2 |
| ZBED9 | GO:0048863 | stem cell differentiation | 2/31 | 206/18723 | 0.045 | SEMA5B/ESRRB | 2 |
| ZBED9 | GO:0007601 | visual perception | 2/31 | 215/18723 | 0.049 | SEMA5B/RGR | 2 |
| ZBED9 | hsa04950 | Maturity onset diabetes of the young | 2/17 | 26/8142 | 0.001 | NEUROG3/PKLR | 2 |
| ZBED9 | hsa04612 | Antigen processing and presentation | 2/17 | 78/8142 | 0.011 | KIR2DL5A/KIR2DS5 | 2 |
| ZBED9 | hsa00983 | Drug metabolism - other enzymes | 2/17 | 80/8142 | 0.012 | DPYS/CYP2A7 | 2 |
| ZBED9 | hsa01200 | Carbon metabolism | 2/17 | 115/8142 | 0.023 | PKLR/GLUD2 | 2 |
| ZBED9 | hsa04650 | Natural killer cell mediated cytotoxicity | 2/17 | 131/8142 | 0.030 | KIR2DL5A/KIR2DS5 | 2 |
