# Supplementary material for: Effects of Danhong Injection on 90‐Day Functional Outcome in Acute Ischemic Stroke: An Adaptive, Multicenter, Randomized, Double‐Blind, Placebo‐Controlled Phase IV Trial
Source: MedComm (2020). 2026 Nov 8;7(11):e71135. doi: 10.1002/mco2.71135 (PMC13744646; doi:10.1002/mco2.71135)
Supplement: Supplementary file 2 [file MCO2-7-e71135-s002.docx]

**Supplement Files**

**Protocol &Statistical Analysis Plan**

**This supplement contains the following items:**

1. **Original protocol (Page 1-40)**
2. **Final protocol (Page 41-102)**
3. **Summary of changes in the protocol (Page 103)**
4. **Original statistical analysis plan (Page 104-123)**
5. **Final statistical analysis plan (Page124-145)**
6. **Summary of changes in the statistical analysis plan (Page 146)**

**Protocol (Original)**

**The Original Version: June 21, 2012**

**Danhong Injection for the treatment of acute ischemic stroke (Phase IV): an adaptive-design, randomized, multi-center, double-blind, placebo-controlled clinical trial**

**Sponsored by:** China National Science and Technology Major Project for "Significant New Drugs Development" (2011ZX09304-07)

**CLINICAL TRIAL STEERING COMMITTEE**

**Prof. Yong-Yan Wang (Chair),** Institute of Basic Research in Clinical Medicine, China Academy of Chinese Medical Sciences

**Prof. Xiao-Xi Du (Member),** State Food and Drug Administration

**Prof. Bo-Li Zhang (Member)**, China Academy of Chinese Medical Sciences

**Prof. Run-Lin Gao (Member)**, Fuwai Hospital Chinese Academy of Medical Sciences

**Prof. Wei-Liang Weng (Member)**, Xi-yuan Hospital Affiliated to China Academy of Chinese Medical Sciences

**Dr. Ai-Ping Lv (Member)**, 1. Institute of Basic Research in Clinical Medicine, China Academy of Chinese Medical Sciences; 2. School of Chinese Medicine, Hong Kong Baptist University

**Prof. Ming-Hui Yang (Member)**, Chinese PLA General Hospital

**PRINCIPLE INVESTIGATORS**

**Prof. Zhong Wang, Ph.D (for grant)**

Institute of Basic Research in Clinical Medicine, China Academy of Chinese Medical Sciences

No.16 Nanxiaojie, Dongzhimennei, Beijing, 100700, China.

Tel:86-10-64093305

Email: zhonw@vip.sina.com

**Prof. Xingquan Zhao, M.D. (for trial)**

Department of Neurology, Beijing Tiantan Hospital, Capital Medical University .

Tiantan xili 6, Dongcheng, Beijing 100050, P.R. China.

E-mail: zxq@vip.163.com

**PARTICIPATING SITES AND INVESTIGATOR**

1. **Beijing Tiantan Hospital, Capital Medical University**

**Prof. Xingquan Zhao, M.D.**

Site Principal Investigator

Department of Neurology

Beijing Tiantan Hospital, Capital Medical University .

Tiantan xili 6, Dongcheng, Beijing, 100050, China.

E-mail: zxq@vip.163.com.
2. **Peking University First Hospital**

**Prof. Xuezhi Zhang, M.D.**

Site Principal Investigator

Department of Traditional Chinese Medicine

Peking University First Hospital

No. 8 nishiku Street, Beijing, 100034, China.

E-mail: zhang.xuezhi@263.net.

3. **The Second Artillery General Hospital of Chinese People's Liberation Army**

**Prof. Lei Wang, M.D.**

Site Principal Investigator,

Department of Neurology

The Second Artillery General Hospital of Chinese People's Liberation Army

No. 16 Xinjiekou Wai Street, Beijing, 100088, China

E-mail: hellowanglei068@163.com

4. **Peking University of People's Hospital**

**Prof. Xuguang Gao, M.D.**

Site Principal Investigator

Department of Neurology

Peking University of People's Hospital

No.11 South Avenue Street, Beijing, 100044, China

E-mail: gxg56@tom.com.

5. **Xuan Wu Hospital Affiliated to Capital Medical University**

**Prof. Li Gao.**

Site Principal Investigator

Department of Neurology

Xuan Wu Hospital Affiliated to Capital Medical University

No.45 Chang Chun Street, Beijing, 100053, China

Email: xuanwugaoli@126.com

6. **The First Affiliated Hospital of Henan University of T.C.M.**

**Prof. Xinzhi Wang**

Site Principal Investigator

Department of Neurology

The First Affiliated Hospital of Henan University of T.C.M.

No. 19, Renmin Road, Zhengzhou, 450000, Henan, China

Email: [znqznq@163.com](mailto:znqznq@163.com)

**7. Xiangya Hospital, Central South University**

**Prof. Xiaosu Yang, M.D.**

Site Principal Investigator

Department of Neurology

Xiangya Hospital Central-south University

No. 87, Xiangya Road, Changsha, 410008, Hunan, China,

Email: [sjnk_xy@yahoo.com.cn](mailto:sjnk_xy@yahoo.com.cn)

**8. Renmin Hospital, Wuhan University**

**Prof. Zhaohui Zhang, M.D**

Site Principal Investigator

Department of Neurology

Renmin Hospital of Wuhan University

No. 238, Jiefang Road, Wuhan, 430060, Hubei, China.

Email:zhzhqing1990@163.com

**9. Zhongnan Hospital, Wuhan University**

**Prof. Yumin Liu, M.D**

Site Principal Investigator

Department of Neurology

Zhongnan Hospital of Wuhan University

No. 169, Donghu Road, Wuhan, 430030, Hubei, China.

Email:lym9381@123.com

**10. Affiliated Zhongshan Hospital of Dalian University**

**Prof. Qiang Ma**

Site Principal Investigator

Department of Neurology

Affiliated Zhongshan Hospital of Dalian University

No. 6, Jiefang Street, Dalian, 116001, Liaoning, China.

Email: [mqiang2100@yahoo.com.cn](mailto:mqiang2100@yahoo.com.cn)

**DATA MONITORING COMMITTEE (DMC)**

Prof. Chun-Ti Shen (Chair), Changzhou TCM Hospital Affiliated to Nanjing University of Chinese Medicine

Prof. Qi-Guang Chen (Member, independent statistician), Southeast University

Prof. Hua-Qiang Zhang (Member), Jiangsu Provincial Commission of Health and Family Planning

Dr. Hong-Wu Wang (Member), Tianjin University of Traditional Chinese Medicine

Dr. Xiao-Chun Sun (Member), Beijing Kelunrui Pharmaceutical Technology Co. Ltd.

Prof. Cheng-Shu Wang (Member), Zhongda Hospital Southeast University

Dr. Hai-Xia Dang (Member), China Academy of Chinese Medical Sciences

Dr. Jun Liu (Member), Institute of Basic Research in Clinical Medicine, China Academy of Chinese Medical Sciences

**PROTOCOL STATISTICIANS**

**Prof. Bi-Wei Chen, Ph.D.,** School of Public Health, Southeast University

Dijia Qiao 87, Nanjing,210009, Jiangsu,China

E-mail: drchenbw@126.com

**TABLE OF** **CONTENTS**

[EXECUTIVE COMMITTEE CHAIR	I](#_Toc5349)

[PRINCIPLE INVESTIGATORS	I](#_Toc2352)

[PARTICIPATING SITES AND INVESTIGATOR	II](#_Toc17115)

[DATA MONITORING COMMITTEE (DMC)	VI](#_Toc24674)

[PROTOCOL STATISTICIANS	VI](#_Toc15784)

[PROTOCOL SYNOPSIS	- 1 -](#_Toc1011)

[1. BACKGROUND INFORMATION	- 4 -](#_Toc22447)

[2. OBJECTIVES	- 5 -](#_Toc26066)

[3. STUDY DESIGN	- 5 -](#_Toc26580)

[3.1 Overview	- 5 -](#_Toc17214)

[3.2 Sample size	- 5 -](#_Toc6299)

[3.3 Randomization	- 6 -](#_Toc32233)

[3.4 Blinding	- 6 -](#_Toc2369)

[3.5 Interim analysis	- 6 -](#_Toc13088)

[4．SUBJECTS	- 7 -](#_Toc6340)

[4.1 Diagnostic criteria	- 7 -](#_Toc10564)

[4.2 Inclusion Criteria	- 8 -](#_Toc12782)

[4.3 Exclusion Criteria	- 9 -](#_Toc11811)

[4.4 Withdrawal/dropout criteria	- 10 -](#_Toc26840)

[4.5 Criteria for study termination	- 11 -](#_Toc5355)

[4.6 Criteria for subject discontinuation	- 11 -](#_Toc32141)

[4.7 Trial flow Chart	- 12 -](#_Toc14535)

[5 INTERVENTION	- 13 -](#_Toc13484)

[5.1 Routine medical care	- 13 -](#_Toc20847)

[5.2 Treatment regimens	- 14 -](#_Toc15937)

[5.3 Duration and follow-up	- 14 -](#_Toc4399)

[5.4 Concomitant medications	- 14 -](#_Toc25636)

[6 OUTCOMES MEASUREMENTS	- 14 -](#_Toc53)

[6.1 Primary outcome	- 14 -](#_Toc17574)

[6.2 Secondary Outcomes	- 15 -](#_Toc26395)

[6.3 Other Pre-specified Efficacy Outcomes	- 17 -](#_Toc17236)

[6.4 Safety Outcomes	- 17 -](#_Toc27409)

[6.5 Study Schema	- 17 -](#_Toc26898)

[7 ADVERSE EVENTS	- 19 -](#_Toc20502)

[7.1 Definition	- 19 -](#_Toc30180)

[7.2 Recording	- 20 -](#_Toc14870)

[7.3 Severity of an AE	- 20 -](#_Toc14874)

[7.4 Evaluation the correlation between AEs and study drugs	- 20 -](#_Toc19441)

[7.5 Handling of SAEs	- 22 -](#_Toc14777)

[7.6 Opening and handling of emergency envelops	- 22 -](#_Toc15277)

[7.7 Follow-up of unresolved AEs	- 22 -](#_Toc29784)

[8. DATA COLLECTION AND MANAGEMENT	- 22 -](#_Toc28015)

[8.1 Establishment of Three Committees	- 22 -](#_Toc15569)

[8.2 Data Management	- 23 -](#_Toc29413)

[8.3 Data Report	- 23 -](#_Toc10092)

[8.4 Data Blind Review	- 24 -](#_Toc31554)

[8.5 Database Locking	- 24 -](#_Toc2671)

[9. STATISTICAL CONSIDERATIONS	- 25 -](#_Toc24586)

[9.1 Study Populations	- 25 -](#_Toc11329)

[9.2 Statistical Analyses	- 25 -](#_Toc7478)

[9.3 Statistical Analysis Plan (SAP)	- 26 -](#_Toc21369)

[10 ETHICAL ASPECTS	- 26 -](#_Toc14201)

[REFERENCES	- 27 -](#_Toc20626)

**PROTOCOL SYNOPSIS**

TITLE:

Danhong Injection for the treatment of acute ischemic stroke (Phase IV): an adaptive-design, randomized, multi-center, double-blind, placebo-controlled clinical trial.

DESIGN:

A multi-center, randomized, placebo-controlled, double-blind, adaptive clinical trial (superiority design)

NUMBER OF SITES:

10 sites

SAMPLE SIZE:

A total of 846 participants with acute ischemic stroke with “Xueyu-Zheng” (Blood Stasis Syndrome) were planned initially.

POPULATION:

Patients aged 18 to 70 years who suffered from acute ischemic stroke with cerebral anterior circulation

obstruction with a clinical diagnosis of “Xueyu-Zheng” (Blood Stasis Syndrome), 4≤NIHSS score＜20 were eligible for inclusion in the study.

RANDOMIZATION：

Randomization to Danhong Injection (DHI) group or control group (0.9% normal saline as placebo) will be in a 2:1 ratio.

REGIMEN:

Routine medical care (RMC) was provided to all patients according to the China Guideline for the Diagnosis and Treatment of Acute Ischemic Stroke, including control of temperature, blood pressure, lipid and glucose, improving cerebral blood circulation, anti-platelet treatment, anticoagulants, neuroprotective drugs, stroke rehabilitation, and stroke prevention care.

DHI group: RMC + DHI (40 ml, qd) plus 0.9% normal saline (250 ml, ivgtt, qd)

Control group: RMC + placebo of DHI (0.9% normal saline, 40ml, qd) plus 0.9% normal saline (250 ml, ivgtt, qd)

TREATMENT DURATION:

14 days

FOLLOWING UP

76 days after treatment

OUTCOME MEASURES:

Efficacy:

Primary outcome:

The proportion of patients with excellent functional outcome, defined as a score of 0–1 on the Modified Rankin Scale (mRS) at day 90.

Secondary outcomes:

(1). The proportion of patients with a significant improvement in “Xueyu Zheng”, defined as at least 70% reduction in the Efficacy Evaluation Scale of Chinese Medicine Symptom in “Xueyu Zheng”;

(2). The proportion of patients with the Barthel Index (BI) score of ≥90;

(3) The proportion of patients with an improvement in the NIHSS score of ≥4 or the NIHSS score of 0-1;

(4) The quality of life measured by the EQ-5D scale;

(5) Global disability (mRS: 3-5) at 90 days;

(6) Incidence of new-onset major vascular events within 90 days;

Other pre-specified efficacy endpoints are:

(1) The changes on the profiles of micro-RNA in 96 patients selected from certain centers；

(2) The changes on the expression profiles of mRNA in 96 patients selected from certain centers.

Safety:

(1) Overall mortality within 90 days;

(2) Incidence of severe haemorrhages according to the GUSTO bleeding criteria within 90 days;

(3) Incidence of moderate haemorrhages according to the GUSTO bleeding criteria within 90 days;

(4) Incidence of adverse and serious adverse events.

1. **BACKGROUND INFORMATION**

Acute ischemic stroke (AIS), characterized by an occlusion of the cerebral artery, is one of the leading causes of mortality, long-term disability, and morbidity. Thrombolytic therapy with intravenous tissue plasminogen activator (tPA), which remains the only therapeutic drug for AIS approved by the US Food and Drug Administration, is limited by the narrow time window of thrombolysis, bleeding complications, or high costs^1^. Other treatment strategies mainly utilize therapeutic agents to prevent or reduce cell damage from ischemia^2^. Hundreds of neuro protective agents for ischemic stroke have reached clinical trials, but no optimal drugs for the clinical treatment of AIS have yet been developed^3^. This leads us to search for novel therapeutic approaches, and to investigate whether natural compounds extracted from herbs may be a novel option to improve treatment for AIS^4^.

Danhong injection (DHI), extracted from Radix Salviae miltiorrhizae (Danshen) and Flos Carthami tinctorii (Honghua), is a Chinese medicinal product approved by the State Food and Drug Administration (SFDA), and it is widely used in China for treating AIS^5^. Previous studies demonstrated the neuroprotective efficacy of DHI in rat models with cerebral ischemic-reperfusion injury^6,7^, and that the underlying pharmacological mechanisms might involve anti-inflammatory, anti-coagulant, antithrombotic, antifibrinolytic, and antioxidant activities^8^. A number of clinical studies also supported the efficacy and safety of DHI in the management of AIS, but due to the methodological deficiencies of inadequate randomization, no double blinding, no placebo control, or incomplete outcome data, the quality of these studies was assessed as generally low^9^. There is a lack of critically appraised evidence such as well designed randomized controlled trials (RCTs) to justify its clinical use and recommendation. Thus, we aim to investigate the efficacy and safety of DHI in the treatment of AIS in a well-designed, randomized, multicenter, double-blind and placebo-controlled trial.

**2. OBJECTIVES**

The objective of this study is mainly to determine whether Danhong injection is effective in the treatment of acute ischemic stroke.

**3. STUDY DESIGN**

**3.1 Overview**

This study is a three-stage adaptive-design, randomized, multicenter, double-blind, placebo-controlled trial. The number of subjects is initially estimated to be 846 and randomly assigned to be 564 in DHI group and 282 in the control group. Both groups received routine medical care as recent guideline advocated. Participants in the DHI group are treated by DHI (40 ml, qd) plus 0.9% normal saline (250 ml, ivgtt, qd), while participants in the control group are treated by DHI placebo (0.9% normal saline, 40ml, qd) plus 0.9% normal saline (250 ml, ivgtt, qd).

**3.2 Sample size**

The proportion of patients who had a favorable outcome, defined as a modified Rankin Scale (mRS) score of 0–1, afterroutine medical care coupled with placebo or DHI for 90 days was unclear. According to previous studies^10, 11^, the proportion of patients who had a mRS score of 0–1 was about 28.7–45.2 % when treated with placebo, so we estimate the proportion is 45% when routine medical care with placebo are used. In this trial, it is hypothesized that an increase of at least 10 % is of clinical significance for the DHI group. This is an adaptive-designed trial with 3-stage, and using EAST5.2, the number of subjects was initially estimated to be 703 (two-sided test, α =0.05, β =0.2). To allow for a dropout rate of 20%, a total of 846 patients will be recruited. With a 2:1 randomization ratio, the number of participants in the DHI group is 564 and that in the control group is 282. We planned interim analyses at n = 282 and n = 564 patients using the O’Brien-Fleming boundary approach.

**3.3** **Randomization**

All eligible patients who consent to participation will be randomized into either the Danhong injection or the placebo group in a 2:1 ratio. Randomization will be conducted using the central online randomization system (Brightech, Somerset, USA).The system generated a randomization number and randomized patients in randomly generated blocks of six.

**3.4 Blinding**

All patients, treating physicians and statisticians will be blinded to Danhong injection therapy or placebo treatment group assignments until the study has completed. Because the colour of Danhong injection and 0.9 % saline are different,the dropping bottles will be wrapped in sealed shaded bags, and brown infusion devices will be used for infusion. These procedures will be implemented by two professional nurses who will be required to sign a confidentiality agreement before study initiation and not to contact each other. One of the professional nurses will be in charge of preparing the drugs in a special transfusion room and sealing the infusion bottles with shaded brown bags. The other nurse will take the prepared drugs from the transfusion room to the infusion nurse and supervise the infusion process to ensure that the allocation of the drugs is blinded to the patients (the shaded brown bags will not be unwrapped during infusion and will be checked for integrity after infusion).

**3.5 Interim analysis**

Because this is an adaptive trial, two interim analyses are prospectively planned, and these will be performed in an unblinded manner by an independent statistician in the data monitoring committee (DMC) support group after one-third and two-thirds of the patients, respectively, have completed the trial. The statistical results of the interim analyses will be discussed in a close meeting of the DMC, who will decide on the re-estimation of sample size and determine whether any subsequent modifications must be made to the trial protocol. Because the two interim analyses may lead to an increased possibility of a type I error, we will apply the Lan-DeMets alpha spending function with an O’Brien-Fleming boundary to adjust the results.

**4．SUBJECTS**

**4.1 Diagnostic criteria**

**4.1.1 Disease Diagnostic criteria**

The diagnostic criteria for acute ischemic stroke is determined according to the Chinese Guidelines for the Diagnosis and Treatment of Acute Ischemic Stroke (2010)^12^.

Ischemic stroke causing a measurable neurological deficit defined as impairment of language, motor function, cognition and/or gaze, vision or neglect. Ischemic stroke is defined as an event characterized by the sudden onset of an acute focal neurologic deficit presumed to be due to cerebral ischemia after computed tomography (CT) scan excludes hemorrhage.

Interpretation of imaging should be performed using the Alberta Stroke Program Early CT Score (ASPECTS, range 0-10, with 1 point subtracted for any evidence of early ischaemic change in each defined region on the CT or MRI scan)^13^ at a central core laboratory by two independent superior neurology imaging doctors.

**4.1.2. Zhenghou Diagnostic criteria**

The diagnostic criteria of the “Xueyu Zheng” in Stroke is determined according to the Guidelines for Clinical Research of New Drugs of Traditional Chinese Medicine (2002)^14^, Guideline for Diagnosis and Effect Evaluation of Stroke (1996)^15^ and Zhenghou Diagnostic Scale in Ischemic Stroke^16^.

We will choose the patients with a clinical diagnosis of “Xueyu Zheng” (blood stasis syndrome), which is defined as a score of at least 20 on the Chinese Medicine Symptom Diagnostic Scale of “Xueyu Zheng” for acute ischemic stroke patients.

The Chinese Medicine Symptom Diagnostic Scale of “Xueyu Zheng” includes the 12 items as Table 1**.**

**Table 1 Chinese Medicine Symptom Diagnostic Scale of “Xueyu Zheng”**

| Symptoms | With | Without | Score |
| --- | --- | --- | --- |
| Hemiplegia | 10 | 0 |  |
| Numbness of limbs | 10 | 0 |  |
| Dimmish and darkish complexion | 9 | 0 |  |
| Dark purple lips | 8 | 0 |  |
| Rough skin | 4 | 0 |  |
| Pain in a fixed body location | 5 | 0 |  |
| Purple or darkish tongue | 10 | 0 |  |
| Ecchymosis on the tongue | 10 | 0 |  |
| Purple sublingual vessels | 10 | 0 |  |
| Sublingual varices | 8 | 0 |  |
| Unsmooth pulse | 8 | 0 |  |
| Intermittent pulse | 1 | 0 |  |
| Total Score* | | |  |

* If the total score ≥20, the patiet should be diagnosed as ischemic stroke with “Xueyu Zheng”.

**4.2 Inclusion Criteria**

(1). Female or male inpatients.

(2). Age: 18–70 years.

(3). Clinical diagnosis of ischemic stroke causing a measurable neurological deficit defined as impairment of language, motor function, cognition and/or gaze, vision or neglect. Ischemic stroke is defined as an event characterized by the sudden onset of an acute focal neurologic deficit presumed to be due to cerebral ischemia after computed tomography (CT) scan excludes hemorrhage.

(4). Clinical diagnosis of "Xueyu Zheng" (Blood Stasis Syndrome) as the scores of Chinese Medicine Symptom Diagnostic Scale of "Xueyu Zheng" in ischemic stroke ≥ 20.

(5). Onset of symptoms within 1 week prior to initiation of study drug administration.

(6). Patients with a clinical diagnosis of cerebral anterior circulation obstruction.

(7). 4≤National Institutes of Health Stroke Scale (NIHSS) score <20.

(8). Patient is willing to participate voluntarily and to sign a written patient informed consent. Informed consent will be obtained from each patient or the subject's legally authorized representative or relatives.

**4.3 Exclusion Criteria**

(1). Evidence of intracranial hemorrhage (ICH) or other cerebral diseases (e.g., vascular malformation, tumor, abscess or multiple sclerosis) on the CT or magnetic resonance imaging (MRI) scan.

(2). Patients who underwent thrombolysis or endovascular treatment.

(3). Known history of allergy or suspected allergy to the study drug.

(4). Fasting blood glucose <2.8 or >16.8 mmol/l or with severe complications due to diabetes (e.g., peripheral neuropathy, or diabetic gangrene).

(5). Liver function impairment with the value of alanine aminotransferase (ALT) or aspartate aminotransaminase (AST) over 1.5-fold the upper limit of normal range.

(6). Renal dysfunction with the value of serum creatinine over 1.5-fold the upper limit of normal range.

(7). Severe cardiac dysfunction on echocardiogram or the New York Heart Association (NYHA) heart functional class III or IV.

(8). History of prior stroke with a modified Rankin Scale (mRS) score ≥2.

(9). Complicated with atrial fibrillation.

(10). Severe stroke as assessed by appropriate imaging techniques (e.g., massive cerebral infarction affecting more than one lobe of the brain or over one-third of the blood-supply area of the middle cerebral artery).

(11). Physical disability before onset.

(12).Hemorrhagic tendency or severe or dangerous bleeding within the prior 3 months.

(13).Suspected alcohol addiction or drug abuse.

(14).Severe complications that would make the condition more complicated as assessed by the investigator.

(15).Women who are pregnant, lactating or have a positive pregnancy test, or women who plan to become pregnant in the subsequent 6 months.

(16).Women who have a menstrual period at baseline.

(17).Patients who are participating in other trials or have participated in other trials within the prior 3 months.

**4.4 Withdrawal/dropout criteria**

(1) Those who did not fulfill the eligibility criteria but were included by mistake;
(2) Those who fulfilled the eligibility criteria but did not take any study drug after randomization;
(3) Those with poor compliance that might interfere with the efficacy and safety evaluation;
(4) Those who experienced serious AEs, complications or special physiological changes, making it inappropriate to continue with the trial;
(5) Individual cases that were unblinded prematurely;
(6) Those who discontinued the trial voluntarily;
(7) Those who used any disallowed concomitant medication, especially those that had an obvious effect on the study drug and might interfere with the efficacy and safety evaluation;
(8) Those who discontinued or lost to follow-up for any other reasons or died during the trial;
(9) Those with incomplete data that might interfere with the efficacy and safety evaluation.

For all withdrawal/dropout cases, the potential reasons must be recorded. Patients who experienced any AE during the trial must be included in the statistical analysis of adverse drug reactions. Those who have taken the study drug for at least 1 week should be included in the statistical analysis of drug efficacy.

**4.5 Criteria for study termination**

Circumstances that may warrant termination include, but are not limited to:

(1) Identification of unexpected, significant, or unacceptable risk to subjects;
(2) Poor efficacy or no efficacy of the study drug;
(3) Major mistakes in the trial protocol;
(4) Insufficient adherence to protocol requirements;
(5) Upon sponsor’s request (e.g. economic or administrative reasons).

**4.6 Criteria for subject discontinuation**

(1) Any allergic reaction or AE occurs such that continued participation in the study would not be in the best interest of the subject at the discretion of the treating physician;
(2) Worsening of clinical conditions occurs such that continued participation in the study would not be in the best interest of the subject at the discretion of the treating physician; considered as ineffective cases;
(3) Subjects are free to withdraw from participation in the study at any time upon request.

In any case, every effort must be made to determine why patients discontinued the study treatment prematurely, which may include but are not limited to poor confidence in efficacy, occurrence of AEs, SAEs, or other serious complications, worsening of symptoms such that emergent intervention is required. As for those who withdrew from participation in the study or lost to follow-up, it is essential to investigate the potential reasons via telephone. Investigators should record in detail when and how the last dose was administered, the efficacy and safety parameters at and after discontinuation, the relationship between discontinuation or withdrawal and the study drug, and the potential impact of discontinuation cases on the final conclusion of the study. Moreover, case report form (CRF) of withdrawal/dropout cases should also be completed and their original data should also be documented and stored in a secure manner.

**4.7 Trial flow Chart**

The trial flow chart was shown in Figure 1.

Figure 1. The schedule of enrollment, interventions, and assessments

**5 INTERVENTION**

**5.1 Routine medical care**

Routine medical care is in accordance with China Guideline for the Diagnosis and Treatment of Acute Ischemic Stroke (2010)^12^, including control of temperature, blood pressure, lipid and glucose, improving cerebral blood circulation, anti-platelet treatment, anticoagulants, neuroprotective drugs, stroke rehabilitation, and stroke prevention care.

All of these basic treatments will be recorded in detail in the patients’ medical records as well as in their e-CRFs.

**5.2 Treatment regimens**

DHI group: Participants will be treated using Danhong injection (40 ml per day) plus 0.9 % saline (250 ml intravenously per day).

Control group: Participants will be treated using placebo (0.9 % saline, 40 ml per day) plus 0.9 % saline (250 ml intravenously per day).

The trial drug should be administered as soon as possible after randomisation. Centralised monitoring of imaging and treatment speed, with feedback to sites, ensure adherence to patient eligibility criteria and to workflow metrics.

**5.3 Duration and follow-up**

All of the included patients will undergo a 14-day treatment regimen and a 76-day follow-up period.

**5.4 Concomitant medications**

(1) Participants are not allowed to take any other Chinese herbal medicines or Chinese patent medicines that have the effect of promoting blood circulation or removing blood stasis throughout the study period.

(2) If other medication or therapy is required to treat the concomitant diseases, the name of the drug or therapy, dosage, dosing frequency and start/stop time should be well-documented.

**6 OUTCOMES MEASUREMENTS**

**6.1 Primary outcome**

The primary outcome is the proportion of patients with excellent functional outcome, defined as a score of 0–1 on the mRS^17^ at day 90. The mRS is a commonly used scale for measuring the degree of disability or dependence in the daily activities of people who have suffered a stroke or other causes of neurological disability. The scale runs from 0–6, ranging from perfect health without symptoms to death.

**6.2 Secondary Outcomes**

6.2.1 The efficacy evaluation scale of Chinese Medicine Symptoms in “Xueyu Zheng” of ischemic stroke ^15^

The efficacy evaluation scale of Chinese Medicine Symptoms in “Xueyu Zheng” of ischemic stroke (Table 2) will be used to assess the condition of patients with *Xueyu Zheng* at each visit (Day 0, Day 7, Day 14, Day 30, Day 60, Day 90).

**Table 2 The efficacy evaluation scale of Chinese Medicine Symptom in “Xueyu Zheng” of ischemic stroke**

| Symptoms | Visual Analogue Scale* | Score |
| --- | --- | --- |
| Hemiplegia | 0------------------------------------10 |  |
| Numbness of limbs | 0------------------------------------10 |  |
| Dimmish and darkish complexion | 0------------------------------------9 |  |
| Dark purple lips | 0------------------------------------8 |  |
| Rough skin | 0------------------------------------4 |  |
| Pain in a fixed body location | 0------------------------------------5 |  |
| Purple or darkish tongue | 0------------------------------------10 |  |
| Ecchymosis on the tongue | 0------------------------------------10 |  |
| Purple sublingual vessels | 0------------------------------------10 |  |
| Sublingual varices | 0------------------------------------8 |  |
| Unsmooth pulse | 0------------------------------------8 |  |
| Intermittent pulse | 0------------------------------------1 |  |

* According to the severity of the symptoms, the more severe the symptom is, the higher the score be determined. If the symptom is disappeared, the score will be recorded as 0.

The mean total score of symptoms in a questionnaire of *Xueyu Zheng* will be analyzed and compared between two groups at each visit. Moreover, the proportions of patients with improvement in *Xueyue Zheng* between the two groups will be recorded at each visit. Significant “*Xueyu Zheng*” improvement is defined as at least 70% reduction in the *Xueyue Zheng* score^15^.

6.2.2 The proportion of patients with the Barthel Index (BI) score of ≥90.

BI uses ten variables to describe the activities of daily living (ADL) and mobility, and a higher number is associated with a better performance of ADL^18^. At each visit, investigators will observe the proportions of patients with the Barthel Index (BI) score of ≥90 in each treatment group.

6.2.3 The proportion of patients with an improvement in the NIHSS score of ≥4 or the NIHSS score of 0-1.

The patients will be asked to record their NIHSS score. NIHSS is a tool used to objectively quantify the impairment caused by stroke. A NIHSS score of 0 typically indicates normal function in that specific ability, while a higher score is indicative of some level of impairment^19^. At each visit (Day 7, 14, 30, 60, 90), the proportion of patients with an improvement in the NIHSS score of ≥4 or the NIHSS score of 0-1 will be compared between two groups.

6.2.4 The quality of life measured by the EQ-5D scale.

The EQ-5D scale has five standard dimensions to measure the generalized health-related quality of life^20^. It is a standardized instrument for the measurement of health status with Time Trade-Off Value for different race group. Here, we need to find the TTO value of EQ-5D for Chinese population. The mean TTO value of EQ-5D scale will be analyzed and compared between two groups at each visit (Day0, 7, 14, 30, 60, 90).

6.2.5 Global disability at day 90.

Global disability, which defined as the proportion of patients whose mRS≥3 except death, will be compared at Day 90.

6.2.6 Incidence of new-onset major vascular events within 90 days.

Major adverse vascular events include ischemic stroke, hemorrhagic stroke, transient ischemic attack, myocardial infarction and vascular-related death. The proportion of patients with new-onset major vascular events within 3 months will be compared between two groups.

**6.3 Other Pre-specified Efficacy Outcomes**

Changes in the micro-RNA and mRNA profiles of 96 patients selected from Tiantan Hospital and Xuanwu Hospital. The detail of these outcomes will be shown in the RNA sequencing protocol for the mechanism of Danhong injection in the treatment of ischemic stroke.

**6.4 Safety Outcomes**

6.4.1 Overall mortality within 90 days.

6.4.2 Incidence of severe haemorrhages within 90 days.

Severe haemorrhages is defined according to the GUSTO bleeding criteria^21^, including fatal bleeding, primary intracranial hemorrhage, post-traumatic symptomatic intracranial hemorrhage, or bleeding requiring transfusion, infusion, use of vasoconstrictor drugs and surgical intervention.

6.4.3 Incidence of moderate haemorrhages within 90 days.

Moderate haemorrhages is also defined according to the GUSTO bleeding criteria^21^, including bleeding requiring infusion but do not reach severe bleeding, absolute reduction in hemoglobin/hematocrit, severe disability induced by bleeding, intraocular hemorrhage with severe visual field defects, etc.

6.4.4 Incidence of adverse and serious adverse events (Section 7).

**6.5 Study Schema**

| **Period** | **Baseline** | **Treatment period** | | **Follow-up period** | | |
| --- | --- | --- | --- | --- | --- | --- |
| Day | 0 | 7±2 | 14±2 | 30±5 | 60±5 | 90±5 |
| Informed consent | × |  |  |  |  |  |
| Inclusion/exclusion criteria | × |  |  |  |  |  |
| Medical history | × |  |  |  |  |  |
| UCG | × |  |  |  |  |  |
| TOAST | × |  |  |  |  |  |
| ASPECTS | × |  |  |  |  |  |
| Medical examination | × | × | × | × | × | × |
| Concomitant medications | × | × | × | × | × | × |
| **Outcomes** |  |  |  |  |  |  |
| BI scale | × | × | × | × | × | × |
| mRS scale | × | × | × | × | × | × |
| NIHSS | × | × | × | × | × | × |
| Efficacy evaluation scale of Chinese Medicine Symptom in “Xueyu Zheng” | × | × | × | × | × | × |
| EQ-5D scale | × | × | × | × | × | × |
| Global disability on mRS |  |  |  |  |  | × |
| New-onset major vascular events |  |  |  |  |  | × |
| Overall mortality |  |  |  |  |  | × |
| Incidence of severe hemorrhages |  |  |  |  |  | × |
| Incidence of moderate hemorrhages |  |  |  |  |  | × |
| AEs and SAEs |  | × | × | × | × | × |
| Profiles of micro-RNA in 96 patients in certain centers | × |  |  | × |  | × |
| Profiles of mRNA in 96 patients in certain centers | × |  |  | × |  | × |

*UCG: ultrasound cardiogram; TOAST: Trial of Org 10 172 in Acute Stroke Treatment; ASPECTS: Alberta Stroke Programme Early CT Score; BI: Barthel Index; mRS: modified Rankin Scale; NIHSS: National Institutes of Health Stroke Scale; AEs: adverse events; SAEs: serious adverse events; microRNA: micro-ribonucleic acid; mRNA: messenger ribonucleic acid.

**7 ADVERSE EVENTS**

**7.1 Definition**

**Adverse event (AE)**: An AE is any untoward medical occurrence in a patient or clinical investigation subject administered a pharmaceutical product and that does not necessarily have a causal relationship with this treatment. An AE can therefore be any unfavorable and unintended sign (including an abnormal laboratory finding), symptom, or disease temporally associated with the use of a medicinal (investigational) product, whether or not related to the medicinal (investigational) product. Pre-existing conditions which worsen during a study are also to be reported as AEs.

**Serious Adverse Event (SAE):** A SAE is any experience that suggests a significant hazard, contraindication, side effect or precaution. It is any AE that at any dose fulfils at least one of the following criteria:

– is fatal;

– is life-threatening;

– required in-patient hospitalization or prolongation of existing hospitalization;

– results in persistent or significant disability/incapacity;

– is a congenital anomaly/birth defect;

– is medically significant or requires intervention to prevent one or other of the outcomes listed above.

**Adverse drug reaction (ADR):** An ADR is any unintended harm or injury associated with the use of given medications at a normal dosage during normal use. In clinical trials of any new drug or any new usage of a certain drug, any unintended harm or injury that has a causal relationship to the use of the drug should also be considered as an ADR.

**7.2 Recording**

For all AEs encountered during the clinical study, the following must be assessed and recorded on the adverse events form of the e-CRF: intensity, relationship to study drug, action taken regarding study drug, and outcome to date. All AEs, especially those for which the relationship to test drug is not “unrelated”, should be followed up until they have returned to baseline status or stabilized. The follow-up procedures could be performed in clinic visits or during hospitalization, or via home visit, telephone or mail, depending on the severity of AEs.

**7.3 Severity of an AE**

**Mild:** events require minimal or no treatment and do not interfere with the subject’s daily activities.

**Moderate:** events result in a low level of inconvenience or concern with the therapeutic measures. Moderate events may cause some interference with functioning.

**Severe:** events interrupt a subject’s usual daily activity and may require systemic drug therapy or other treatment. Severe events are usually incapacitating.

**7.4 Evaluation the correlation between AEs and study drugs**

Based on the current 6-level evaluation standard of the China National Center for Adverse Drug Reaction Monitoring, according to the answers to 5-iterm questionnaire, the correlation between AEs and study drugs can be categorized as to definitely related, probably related, possibly related, unlikely related, to be evaluated, and unable to evaluate (Table 3) . The adverse drug reaction was determined as "definitely related", "probably related", " possibly related".

**Table 3 The assessment of the causality between adverse events and observed drug**

| **Item*** | **Index** | | | | | |
| --- | --- | --- | --- | --- | --- | --- |
|  | **1= definitely related** | **2= probably related** | **3=**  **possibly related** | **4=**  **unlikely related** | **5=**  **to be evaluated** | **6=**  **unable to evaluate** |
| A reasonable timing relationship^1^ | + | + | + | - | Additional detailed information to be evaluated | The necessary information for the evaluation is not available |
| Belonging to the Known ADR of the drug ^2^ | + | + | ± | - |  |  |
| Relief after stopping the drug^3^ | + | + | ±? | ±? |  |  |
| Recurrent AE after re-administration^4^ | + | ? | ? | ? |  |  |
| Suspected adverse reaction could be explained with other certain reasons^5^ | - | - | ±? | ±? |  |  |

*The 5-iterm questionnaire to assess the causality between adverse events and observed drug is listed in the items 1 to 5, detailed as following:

1. Is there a reasonable relationship between the time of starting the drug and the time of occurrence of the suspected adverse reaction?

2. Is the suspected adverse drug reaction consistent with the types of adverse reaction known to the drug?

3.Could the suspected adverse reaction be alleviated or disappeared after stopping the drug or reducing the dosage?

4. Dose the same adverse event occur again after given the same drug again?

5. Could the suspected adverse reaction be explained by the patient's pathological condition, combined medication, combined therapy, and used therapy etc?

**7.5 Handling of SAEs**

All SAEs should be managed properly and timely, and recorded on the appropriate SAE eCRF. Any AE considered serious by the investigator or which meets the aforementioned criteria must be submitted on a signed and dated SAE form to the sponsor, ethics review committee and relevant authorities within 24 hours of site awareness. And the sponsor should also inform all study sites of such SAEs in a timely manner to ensure subjects’ safety in accordance with applicable laws and regulations.

Fatal or life-threatening events considered to be caused by the study drugs must be reported immediately to the sponsor and relevant authorities via telephone, fax, EMS or E-mail.

**7.6 Opening and handling of emergency envelops**

The emergency envelope provided along with each test drug in its package should only be opened in case of a serious complication or a serious adverse event, which is called emergency unblinding. Once unblinded, the patient should be withdrawn from the study and regarded as a dropout case. Information about when, where and why the emergency unblinding occurs as well as each personnel’s signature should also be documented.

**7.7 Follow-up of unresolved AEs**

All AEs should be followed up until they have been resolved, stabilized or returned to baseline status.

8. **DATA COLLECTION AND MANAGEMENT**

**8.1 Establishment of Three Committees**

To ensure the effectiveness and integrity of the trial design, three committees have been established by the multicentre trial coordination group: the clinical trial steering committee, the data monitoring committee (DMC) and the outcome evaluation committee. The first committee is responsible for the study design and the implementation process. The DMC will supervise the integrity and accuracy of data collection to control its quality. The outcome evaluation committee will evaluate key outcomes (including outcome measurements and adverse events) based on their clinical expertise.

**8.2 Data Management**

(1) All data will be recorded by trained clinical investigators using standardized e-CRFs in Brightech clinical information management systems (Brightech, Somerset, NJ, USA).

(2) The accuracy and reliability of the data will be ensured by the study monitor, who will verify and cross-check the e-CRFs against the investigator’s records (source document verification), using the maintenance of a drug dispensing log by the investigator.

(3) Front-end checks in the e-CRFs and back-end checks in the clinical information management system will be used as a validation check of the data.

(4) If there are any discrepancies in the e-CRFs, the results will be sent to the investigator for resolution.

(5)The results of the analysis must not be released with individual identification of the subjects until the database is closed.

**8.3 Data Report**

Data report contains the aspects as followed: (1) members of the project; (2) disagreement from the primary data management plan; (3) actual finish time of every project; (4) problems and the solution during the data management (if have any); (5) reconstruction of the database (if have any); (6) distribution of the participants; (7) participants who disobey the trial protocol; (7) classifying plan of the statistical analysis population. Data report will be performed monthly and then send to DMC since the first entry of the e-CRF.

**8.4 Data Blind Review**

When the data checking is finished, a data blind review meeting will be hold. On the meeting, the data administrators, statisticians, researchers, clinical inspectors, and other related members would have a discussion on the following items according to the data management report and the data lists:

•Distribution of the participants;

•Protocol disobeying or not;

•Possible outlier;

•Baseline data;

•Outcomes;

•Statistical analysis plan.

Participants will be classified to their suitable statistical analysis sets according to the definition in the protocol. No patient can be excluded from the analysis, unless getting the permission of the meeting participants. All the meeting participants should sign the data locking consent, and the data auditing resolution.

**8.5 Database Locking**

The database will be locked if it fulfills all the aspects as followed:

•All the queries have been solved, and the database has been updated;

•No query has been found through the data inspection;

•The medical coding has been completed;

•The plan of the participants’ classification has been approved;

•The final draft of the SAP has been made, and approved by the project leader.

The statisticians and the data administrators will sign the data locking form, and then the database will be locked. The locked database will be sent to the statisticians for further statistical analysis through the data format of SAS.

**9. STATISTICAL CONSIDERATIONS**

**9.1 Study Populations**

All patients with randomization will be included in the analysis set regardless of whether they receive any treatment. Most outcome measurements (except micro-RNA and mRNA profiles) will be analyzed on the intention-to-treat (ITT) and per-protocol population (PPS). Safety analysis will be performed in a safety set, which is defined as a subset of subjects who were randomized and who received at least one treatment.

**9.2 Statistical Analyses**

The primary study hypothesis is that an initial usage of DHI added to routine medical care could bring more clinical benefit for the patients with acute ischemic stroke than an initial strategy of routine medical care alone. Missing data of the primary outcome were handled using the baseline-observation-carried-forward (BOCF) approach, while missing data of secondary outcomes were not imputed. The chi-square test will be used to compare the proportion of patients with a favorable outcome, defined as a score of 0–1 on the mRS at day 90 between the two groups. Pre-specified subgroup analysis will also be performed according to the Statistical Analysis Plan (SAP).

The secondary efficacy and safety outcomes will be analyzed using the two sample t-tests, Wilcoxon rank-sum test, Chi-square test, Poisson regression or Fisher’s exact test according to the Statistical Analysis Plan (SAP).

All reported P values were two-sided; and a P value less than 0.05 was considered to be statistically significant except that from the primary outcome analyses. All calculations were performed using SAS software, version 9.1.3 (SAS Institute, Cary, NC).

**9.3 Statistical Analysis Plan (SAP)**

Prior to database lock and before code breaking, a final version of the SAP shall be issued and approved by the study statistician, and the principal investigator. The SAP will define all “pre-specified, planned analyses” and provide the general specifications for the analysis of the data to be collected and presented in clinical information management systems.

**10 ETHICAL ASPECTS**

The investigator will ensure that this study is conducted in full conformance with the principles of the “Declaration of Helsinki” and applicable laws and regulations on the conduct of clinical trials.

This trial firstly will be approved by center institutional review board (IRB) in Beijing Beijing Tiantan Hospital, Capital Medical University . For the other sites, if need, the trail also should be approved by the local IRB at the certain enrolling sites. Only when the trial protocol is approved by the IRB, the enrollment of participant will begin.

Informed consent is a process that is initiated prior to the individual’s agreeing to participate in the study and continues throughout the individual’s study participation. Extensive discussion of risks and possible benefits of this treatment will be provided to the subjects and their families. Consent forms describing in detail the study interventions/products, study procedures, and risks are given to the subject and written documentation of informed consent is required prior to starting intervention/administering study product. It must also be explained to the patients that they are completely free to refuse to enter the study or to withdraw from it at any time for any reason. A copy of the informed consent document will be given to the subjects for their records.

**REFERENCES**

1. Matsuo R, Kamouchi M, Ago T, Hata J, Shono Y, Kuroda J, et al. Thrombolytic therapy with intravenous recombinant tissue plasminogen activator in Japanese older patients with acute ischemic stroke: the Fukuoka Stroke Registry. Geriatr Gerontol Int. 2014;14:954–9.

2. Chen J, Venkat P, Zacharek A, Chopp M. Neurorestorative therapy for stroke. Front Hum Neurosci. 2014;8:382.

3. Turner RC, Lucke-Wold B, Lucke-Wold N, Elliott AS, Logsdon AF, Rosen CL, et al. Neuroprotection for ischemic stroke: moving past shortcomings and identifying promising directions. Int J Mol Sci. 2013;14:1890–917.

4. Behravan E, Razavi BM, Hosseinzadeh H. Review of plants and their constituents in the therapy of cerebral ischemia. Phytother Res. 2014; 28:1265–74.

5. Li X, Tang J, Meng F, Li C, Xie Y. Study on 10 409 cases of post-marketing safety Danhong injection centralized monitoring of hospital. Zhong guo Zhong Yao Za Zhi. 2011;36:2783–5.

6. He Y, Wan H, Du Y, Bie X, Zhao T, Fu W, et al. Protective effect of Danhong injection on cerebral ischemia-reperfusion injury in rats. J Ethnopharmacol. 2012;144:387-94.

7. Xiujuan Mi, Guangqin Li. Effect of Danhong on Neural Functional Recovery after Focal Cerebral Ischemia in Rats and Its Mechanism. Zhongguo Quanke Yixue. 2010;13:870-3.

8. He Y, Wan H, Du Y, Bie X, Zhao T, Fu W, et al. Protective effect of Danhong injection on cerebral ischemia-reperfusion injury in rats. J Ethnopharmacol. 2012;144:387–94.

9. Ma L, Wang S, Li K. A systematic review of Danhong injection treatment for acute cerebral ischemic stroke and the safety of this treatment. Journal of Emergency in Traditional Chinese Medicine. 2012;10:1609–10.

10.Shuaib A, Lees KR, Lyden P, Grotta J, Davalos A, Davis SM, et al. NXY-059 for the treatment of acute ischemic stroke. N Engl J Med. 2007;357:562–71.

11. Hacke W, Kaste M, Bluhmki E, Brozman M, Davalos A, Guidetti D, et al. Thrombolysis with alteplase 3 to 4.5 hours after acute ischemic stroke. N Engl J Med. 2008;359:1317–29.

12. Chinese Society of Neurology. China Guideline for the Diagnosis and Treatment of Acute Ischemic Stroke. Chin J Neurol. 2010;43:146–53.

13.Barber PA, Demchuk AM, Zhang J, Buchan AM, Group AS. Validity and reliability of a quantitative computed tomography score in predicting outcome of hyperacute stroke before thrombolytic therapy. ASPECTS Study Group. Alberta Stroke Programme Early CT Score. Lancet 2000; 355: 1670-7

14. Zheng X. Guidelines of Clinical Research of New Drugs of Traditional Chinese Medicine. Beijing: China Medical Science Press; 2002.

15. Brain disease emergency cooperation group of State Administration of traditional Chinese Medicine, Criteria For Diagnosis And Curative Effect Evaluation Of Stroke, 1996,19(1):55-56

16. Ying Gao, Bin Ma, Qiang Liu, Yongyan Wang. Development and methodology discussion of the diagnostic scale of ischemic stroke syndrome elements. Chinese Journal of Traditional Chinese Medicine. 2011;52: 2097-101.

17. van Swieten JC, Koudstaal PJ, Visser MC, Schouten HJ, van Gijn J. Interobserver agreement for the assessment of handicap in stroke patients. Stroke. 1988;19:604-7.

18. Mahoney FI, Barthel DW. Functional evaluation: the Barthel Index. Md State Med J. 1965;14:61–5.

19. Wityk RJ, Pessin MS, Kaplan RF, Caplan LR. Serial assessment of acute stroke using the NIH Stroke Scale. Stroke. 1994;25:362–5.

20. Brooks R. EuroQol: the current state of play. Health Policy. 1996;37:53–72.

21. Mehran R, Rao SV, Bhatt DL, Gibson CM, Caixeta A, Eikelboom J, et al. Standardized bleeding definitions for cardiovascular clinical trials: a consensus report from the Bleeding Academic Research Consortium.Circulation. 2011;123:2736-2747.

**Protocol (Final)**

**The Final Version: February 18, 2016**

**Danhong Injection for the treatment of acute ischemic stroke (Phase IV): an adaptive-design, randomized, multi-center, double-blind, placebo-controlled clinical trial**

**Sponsored by:** China National Science and Technology Major Project for "Significant New Drugs Development" (2011ZX09304-07)

**CLINICAL TRIAL STEERING COMMITTEE**

**Prof. Yong-Yan Wang (Chair),** Institute of Basic Research in Clinical Medicine, China Academy of Chinese Medical Sciences

**Prof. Xiao-Xi Du (Member),** China Food and Drug Administration

**Prof. Bo-Li Zhang (Member)**, China Academy of Chinese Medical Sciences

**Prof. Run-Lin Gao (Member)**, Fuwai Hospital Chinese Academy of Medical Sciences

**Prof. Wei-Liang Weng (Member)**, Xi-yuan Hospital Affiliated to China Academy of Chinese Medical Sciences

**Dr. Ai-Ping Lv (Member)**, 1. Institute of Basic Research in Clinical Medicine, China Academy of Chinese Medical Sciences; 2. School of Chinese Medicine, Hong Kong Baptist University

**Prof. Ming-Hui Yang (Member)**, Chinese PLA General Hospital

**PRINCIPLE INVESTIGATORS**

**Prof. Zhong Wang, Ph.D (for grant)**

Institute of Basic Research in Clinical Medicine, China Academy of Chinese Medical Sciences

No.16 Nanxiaojie, Dongzhimennei, Beijing, 100700, China.

Tel:86-10-64093305

Email: zhonw@vip.sina.com

**Prof. Xingquan Zhao, M.D. (for trial)**

Department of Neurology, Beijing Tiantan Hospital, Capital Medical University

Tiantan xili 6, Dongcheng, Beijing, 100050, China.

Email: zxq@vip.163.com

**PARTICIPATING SITES AND INVESTIGATOR**

**1. Beijing Tiantan Hospital, Capital Medical University**

**Prof. Xing-quan Zhao, M.D. (for trial)**

Site Principal Investigator,

Department of Neurology,

Beijing Tiantan Hospital, Capital Medical University.

Tiantan xili 6, Dongcheng, Beijing, 100050, China.

Email: zxq@vip.163.com.

2. **Peking University First Hospital (Terminated)**

**Prof. Xuezhi Zhang, M.D.**

Site Principal Investigator,

Department of Traditional Chinese Medicine,

Peking University First Hospital,

No. 8 nishiku Street, Beijing, 100034, China.

Email: zhang.xuezhi@263.net.

3. **The Second Artillery General Hospital of Chinese People's Liberation Army**

**Prof. Lei Wang, M.D.**

Site Principal Investigator,

Department of Neurology,

The Second Artillery General Hospital of Chinese People's Liberation Army

No. 16 Xinjiekou Wai Street, Beijing, 100088, China

Email: hellowanglei068@163.com

4. **Peking University of People's Hospital (Withdrawn)**

**Prof. Xu-guang Gao, M.D.**

Site Principal Investigator,

Department of Neurology,

Peking University of People's Hospital

No.11 South Avenue Street, Beijing, 100044, China

Email: gxg56@tom.com.

5. **Xuan Wu Hospital Affiliated to Capital Medical University**

**Prof. Li Gao.**

Site Principal Investigator, Department of Neurology

Xuan Wu Hospital Affiliated to Capital Medical University

No.45 Chang Chun Street, Beijing, 100053, China

Email: xuanwugaoli@126.com

6. **The First Affiliated Hospital of Henan University of T.C.M.**

**Prof. Xinzhi Wang**

Site Principal Investigator,

Department of Neurology,

The First Affiliated Hospital of Henan University of T.C.M.

No. 19, Renmin Road, Zhengzhou, 450000, Henan, China

Email: [znqznq@163.com](mailto:znqznq@163.com)

**7. Xiangya Hospital, Central South University**

**Prof. Xiaosu Yang, M.D.**

Site Principal Investigator, Department of Neurology

Xiangya Hospital Central-south University

No. 87, Xiangya Road, Changsha, 410008, Hunan, China,

Email: [sjnk_xy@yahoo.com.cn](mailto:sjnk_xy@yahoo.com.cn)

**8. Renmin Hospital, Wuhan University**

**Prof. Zhaohui Zhang, M.D**

Site Principal Investigator

Department of Neurology,

Renmin Hospital of Wuhan University,

No. 238, Jiefang Road, Wuhan, 430060, Hubei, China.

Email: zhzhqing1990@163.com

**9. Zhongnan Hospital, Wuhan University**

**Prof. Yumin Liu, M.D**

Site Principal Investigator, Department of Neurology

Zhongnan Hospital of Wuhan University

No. 169, Donghu Road, Wuhan, 430030, Hubei, China.

Email: lym9381@123.com

**10. Affiliated Zhongshan Hospital of Dalian University**

**Prof. Qiang Ma**

Site Principal Investigator

Department of Neurology

Affiliated Zhongshan Hospital of Dalian University

No. 6, Jiefang Street, Dalian, 116001, Liaoning, China.

Email: 13332258950@163.com

**11. The Second Hospital of Jilin University (Terminated)**

**Prof. Tingmin Yu,** **M.D.**

Site Principal Investigator

Department of Neurology

The Second Hospital of Jilin University

No. 218, Ziqiang Street, Changchun, 130041, Jilin, China.

Email: ytm396@163.com

**12. First Teaching Hospital of Tianjin University of Traditional Chinese Medicine**

**Prof. Zhihong Meng, M.D.**

Site Principal Investigator

Department of Neurology

First Teaching Hospital of Tianjin University of Traditional Chinese Medicine

No. 88, Changling Road, Tianjin, 300193, China,.

Email: jy1988mzh@163.com

**13. Affiliated Hospital of Shanxi University of Chinese Medicine**

**Prof. Yongmei Yan.**

Site Principal Investigator

Department of Neurology

Affiliated Hospital of Shanxi University of Chinese Medicine

No.75, Section 1, Jinci Road, Xianyang, 712000, Shanxi, China.

Email: 13609216551@173.com

**14. The Second Affiliated Hospital to Shanxi Medical University**

**Prof. Guanglai Li.**

Site Principal Investigator

Department of Neurology

The Second Affiliated Hospital to Shanxi Medical University

No. 382, Wuyi Road, Taiyuan, 030001, Shanxi, China.

Email: smuneu@163.com

**15. Changsha Central Hospital**

**Prof. Zhuqin Liu**

Site Principal Investigator

Department of Neurology

Changsha Central Hospital

No. 161, South Shaoshan Road, Changsha, 410004, Hunan, China,.

Email: zhuqin_630@126.com

**16. Nanjing Hospital of TCM. (Terminated)**

**Prof. Shuping Cao**

Site Principal Investigator

Department of Neurology

Nanjing Hospital of T.C.M.

No. 157 Daming Road, Nanjing, 210001, Jiangsu, China.

Email: njcaoshuping@126.com

**17. Shanxi Provincial People's Hospital**

**Prof. Qian Yang.**

Site Principal Investigator

Department of Neurology

Shanxi Provincial People's Hospital.

No. 29 shuangtasi Street, Xi'an, 710068, Shanxi, China.

Email: yangqdoctor@163.com

**18. Shanxi Province Hospital of T.C.M (Terminated)**

**Prof. Zucheng Han.**

Site Principal Investigator, Department of Neurology

Shanxi Province Hospital of T.C.M.

No. 16, Bingzhou West Street, Xi'an, 710003, Shanxi, China.

Email: 13759968645@139.com

**19. The PLA 451 Hospital**

**Prof. Liyi Chi, M.D.**

Site Principal Investigator

Department of Neurology

The PLA 451 Hospital

No. 269, Youyi East Road, Xi'an, 710054, Shanxi, China.

Email: [1173319088@qq.com](mailto:1173319088@qq.com)

**20. Xin Hua Hospital of Zhejiang Province**

**Prof. Bin Xu.**

Site Principal Investigator

Department of Neurology

Xin Hua Hospital of Zhejiang Province.

No. 318, Chaowang Road, Hangzhou, 310005, Zhejiang, China.

Email: xubin2008.love@163.com

**21. Nanjing Drum Tower Hospital (Terminated)**

**Prof. Yun Xu, M.D.**

Site Principal Investigator

Department of Neurology

Nanjing Drum Tower Hospital.

No. 321, Zhongshan Road, Nanjing, 210008, Jiangsu, China.

Email: [xuyunz@hotmail.com](mailto:xuyunz@hotmail.com)

**22. The First Affiliated Hospital of College of Medicine, Zhejiang University (Withdrawn)**

**Prof. Benyan Luo, M.D.**

Site Principal Investigator

Department of Neurology

The First Affiliated Hospital of College of Medicine, Zhejiang University.

No. 79, Qingchun Road, Hangzhou, 310003, Zhejiang, China.

Email: [luobenyan@zju.edu.cn](mailto:luobenyan@zju.edu.cn)

**23. The Second Affiliated Hospital of Nanjing Medical University**

**Prof. Zhiqiang Gao.**

Site Principal Investigator

Department of Neurology

The Second Affiliated Hospital of Nanjing Medical University.

No. 121, jiangjiayuan, Nanjing, 210003, Jiangsu, China.

Email: 13952000693@139.com

**24. The First Hospital of Changsha**

**Prof. Aimin Wang.**

Site Principal Investigator

Department of Neurology

The First Hospital of Changsha.

No. 311, Yingpan Road, Changsha, 410005, Hunan, China.

Email: 13873188716@163.com

**25. Xuhui District Central Hospital of Shanghai City (Withdrawn)**

**Prof. Hui Zhang.**

Site Principal Investigator

Department of Neurology

Xuhui District Central Hospital of Shanghai City.

No. 966, Huaihai Middle Road, Shanghai, 200031, China.

Email: [zhanghui1956@263.net](mailto:zhanghui1956@263.net)

**26. Teaching Hospital of Chengdu University of Traditional Chinese Medicine**

**Prof. Yun Lu, M.D.**

Site Principal Investigator

Emergency Department

Teaching Hospital of Chengdu University of Traditional Chinese Medicine

No. 39, Shierqiao Road, Chengdu, 610072, Sichuan, China.

Email: [luyun999@126.com](mailto:luyun999@126.com)

**27. Zhejiang Provincial People's Hospital**

**Prof. Yu Geng, M.D.**

Site Principal Investigator

Department of Neurology

Zhejiang Provincial People's Hospital.

No. 158, Shangtang Road, Hangzhou, 310014, Zhejiang, China.

Email: [gengy2004@126.com](mailto:gengy2004@126.com)

**28. Zhejiang Provincial Hospital of T.C.M.**

**Prof. Qun Hou.**

Site Principal Investigator, Department of Neurology

Zhejiang Provincial Hospital of T.C.M.

No. 54, Youdian Road, Hangzhou, 310006, Zhejiang, China.

Email: [houqun168@163.com](mailto:houqun168@163.com)

**29. The Sixth People's Hospital of Shanghai City (Withdrawn)**

**Prof. Qing-ping Huo.**

Site Principal Investigator, Department of Neurology

The Sixth People's Hospital of Shanghai City.

No. 600, Yishan Road, Shanghai, 200233, China.

**30. Hubei Provincial Hospital of T.C.M. (Withdrawn)**

**Prof. Yanbing Ding, M.D.**

Site Principal Investigator, Department of Neurology

Hubei Provincial Hospital of T.C.M.

No. 4, Huayuanshan, Wuhan, 430061, Hubei, China.

**31. The Second People's Hospital of Hunan Province /Brain Hospital of Hunan Province**

**Prof. Qiu-ting Liu.**

Site Principal Investigator

Department of Neurology

The second people's hospital of Hunan Province.

No. 427, section 3, Furong Middle Road, Changsha, 410007, Hunan, China.

Email: 13974809911@163.com

**32. Chongqing Three Gorges Central Hospital**

**Prof. Wei Xue**

Site Principal Investigator

Department of Neurology

Chongqing Three Gorges Central Hospital.

No.165, Xincheng Road, Chongqing, 404000, China.

Email: 13706766228@qq.com

**33. Xinqiao Hospital of Third Military Medical University**

**Prof. Qingwu Yang, M.D.**

Site Principal Investigator

Department of Neurology

Xinqiao Hospital of Third Military Medical University.

No. 83, Xinqiao Main Street, Chongqing, 400037, China.

Email: yangqwmlys@163.com

34. **The First Hospital Affiliated to Jilin University**

**Prof. Jiafeng Chen, M.D.**

Site Principal Investigator, Department of Neurology

The First Hospital Affiliated to Jilin University.

No. 71, Xinmin Street, Changchun, 130021, Jilin, China.

Email:1477478558@qq.com

**35. Jilin Province People's Hospital**

**Prof. Hongxia Sun.**

Site Principal Investigator

Department of Neurology

Jilin Province People's Hospital.

No. 1183, Gongnong Road, Changchun, 130021, Jilin, China.

Email: 984897342@qq.com

**36. Zhongshan Hospital of Hubei Province (The Third People's Hospital of Hubei Province)**

**Prof. Xiaorong Deng**

Site Principal Investigator

Department of Neurology

Zhongshan Hospital of Hubei Province.

No. 26 Zhongshan Avenue, Wuhan, 430033, Hubei, China.

Email: [xiaorong9596@aliyun.com](mailto:xiaorong9596@aliyun.com)

**37. The Affiliated Hopital to Changchun University of Chinese Medicine (Terminated)**

**Prof. Jianjun Zhao.**

Site Principal Investigator

Department of Neurology

The Affiliated Hopital to Changchun University of Chinese Medicine

No. 1478, Gongnong Road, Changchun, 130021, Jilin, China.

**38. China-Japan Union Hospital of Jilin University**

**Prof. Zhongxin Xu, M.D.**

Site Principal Investigator

Department of Neurology

China-Japan Union Hospital of Jilin University

No. 126, Sendai Street, Changchun, 130033, Jilin, China.

Email: xuzhongxin999@aliyn.com

**39. People's Liberation Army No.202 Hospital**

**Prof. Zhilin Jiang**

Site Principal Investigator

Department of Neurology

People's Liberation Army No.202 Hospital

No. 5, Guangrong Street, Shenyang, 110003, Liaoning, China.

Email: 53176102@qq.com

**40. People's Liberation Army No.463 Hospital**

**Prof. Yansong Li**

Site Principal Investigator

Department of Neurology

People's Liberation Army No.463 Hospital

No. 46, Xiaoheyan Road, Shenyang, 110046, Liaoning, China.

Email: 28837893@qq.com

**41. People's Hospital of Zhengzhou**

**Prof. Laihui Feng.**

Site Principal Investigator

Department of Neurology

People's Hospital of Zhengzhou

No. 33, Huanghe Road, Zhengzhou, 450003, Henan, China.

Email: fch630@163.com

**42. The 7th People's Hospital of Zhengzhou**

**Prof. Junxing Dong.**

Site Principal Investigator

Department of Neurology

The 7th People's Hospital of Zhengzhou

No. 17, Jingnan 5th Road, Zhengzhou, 450006, Henan, China.

Email:15939026596@139.com

**43. The Central Hospital of Wuhan**

**Prof. Linhong Zhang.**

Site Principal Investigator

Department of Neurology

The Central Hospital of Wuhan

No. 26, Shengli Street, Wuhan, 430014, Hubei, China.

Email: zhanglinhong05@sina.com

**44. The Second Affiliated Hospital of Liaoning University of Traditional Chinese (Withdrawn)**

**Prof. Fuying Jiao.**

Site Principal Investigator, Department of Neurology

The Second Affiliated Hospital of Liaoning University of Traditional Chinese

No. 60, Huanghe North Street, Shenyang, 110034, Liaoning, China.

**45. Guangdong Second Provincial General Hospital**

**Prof. Xintong Liu, M.D.**

Site Principal Investigator

Department of Neurology

Guangdong Second Provincial General Hospital

No. 466, Xingang Middle Road, Guangzhou, 510317, Guangdong, China.

Email: xintongliu@163.com

**46. Fifth Hospital in Wuhan**

**Prof. Ming Li.**

Site Principal Investigator

Department of Neurology

Fifth Hospital in Wuhan

No. 122, Xianzheng Street, Wuhan, 430050, Hubei, China.

Email: 18602702599@163.com

**47. Affiliated Hospital of Chengde Medical University**

**Prof. Yanjun Gao.**

Site Principal Investigator

Department of Neurology

Affiliated Hospital of Chengde Medical University

No. 36, Nanyingzi Street, Chengde, 067000, Hebei, China.

Email: yanjungao2009@126.com

**48. Affiliated Hospital of Chifeng University**

**Prof. Weili Zhao.**

Site Principal Investigator

Department of Neurology

Affiliated Hospital of Chifeng University

No. 42, Wangfu Street, Chifeng, 024005, Neimenggu, China.

Email: wlzhao1968@163.com

**49. The Second Affiliated Hospital of Shanxi University of T.C.M.**

**Prof. Jumei Du.**

Site Principal Investigator

Department of Neurology

The Second Affiliated Hospital of Shanxi University of T.C.M.

No. 5, Weiyang West Road, Xianyang, 712000, Shanxi, China.

Email: 15091806663@126.com

**50. Fenyang Hospital of Shanxi Province**

**Prof. Bao’ai Wang.**

Site Principal Investigator

Department of Neurology

Fenyang Hospital of Shanxi Province.

No. 186, Shengli Street, Luliang, 032299, Shanxi, China.

Email: wbaoai@tom.com

**51. Tianyou Hospital Affiliated to Wuhan University of Science & Technology**

**Prof. Jun Peng.**

Site Principal Investigator

Department of Neurology

Tianyou Hospital Affiliated to Wuhan University of Science & Technology.

No. 186, Shengli Street, Wuhan, 430064, Hubei, China.

Email: pengjun63812@sina.com

**52. Panjin Central Hospital**

**Prof. Yanhua Zhou.**

Site Principal Investigator

Department of Neurology

Panjin Central Hospital.

No. 32, Liaohe Middle Road, Panjin, 124013, Liaoning, China.

Email: pjzyh111@163.com

**53. Guangzhou hospital of integrated Chinese and Western medicine**

**Prof. Chaojun Chen, M.D.**

Site Principal Investigator

Department of Neurology

Guangzhou hospital of integrated Chinese and Western medicine.

No. 87, Yingbin Avenue, Guangzhou, 510813, Guangdong, China.

Email: ccjbs@126.com

**54. Anshan Central Hospital**

**Prof. Zhen Jiao.**

Site Principal Investigator

Department of Neurology

Anshan Central Hospital.

No. 77, South China Road, Anshan, 114001, Liaoning, China.

Email: asjiaozhen@163.com

**55. Handan Central Hospital**

**Prof. Juntao Li.**

Site Principal Investigator

Department of Neurology

Handan Central Hospital.

No. 15, Zhonghua South Street, Handan, 056001, Hebei, China.

Email: 504422405@qq.com

56. **The Second Affiliated Hospital of Xingtai Medical College (Withdrawn)**

**Prof. Fengqing Gao.**

Site Principal Investigator

Department of Neurology

The Second Affiliated Hospital of Xingtai Medical College.

No. 1628, Quanbei West Street, Xingtai, 054099, Hebei, China.

**57. Huzhou Central Hospital (Terminated)**

**Prof. Yaxian Wang.**

Site Principal Investigator

Department of Neurology

Huzhou Central Hospital.

No. 198, Hongqi Road, Huzhou, 313003, Zhejiang, China.

Email: zxwyx022@163.com

**58. Liaoyuan Traditional Chinese Medical Hospital**

**Prof. Hongwei Dai.**

Site Principal Investigator

Department of Neurology

Liaoyuan Traditional Chinese Medical Hospital.

No. 616, Kangning street, Liaoyuan, 136299, Jilin, China.

Email: jLLYYHY@126.com

**59. The General Hospital of Shenyang Military**

**Prof. Huisheng Chen, M.D.**

Site Principal Investigator

Department of Neurology

The General Hospital of Shenyang Military.

No. 83 Wenhua Road, Shenyang, 110016, Liaoning, China.

Email: chszh@aliyun.com

**60. The Second Central Hospital of Baoding City**

**Prof. Shuangxia Du.**

Site Principal Investigator

Department of Neurology

The Second Central Hospital of Baoding City.

No. 57, Fanyang Middle Road, Baoding, 072750, Hebei, China.

Email: 1720481082@qq.com

**61. Tianjin Medical University General Hospital (Withdrawn)**

**Prof. Fudong Shi, M.D.**

Site Principal Investigator

Department of Neurology

Tianjin Medical University General Hospital.

No. 154, Anshan Road, Tianjin, 300052, China.

**62. The First Affiliated Hopital of Chengdu Medical College**

**Prof. Jie Yang, M.D..**

Site Principal Investigator

Department of Neurology

The First Affiliated Hopital of Chengdu Medical College.

No. 278, middle section of Baoguang Avenue, Chengdu, 610500, Sichuan, China.

Email: yangjie1126@163.com

**63. Yanbian University Hospital (Yanbian Hospital) (Withdrawn)**

**Prof. Chao Liu.**

Site Principal Investigator

Department of Neurology

Yanbian University Hospital (Yanbian Hospital).

No. 1327, Juzi Street, Yanji, 133099, Jilin, China.

**64. Huangpi District Hospital of T.C.M.**

**Prof. Junhua He.**

Site Principal Investigator

Department of Neurology

Huangpi District Hospital of T.C.M.

No. 48, Banqiao Avenue, Wuhan, 430399, Hubei, China.

Email: 632854751@qq.com

**65. The Second Affiliated Hopital of Harbin Medical University**

**Prof. Lihua Wang, M.D.**

Site Principal Investigator

Department of Neurology

The Second Affiliated Hopital of Harbin Medical University.

No. 246, Xuefu Road, Harbin, 150001, Helongjiang, China.

Email: wanglh211@163.com

**66. Dandong First Hospital**

**Prof. Lishu Wan.**

Site Principal Investigator

Department of Neurology

Dandong First Hospital.

No. 76, Baoshan Street, Dandong, 118015, Liaoning, China.

Email: wanwan0409@163.com

**67. The Second Chinese Medical Hospital of Luoyang City**

**Prof. Xuewen Huo.**

Site Principal Investigator

Department of Neurology

The Second Chinese Medical Hospital of Luoyang City.

No. Jiudu West Road, Luoyang, 471003, Henan, China.

Email: 419088841@qq.com

**68. The People's Hospital of Daqing City (Withdrawn)**

**Prof. De-xin Luo.**

Site Principal Investigator

Department of Neurology

The People's Hospital of Daqing City.

No. 213, Jianshe Road, Daqing, 163316, Heilongjiang, China.

**69. Nanyang T.C.M. Hospital**

**Prof. Zengxiang Li.**

Site Principal Investigator

Department of Neurology

Nanyang T.C.M. Hospital.

No. 939, Qiyi Road, Nanyang, 473007, Henan, China.

Email: 653140416@qq.com

**70. Zhoukou Hospital of T.C.M.**

**Prof. Yanxia Zhang.**

Site Principal Investigator

Department of Neurology

Zhoukou Hospital of T.C.M.

No. 33, Qiyi West Road, Zhoukou, 466099, Henan, China.

Email: 2795853393@qq.com

**71. The Second People's Hospital of Shenzhen**

**Prof. Xiaohua Xiao, M.D.**

Site Principal Investigator

Department of Neurology

The Second People's Hospital of Shenzhen

Sungang Road, Shenzhen, 518028, Guangdong, China.

Email: tu_xi8888@163.com

**72. Nanshi Hospital of Nanyang**

**Prof. Yukun Yang.**

Site Principal Investigator

Department of Neurology

Nanshi Hospital of Nanyang

No. 130, Zhongzhou West Road, Nanyang, 473065, Henan, China.

Email: 1126235541@qq.com

**DATA MONITORING COMMITTEE (DMC)**

Prof. Chun-Ti Shen (Chair), Changzhou TCM Hospital Affiliated to Nanjing University of Chinese Medicine

Prof. Qi-Guang Chen (Member, independent statistician), Southeast University

Prof. Hua-Qiang Zhang (Member), Jiangsu Provincial Commission of Health and Family Planning

Dr. Hong-Wu Wang (Member), Tianjin University of Traditional Chinese Medicine

Dr. Xiao-Chun Sun (Member), Beijing Kelunrui Pharmaceutical Technology Co. Ltd.

Prof. Cheng-Shu Wang (Member), Zhongda Hospital Southeast University

Dr. Hai-Xia Dang (Member), China Academy of Chinese Medical Sciences

Dr. Jun Liu (Member), Institute of Basic Research in Clinical Medicine, China Academy of Chinese Medical Sciences

**PROTOCOL STATISTICIANS**

**Prof. Bi-Wei Chen, Ph.D.,** School of Public Health, Southeast University

Dijia Qiao 87, Nanjing,210009, Jiangsu,China

E-mail: drchenbw@126.com

**TABLE OF** **CONTENTS**

[EXECUTIVE COMMITTEE CHAIR	I](#_Toc21341)

[PRINCIPLE INVESTIGATORS	I](#_Toc12397)

[PARTICIPATING SITES AND INVESTIGATOR	II](#_Toc16352)

[DATA MONITORING COMMITTEE (DMC)	XXVIII](#_Toc273)

[PROTOCOL STATISTICIANS	XXVIII](#_Toc1171)

[PROTOCOL SYNOPSIS	1](#_Toc9155)

[1. BACKGROUND INFORMATION	4](#_Toc3132)

[2. OBJECTIVES	5](#_Toc30499)

[3. STUDY DESIGN	5](#_Toc9775)

[3.1 Overview	5](#_Toc29621)

[3.2 Sample size	5](#_Toc15698)

[3.3 Randomization	7](#_Toc17244)

[3.4 Blinding	7](#_Toc23627)

[3.5 Interim analysis	8](#_Toc21834)

[4．SUBJECTS	8](#_Toc4125)

[4.1 Diagnostic criteria	8](#_Toc23203)

[4.2 Inclusion Criteria	10](#_Toc14646)

[4.3 Exclusion Criteria	11](#_Toc21388)

[4.4 Withdrawal/dropout criteria	13](#_Toc11508)

[4.5 Criteria for study termination	14](#_Toc31028)

[4.6 Criteria for subject discontinuation	14](#_Toc22017)

[4.7 Trial flow Chart	15](#_Toc23070)

[5 INTERVENTION	16](#_Toc8359)

[5.1 Routine medical care	16](#_Toc11552)

[5.2 Treatment regimens	16](#_Toc7526)

[5.3 Duration and follow-up	16](#_Toc10671)

[5.4 Concomitant medications	16](#_Toc21237)

[6 OUTCOMES MEASUREMENTS	17](#_Toc31859)

[6.1 Primary outcome	17](#_Toc8442)

[6.2 Secondary Outcomes	17](#_Toc23954)

[6.3 Other Pre-specified Efficacy Outcomes	20](#_Toc16824)

[6.4 Safety Outcomes	20](#_Toc15915)

[6.5 Study Schema	20](#_Toc10766)

[7 ADVERSE EVENTS	22](#_Toc28383)

[7.1 Definition	22](#_Toc17010)

[7.2 Recording	23](#_Toc28499)

[7.3 Severity of an AE	23](#_Toc2261)

[7.4 Evaluation the correlation between AEs and study drugs	24](#_Toc22286)

[7.5 Handling of SAEs	25](#_Toc27281)

[7.6 Opening and handling of emergency envelops	26](#_Toc2609)

[7.7 Follow-up of unresolved AEs	26](#_Toc24266)

[8. DATA COLLECTION AND MANAGEMENT	26](#_Toc16581)

[8.1 Establishment of Three Committees	26](#_Toc19697)

[8.2 Data Management	27](#_Toc24000)

[8.3 Data Report	27](#_Toc26700)

[8.4 Data Blind Review	28](#_Toc15087)

[8.5 Database Locking	28](#_Toc12451)

[9. STATISTICAL CONSIDERATIONS	29](#_Toc31073)

[9.1 Study Populations	29](#_Toc2443)

[9.2 Statistical Analyses	29](#_Toc8718)

[9.3 Statistical Analysis Plan (SAP)	30](#_Toc9453)

[10 ETHICAL ASPECTS	30](#_Toc205)

[REFERENCES	31](#_Toc8872)

**PROTOCOL SYNOPSIS**

TITLE:

Danhong Injection for the treatment of acute ischemic stroke (Phase IV): an adaptive-design, randomized, multi-center, double-blind, placebo-controlled clinical trial.

DESIGN:

A multi-center, randomized, placebo-controlled, double-blind, adaptive clinical trial (superiority design)

NUMBER OF SITES:

72 sites

SAMPLE SIZE:

A total of 846 study participants with acute ischemic stroke with “Xueyu-Zheng” (Blood Stasis Syndrome) were planned initially. And the sample size was enlarged to 1513 by the DMC based on the results of the second interim analyses.

POPULATION:

Patients aged 18 to 70 years who suffered from acute ischemic stroke with cerebral anterior circulation obstruction with a clinical diagnosis of “Xueyu-Zheng” (Blood Stasis Syndrome), 4≤NIHSS score＜20 were eligible for inclusion in the study.

RANDOMIZATION：

Randomization to Danhong Injection (DHI) group or control group (0.9% normal saline as placebo) will be in a 2:1 ratio.

REGIMEN:

Routine medical care (RMC) was provided to all patients according to the China Guideline for the Diagnosis and Treatment of Acute Ischemic Stroke, including control of temperature, blood pressure, lipid and glucose, improving cerebral blood circulation, anti-platelet treatment, anticoagulants, neuroprotective drugs, stroke rehabilitation, and stroke prevention care.

DHI group: RMC + DHI (40 ml, qd) plus 0.9% normal saline (250 ml, ivgtt, qd)

Control group: RMC + placebo of DHI (0.9% normal saline, 40ml, qd) plus 0.9% normal saline (250 ml, ivgtt, qd)

TREATMENT DURATION:

14 days

FOLLOWING UP

76 days after treatment

OUTCOME MEASURES:

Efficacy:

Primary outcome:

The proportion of patients with excellent functional outcome, defined as a score of 0–1 on the Modified Rankin Scale (mRS) at day 90.

Secondary outcomes:

(1). The proportion of patients with a significant improvement in “Xueyu Zheng”, defined as at least 70% reduction in the Efficacy Evaluation Scale of Chinese Medicine Symptom in “Xueyu Zheng”;

(2). The proportion of patients with the Barthel Index (BI) score of ≥90;

(3) The proportion of patients with an improvement in the NIHSS score of ≥4 or the NIHSS score of 0-1;

(4) The quality of life measured by the EQ-5D scale;

(5) Global disability (mRS: 3-5) at 90 days;

(6) Incidence of new-onset major vascular events within 90 days;

Other pre-specified efficacy endpoints are:

(1) The changes on the profiles of micro-RNA in 96 patients selected from certain centers；

(2) The changes on the expression profiles of mRNA in 96 patients selected from certain centers.

Safety:

(1) Overall mortality within 90 days;

(2) Incidence of severe haemorrhages according to the GUSTO bleeding criteria within 90 days;

(3) Incidence of moderate haemorrhages according to the GUSTO bleeding criteria within 90 days;

(4) Incidence of adverse and serious adverse events.

1. **BACKGROUND INFORMATION**

Acute ischemic stroke (AIS), characterized by an occlusion of the cerebral artery, is one of the leading causes of mortality, long-term disability, and morbidity. Thrombolytic therapy with intravenous tissue plasminogen activator (tPA), which remains the only therapeutic drug for AIS approved by the US Food and Drug Administration, is limited by the narrow time window of thrombolysis, bleeding complications, or high costs^1^. Other treatment strategies mainly utilize therapeutic agents to prevent or reduce cell damage from ischemia^2^. Hundreds of neuro protective agents for ischemic stroke have reached clinical trials, but no optimal drugs for the clinical treatment of AIS have yet been developed^3^. This leads us to search for novel therapeutic approaches, and to investigate whether natural compounds extracted from herbs may be a novel option to improve treatment for AIS^4^.

Danhong injection (DHI), extracted from Radix Salviae miltiorrhizae (Danshen) and Flos Carthami tinctorii (Honghua), is a Chinese medicinal product approved by the China Food and Drug Administration (CFDA), and it is widely used in China for treating AIS^5^. Previous studies demonstrated the neuroprotective efficacy of DHI in rat models with cerebral ischemic-reperfusion injury^6,7^, and that the underlying pharmacological mechanisms might involve anti-inflammatory, anti-coagulant, antithrombotic, antifibrinolytic, and antioxidant activities^8^. A number of clinical studies also supported the efficacy and safety of DHI in the management of AIS, but due to the methodological deficiencies of inadequate randomization, no double blinding, no placebo control, or incomplete outcome data, the quality of these studies was assessed as generally low^9^. There is a lack of critically appraised evidence such as well designed randomized controlled trials (RCTs) to justify its clinical use and recommendation. Thus, we aim to investigate the efficacy and safety of DHI in the treatment of AIS in a well-designed, randomized, multicenter, double-blind and placebo-controlled trial.

**2. OBJECTIVES**

The objective of this study is mainly to determine whether Danhong injection is effective in the treatment of acute ischemic stroke.

**3. STUDY DESIGN**

**3.1 Overview**

This study is a three-stage adaptive-design, randomized, multicenter, double-blind, placebo-controlled trial. The number of subjects is initially estimated to be 846 and randomly assigned to be 564 in DHI group and 282 in the control group. After Both groups received routine medical care as recent guideline advocated. Participants in the DHI group are treated by DHI (40 ml, qd) plus 0.9% normal saline (250 ml, ivgtt, qd), while participants in the control group are treated by DHI placebo (0.9% normal saline, 40ml, qd) plus 0.9% normal saline (250 ml, ivgtt, qd).

**3.2 Sample size**

**3.2.1 Initial estimation of sample size**

The proportion of patients who had a favorable outcome, defined as a modified Rankin Scale (mRS) score of 0-1, afterroutine medical care coupled with placebo or DHI for 90 days was unclear. According to previous studies^10, 11^, the proportion of patients who had a mRS score of 0-1 was about 28.7-45.2 % when treated with placebo, so we estimate the proportion is 45% when routine medical care with placebo are used. In this trial, it is hypothesized that an increase of at least 10 % is of clinical significance for the DHI group. This is an adaptive-designed trial with 3-stage, and using EAST5.2, the number of subjects was initially estimated to be 703 (two-sided test, α =0.05, β =0.2). To allow for a dropout rate of 20 %, a total of 846 patients will be recruited. With a 2:1 randomization ratio, the number of participants in the DHI group is 564 and that in the control group is 282. We planned interim analyses at n = 282 and n = 564 patients using the O’Brien-Fleming boundary approach.

**3.2.2 Sample size re-estimation**

At the first interim analysis, 282 patients completed the trial. In the DHI group, 106 out of 188 patients (56.50%) achieved excellent functional outcome (an mRS score of 0-1 compared to 46 out of 94 patients (48.94%) in the placebo group (Z_1_ = 1.19, P_1_ = 0.23). According to the recommendation of DMC, the trial continued without sample size reassessment.

At the second interim analysis, 564 patients completed the trial. In the DHI group, 60.94% of patients achieved excellent functional outcome (an mRS score of 0-1) compared to 54.82% of patients in the placebo group (Z_2_ = 1.41, P_2_ = 0.16). The study did not reach the stopping boundary; therefore the sample size was estimated again to be 1261, inflated approximately 20% to a maximum of 1513 patients to account for potential losses to follow-up and dropouts (Table 1). Therefore, according to the recommendation of DMC, the sample size will be enlarged to be 1513, in which 1008 will be enrolled in Danhong group and 505 in control group.

**Table 1 The treatment effect on the primary outcome in the two interim analyses in the intention-to-treat and the per-protocol populations***

| **Interim analysis** | **Populations** | **DHI（%）** | **Control（%）** | **Difference（%）** | **Z-value** | **P-value** |
| --- | --- | --- | --- | --- | --- | --- |
| 1 | ITT | 56.50 | 48.94 | 7.56 | 1.19 | 0.23 |
|  | PPS | 58.24 | 49.46 | 10.38 | 1.37 | 0.17 |
| 2 | ITT | 60.94 | 54.82 | 6.12 | 1.41 | 0.16 |
|  | PPS | 62.43 | 55.03 | 7.40 | 1.67 | 0.09 |

* DHI:Danhong injection; ITT=intention-to-treat; PPS=per-protocol set. The difference between two groups were done using chi-square test.

**3.3** **Randomization**

All eligible patients who consent to participation will be randomized into either the Danhong injection or the placebo group in a 2:1 ratio. Randomization will be conducted using the central online randomization system (Brightech, Somerset, USA).The system generated a randomization number and randomized patients in randomly generated blocks of six.

**3.4 Blinding**

All patients, treating physicians and statisticians will be blinded to Danhong injection therapy or placebo treatment group assignments until the study has completed. Because the colour of Danhong injection and 0.9 % saline are different,the dropping bottles will be wrapped in sealed shaded bags, and brown infusion devices will be used for infusion. These procedures will be implemented by two professional nurses who will be required to sign a confidentiality agreement before study initiation and not to contact each other. One of the professional nurses will be in charge of preparing the drugs in a special transfusion room and sealing the infusion bottles with shaded brown bags. The other nurse will take the prepared drugs from the transfusion room to the infusion nurse and supervise the infusion process to ensure that the allocation of the drugs is blinded to the patients (the shaded brown bags will not be unwrapped during infusion and will be checked for integrity after infusion).

**3.5 Interim analysis**

Because this is an adaptive trial, two interim analyses are prospectively planned, and these will be performed in an unblinded manner by an independent statistician in the data monitoring committee (DMC) support group after one-third and two-thirds of the patients, respectively, have completed the trial. The statistical results of the interim analyses will be discussed in a close meeting of the DMC, who will decide on the re-estimation of sample size and determine whether any subsequent modifications must be made to the trial protocol. Because the two interim analyses may lead to an increased possibility of a type I error, we will apply the Lan-DeMets alpha spending function with an O’Brien-Fleming boundary to adjust the results.

**4．SUBJECTS**

**4.1 Diagnostic criteria**

**4.1.1 Disease Diagnostic criteria**

The diagnostic criteria for acute ischemic stroke is determined according to the Chinese Guidelines for the Diagnosis and Treatment of Acute Ischemic Stroke (2010)^12^.

Ischemic stroke causing a measurable neurological deficit defined as impairment of language, motor function, cognition and/or gaze, vision or neglect. Ischemic stroke is defined as an event characterized by the sudden onset of an acute focal neurologic deficit presumed to be due to cerebral ischemia after computed tomography (CT) scan excludes hemorrhage.

Interpretation of imaging should be performed using the Alberta Stroke Program Early CT Score (ASPECTS, range 0-10, with 1 point subtracted for any evidence of early ischaemic change in each defined region on the CT or MRI scan)^13^ at a central core laboratory by two independent superior neurology imaging doctors.

**4.1.2. Zhenghou Diagnostic criteria**

The diagnostic criteria of the “Xueyu Zheng” in Stroke is determined according to the Guidelines for Clinical Research of New Drugs of Traditional Chinese Medicine (2002)^14^, Guideline for Diagnosis and Effect Evaluation of Stroke (1996)^15^ and Zhenghou Diagnostic Scale in Ischemic Stroke^16^.

We will choose the patients with a clinical diagnosis of “Xueyu Zheng” (blood stasis syndrome), which is defined as a score of at least 20 on the Chinese Medicine Symptom Diagnostic Scale of “Xueyu Zheng” for acute ischemic stroke patients.

The Chinese Medicine Symptom Diagnostic Scale of “Xueyu Zheng” includes the 12 items as Table 2.

Table 2. **Chinese Medicine Symptom Diagnostic Scale of “Xueyu Zheng”**

| Symptoms | With | Without | Score |
| --- | --- | --- | --- |
| Hemiplegia | 10 | 0 |  |
| Numbness of limbs | 10 | 0 |  |
| Dimmish and darkish complexion | 9 | 0 |  |
| Dark purple lips | 8 | 0 |  |
| Rough skin | 4 | 0 |  |
| Pain in a fixed body location | 5 | 0 |  |
| Purple or darkish tongue | 10 | 0 |  |
| Ecchymosis on the tongue | 10 | 0 |  |
| Purple sublingual vessels | 10 | 0 |  |
| Sublingual varices | 8 | 0 |  |
| Unsmooth pulse | 8 | 0 |  |
| Intermittent pulse | 1 | 0 |  |
| Total Score* | | |  |

* If the total score ≥20, the patiet should be diagnosed as ischemic stroke with “Xueyu Zheng”.

**4.2 Inclusion Criteria**

(1). Female or male inpatients.

(2). Age: 18–70 years.

(3). Clinical diagnosis of ischemic stroke causing a measurable neurological deficit defined as impairment of language, motor function, cognition and/or gaze, vision or neglect. Ischemic stroke is defined as an event characterized by the sudden onset of an acute focal neurologic deficit presumed to be due to cerebral ischemia after computed tomography (CT) scan excludes hemorrhage.

(4). Clinical diagnosis of "Xueyu Zheng" (Blood Stasis Syndrome) as the scores of Chinese Medicine Symptom Diagnostic Scale of "Xueyu Zheng" in ischemic stroke ≥ 20.

(5). Onset of symptoms within 1 week prior to initiation of study drug administration.

(6). Patients with a clinical diagnosis of cerebral anterior circulation obstruction.

(7). 4≤National Institutes of Health Stroke Scale (NIHSS) score <20.

(8). Patient is willing to participate voluntarily and to sign a written patient informed consent. Informed consent will be obtained from each patient or the subject's legally authorized representative or relatives.

**4.3 Exclusion Criteria**

(1). Evidence of intracranial hemorrhage (ICH) or other cerebral diseases (e.g., vascular malformation, tumor, abscess or multiple sclerosis) on the CT or magnetic resonance imaging (MRI) scan.

(2). Patients who underwent thrombolysis or endovascular treatment.

(3). Known history of allergy or suspected allergy to the study drug.

(4). Fasting blood glucose <2.8 or >16.8 mmol/l or with severe complications due to diabetes (e.g., peripheral neuropathy, or diabetic gangrene).

(5). Liver function impairment with the value of alanine aminotransferase (ALT) or aspartate aminotransaminase (AST) over 1.5-fold the upper limit of normal range.

(6). Renal dysfunction with the value of serum creatinine over 1.5-fold the upper limit of normal range.

(7). Severe cardiac dysfunction on echocardiogram or the New York Heart Association (NYHA) heart functional class III or IV.

(8). History of prior stroke with a modified Rankin Scale (mRS) score ≥2.

(9). Complicated with atrial fibrillation.

(10). Severe stroke as assessed by appropriate imaging techniques (e.g., massive cerebral infarction affecting more than one lobe of the brain or over one-third of the blood-supply area of the middle cerebral artery).

(11). Physical disability before onset.

(12).Hemorrhagic tendency or severe or dangerous bleeding within the prior 3 months.

(13).Suspected alcohol addiction or drug abuse.

(14).Severe complications that would make the condition more complicated as assessed by the investigator.

(15).Women who are pregnant, lactating or have a positive pregnancy test, or women who plan to become pregnant in the subsequent 6 months.

(16).Women who have a menstrual period at baseline.

(17).Patients who are participating in other trials or have participated in other trials within the prior 3 months.

**4.4 Withdrawal/dropout criteria**

(1) Those who did not fulfill the eligibility criteria but were included by mistake;
(2) Those who fulfilled the eligibility criteria but did not take any study drug after randomization;
(3) Those with poor compliance that might interfere with the efficacy and safety evaluation;
(4) Those who experienced serious AEs, complications or special physiological changes, making it inappropriate to continue with the trial;
(5) Individual cases that were unblinded prematurely;
(6) Those who discontinued the trial voluntarily;
(7) Those who used any disallowed concomitant medication, especially those that had an obvious effect on the study drug and might interfere with the efficacy and safety evaluation;
(8) Those who discontinued or lost to follow-up for any other reasons or died during the trial;
(9) Those with incomplete data that might interfere with the efficacy and safety evaluation.

For all withdrawal/dropout cases, the potential reasons must be recorded. Patients who experienced any AE during the trial must be included in the statistical analysis of adverse drug reactions. Those who have taken the study drug for at least 1 week should be included in the statistical analysis of drug efficacy.

**4.5 Criteria for study termination**

Circumstances that may warrant termination include, but are not limited to:

(1) Identification of unexpected, significant, or unacceptable risk to subjects;
(2) Poor efficacy or no efficacy of the study drug;
(3) Major mistakes in the trial protocol;
(4) Insufficient adherence to protocol requirements;
(5) Upon sponsor’s request (e.g. economic or administrative reasons).

**4.6 Criteria for subject discontinuation**

(1) Any allergic reaction or AE occurs such that continued participation in the study would not be in the best interest of the subject at the discretion of the treating physician;
(2) Worsening of clinical conditions occurs such that continued participation in the study would not be in the best interest of the subject at the discretion of the treating physician; considered as ineffective cases;
(3) Subjects are free to withdraw from participation in the study at any time upon request.

In any case, every effort must be made to determine why patients discontinued the study treatment prematurely, which may include but are not limited to poor confidence in efficacy, occurrence of AEs, SAEs, or other serious complications, worsening of symptoms such that emergent intervention is required. As for those who withdrew from participation in the study or lost to follow-up, it is essential to investigate the potential reasons via telephone. Investigators should record in detail when and how the last dose was administered, the efficacy and safety parameters at and after discontinuation, the relationship between discontinuation or withdrawal and the study drug, and the potential impact of discontinuation cases on the final conclusion of the study. Moreover, case report form (CRF) of withdrawal/dropout cases should also be completed and their original data should also be documented and stored in a secure manner.

**4.7 Trial flow Chart**

The trial flow chart was shown in Figure 1.

Figure 1. The schedule of enrollment, interventions, and assessments

**5 INTERVENTION**

**5.1 Routine medical care**

Routine medical care is in accordance with China Guideline for the Diagnosis and Treatment of Acute Ischemic Stroke (2010)^12^, including control of temperature, blood pressure, lipid and glucose, improving cerebral blood circulation, anti-platelet treatment, anticoagulants, neuroprotective drugs, stroke rehabilitation, and stroke prevention care.

All of these basic treatments will be recorded in detail in the patients’ medical records as well as in their e-CRFs.

**5.2 Treatment regimens**

DHI group: Participants will be treated using Danhong injection (40 ml per day) plus 0.9 % normal saline (250 ml intravenously per day).

Control group: Participants will be treated using placebo (0.9 % normal saline, 40 ml per day) plus 0.9 % normal saline (250 ml intravenously per day).

**5.3 Duration and follow-up**

All of the included patients will undergo a 14-day treatment regimen and a 76-day follow-up period.

**5.4 Concomitant medications**

(1) Participants are not allowed to take any other Chinese herbal medicines or Chinese patent medicines that have the effect of promoting blood circulation or removing blood stasis throughout the study period.

(2) If other medication or therapy is required to treat the concomitant diseases, the name of the drug or therapy, dosage, dosing frequency and start/stop time should be well-documented.

**6 OUTCOMES MEASUREMENTS**

**6.1 Primary outcome**

The primary outcome is the proportion of patients with excellent functional outcome, defined as a score of 0–1 on the mRS^17^ at day 90. The mRS is a commonly used scale for measuring the degree of disability or dependence in the daily activities of people who have suffered a stroke or other causes of neurological disability. The scale runs from 0–6, ranging from perfect health without symptoms to death.

**6.2 Secondary Outcomes**

6.2.1 The efficacy evaluation scale of Chinese Medicine Symptoms in “Xueyu Zheng” of ischemic stroke ^15^

The efficacy evaluation scale of Chinese Medicine Symptoms in “Xueyu Zheng” of ischemic stroke (Table 3) will be used to assess the condition of patients with *Xueyu Zheng* at each visit (Day 0, Day 7, Day 14, Day 30, Day 60, Day 90).

**Table 3 The efficacy evaluation scale of Chinese Medicine Symptom in “Xueyu Zheng” of ischemic stroke**

| Symptoms | Visual Analogue Scale* | Score |
| --- | --- | --- |
| Hemiplegia | 0------------------------------------10 |  |
| Numbness of limbs | 0------------------------------------10 |  |
| Dimmish and darkish complexion | 0------------------------------------9 |  |
| Dark purple lips | 0------------------------------------8 |  |
| Rough skin | 0------------------------------------4 |  |
| Pain in a fixed body location | 0------------------------------------5 |  |
| Purple or darkish tongue | 0------------------------------------10 |  |
| Ecchymosis on the tongue | 0------------------------------------10 |  |
| Purple sublingual vessels | 0------------------------------------10 |  |
| Sublingual varices | 0------------------------------------8 |  |
| Unsmooth pulse | 0------------------------------------8 |  |
| Intermittent pulse | 0------------------------------------1 |  |

* According to the severity of the symptoms, the more severe the symptom is, the higher the score be determined. If the symptom is disappeared, the score will be recorded as 0.

The mean total score of symptoms in a questionnaire of *Xueyu Zheng* will be analyzed and compared between two groups at each visit. Moreover, the proportions of patients with improvement in *Xueyue Zheng* between the two groups will be recorded at each visit. Significant “*Xueyu Zheng*” improvement is defined as at least 70% reduction in the *Xueyue Zheng* score^15^.

6.2.2 The proportion of patients with the Barthel Index (BI) score of ≥90.

BI uses ten variables to describe the activities of daily living (ADL) and mobility, and a higher number is associated with a better performance of ADL^18^. At each visit, investigators will observe the proportions of patients with the Barthel Index (BI) score of ≥90 in each treatment group.

6.2.3 The proportion of patients with an improvement in the NIHSS score of ≥4 or the NIHSS score of 0-1.

The patients will be asked to record their NIHSS score. NIHSS is a tool used to objectively quantify the impairment caused by stroke. A NIHSS score of 0 typically indicates normal function in that specific ability, while a higher score is indicative of some level of impairment^19^. At each visit (Day 7, 14, 30, 60, 90), the proportion of patients with an improvement in the NIHSS score of ≥4 or the NIHSS score of 0-1 will be compared between two groups.

6.2.4 The quality of life measured by the EQ-5D scale.

The EQ-5D scale has five standard dimensions to measure the generalized health-related quality of life^20^. It is a standardized instrument for the measurement of health status with Time Trade-Off Value for different race group. Here, we need to find the TTO value of EQ-5D for Chinese population. The mean TTO value of EQ-5D scale will be analyzed and compared between two groups at each visit (Day0, 7, 14, 30, 60, 90).

6.2.5 Global disability at day 90.

Global disability, which defined as the proportion of patients whose mRS≥3 except death, will be compared at Day 90.

6.2.6 Incidence of new-onset major vascular events within 90 days.

Major adverse vascular events include ischemic stroke, hemorrhagic stroke, transient ischemic attack, myocardial infarction and vascular-related death. The proportion of patients with new-onset major vascular events within 3 months will be compared between two groups.

**6.3 Other Pre-specified Efficacy Outcomes**

Changes in the micro-RNA and mRNA profiles of 96 patients selected from Tiantan Hospital and Xuanwu Hospital. The detail of these outcomes will be shown in the RNA sequencing protocol for the mechanism of Danhong injection in the treatment of ischemic stroke.

**6.4 Safety Outcomes**

6.4.1 Overall mortality within 90 days.

6.4.2 Incidence of severe haemorrhages within 90 days.

Severe haemorrhages is defined according to the GUSTO bleeding criteria^21^, including fatal bleeding, primary intracranial hemorrhage, post-traumatic symptomatic intracranial hemorrhage, or bleeding requiring transfusion, infusion, use of vasoconstrictor drugs and surgical intervention.

6.4.3 Incidence of moderate haemorrhages within 90 days.

Moderate haemorrhages is also defined according to the GUSTO bleeding criteria^21^, including bleeding requiring infusion but do not reach severe bleeding, absolute reduction in hemoglobin/hematocrit, severe disability induced by bleeding, intraocular hemorrhage with severe visual field defects, etc.

6.4.4 Incidence of adverse and serious adverse events (Section 7).

**6.5 Study Schema**

| **Period** | **Baseline** | **Treatment period** | | **Follow-up period** | | |
| --- | --- | --- | --- | --- | --- | --- |
| Day | 0 | 7±2 | 14±2 | 30±5 | 60±5 | 90±5 |
| Informed consent | × |  |  |  |  |  |
| Inclusion/exclusion criteria | × |  |  |  |  |  |
| Medical history | × |  |  |  |  |  |
| UCG | × |  |  |  |  |  |
| TOAST | × |  |  |  |  |  |
| ASPECTS | × |  |  |  |  |  |
| Medical examination | × | × | × | × | × | × |
| Concomitant medications | × | × | × | × | × | × |
| **Outcomes** |  |  |  |  |  |  |
| BI scale | × | × | × | × | × | × |
| mRS scale | × | × | × | × | × | × |
| NIHSS | × | × | × | × | × | × |
| Efficacy evaluation scale of Chinese Medicine Symptom in “Xueyu Zheng” | × | × | × | × | × | × |
| EQ-5D scale | × | × | × | × | × | × |
| Global disability on mRS |  |  |  |  |  | × |
| New-onset major vascular events |  |  |  |  |  | × |
| Overall mortality |  |  |  |  |  | × |
| Incidence of severe hemorrhages |  |  |  |  |  | × |
| Incidence of moderate hemorrhages |  |  |  |  |  | × |
| AEs and SAEs |  | × | × | × | × | × |
| Profiles of micro-RNA in 96 patients in certain centers | × |  |  | × |  | × |
| Profiles of mRNA in 96 patients in certain centers | × |  |  | × |  | × |

*UCG: ultrasound cardiogram; TOAST: Trial of Org 10 172 in Acute Stroke Treatment; ASPECTS: Alberta Stroke Programme Early CT Score; BI: Barthel Index; mRS: modified Rankin Scale; NIHSS: National Institutes of Health Stroke Scale; AEs: adverse events; SAEs: serious adverse events; microRNA: micro-ribonucleic acid; mRNA: messenger ribonucleic acid.

**7 ADVERSE EVENTS**

**7.1 Definition**

**Adverse event (AE)**: An AE is any untoward medical occurrence in a patient or clinical investigation subject administered a pharmaceutical product and that does not necessarily have a causal relationship with this treatment. An AE can therefore be any unfavorable and unintended sign (including an abnormal laboratory finding), symptom, or disease temporally associated with the use of a medicinal (investigational) product, whether or not related to the medicinal (investigational) product. Pre-existing conditions which worsen during a study are also to be reported as AEs.

**Serious Adverse Event (SAE):** A SAE is any experience that suggests a significant hazard, contraindication, side effect or precaution. It is any AE that at any dose fulfils at least one of the following criteria:

– is fatal;

– is life-threatening;

– required in-patient hospitalization or prolongation of existing hospitalization;

– results in persistent or significant disability/incapacity;

– is a congenital anomaly/birth defect;

– is medically significant or requires intervention to prevent one or other of the outcomes listed above.

**Adverse drug reaction (ADR):** An ADR is any unintended harm or injury associated with the use of given medications at a normal dosage during normal use. In clinical trials of any new drug or any new usage of a certain drug, any unintended harm or injury that has a causal relationship to the use of the drug should also be considered as an ADR.

**7.2 Recording**

For all AEs encountered during the clinical study, the following must be assessed and recorded on the adverse events form of the e-CRF: intensity, relationship to study drug, action taken regarding study drug, and outcome to date. All AEs, especially those for which the relationship to test drug is not “unrelated”, should be followed up until they have returned to baseline status or stabilized. The follow-up procedures could be performed in clinic visits or during hospitalization, or via home visit, telephone or mail, depending on the severity of AEs.

**7.3 Severity of an AE**

**Mild:** events require minimal or no treatment and do not interfere with the subject’s daily activities.

**Moderate:** events result in a low level of inconvenience or concern with the therapeutic measures. Moderate events may cause some interference with functioning.

**Severe:** events interrupt a subject’s usual daily activity and may require systemic drug therapy or other treatment. Severe events are usually incapacitating.

**7.4 Evaluation the correlation between AEs and study drugs**

Based on the current 6-level evaluation standard of the China National Center for Adverse Drug Reaction Monitoring, according to the answers to 5-iterm questionnaire, the correlation between AEs and study drugs can be categorized as to definitely related, probably related, possibly related, unlikely related, to be evaluated, and unable to evaluate (Table 4) . The adverse drug reaction was determined as "definitely related", "probably related", " possibly related".

**Table 4 The assessment of the causality between adverse events and observed drug**

| **Item*** | **Index** | | | | | |
| --- | --- | --- | --- | --- | --- | --- |
|  | **1= definitely related** | **2= probably related** | **3=**  **possibly related** | **4=**  **unlikely related** | **5=**  **to be evaluated** | **6=**  **unable to evaluate** |
| A reasonable timing relationship^1^ | + | + | + | - | Additional detailed information to be evaluated | The necessary information for the evaluation is not available |
| Belonging to the Known ADR of the drug ^2^ | + | + | ± | - |  |  |
| Relief after stopping the drug^3^ | + | + | ±? | ±? |  |  |
| Recurrent AE after re-administration^4^ | + | ? | ? | ? |  |  |
| Suspected adverse reaction could be explained with other certain reasons^5^ | - | - | ±? | ±? |  |  |

*The 5-iterm questionnaire to assess the causality between adverse events and observed drug is listed in the items 1 to 5, detailed as following:

2. Is there a reasonable relationship between the time of starting the drug and the time of occurrence of the suspected adverse reaction?

2. Is the suspected adverse drug reaction consistent with the types of adverse reaction known to the drug?

3.Could the suspected adverse reaction be alleviated or disappeared after stopping the drug or reducing the dosage?

4. Dose the same adverse event occur again after given the same drug again?

5. Could the suspected adverse reaction be explained by the patient's pathological condition, combined medication, combined therapy, and used therapy etc?

**7.5 Handling of SAEs**

All SAEs should be managed properly and timely, and recorded on the appropriate SAE eCRF. Any AE considered serious by the investigator or which meets the aforementioned criteria must be submitted on a signed and dated SAE form to the sponsor, ethics review committee and relevant authorities within 24 hours of site awareness. And the sponsor should also inform all study sites of such SAEs in a timely manner to ensure subjects’ safety in accordance with applicable laws and regulations.

Fatal or life-threatening events considered to be caused by the study drugs must be reported immediately to the sponsor and relevant authorities via telephone, fax, EMS or E-mail.

**7.6 Opening and handling of emergency envelops**

The emergency envelope provided along with each test drug in its package should only be opened in case of a serious complication or a serious adverse event, which is called emergency unblinding. Once unblinded, the patient should be withdrawn from the study and regarded as a dropout case. Information about when, where and why the emergency unblinding occurs as well as each personnel’s signature should also be documented.

**7.7 Follow-up of unresolved AEs**

All AEs should be followed up until they have been resolved, stabilized or returned to baseline status.

9. **DATA COLLECTION AND MANAGEMENT**

**8.1 Establishment of Three Committees**

To ensure the effectiveness and integrity of the trial design, three committees have been established by the multicentre trial coordination group: the clinical trial steering committee, the data monitoring committee (DMC) and the outcome evaluation committee. The first committee is responsible for the study design and the implementation process. The DMC will supervise the integrity and accuracy of data collection to control its quality. The outcome evaluation committee will evaluate key outcomes (including outcome measurements and adverse events) based on their clinical expertise.

**8.2 Data Management**

(1) All data will be recorded by trained clinical investigators using standardized e-CRFs in Brightech clinical information management systems (Brightech, Somerset, NJ, USA).

(2) The accuracy and reliability of the data will be ensured by the study monitor, who will verify and cross-check the e-CRFs against the investigator’s records (source document verification), using the maintenance of a drug dispensing log by the investigator.

(3) Front-end checks in the e-CRFs and back-end checks in the clinical information management system will be used as a validation check of the data.

(4) If there are any discrepancies in the e-CRFs, the results will be sent to the investigator for resolution.

(5)The results of the analysis must not be released with individual identification of the subjects until the database is closed.

**8.3 Data Report**

Data report contains the aspects as followed: (1) members of the project; (2) disagreement from the primary data management plan; (3) actual finish time of every project; (4) problems and the solution during the data management (if have any); (5) reconstruction of the database (if have any); (6) distribution of the participants; (7) participants who disobey the trial protocol; (7) classifying plan of the statistical analysis population. Data report will be performed monthly and then send to DMC since the first entry of the e-CRF.

**8.4 Data Blind Review**

When the data checking is finished, a data blind review meeting will be hold. On the meeting, the data administrators, statisticians, researchers, clinical inspectors, and other related members would have a discussion on the following items according to the data management report and the data lists:

•Distribution of the participants;

•Protocol disobeying or not;

•Possible outlier;

•Baseline data;

•Outcomes;

•Statistical analysis plan.

Participants will be classified to their suitable statistical analysis sets according to the definition in the protocol. No patient can be excluded from the analysis, unless getting the permission of the meeting participants. All the meeting participants should sign the data locking consent, and the data auditing resolution.

**8.5 Database Locking**

The database will be locked if it fulfills all the aspects as followed:

•All the queries have been solved, and the database has been updated;

•No query has been found through the data inspection;

•The medical coding has been completed;

•The plan of the participants’ classification has been approved;

•The final draft of the SAP has been made, and approved by the project leader.

The statisticians and the data administrators will sign the data locking form, and then the database will be locked. The locked database will be sent to the statisticians for further statistical analysis through the data format of SAS.

**9. STATISTICAL CONSIDERATIONS**

**9.1 Study Populations**

All patients with randomization will be included in the analysis set regardless of whether they receive any treatment. Most outcome measurements (except micro-RNA and mRNA profiles) will be analyzed on the intention-to-treat (ITT) and per-protocol population (PPS). Safety analysis will be performed in a safety set, which is defined as a subset of subjects who were randomized and who received at least one treatment.

**9.2 Statistical Analyses**

The primary study hypothesis is that an initial usage of DHI added to routine medical care could bring more clinical benefit for the patients with acute ischemic stroke than an initial strategy of routine medical care alone. Missing data of the primary outcome were handled using the baseline-observation-carried-forward (BOCF) approach, while missing data of secondary outcomes were not imputed. The chi-square test will be used to compare the proportion of patients with a favorable outcome, defined as a score of 0–1 on the mRS at day 90 between the two groups. Pre-specified subgroup analysis will also be performed according to the Statistical Analysis Plan (SAP).

The secondary efficacy and safety outcomes will be analyzed using the two sample t-tests, Wilcoxon rank-sum test, Chi-square test, Poisson regression or Fisher’s exact test according to the Statistical Analysis Plan (SAP).

All reported P values were two-sided; and a P value less than 0.05 was considered to be statistically significant except that from the primary outcome analyses. All calculations were performed using SAS software, version 9.4 (SAS Institute, Cary, NC).

**9.3 Statistical Analysis Plan (SAP)**

Prior to database lock and before code breaking, a final version of the SAP shall be issued and approved by the study statistician, and the principal investigator. The SAP will define all “pre-specified, planned analyses” and provide the general specifications for the analysis of the data to be collected and presented in clinical information management systems.

**10 ETHICAL ASPECTS**

The investigator will ensure that this study is conducted in full conformance with the principles of the “Declaration of Helsinki” and applicable laws and regulations on the conduct of clinical trials.

This trial firstly will be approved by center institutional review board (IRB) in Beijing Beijing Tiantan Hospital, Capital Medical University . For the other sites, if need, the trail also should be approved by the local IRB at the certain enrolling sites. Only when the trial protocol is approved by the IRB, the enrollment of participant will begin.

Informed consent is a process that is initiated prior to the individual’s agreeing to participate in the study and continues throughout the individual’s study participation. Extensive discussion of risks and possible benefits of this treatment will be provided to the subjects and their families. Consent forms describing in detail the study interventions/products, study procedures, and risks are given to the subject and written documentation of informed consent is required prior to starting intervention/administering study product. It must also be explained to the patients that they are completely free to refuse to enter the study or to withdraw from it at any time for any reason. A copy of the informed consent document will be given to the subjects for their records.

**REFERENCES**

1. Matsuo R, Kamouchi M, Ago T, Hata J, Shono Y, Kuroda J, et al. Thrombolytic therapy with intravenous recombinant tissue plasminogen activator in Japanese older patients with acute ischemic stroke: the Fukuoka Stroke Registry. Geriatr Gerontol Int. 2014;14:954–9.

2. Chen J, Venkat P, Zacharek A, Chopp M. Neurorestorative therapy for stroke. Front Hum Neurosci. 2014;8:382.

3. Turner RC, Lucke-Wold B, Lucke-Wold N, Elliott AS, Logsdon AF, Rosen CL, et al. Neuroprotection for ischemic stroke: moving past shortcomings and identifying promising directions. Int J Mol Sci. 2013;14:1890–917.

4. Behravan E, Razavi BM, Hosseinzadeh H. Review of plants and their constituents in the therapy of cerebral ischemia. Phytother Res. 2014; 28:1265–74.

5. Li X, Tang J, Meng F, Li C, Xie Y. Study on 10 409 cases of post-marketing safety Danhong injection centralized monitoring of hospital. Zhong guo Zhong Yao Za Zhi. 2011;36:2783–5.

6. He Y, Wan H, Du Y, Bie X, Zhao T, Fu W, et al. Protective effect of Danhong injection on cerebral ischemia-reperfusion injury in rats. J Ethnopharmacol. 2012;144:387-94.

7. Xiujuan Mi, Guangqin Li. Effect of Danhong on Neural Functional Recovery after Focal Cerebral Ischemia in Rats and Its Mechanism. Zhongguo Quanke Yixue. 2010;13:870-3.

8. He Y, Wan H, Du Y, Bie X, Zhao T, Fu W, et al. Protective effect of Danhong injection on cerebral ischemia-reperfusion injury in rats. J Ethnopharmacol. 2012;144:387–94.

9. Ma L, Wang S, Li K. A systematic review of Danhong injection treatment for acute cerebral ischemic stroke and the safety of this treatment. Journal of Emergency in Traditional Chinese Medicine. 2012;10:1609–10.

10.Shuaib A, Lees KR, Lyden P, Grotta J, Davalos A, Davis SM, et al. NXY-059 for the treatment of acute ischemic stroke. N Engl J Med. 2007;357:562–71.

11. Hacke W, Kaste M, Bluhmki E, Brozman M, Davalos A, Guidetti D, et al. Thrombolysis with alteplase 3 to 4.5 hours after acute ischemic stroke. N Engl J Med. 2008;359:1317–29.

12. Chinese Society of Neurology. China Guideline for the Diagnosis and Treatment of Acute Ischemic Stroke. Chin J Neurol. 2010;43:146–53.

13.Barber PA, Demchuk AM, Zhang J, Buchan AM, Group AS. Validity and reliability of a quantitative computed tomography score in predicting outcome of hyperacute stroke before thrombolytic therapy. ASPECTS Study Group. Alberta Stroke Programme Early CT Score. Lancet 2000; 355: 1670–7

14. Zheng X. Guidelines of Clinical Research of New Drugs of Traditional Chinese Medicine. Beijing: China Medical Science Press; 2002.

15. Brain disease emergency cooperation group of State Administration of traditional Chinese Medicine, Criteria For Diagnosis And Curative Effect Evaluation Of Stroke, 1996,19(1):55-56

16. Ying Gao, Bin Ma, Qiang Liu, Yongyan Wang. Development and methodology discussion of the diagnostic scale of ischemic stroke syndrome elements. Chinese Journal of Traditional Chinese Medicine. 2011;52: 2097-101.

17. van Swieten JC, Koudstaal PJ, Visser MC, Schouten HJ, van Gijn J. Interobserver agreement for the assessment of handicap in stroke patients. Stroke. 1988;19:604-7.

18. Mahoney FI, Barthel DW. Functional evaluation: the Barthel Index. Md State Med J. 1965;14:61–5.

19. Wityk RJ, Pessin MS, Kaplan RF, Caplan LR. Serial assessment of acute stroke using the NIH Stroke Scale. Stroke. 1994;25:362–5.

20. Brooks R. EuroQol: the current state of play. Health Policy. 1996;37:53–72.

21. Mehran R, Rao SV, Bhatt DL, Gibson CM, Caixeta A, Eikelboom J, et al. Standardized bleeding definitions for cardiovascular clinical trials: a consensus report from the Bleeding Academic Research Consortium.Circulation. 2011;123:2736-2747.

**SUMMARY OF CHANGES IN THE PROTOCOL**

All changes in final version of the protocol appear in blue. Major changes include the following:

1. In **Participating Sites and Investigator,** 10 sites and corresponding investigators initially took part in this trail and at last 72 sites and corresponding investigators all over China have participated in our study.
2. After the second interim analysis, the sample size is expanded to 1513. The detailed information about the process of this change could be see in 3.2.2 **Sample size re-estimation.**

**Danhong Injection for the treatment of acute ischemic stroke (Phase IV): an adaptive-design, randomized, multi-center, double-blind, placebo-controlled clinical trial**

**(Danhong Phase IV, DH20120423)**

**Statistical Analysis Plan**

**The Original Version: December 12, 2012**

**Prepared for and approved by the Clinical Trial Steering Committee of the study:**

Prof. Yong-Yan Wang (Chair), Institute of Basic Research in Clinical Medicine, China Academy of Chinese Medical Sciences

Prof. Xiao-Xi Du (Member), State Food and Drug Administration

Prof. Bo-Li Zhang (Member), China Academy of Chinese Medical Sciences

Prof. Run-Lin Gao (Member), Fuwai Hospital Chinese Academy of Medical Sciences

Prof. Wei-Liang Weng (Member), Xi-yuan Hospital Affiliated to China Academy of Chinese Medical Sciences

Dr. Ai-Ping Lv (Member), 1. Institute of Basic Research in Clinical Medicine, China Academy of Chinese Medical Sciences; 2. School of Chinese Medicine, Hong Kong Baptist University

Prof. Ming-Hui Yang (Member), Chinese PLA General Hospital

**Prepared for Data Monitoring Committee**

Prof. Chun-Ti Shen (Chair), Changzhou TCM Hospital Affiliated to Nanjing University of Chinese Medicine

Prof. Qi-Guang Chen (Member, independent statistician), Southeast University

Prof. Hua-Qiang Zhang (Member), Jiangsu Provincial Commission of Health and Family Planning

Dr. Hong-Wu Wang (Member), Tianjin University of Traditional Chinese Medicine

Dr. Xiao-Chun Sun (Member), Beijing Kelunrui Pharmaceutical Technology Co. Ltd.

Prof. Cheng-Shu Wang (Member), Zhongda Hospital Southeast University

Dr. Hai-Xia Dang (Member), China Academy of Chinese Medical Sciences

Dr. Jun Liu (Member), Institute of Basic Research in Clinical Medicine, China Academy of Chinese Medical Sciences

**Prepared by an Independent Statistician:**

Dr. Bing-Wei Chen (Statistician), Southeast University

**Table of Contents**

[1. Introduction	- 1 -](#_Toc19160)

[2. Study Objective	- 2 -](#_Toc17997)

[3. Design	- 2 -](#_Toc17399)

[3.1 Overview	- 2 -](#_Toc29644)

[3.2 Eligibility criteria	- 2 -](#_Toc16130)

[3.2.1 Diagnostic criteria	- 2 -](#_Toc3504)

[3.2.3 Exclusion Criteria	- 4 -](#_Toc3943)

[3.3 Efficacy and Safety Outcomes	- 5 -](#_Toc10359)

[3.3.1 Primary Efficacy Outcome	- 5 -](#_Toc5356)

[3.3.2 Secondary Efficacy Outcomes	- 5 -](#_Toc24609)

[3.3.3 Other Pre-specified Efficacy Outcomes	- 6 -](#_Toc19385)

[3.3.4 Safety Outcomes	- 6 -](#_Toc32261)

[3.4 Sample Size Calculation	- 6 -](#_Toc25258)

[3.5 Study Schema	- 7 -](#_Toc19173)

[4. Data Monitoring and Interim Analyses	- 9 -](#_Toc4578)

[4.1 Data Management	- 9 -](#_Toc14156)

[4.2 Establishment of Three Committees for DHI Phase IV Trials	- 9 -](#_Toc11162)

[4.3 Interim Analyses	- 10 -](#_Toc26108)

[5. Statistical Considerations	- 10 -](#_Toc22570)

[5.1 Study Hypothesis	- 10 -](#_Toc12624)

[5.2 Study Populations	- 10 -](#_Toc17459)

[5.3 Methods of Statistical Analyses	- 11 -](#_Toc181)

[5.3.1 The General Principle	- 11 -](#_Toc15129)

[5.3.2 Derived data	- 11 -](#_Toc3556)

[5.3.3 The Management of Missing data	- 11 -](#_Toc23185)

[5.3.4 Statistical Comparisons between Groups	- 12 -](#_Toc1167)

[5.3.4.1 Distribution of participants	- 12 -](#_Toc30963)

[5.3.4.2 Demographics and Baseline Characteristics	- 12 -](#_Toc7517)

[5.3.4.3 Compliance and concomitant medication	- 12 -](#_Toc24912)

[5.3.4.4 Analyses for Primary Outcome	- 12 -](#_Toc8209)

[5.3.4.5 Analyses for Secondary Outcomes	- 13 -](#_Toc10607)

[5.3.4.6 Other Pre-specified Efficacy Outcomes	- 14 -](#_Toc19492)

[5.3.4.7 Safety Analyses	- 14 -](#_Toc14470)

[5.3.5 Interim Analyses	- 15 -](#_Toc780)

[5.3.6 Analysis Software	- 15 -](#_Toc3304)

[References	- 15 -](#_Toc10681)

# 2. Introduction

Acute ischemic stroke (AIS), characterized by an occlusion of the cerebral artery, is one of the leading causes of mortality, long-term disability, and morbidity. Thrombolytic therapy with intravenous tissue plasminogen activator (tPA), which remains the only therapeutic drug for AIS approved by the US Food and Drug Administration, is limited by the narrow time window of thrombolysis, bleeding complications, or high costs^1^. Other treatment strategies mainly utilize therapeutic agents to prevent or reduce cell damage from ischemia^2^. Hundreds of neuro protective agents for ischemic stroke have reached clinical trials, but no optimal drugs for the clinical treatment of AIS have yet been developed^3^. This leads us to search for novel therapeutic approaches, and to investigate whether natural compounds extracted from herbs may be an alternative option to improve treatment for AIS^4^.

Danhong injection (DHI), extracted from Radix Salviae miltiorrhizae (Danshen) and Flos Carthami tinctorii (Honghua), is a Chinese medicinal product approved by the State Food and Drug Administration (SFDA), and it is widely used in China for treating AIS^5^. Previous studies demonstrated the neuroprotective efficacy of DHI in rat models with cerebral ischemic-reperfusion injury^6, 7^, and that the underlying pharmacological mechanisms might involve anti-inflammatory, anti-coagulant, antithrombotic, antifibrinolytic, and antioxidant activities^8^. A number of clinical studies also supported the efficacy and safety of DHI in the management of AIS, but due to the methodological deficiencies of inadequate randomization, no double blinding, no placebo control, or incomplete outcome data, the quality of these studies was assessed as generally low^9^. There is a lack of critically appraised evidence such as well designed randomized controlled trials (RCTs) to justify its clinical use and recommendation. Thus, we aim to investigate the efficacy and safety of DHI in the treatment of AIS in a well-designed, randomized, multicenter, double-blind and placebo-controlled trial.

# 3. Study Objective

The objective of this study is mainly to determine whether Danhong injection is effective in the treatment of acute ischemic stroke.

# 4. Design

## 3.1 Overview

This study is a three-stage adaptive-design, randomized, multicenter, double-blind, placebo-controlled trial. The number of subjects is initially estimated to be 846 and randomly assigned to be 564 in DHI group and 282 in the control group. Both groups received routine medical care as recent guideline advocated. Participants in the DHI group are treated by DHI (40 ml, qd) plus 0.9% normal saline (250 ml, ivgtt, qd), while participants in the control group are treated by DHI placebo (0.9% normal saline, 40ml, qd) plus 0.9% normal saline (250 ml, ivgtt, qd).

## 3.2 Eligibility criteria

Eligible subjects are those who meet all of the following inclusion criteria and who do not have any of the listed exclusion criterion.

### 3.2.1 Diagnostic criteria

(1). The diagnostic criteria for acute ischemic stroke is determined according to the Chinese Guidelines for the Diagnosis and Treatment of Acute Ischemic Stroke (2010)^10^.

(2). The diagnostic criteria for “Xueyu Zheng” (Blood stasis syndrome) is determined according to the Guidelines for Clinical Research of New Drugs of Traditional Chinese Medicine (2002)^11^, Guideline for Diagnosis and Effect Evaluation of Stroke (1996)^12^ and Zhenghou Diagnostic Scale in Ischemic Stroke^13^.

**3.2.2 Inclusion Criteria**

(1). Female or male inpatients.

(2). Age: 18–70 years.

(3). Clinical diagnosis of ischemic stroke causing a measurable neurological deficit defined as impairment of language, motor function, cognition and/or gaze, vision or neglect. Ischemic stroke is defined as an event characterized by the sudden onset of an acute focal neurologic deficit presumed to be due to cerebral ischemia after computed tomography (CT) scan excludes hemorrhage.

(4). Clinical diagnosis of "*Xueyu Zheng*" (Blood Stasis Syndrome) as the scores of Chinese Medicine Symptom Diagnostic Scale of "*Xueyu Zheng*" in ischemic stroke ≥ 20.

(5). Onset of symptoms within 1 week prior to initiation of study drug administration.

(6). Patients with a clinical diagnosis of cerebral anterior circulation obstruction.

(7). 4≤National Institutes of Health Stroke Scale (NIHSS) score <20.

(8). Patient is willing to participate voluntarily and to sign a written patient informed consent. Informed consent will be obtained from each patient or the subject's legally authorized representative or relatives.

### 3.2.3 Exclusion Criteria

(1). Evidence of intracranial hemorrhage (ICH) or other cerebral diseases (e.g., vascular malformation, tumor, abscess or multiple sclerosis) on the CT or magnetic resonance imaging (MRI) scan.

(2). Patients who underwent thrombolysis or endovascular treatment.

(3). Known history of allergy or suspected allergy to the study drug.

(4). Fasting blood glucose <2.8 or >16.8 mmol/l or with severe complications due to diabetes (e.g., peripheral neuropathy, or diabetic gangrene).

(5). Liver function impairment with the value of alanine aminotransferase (ALT) or aspartate aminotransaminase (AST) over 1.5-fold the upper limit of normal range.

(6). Renal dysfunction with the value of serum creatinine over 1.5-fold the upper limit of normal range.

(7). Severe cardiac dysfunction on echocardiogram or the New York Heart Association (NYHA) heart functional class III or IV.

(8). History of prior stroke with a modified Rankin Scale (mRS) score ≥2.

(9). Complicated with atrial fibrillation.

(10). Severe stroke as assessed by appropriate imaging techniques (e.g., massive cerebral infarction affecting more than one lobe of the brain or over one-third of the blood-supply area of the middle cerebral artery).

(11). Physical disability before onset.

(12).Hemorrhagic tendency or severe or dangerous bleeding within the prior 3 months.

(13).Suspected alcohol addiction or drug abuse.

(14).Severe complications that would make the condition more complicated as assessed by the investigator.

(15).Women who are pregnant, lactating or have a positive pregnancy test, or women who plan to become pregnant in the subsequent 6 months.

(16).Women who have a menstrual period at baseline.

## (17).Patients who are participating in other trials or have participated in other trials within the prior 3 months.

## 3.3 Efficacy and Safety Outcomes

### 3.3.1 Primary Efficacy Outcome

The primary outcome is the proportion of patients with a favorable outcome, defined as a score of 0–1 on the mRS^14^ at day 90. The mRS is a commonly used scale for measuring the degree of disability or dependence in the daily activities of people who have suffered a stroke or other causes of neurological disability. The scale runs from 0–6, ranging from perfect health without symptoms to death.

### 3.3.2 Secondary Efficacy Outcomes

(1). The proportion of patients with a significant improvement in “*Xueyu Zheng*”, defined as at least 70% reduction in the Efficacy Evaluation Scale of Chinese Medicine Symptom in “*Xueyu Zheng*”;

(2). The proportion of patients with the Barthel Index (BI) score of ≥90;

(3) The proportion of patients with an improvement in the NIHSS score of ≥4 or the NIHSS score of 0-1;

(4) The quality of life measured by the EQ-5D scale;

(5) Global disability (mRS: 3-5) at 90 days;

(6) Incidence of new-onset major vascular events within 90 days.

### 3.3.3 Other Pre-specified Efficacy Outcomes

(1) The changes on the profiles of micro-RNA in 96 patients selected from certain centers；

(2) The changes on the expression profiles of mRNA in 96 patients selected from certain centers.

### The statistical analysis of these outcomes are not included in this statistical analysis plan, which will be shown in the RNA sequencing protocol for the mechanism of Danhong injection in the treatment of ischemic stroke.

### 3.3.4 Safety Outcomes

(1) Overall mortality within 90 days;

(2) Incidence of severe haemorrhages within 90 days;

(3) Incidence of moderate haemorrhages within 90 days;

(4) Incidence of adverse and serious adverse events.

## 3.4 Sample Size Calculation

The proportion of patients who had a favorable outcome, defined as a modified Rankin Scale (mRS) score of 0–1, afterroutine medical care coupled with placebo or DHI for 90 days was unclear. According to previous studies^15,16^, the proportion of patients who had a mRS score of 0–1 was about 28.7–45.2 % when only treated with placebo, so we estimate the proportion is 45 % when placebo coupled withroutine medical care are used. In this trial, it is hypothesized that an increase of at least 10 % is of clinical significance for the DHI group. An adaptive design was selected and EAST5.2 software was used to calculate the statistical sample size. After calculation, the number of subjects was initially estimated to be 703 (one-sided test, α =0.05, β =0.2). To allow for a dropout rate of 20 %, a total of 846 patients will be recruited. According to the adaptive design, the sample size may be adjusted after the interim analyses, which will be carried out when one-third (282) and two thirds (564) of patients have completed the trial, respectively. With a 2:1 randomization ratio, the number of participants in the DHI group is 564 and that in the control group is 282.

## 3.5 Study Schema

| **Period** | **Baseline** | **Treatment period** | | **Follow-up period** | | |
| --- | --- | --- | --- | --- | --- | --- |
| Day | 0 | 7±2 | 14±2 | 30±5 | 60±5 | 90±5 |
| Informed consent | × |  |  |  |  |  |
| Inclusion/exclusion criteria | × |  |  |  |  |  |
| Medical history | × |  |  |  |  |  |
| UCG | × |  |  |  |  |  |
| TOAST | × |  |  |  |  |  |
| ASPECTS | × |  |  |  |  |  |
| Medical examination | × | × | × | × | × | × |
| Concomitant medications | × | × | × | × | × | × |
| **Outcomes** |  |  |  |  |  |  |
| BI scale | × | × | × | × | × | × |
| mRS scale | × | × | × | × | × | × |
| NIHSS | × | × | × | × | × | × |
| Efficacy evaluation scale of Chinese Medicine Symptom in “Xueyu Zheng” | × | × | × | × | × | × |
| EQ-5D scale | × | × | × | × | × | × |
| Global disability on mRS |  |  |  |  |  | × |
| New-onset major vascular events |  |  |  |  |  | × |
| Overall mortality |  |  |  |  |  | × |
| Incidence of severe hemorrhages |  |  |  |  |  | × |
| Incidence of moderate hemorrhages |  |  |  |  |  | × |
| AEs and SAEs |  | × | × | × | × | × |
| Profiles of micro-RNA in 96 patients in certain centers | × |  |  | × |  | × |
| Profiles of mRNA in 96 patients in certain centers | × |  |  | × |  | × |

*UCG: ultrasound cardiogram; TOAST: Trial of Org 10 172 in Acute Stroke Treatment; ASPECTS: Alberta Stroke Programme Early CT Score; BI: Barthel Index; mRS: modified Rankin Scale; NIHSS: National Institutes of Health Stroke Scale; AEs: adverse events; SAEs: serious adverse events; microRNA: micro-ribonucleic acid; mRNA: messenger ribonucleic acid.

**Figure 1.** The schedule of enrollment, interventions, and assessments

# 5. Data Monitoring and Interim Analyses

## 4.1 Data Management

(1) All data will be recorded by trained clinical investigators using standardized electronic case report forms in Brightech clinical information management systems (Brightech, Somerset, NJ, USA).

(2) The accuracy and reliability of the data will be ensured by the study monitor, who will verify and cross-check the electronic case report forms against the investigator’s records (source document verification), using the maintenance of a drug dispensing log by the investigator.

(3) Front-end checks in the electronic case report forms and back-end checks in the clinical information management system will be used as a validation check of the data.

(4) If there are any discrepancies in the electronic case report forms, the results will be sent to the investigator for resolution.
(5) The results of the analysis must not be released with individual identification of the subjects until the database is closed.

## 4.2 Establishment of Three Committees for DHI Phase IV Trials

To ensure the effectiveness and integrity of the trial design, three committees have been established by the multicentre trial coordination group: the Clinical Trial Steering Committee, the data monitoring committee (DMC) and the Outcome Evaluation Committee. The first committee is responsible for the study design and the implementation process. The DMC will supervise the integrity and accuracy of data collection to control its quality. The Outcome Evaluation Committee will evaluate key outcomes (including outcome measurements and adverse events) based on their clinical expertise.

## 4.3 Interim Analyses

# Because this is an adaptive trial, two interim analyses are prospectively planned, and these will be performed in an unblinded manner by an independent statistician in the data monitoring committee (DMC) support group after one-third and two-thirds of the patients, respectively, have completed the trial. The statistical results of the interim analyses will be discussed in a close meeting of the DMC, who will decide on the re-estimation of sample size and determine whether any subsequent modifications must be made to the trial protocol.

# 5. Statistical Considerations

## 5.1 Study Hypothesis

The primary study hypothesis is that an initial usage of DHI added to routine medical care could bring more clinical benefit for the patients with acute ischemic stroke than an initial strategy of routine medical care alone.

## 5.2 Study Populations

All patients with randomization will be included in the analysis set regardless of whether they receive any treatment. Most outcome measurements (except micro-RNA and mRNA profiles) will be analyzed on the intention-to-treat (ITT) and per-protocol population (PPS). Safety analysis will be performed in a safety set, which is defined as a subset of subjects who were randomized and who received at least one treatment.

## 5.3 Methods of Statistical Analyses

### 5.3.1 The General Principle

The statistical analysis will include the distribution of subjects, baseline characteristics of participants, compliance and concomitant medication, efficacy analysis and safety analysis.

For continuous variables, means and standard deviations will be presented, unless the variable has a skewed distribution, in which case medians, 25th and 75th percentiles will be presented.

For categorical variables, the number and percentage of participants within each category will be presented. For each variable (continuous or categorical), the number of missing values will be reported.

The test statistics and corresponding P values will be presented for certain test. The P value should be directly reported by Fisher test. All reported P values are two-sided. A P value less than 0.05 is considered to be statistically significant. The odds ratios (ORs) and 95% confidence intervals (CIs) from logistic regression models will be also reported.

### 5.3.2 Derived data

(1) Age= INT((“enrollment date” - “date of birth” +1)/365.25);

(2) BMI= weight(kg)/height(m)^2^;

(3) Global disability: defined as a score of 3-5 on the mRS;

### 5.3.3 The Management of Missing data

Regardless of any violations, compliance or early withdrawal from the trial, if the patient is randomized, her data will be analyzed in our primary outcome analyses. Missing data of the primary outcome were handled using the baseline-observation-carried-forward (BOCF) approach, while missing data of secondary outcomes were not imputed.

### 5.3.4 Statistical Comparisons between Groups

### 5.3.4.1 Distribution of participants

The enrollment, completion and reasons for early withdrawal of the participants should be summarized by group. The detail on the early withdrawal and protocol deviation of participants will be listed. Analytical statistics will be calculated to estimate the difference in the number of participants who have been enrolled, who have completed or who have been withdrawn from the trial as well as dropout rate between groups.

### 5.3.4.2 Demographics and Baseline Characteristics

Baseline characteristics in each group will be analyzed using descriptive statistics, including means or medians for continuous variables and percentages for categorical variables.

### 5.3.4.3 Compliance and concomitant medication

Compliance analysis will be based on ITT population, and analysis of concomitant medications will be based on safety sets.

### 5.3.4.4 Analyses for Primary Outcome

The chi-square test will be used to compare the proportion of patients with excellent functional outcome, defined as a score of 0–1 on the mRS at day 90 between the two groups.

The distribution of mRS scores at baseline and after 90 days will be analyzed by Wilcoxon rank-sum test.

The estimate of primary outcome was adjusted for covariates (age, baseline mRS, baseline NIHSS score, baseline ASPECTS, time from stroke onset to randomization, history of previous stroke or TIA, hypertension, hyperlipidemia, diabetes mellitus, ischemic heart disease).

The adjusted and unadjusted odds ratios (ORs) and 95% confidence intervals (CIs) should be reported from multiple logistic regression models.

**Subgroup Analyses for the Primary Outcome**

The primary outcome with respect to health status for prespecified subgroups according to age, sex, body mass index (BMI), baseline NIHSS score, baseline ASPECTS, time from stroke onset to treatment, ischemic location, history of previous diseases and some important combination therapies will be analyzed by covariance, Cox regression or logistic regression.

### 5.3.4.5 Analyses for Secondary Outcomes

(1). The chi-square test will be used to compare the proportions of patients with syndrome improvement in *Xueyue Zheng* at 90 days between the two groups. Significant syndrome improvement is defined as at least 70% reduction in the *Xueyue Zheng score* from baseline.

(2). The chi-square test will be used to compare the proportions of patients with the Barthel Index (BI) score of ≥90 at 90 days between the two groups.

(3) The chi-square test will be used to compare the proportion of patients with an improvement in the NIHSS score of ≥4 or the NIHSS score of 0-1 at 90 days between the two groups.

(4) Mean data for EQ-5D scale TTO value at 90 days will be analyzed with the use of two sample t-tests or Kruskal-Wallis test between the two groups..

(6) The chi-square test will be used to compare the proportion of patients with global disability at 90 days between the two groups.

(7) Incidence of new-onset major vascular events within 90 days will be analyzed with the chi-square test or Fisher’s exact test between the two groups.

### 5.3.4.6 Other Pre-specified Efficacy Outcomes

Changes in the micro-RNA and mRNA profiles will be analyzed by Beijing Genomics Institute (BGI), which is not included in this statistical analysis plan.

### 5.3.4.7 Safety Analyses

(1) Overall mortality within 90 days will be analyzed with the Chi-square test or Fisher’s exact test between the two groups.

(2) Incidence of severe haemorrhages within 90 days will be analyzed with the Chi-square test or Fisher’s exact test between the two groups.

(3) Incidence of moderate haemorrhages within 90 days will be analyzed with the Chi-square test or Fisher’s exact test between the two groups.

(4) All adverse events and serious adverse events will be listed. Chi-square test or Fisher’s exact test will be used to compare the incidence of adverse events between the two groups. P-value will not be corrected for multiple tests.

**5.3.5 Interim Analyses**

Two interim analyses will be performed in a blinded manner after one-third (282) and two-thirds (564) of the patients, respectively. The statistical results of the interim analyses will be performed by independent statistician in the data monitoring committee. Because the two interim analyses may lead to an increased possibility of a type I error, Lan-DeMets alpha spending function with an O’Brien-Fleming boundary will be applied to adjust the results (Table 1).

**Table 1 The α spending using an O’BrienFleming boundary in the two inter analyses**

| *K* | $t_{k}$ | $Z_{k}$ | $\alpha_{k}$(single-side) | $\alpha_{k}$ (two-side) |
| --- | --- | --- | --- | --- |
| 3 | 1/3 | 3.471 | 0.00025 | 0.0005 |
|  | 2/3 | 2.454 | 0.00725 | 0.0145 |
|  | 3/3 | 2.004 | 0.0225 | 0.045 |

### 5.3.6 Analysis Software

For all statistical analyses, SAS 9.1.3 software will be used.

**References**

1.Matsuo R, Kamouchi M, Ago T, Hata J, Shono Y, Kuroda J, et al. Thrombolytic therapy with intravenous recombinant tissue plasminogen activator in Japanese older patients with acute ischemic stroke: the Fukuoka Stroke Registry. Geriatr Gerontol Int. 2014; 14:954–9.

2. Chen J, Venkat P, Zacharek A, Chopp M. Neurorestorative therapy for stroke. Front Hum Neurosci. 2014; 8:382.

3. Turner RC, Lucke-Wold B, Lucke-Wold N, Elliott AS, Logsdon AF, Rosen CL, et al. Neuroprotection for ischemic stroke: moving past shortcomings and identifying promising directions. Int J Mol Sci. 2013; 14:1890–917.

4. Behravan E, Razavi BM, Hosseinzadeh H. Review of plants and their constituents in the therapy of cerebral ischemia. Phytother Res. 2014; 28:1265–74.

5. Li X, Tang J, Meng F, Li C, Xie Y. Study on 10 409 cases of post-marketing safety Danhong injection centralized monitoring of hospital. Zhong guo Zhong Yao Za Zhi. 2011; 36:2783–5.

6. He Y, Wan H, Du Y, Bie X, Zhao T, Fu W, et al. Protective effect of Danhong injection on cerebral ischemia-reperfusion injury in rats. J Ethnopharmacol. 2012;144:387-94.

7. Xiujuan Mi, Guangqin Li. Effect of Danhong on Neural Functional Recovery after Focal Cerebral Ischemia in Rats and Its Mechanism. Zhongguo Quanke Yixue. 2010;13:870-3.

8. He Y, Wan H, Du Y, Bie X, Zhao T, Fu W, et al. Protective effect of Danhong injection on cerebral ischemia-reperfusion injury in rats. J Ethnopharmacol. 2012;144:387–94.

9. Ma L, Wang S, Li K. A systematic review of Danhong injection treatment for acute cerebral ischemic stroke and the safety of this treatment. Journal of Emergency in Traditional Chinese Medicine. 2012; 10:1609–10.

10.Chinese Society of Neurology. China Guideline for the Diagnosis and Treatment of Acute Ischemic Stroke. Chin J Neurol. 2010; 43:146–53.

11. Zheng X. Guidelines of Clinical Research of New Drugs of Traditional Chinese Medicine. Beijing: China Medical Science Press; 2002.

12. Brain disease emergency cooperation group of State Administration of traditional Chinese Medicine, Criteria For Diagnosis And Curative Effect Evaluation Of Stroke, 1996,19(1):55-56

13. Ying Gao, Bin Ma, Qiang Liu, Yongyan Wang. Development and methodology discussion of the diagnostic scale of ischemic stroke syndrome elements. Chinese Journal of Traditional Chinese Medicine, 2011, 52(24): 2097-2101.

14. van Swieten JC, Koudstaal PJ, Visser MC, Schouten HJ, van Gijn J.Interobserver agreement for the assessment of handicap in stroke patients.Stroke. 1988; 19:604–7

15.Shuaib A, Lees KR, Lyden P, Grotta J, Davalos A, Davis SM, et al. NXY-059 for the treatment of acute ischemic stroke. N Engl J Med. 2007; 357:562–71.

16. Hacke W, Kaste M, Bluhmki E, Brozman M, Davalos A, Guidetti D, et al. Thrombolysis with alteplase 3 to 4.5 hours after acute ischemic stroke. N Engl J Med. 2008; 359:1317–29.

**Danhong Injection for the treatment of acute ischemic stroke (Phase IV): an adaptive-design, randomized, multi-center, double-blind, placebo-controlled clinical trial**

**(Danhong Phase IV, DH20120423)**

**Statistical Analysis Plan**

**The Final Version: August 20, 2018**

**Prepared for and approved by the Clinical Trial Steering Committee of the study:**

Prof. Yong-Yan Wang (Chair), Institute of Basic Research in Clinical Medicine, China Academy of Chinese Medical Sciences

Prof. Xiao-Xi Du (Member), China Food and Drug Administration

Prof. Bo-Li Zhang (Member), China Academy of Chinese Medical Sciences

Prof. Run-Lin Gao (Member), Fuwai Hospital Chinese Academy of Medical Sciences

Prof. Wei-Liang Weng (Member), Xi-yuan Hospital Affiliated to China Academy of Chinese Medical Sciences

Dr. Ai-Ping Lv (Member), School of Chinese Medicine, Hong Kong Baptist University

Prof. Ming-Hui Yang (Member), Chinese PLA General Hospital

**Prepared for Data Monitoring Committee**

Prof. Chun-Ti Shen (Chair), Changzhou TCM Hospital Affiliated to Nanjing University of Chinese Medicine

Prof. Qi-Guang Chen (Member, independent statistician), Southeast University

Prof. Hua-Qiang Zhang (Member), Jiangsu Provincial Commission of Health and Family Planning

Dr. Hong-Wu Wang (Member), Tianjin University of Traditional Chinese Medicine

Dr. Xiao-Chun Sun (Member), Beijing Kelunrui Pharmaceutical Technology Co. Ltd.

Prof. Cheng-Shu Wang (Member), Zhongda Hospital Southeast University

Dr. Hai-Xia Dang (Member), China Academy of Chinese Medical Sciences

Dr. Jun Liu (Member), Institute of Basic Research in Clinical Medicine, China Academy of Chinese Medical Sciences

**Prepared by an Independent Statistician:**

Dr. Bing-Wei Chen (Statistician), Southeast University

**Table of Contents**

[1. Introduction	- 1 -](#_Toc7909)

[2. Study Objective	- 2 -](#_Toc29582)

[3. Design	- 2 -](#_Toc21267)

[3.1 Overview	- 2 -](#_Toc18548)

[3.2 Eligibility criteria	- 2 -](#_Toc26419)

[3.2.1 Diagnostic criteria	- 3 -](#_Toc7741)

[3.2.3 Exclusion Criteria	- 4 -](#_Toc20347)

[3.3 Efficacy and Safety Outcomes	- 5 -](#_Toc19172)

[3.3.1 Primary Efficacy Outcome	- 5 -](#_Toc1924)

[3.3.2 Secondary Efficacy Outcomes	- 5 -](#_Toc8667)

[3.3.3 Other Pre-specified Efficacy Outcomes	- 6 -](#_Toc17571)

[3.3.4 Safety Outcomes	- 6 -](#_Toc15085)

[3.4 Sample Size Calculation	- 7 -](#_Toc17055)

[3.5 Study Schema	- 8 -](#_Toc17304)

[4. Data Monitoring and Interim Analyses	- 10 -](#_Toc18478)

[4.1 Data Management	- 10 -](#_Toc29917)

[4.2 Establishment of Three Committees for DHI Phase IV Trials	- 10 -](#_Toc3242)

[4.3 Interim Analyses	- 11 -](#_Toc17443)

[5. Statistical Considerations	- 11 -](#_Toc2680)

[5.1 Study Hypothesis	- 11 -](#_Toc5541)

[5.2 Study Populations	- 11 -](#_Toc1558)

[5.3 Methods of Statistical Analyses	- 12 -](#_Toc19596)

[5.3.1 The General Principle	- 12 -](#_Toc2239)

[5.3.2 Derived data	- 12 -](#_Toc24726)

[5.3.3 The Management of Missing data	- 12 -](#_Toc16942)

[5.3.4 Statistical Comparisons between Groups	- 13 -](#_Toc30396)

[5.3.4.1 Distribution of participants	- 13 -](#_Toc20219)

[5.3.4.2 Demographics and Baseline Characteristics	- 13 -](#_Toc7860)

[5.3.4.3 Compliance and concomitant medication	- 13 -](#_Toc14234)

[5.3.4.4 Analyses for Primary Outcome	- 13 -](#_Toc24683)

[5.3.4.5 Analyses for Secondary Outcomes	- 14 -](#_Toc10335)

[5.3.4.6 Other Pre-specified Efficacy Outcomes	- 15 -](#_Toc29306)

[5.3.4.7 Safety Analyses	- 15 -](#_Toc12983)

[5.3.5 Interim Analyses	- 16 -](#_Toc18696)

[5.3.6 Analysis Software	- 17 -](#_Toc10473)

[References	- 17 -](#_Toc26874)

# 1. Introduction

Acute ischemic stroke (AIS), characterized by an occlusion of the cerebral artery, is one of the leading causes of mortality, long-term disability, and morbidity. Thrombolytic therapy with intravenous tissue plasminogen activator (tPA), which remains the only therapeutic drug for AIS approved by the US Food and Drug Administration, is limited by the narrow time window of thrombolysis, bleeding complications, or high costs^1^. Other treatment strategies mainly utilize therapeutic agents to prevent or reduce cell damage from ischemia^2^. Hundreds of neuro protective agents for ischemic stroke have reached clinical trials, but no optimal drugs for the clinical treatment of AIS have yet been developed^3^. This leads us to search for novel therapeutic approaches, and to investigate whether natural compounds extracted from herbs may be an alternative option to improve treatment for AIS^4^.

Danhong injection (DHI), extracted from Radix Salviae miltiorrhizae (Danshen) and Flos Carthami tinctorii (Honghua), is a Chinese medicinal product approved by the China Food and Drug Administration (CFDA), and it is widely used in China for treating AIS^5^. Previous studies demonstrated the neuroprotective efficacy of DHI in rat models with cerebral ischemic-reperfusion injury^6, 7^, and that the underlying pharmacological mechanisms might involve anti-inflammatory, anti-coagulant, antithrombotic, antifibrinolytic, and antioxidant activities^8^. A number of clinical studies also supported the efficacy and safety of DHI in the management of AIS, but due to the methodological deficiencies of inadequate randomization, no double blinding, no placebo control, or incomplete outcome data, the quality of these studies was assessed as generally low^9^. There is a lack of critically appraised evidence such as well designed randomized controlled trials (RCTs) to justify its clinical use and recommendation. Thus, we aim to investigate the efficacy and safety of DHI in the treatment of AIS in a well-designed, randomized, multicenter, double-blind and placebo-controlled trial.

# 2. Study Objective

The objective of this study is mainly to determine whether Danhong injection is effective in the treatment of acute ischemic stroke.

# 3. Design

## 3.1 Overview

This study is a three-stage adaptive-design, randomized, multicentre, double-blind, placebo-controlled trial. The number of subjects is initially estimated a total of 846 which 564 subjects are randomly assigned to in DHI group and 282 subjects in the control group. After the second interim analysis, the sample size was enlarged to be 1513, and therefore, 1008 will be enrolled in Danhong group and 505 in control group. Both groups receive routine medical therapy as recent guideline advocated. Subjects in the DHI group are treated by DHI (40 ml, qd) plus 0.9% normal saline (250 ml, ivgtt, qd), while subjects in the control group are treated by DHI placebo (0.9% normal saline, 40ml, qd) plus 0.9% normal saline (250 ml, ivgtt, qd).

## 3.2 Eligibility criteria

Eligible subjects are those who meet all of the following inclusion criteria and who do not have any of the listed exclusion criterion.

### 3.2.1 Diagnostic criteria

(1). The diagnostic criteria for acute ischemic stroke is determined according to the Chinese Guidelines for the Diagnosis and Treatment of Acute Ischemic Stroke (2010)^10^.

(2). The diagnostic criteria for “Xueyu Zheng” (Blood stasis syndrome) is determined according to the Guidelines for Clinical Research of New Drugs of Traditional Chinese Medicine (2002)^11^, Guideline for Diagnosis and Effect Evaluation of Stroke (1996)^12^ and Zhenghou Diagnostic Scale in Ischemic Stroke^13^.

**3.2.2 Inclusion Criteria**

(1). Female or male inpatients.

(2). Age: 18–70 years.

(3). Clinical diagnosis of ischemic stroke causing a measurable neurological deficit defined as impairment of language, motor function, cognition and/or gaze, vision or neglect. Ischemic stroke is defined as an event characterized by the sudden onset of an acute focal neurologic deficit presumed to be due to cerebral ischemia after computed tomography (CT) scan excludes hemorrhage.

(4). Clinical diagnosis of "*Xueyu Zheng*" (Blood Stasis Syndrome) as the scores of Chinese Medicine Symptom Diagnostic Scale of "*Xueyu Zheng*" in ischemic stroke ≥ 20.

(5). Onset of symptoms within 1 week prior to initiation of study drug administration.

(6). Patients with a clinical diagnosis of cerebral anterior circulation obstruction.

(7). 4≤National Institutes of Health Stroke Scale (NIHSS) score <20.

(8). Patient is willing to participate voluntarily and to sign a written patient informed consent. Informed consent will be obtained from each patient or the subject's legally authorized representative or relatives.

### 3.2.3 Exclusion Criteria

(1). Evidence of intracranial hemorrhage (ICH) or other cerebral diseases (e.g., vascular malformation, tumor, abscess or multiple sclerosis) on the CT or magnetic resonance imaging (MRI) scan.

(2). Patients who underwent thrombolysis or endovascular treatment.

(3). Known history of allergy or suspected allergy to the study drug.

(4). Fasting blood glucose <2.8 or >16.8 mmol/l or with severe complications due to diabetes (e.g., peripheral neuropathy, or diabetic gangrene).

(5). Liver function impairment with the value of alanine aminotransferase (ALT) or aspartate aminotransaminase (AST) over 1.5-fold the upper limit of normal range.

(6). Renal dysfunction with the value of serum creatinine over 1.5-fold the upper limit of normal range.

(7). Severe cardiac dysfunction on echocardiogram or the New York Heart Association (NYHA) heart functional class III or IV.

(8). History of prior stroke with a modified Rankin Scale (mRS) score ≥2.

(9). Complicated with atrial fibrillation.

(10). Severe stroke as assessed by appropriate imaging techniques (e.g., massive cerebral infarction affecting more than one lobe of the brain or over one-third of the blood-supply area of the middle cerebral artery).

(11). Physical disability before onset.

(12).Hemorrhagic tendency or severe or dangerous bleeding within the prior 3 months.

(13).Suspected alcohol addiction or drug abuse.

(14).Severe complications that would make the condition more complicated as assessed by the investigator.

(15).Women who are pregnant, lactating or have a positive pregnancy test, or women who plan to become pregnant in the subsequent 6 months.

(16).Women who have a menstrual period at baseline.

## (17).Patients who are participating in other trials or have participated in other trials within the prior 3 months.

## 3.3 Efficacy and Safety Outcomes

### 3.3.1 Primary Efficacy Outcome

The primary outcome is the proportion of patients with a favorable outcome, defined as a score of 0–1 on the mRS^14^ at day 90. The mRS is a commonly used scale for measuring the degree of disability or dependence in the daily activities of people who have suffered a stroke or other causes of neurological disability. The scale runs from 0–6, ranging from perfect health without symptoms to death.

### 3.3.2 Secondary Efficacy Outcomes

(1). The proportion of patients with a significant improvement in “*Xueyu Zheng*”, defined as at least 70% reduction in the Efficacy Evaluation Scale of Chinese Medicine Symptom in “*Xueyu Zheng*”;

(2). The proportion of patients with the Barthel Index (BI) score of ≥90;

(3) The proportion of patients with an improvement in the NIHSS score of ≥4 or the NIHSS score of 0-1;

(4) The quality of life measured by the EQ-5D scale using the standardized Time Trade-off (TTO) value of Chinese population range from 0.1702 to 1.00^15^, with higher scores indicating a better quality of life;

(5) Global disability (mRS: 3-5) at 90 days;

(6) Incidence of new-onset major vascular events within 90 days.

### 3.3.3 Other Pre-specified Efficacy Outcomes

(1) The changes on the profiles of micro-RNA in 96 patients selected from certain centers；

(2) The changes on the expression profiles of mRNA in 96 patients selected from certain centers.

### The statistical analysis of these outcomes are not included in this statistical analysis plan, which will be shown in the RNA sequencing protocol for the mechanism of Danhong injection in the treatment of ischemic stroke.

### 3.3.4 Safety Outcomes

(1) Overall mortality within 90 days;

(2) Incidence of severe haemorrhages within 90 days;

(3) Incidence of moderate haemorrhages within 90 days;

(4) Incidence of adverse and serious adverse events.

## 3.4 Sample Size Calculation

**3.4.1 Initial estimation of sample size**

The proportion of patients who had a favorable outcome, defined as a modified Rankin Scale (mRS) score of 0–1, afterroutine medical care coupled with placebo or DHI for 90 days was unclear. According to previous studies^16,17^, the proportion of patients who had a mRS score of 0–1 was about 28.7–45.2 % when only treated with placebo, so we estimate the proportion is 45 % when placebo coupled withroutine medical care are used. In this trial, it is hypothesized that an increase of at least 10 % is of clinical significance for the DHI group. An adaptive design was selected and EAST 5.2 software was used to calculate the statistical sample size. After calculation, the number of subjects was initially estimated to be 703 (one-sided test, α =0.05, β =0.2). To allow for a dropout rate of 20 %, a total of 846 patients will be recruited. According to the adaptive design, the sample size may be adjusted after the interim analyses, which will be carried out when one-third (282) and two thirds (564) of patients have completed the trial, respectively. With a 2:1 randomization ratio, the number of participants in the DHI group is 564 and that in the control group is 282.

**3.4.2 Sample size re-estimation**

At the first interim analysis, 282 patients completed the trial. In the DHI group, 106 out of 188 patients (56.50%) achieved excellent functional outcome (an mRS score of 0-1 compared to 46 out of 94 patients (48.94%) in the placebo group (Z_1_ = 1.19, P_1_ = 0.23). According to the recommendation of DMC, the trial continued without sample size reassessment.

At the second interim analysis, 564 patients completed the trial. In the DHI group, 60.94% of patients achieved excellent functional outcome (an mRS score of 0-1) compared to 54.82% of patients in the placebo group (Z_2_ = 1.41, P_2_ = 0.16). The study did not reach the stopping boundary; therefore the sample size was estimated again to be 1261, inflated approximately 20% to a maximum of 1513 patients to account for potential losses to follow-up and dropouts (Table 1). Therefore, according to the recommendation of DMC, the sample size will be enlarged to be 1513, in which 1008 will be enrolled in Danhong group and 505 in control group.

**Table 1 The treatment effect on the primary outcome in the two interim analyses in the intention-to-treat and the per-protocol populations***

| **Interim analysis** | **Populations** | **DHI（%）** | **Control（%）** | **Difference（%）** | **Z-value** | **P-value** |
| --- | --- | --- | --- | --- | --- | --- |
| 1 | ITT | 56.50 | 48.94 | 7.56 | 1.19 | 0.23 |
|  | PPS | 58.24 | 49.46 | 10.38 | 1.37 | 0.17 |
| 2 | ITT | 60.94 | 54.82 | 6.12 | 1.41 | 0.16 |
|  | PPS | 62.43 | 55.03 | 7.40 | 1.67 | 0.09 |

* DHI:Danhong injection; ITT=intention-to-treat; PPS=per-protocol set. The difference between two groups were done using chi-square test.

## 3.5 Study Schema

| **Period** | **Baseline** | **Treatment period** | | **Follow-up period** | | |
| --- | --- | --- | --- | --- | --- | --- |
| Day | 0 | 7±2 | 14±2 | 30±5 | 60±5 | 90±5 |
| Informed consent | × |  |  |  |  |  |
| Inclusion/exclusion criteria | × |  |  |  |  |  |
| Medical history | × |  |  |  |  |  |
| UCG | × |  |  |  |  |  |
| TOAST | × |  |  |  |  |  |
| ASPECTS | × |  |  |  |  |  |
| Medical examination | × | × | × | × | × | × |
| Concomitant medications | × | × | × | × | × | × |
| **Outcomes** |  |  |  |  |  |  |
| BI scale | × | × | × | × | × | × |
| mRS scale | × | × | × | × | × | × |
| NIHSS | × | × | × | × | × | × |
| Efficacy evaluation scale of Chinese Medicine Symptom in “*Xueyu Zheng*” | × | × | × | × | × | × |
| EQ-5D scale | × | × | × | × | × | × |
| Global disability on mRS |  |  |  |  |  | × |
| New-onset major vascular events |  |  |  |  |  | × |
| Overall mortality |  |  |  |  |  | × |
| Incidence of severe hemorrhages |  |  |  |  |  | × |
| Incidence of moderate hemorrhages |  |  |  |  |  | × |
| AEs and SAEs |  | × | × | × | × | × |
| Profiles of micro-RNA in 96 patients in certain centers | × |  |  | × |  | × |
| Profiles of mRNA in 96 patients in certain centers | × |  |  | × |  | × |

**Figure 1.** The schedule of enrollment, interventions, and assessments

# 4. Data Monitoring and Interim Analyses

## 4.1 Data Management

(1) All data will be recorded by trained clinical investigators using standardized electronic case report forms in Brightech clinical information management systems (Brightech, Somerset, NJ, USA).

(2) The accuracy and reliability of the data will be ensured by the study monitor, who will verify and cross-check the electronic case report forms against the investigator’s records (source document verification), using the maintenance of a drug dispensing log by the investigator.

(3) Front-end checks in the electronic case report forms and back-end checks in the clinical information management system will be used as a validation check of the data.

(6) If there are any discrepancies in the electronic case report forms, the results will be sent to the investigator for resolution.
(7) The results of the analysis must not be released with individual identification of the subjects until the database is closed.

## 4.2 Establishment of Three Committees for DHI Phase IV Trials

To ensure the effectiveness and integrity of the trial design, three committees have been established by the multicentre trial coordination group: the Clinical Trial Steering Committee, the data monitoring committee (DMC) and the Outcome Evaluation Committee. The first committee is responsible for the study design and the implementation process. The DMC will supervise the integrity and accuracy of data collection to control its quality. The Outcome Evaluation Committee will evaluate key outcomes (including outcome measurements and adverse events) based on their clinical expertise.

## 4.3 Interim Analyses

# Because this is an adaptive trial, two interim analyses are prospectively planned, and these will be performed in an unblinded manner by an independent statistician in the data monitoring committee (DMC) support group after one-third and two-thirds of the patients, respectively, have completed the trial. The statistical results of the interim analyses will be discussed in a close meeting of the DMC, who will decide on the re-estimation of sample size and determine whether any subsequent modifications must be made to the trial protocol.

# 5. Statistical Considerations

## 5.1 Study Hypothesis

The primary study hypothesis is that an initial usage of DHI added to routine medical care could bring more clinical benefit for the patients with acute ischemic stroke than an initial strategy of routine medical care alone.

## 5.2 Study Populations

All patients with randomization will be included in the analysis set regardless of whether they receive any treatment. Most outcome measurements (except micro-RNA and mRNA profiles) will be analyzed on the intention-to-treat (ITT) and per-protocol population (PPS). Safety analysis will be performed in a safety set, which is defined as a subset of subjects who were randomized and who received at least one treatment.

## 5.3 Methods of Statistical Analyses

### 5.3.1 The General Principle

The statistical analysis will include the distribution of subjects, baseline characteristics of participants, compliance and concomitant medication, efficacy analysis and safety analysis.

For continuous variables, means and standard deviations will be presented, unless the variable has a skewed distribution, in which case medians, 25th and 75th percentiles will be presented.

For categorical variables, the number and percentage of participants within each category will be presented. For each variable (continuous or categorical), the number of missing values will be reported.

The test statistics and corresponding P values will be presented for certain test. The P value should be directly reported by Fisher test. All reported P values are two-sided. A P value less than 0.05 is considered to be statistically significant. The odds ratios (ORs) and 95% confidence intervals (CIs) from logistic regression models will be also reported.

### 5.3.2 Derived data

(1) Age= INT((“enrollment date” - “date of birth” +1)/365.25);

(2) BMI= weight(kg)/height(m)^2^;

(3) Global disability: defined as a score of 3-5 on the mRS;

### 5.3.3 The Management of Missing data

Regardless of any violations, compliance or early withdrawal from the trial, if the patient is randomized, her data will be analyzed in our primary outcome analyses. Missing data of the primary outcome were handled using the baseline-observation-carried-forward (BOCF) approach, while missing data of secondary outcomes were not imputed.

### 5.3.4 Statistical Comparisons between Groups

### 5.3.4.1 Distribution of participants

The enrollment, completion and reasons for early withdrawal of the participants should be summarized by group. The detail on the early withdrawal and protocol deviation of participants will be listed. Analytical statistics will be calculated to estimate the difference in the number of participants who have been enrolled, who have completed or who have been withdrawn from the trial as well as dropout rate between groups.

### 5.3.4.2 Demographics and Baseline Characteristics

Baseline characteristics in each group will be analyzed using descriptive statistics, including means or medians for continuous variables and percentages for categorical variables.

### 5.3.4.3 Compliance and concomitant medication

Compliance analysis will be based on ITT population, and analysis of concomitant medications will be based on safety sets.

### 5.3.4.4 Analyses for Primary Outcome

The chi-square test will be used to compare the proportion of patients with excellent functional outcome, defined as a score of 0–1 on the mRS at day 90 between the two groups.

The distribution of mRS scores at baseline and after 90 days will be analyzed by Wilcoxon rank-sum test.

The estimate of primary outcome was adjusted for covariates (age, baseline mRS, baseline NIHSS score, baseline ASPECTS, time from stroke onset to randomization, history of previous stroke or TIA, hypertension, hyperlipidemia, diabetes mellitus, ischemic heart disease and ECG showing atrial fibrillation).

The adjusted and unadjusted odds ratios (ORs) and 95% confidence intervals (CIs) should be reported from multiple logistic regression models.

**Subgroup Analyses for the Primary Outcome**

The primary outcome with respect to health status for prespecified subgroups according to age, sex, body mass index (BMI), baseline NIHSS score, baseline ASPECTS, time from stroke onset to treatment, ischemic location, history of stroke or TIA, hypertension, hyperlipidemia, diabetes mellitus, ischemic heart disease and the concomitant administration of aspirin, clopidogrel, anticoagulants, neuroprotective agents, statins or calcium channel blockers (CCB) will be analyzed by logistic regression.

### 5.3.4.5 Analyses for Secondary Outcomes

(1). The chi-square test will be used to compare the proportions of patients with syndrome improvement in *Xueyue Zheng* at 90 days between the two groups. Significant syndrome improvement is defined as at least 70% reduction in the *Xueyue Zheng score* from baseline.

(2). The chi-square test will be used to compare the proportions of patients with the Barthel Index (BI) score of ≥90 at 90 days between the two groups.

(3) The chi-square test will be used to compare the proportion of patients with an improvement in the NIHSS score of ≥4 or the NIHSS score of 0-1 at 90 days between the two groups.

(4) Mean data for EQ-5D scale TTO value at 90 days will be analyzed with the use of two sample t-tests or Kruskal-Wallis test between the two groups..

(6) The chi-square test will be used to compare the proportion of patients with global disability at 90 days between the two groups.

(7) Incidence of new-onset major vascular events within 90 days will be analyzed with the chi-square test or Fisher’s exact test between the two groups.

### 5.3.4.6 Other Pre-specified Efficacy Outcomes

Changes in the micro-RNA and mRNA profiles will be analyzed by Beijing Genomics Institute (BGI), which is not included in this statistical analysis plan.

### 5.3.4.7 Safety Analyses

(1) Overall mortality within 90 days will be analyzed with the Chi-square test or Fisher’s exact test between the two groups.

(2) Incidence of severe haemorrhages within 90 days will be analyzed with the Chi-square test or Fisher’s exact test between the two groups.

(3) Incidence of moderate haemorrhages within 90 days will be analyzed with the Chi-square test or Fisher’s exact test between the two groups.

(4) All adverse events and serious adverse events will be listed. Chi-square test or Fisher’s exact test will be used to compare the incidence of adverse events between the two groups. P-value will not be corrected for multiple tests.

**5.3.5 Interim Analyses**

Two interim analyses will be performed in a blinded manner after one-third (282) and two-thirds (564) of the patients, respectively. The statistical results of the interim analyses will be performed by independent statistician in the data monitoring committee. Because the two interim analyses may lead to an increased possibility of a type I error, Lan-DeMets alpha spending function with an O’Brien-Fleming boundary will be applied to adjust the results (Table 2).

**Table 1 The α spending using an O’BrienFleming boundary in the two inter analyses**

| *K* | $t_{k}$ | $Z_{k}$ | $\alpha_{k}$(single-side) | $\alpha_{k}$ (two-side) |
| --- | --- | --- | --- | --- |
| 3 | 1/3 | 3.471 | 0.00025 | 0.0005 |
|  | 2/3 | 2.454 | 0.00725 | 0.0145 |
|  | 3/3 | 2.004 | 0.0225 | 0.045 |

**Interim Analyses Results**

At the first interim analysis, 282 patients completed the trial. In the DHI group, 106 out of 188 patients (56.50%) achieved excellent functional outcome (an mRS score of 0-1 compared to 46 out of 94 patients (48.94%) in the placebo group (Z_1_ = 1.19, P_1_ = 0.23). According to the recommendation of DMC, the trial continued without sample size reassessment.

At the second interim analysis, 564 patients completed the trial. In the DHI group, 60.94% of patients achieved excellent functional outcome (an mRS score of 0-1) compared to 54.82% of patients in the placebo group (Z_2_ = 1.41, P_2_ = 0.16). The study did not reach the stopping boundary; therefore the sample size was estimated again to be 1261, inflated approximately 20% to a maximum of 1513 patients to account for potential losses to follow-up and dropouts (Table 1). Therefore, according to the recommendation of DMC, the sample size will be enlarged to be 1513, in which 1008 will be enrolled in Danhong group and 505 in control group.

### 5.3.6 Analysis Software

For all statistical analyses, SAS 9.4 software will be used.

**References**

1.Matsuo R, Kamouchi M, Ago T, Hata J, Shono Y, Kuroda J, et al. Thrombolytic therapy with intravenous recombinant tissue plasminogen activator in Japanese older patients with acute ischemic stroke: the Fukuoka Stroke Registry. Geriatr Gerontol Int. 2014; 14:954–9.

2. Chen J, Venkat P, Zacharek A, Chopp M. Neurorestorative therapy for stroke. Front Hum Neurosci. 2014; 8:382.

3. Turner RC, Lucke-Wold B, Lucke-Wold N, Elliott AS, Logsdon AF, Rosen CL, et al. Neuroprotection for ischemic stroke: moving past shortcomings and identifying promising directions. Int J Mol Sci. 2013; 14:1890–917.

4. Behravan E, Razavi BM, Hosseinzadeh H. Review of plants and their constituents in the therapy of cerebral ischemia. Phytother Res. 2014; 28:1265–74.

5. Li X, Tang J, Meng F, Li C, Xie Y. Study on 10 409 cases of post-marketing safety Danhong injection centralized monitoring of hospital. Zhong guo Zhong Yao Za Zhi. 2011; 36:2783–5.

6. He Y, Wan H, Du Y, Bie X, Zhao T, Fu W, et al. Protective effect of Danhong injection on cerebral ischemia-reperfusion injury in rats. J Ethnopharmacol. 2012;144:387-94.

7. Xiujuan Mi, Guangqin Li. Effect of Danhong on Neural Functional Recovery after Focal Cerebral Ischemia in Rats and Its Mechanism. Zhongguo Quanke Yixue. 2010;13:870-3.

8. He Y, Wan H, Du Y, Bie X, Zhao T, Fu W, et al. Protective effect of Danhong injection on cerebral ischemia-reperfusion injury in rats. J Ethnopharmacol. 2012;144:387–94.

9. Ma L, Wang S, Li K. A systematic review of Danhong injection treatment for acute cerebral ischemic stroke and the safety of this treatment. Journal of Emergency in Traditional Chinese Medicine. 2012; 10:1609–10.

10.Chinese Society of Neurology. China Guideline for the Diagnosis and Treatment of Acute Ischemic Stroke. Chin J Neurol. 2010; 43:146–53.

11. Zheng X. Guidelines of Clinical Research of New Drugs of Traditional Chinese Medicine. Beijing: China Medical Science Press; 2002.

12. Brain disease emergency cooperation group of State Administration of traditional Chinese Medicine, Criteria For Diagnosis And Curative Effect Evaluation Of Stroke, 1996,19(1):55-56

13. Ying Gao, Bin Ma, Qiang Liu, Yongyan Wang. Development and methodology discussion of the diagnostic scale of ischemic stroke syndrome elements. Chinese Journal of Traditional Chinese Medicine, 2011, 52(24): 2097-2101.

14. van Swieten JC, Koudstaal PJ, Visser MC, Schouten HJ, van Gijn J.Interobserver agreement for the assessment of handicap in stroke patients.Stroke. 1988; 19:604–7

15. Zhuo L, Xu L, Ye J, et al. Time Trade-Off Value Set for EQ-5D-3L Based on a Nationally Representative Chinese Population Survey. Value Health, 2018 May 18. ahead of print. doi: 10.1016/j.jval.2018.04.1370.

16. Shuaib A, Lees KR, Lyden P, Grotta J, Davalos A, Davis SM, et al. NXY-059 for the treatment of acute ischemic stroke. N Engl J Med. 2007; 357:562–71.

17. Hacke W, Kaste M, Bluhmki E, Brozman M, Davalos A, Guidetti D, et al. Thrombolysis with alteplase 3 to 4.5 hours after acute ischemic stroke. N Engl J Med. 2008; 359:1317–29.

**SUMMARY OF CHANGES IN THE STATISTICAL ANALYSIS PLAN**

All changes in the final version appear in blue. Major changes include the following:

1. Since the Time Trade-Off (TTO) value set for EQ-5D-3L on Chinese population was published in May 2018, the TTO value of EQ-5D-3L should be updated in our study. We added the value in the final SAP, Please see in 3.3.2 Secondary Efficacy Outcomes.
2. After the second interim analysis, the sample size is expanded to 1513. The detailed information about the process of this change could be see in 3.4 Sample Size Calculation.
3. After the second interim analysis, we added the details for the subgroup anlysis in the history of previous disease and the combiantion therapies using in our study, see “Subgroup Analyses for the Primary Outcome ” in 5.3.4.4 Analyses for Primary Outcome.
4. After the second interim analysis, we added the “Interim Analyses Results” in 5.3.5 Interim Analyses.
